# Pyronaridine–artesunate real-world safety, tolerability, and effectiveness in malaria patients in 5 African countries: A single-arm, open-label, cohort event monitoring study

**DOI:** 10.1371/journal.pmed.1003669

**Published:** 2021-06-15

**Authors:** Gaston Tona Lutete, Ghyslain Mombo-Ngoma, Serge-Brice Assi, Jude D. Bigoga, Felix Koukouikila-Koussounda, Nsengi Y. Ntamabyaliro, Francine Ntoumi, Selidji T. Agnandji, Mirjam Groger, Jangsik Shin, Isabelle Borghini-Fuhrer, Sarah Arbe-Barnes, Stephen J. Allen, Peter G. Kremsner, Robert Miller, Stephan Duparc, Michael Ramharter, the CANTAM study group

**Affiliations:** 1 Unité de Pharmacologie Clinique et Pharmacovigilance (UPC-PV), University of Kinshasa, Kinshasa, Democratic Republic of Congo; 2 Centre de Recherches Médicales de Lambaréné, Lambaréné, Gabon; 3 Institut für Tropenmedizin, Reisemedizin und Humanparasitologie, University of Tübingen, Tübingen, Germany; 4 Department of Tropical Medicine, Bernhard Nocht Institute for Tropical Medicine and I. Department of Medicine, University Medical Center Hamburg-Eppendorf, Hamburg, Germany; 5 Institut Pierre Richet/Institut National de Santé Publique (IPR/INSP), Bouaké, Ivory Coast; 6 The Biotechnology Center, University of Yaounde I, Yaounde, Cameroon; 7 Fondation Congolaise pour la Recherche Médicale (FCRM), WHO-AFRO Campus Djoué, Brazzaville, Republic of Congo; 8 Shin-Poong Pharmaceutical Co. Ltd, Seoul, Korea; 9 Medicines for Malaria Venture, Geneva, Switzerland; 10 Artemida Pharma, Stevenage, United Kingdom; 11 Liverpool School of Tropical Medicine, Liverpool, United Kingdom; 12 German Center for Infection Research (DZIF), partner site Hamburg-Lübeck-Borstel-Riems, Hamburg, Germany; Mahidol Oxford Tropical Medicine Research Unit, Faculty of Tropical Medicine, Mahidol University, THAILAND

## Abstract

**Background:**

In Phase II/III randomized controlled clinical trials for the treatment of acute uncomplicated malaria, pyronaridine–artesunate demonstrated high efficacy and a safety profile consistent with that of comparators, except that asymptomatic, mainly mild-to-moderate transient increases in liver aminotransferases were reported for some patients. Hepatic safety, tolerability, and effectiveness have not been previously assessed under real-world conditions in Africa.

**Methods and findings:**

This single-arm, open-label, cohort event monitoring study was conducted at 6 health centers in Cameroon, Democratic Republic of Congo, Gabon, Ivory Coast, and Republic of Congo between June 2017 and April 2019. The trial protocol as closely as possible resembled real-world clinical practice for the treatment of malaria at the centers. Eligible patients were adults or children of either sex, weighing at least 5 kg, with acute uncomplicated malaria who did not have contraindications for pyronaridine–artesunate treatment as per the summary of product characteristics. Patients received fixed-dose pyronaridine–artesunate once daily for 3 days, dosed by body weight, without regard to food intake. A tablet formulation was used in adults and adolescents and a pediatric granule formulation in children and infants under 20 kg body weight. The primary outcome was the hepatic event incidence, defined as the appearance of the clinical signs and symptoms of hepatotoxicity confirmed by a >2× rise in alanine aminotransferase/aspartate aminotransferase (ALT/AST) versus baseline in patients with baseline ALT/AST >2× the upper limit of normal (ULN). As a secondary outcome, this was assessed in patients with ALT/AST >2× ULN prior to treatment versus a matched cohort of patients with normal baseline ALT/AST. The safety population comprised 7,154 patients, of mean age 13.9 years (standard deviation (SD) 14.6), around half of whom were male (3,569 [49.9%]). Patients experienced 8,560 malaria episodes; 158 occurred in patients with baseline ALT/AST elevations >2×ULN. No protocol-defined hepatic events occurred following pyronaridine–artesunate treatment of malaria patients with or without baseline hepatic dysfunction. Thus, no cohort comparison could be undertaken. Also, as postbaseline clinical chemistry was only performed where clinically indicated, postbaseline ALT/AST levels were not systematically assessed for all patients. Adverse events of any cause occurred in 20.8% (1,490/7,154) of patients, most frequently pyrexia (5.1% [366/7,154]) and vomiting (4.2% [303/7,154]). Adjusting for *Plasmodium falciparum* reinfection, clinical effectiveness at day 28 was 98.6% ([7,369/7,746] 95% confidence interval (CI) 98.3 to 98.9) in the per-protocol population. There was no indication that comorbidities or malnutrition adversely affected outcomes. The key study limitation was that postbaseline clinical biochemistry was only evaluated when clinically indicated.

**Conclusions:**

Pyronaridine–artesunate had good tolerability and effectiveness in a representative African population under conditions similar to everyday clinical practice. These findings support pyronaridine–artesunate as an operationally useful addition to the management of acute uncomplicated malaria.

**Trial registration:**

ClinicalTrials.gov NCT03201770.

## Abstract 2

Gaston Tona Lutete and co-workers report on safety and effectiveness of the antimalarial drug pyronaridine-artesunate in African countries.

## Author summary

**Why was this study done?:**

- Pyronaridine–artesunate has shown high efficacy and an acceptable safety profile in randomized controlled clinical trials for the treatment of acute uncomplicated *Plasmodium falciparum* malaria.
- Transient, asymptomatic, mostly mild-to-moderate increases in liver aminotransferases have been observed with pyronaridine–artesunate in some patients.
- We assessed pyronaridine–artesunate hepatic safety, tolerability, and effectiveness in adults and children for the treatment of acute uncomplicated malaria under real-world conditions in Africa, including patients with elevated baseline liver aminotransferases.

**What did the researchers do and find?:**

- In a single-arm, open-label, cohort event monitoring study conducted across 5 African countries, patients of any age, weighing >5 kg with acute uncomplicated malaria received fixed-dose pyronaridine–artesunate once daily for 3 days.
- Patient exclusion and inclusion criteria were consistent with the approved prescribing information, and patients with elevated baseline liver enzymes could be treated under the protocol.
- The study included 8,560 malaria episodes, 158 of which occurred in patients with baseline alanine aminotransferase (ALT) or aspartate aminotransferase (AST) abnormalities.
- There were no hepatic events following pyronaridine–artesunate treatment. Tolerability was as reported in previous studies, and treatment effectiveness at day 28 was 98.6% in patients with acute uncomplicated *P*. *falciparum* malaria.

**What do these findings mean?:**

- Pyronaridine–artesunate was an operationally effective and well-tolerated antimalarial therapy in this unselected population of patients from 5 African countries.

## Introduction

Africa carries the greatest malaria burden, with an estimated 215 million cases in 2019 and 94% of global deaths, mainly in children [1]. Children are also most vulnerable to ongoing morbidity, particularly from severe anemia [2].

Artemisinin-based combination therapy (ACT) is recommended for first-line therapy of acute uncomplicated malaria, with several artemisinin and partner drug combinations deployed worldwide. Artemisinin-resistant *Plasmodium falciparum* are established in Southeast Asia, and where these strains are also resistant to partner drugs, high clinical failure rates are reported [3]. De novo *Kelch 13* artemisinin resistance mutations have been verified in Africa [4], and parasites will eventually overcome the effectiveness of the most widely used therapies [5].

Pyronaridine–artesunate has demonstrated high efficacy in acute uncomplicated malaria in both Africa and Asia [6–18], including against artemisinin-resistant *P*. *falciparum* [7–10]. In controlled clinical trials, pyronaridine–artesunate safety compared favorably with other antimalarial drugs [6,11,14]. Asymptomatic, transient, mild-to-moderate elevations in alanine aminotransferase (ALT) or aspartate aminotransferase (AST) occurred in some patients treated with pyronaridine–artesunate [6,11,14]. A systematic meta-analysis showed that following first treatment, the risk of ALT greater than 5 times the upper limit of normal (>5×ULN) was higher in those treated with pyronaridine–artesunate (0.7%) compared to comparators (0.2%; relative risk [RR] 3.34 [95% confidence interval CI 1.63, 6.84]), but there was no difference in the proportion of patients with elevated AST >5×ULN (RR 1.80 [95% CI 0.89, 3.65]), or increased bilirubin >2.5×ULN (RR 1.03 [95% CI 0.49, 2.18]) [6]. Although 1 case of raised ALT with raised bilirubin was reported, there were no reports of severe drug-induced liver injury, and no clinical observations indicative of hepatic injury with pyronaridine–artesunate [6,11,14]. Subsequently, an extensive longitudinal study in West African malaria patients indicated no increased risk of biochemically defined hepatic dysfunction on repeated pyronaridine–artesunate treatment [11]. In that study, the incidence of ALT or AST >3×ULN and total bilirubin >2×ULN was 0.31% (3/967) with artemether–lumefantrine, 0.22% (3/1,342) with pyronaridine–artesunate, 0.15% (2/1,340) with dihydroartemisinin–piperaquine, and 0.09% (1/1,061) with artesunate–amodiaquine [11]. Similar to previous studies, the elevations in ALT/AST occurred in the first 7 days after treatment with pyronaridine–artesunate and generally had normalized by day 15. The liver enzyme observations were not associated with any clinical signs or symptoms of hepatic injury and were without sequelae or the need for any intervention [11].

Although the clinical relevance of the observed transient asymptomatic liver aminotransferase elevations is unclear, all pyronaridine–artesunate clinical trial populations to date have excluded patients with underlying hepatic dysfunction. Consequently, it is currently unknown if underlying hepatic dysfunction will progress to clinical manifestations of liver impairment with pyronaridine–artesunate. It is important, therefore, to clarify the risk–benefit of pyronaridine–artesunate in an unselected, representative population treated under normal clinical conditions in accordance with current drug prescribing recommendations.

This large clinical trial was conducted as part of the postmarketing requirements specified by the European Medicines Agency (EMA) for pyronaridine–artesunate. The pyronaridine–artesunate-approved product prescribing information does not require prior measurement of liver enzymes before treatment [19]. The primary aim of the study was to assess the incidence of clinical hepatic signs and symptoms in patients recruited in a normal clinical context, including patients with baseline elevations in ALT/AST. Any potential differences in the incidence of clinical hepatic events in malaria patients with elevated baseline ALT/AST values were to be compared to a matched cohort with normal values. The EMA considered the protocol appropriate to answer the question of whether clinically relevant hepatic events occurred following pyronaridine–artesunate in patients recruited as per the summary of product characteristics (i.e., a clinically relevant population) [19]. The study encompassed active pharmacovigilance alongside launch and scale-up of pyronaridine–artesunate deployment in Africa and was designed in accordance with standard cohort event monitoring protocols [20,21].

In summary, the aim of this study was to characterize pyronaridine–artesunate hepatic safety, tolerability, and effectiveness in a real-world setting in a clinically relevant population across a network of representative study sites in Central and West Africa.

## Methods

### Study design

This single-arm, open-label cohort event monitoring study was conducted at 6 health centers in Cameroon, Democratic Republic of Congo, Gabon, Ivory Coast, and Republic of Congo between 22 June 2017 and 10 April 2019. The protocol adhered to Good Clinical Practice, the Declaration of Helsinki, and relevant regulations in the respective countries and was reviewed and accepted as a valid postmarketing safety assessment by the EMA. The authors vouch for the accuracy and completeness of the data and the fidelity of the trial conduct, analysis, and reporting to the protocol. A copy of the protocol and statistical analysis plan is provided in the Supporting information (S1 Protocol and SAP). This study is reported as per the Strengthening the Reporting of Observational Studies in Epidemiology (STROBE) guideline (S1 STROBE Checklist).

### Ethics statement

All patients, or their parents/guardians, provided written informed consent, and children able to understand the study gave signed assent. The protocol was conducted in compliance with the Declaration of Helsinki (amended Tokyo 2004) and the directives in the respective countries, in particular concerning the submission to Ethics Committees and the protection of personal data. Additionally, the study was conducted in accordance with the principles of the Good Clinical Practice according to the International Council for Harmonization (ICH) Harmonized Tripartite Guidance (CPMP/ICH/135/95). The ethics committee at each study site granted ethical approval as follows. Cameroon: Comité National d’Ethique de la Recherche pour la Santé Humaine; Democratic Republic of Congo: Comité d’Ethique de l’Université Protestante au Congo; Gabon: Comité Nationale d’Ethique et de la Recherche Scientifique and Comité d’Ethique Institutionnel Albert Schweitzer Hospital, Lambaréné; Ivory Coast: Comité Nationale d’Ethique et de la Recherche Scientifique and Comité National d’Ethique des Sciences de la Vie et de Santé; and Republic of Congo: Institutional Ethics Committee of the Fondation Congolaise pour la Recherche Médicale.

### Patients

Eligible patients were male or female, of any age, with body weight of at least 5 kg. All patients had acute uncomplicated malaria and a fever or history of fever within the previous 24 hours and/or anemia. Exclusion criteria were severe malaria, clinical signs or symptoms of hepatic injury (nausea and abdominal pain associated with jaundice), known severe liver disease (i.e., decompensated cirrhosis, Child–Pugh stage C or D), known allergy to artemisinin and/or pyronaridine, known pregnancy, lactation, treatment with pyronaridine–artesunate in the previous 28 days, or if the patient was considered by the investigator to be at particular risk.

### Treatment

Fixed-dose pyronaridine–artesunate (Shin Poong Pharmaceutical, Seoul, South Korea) was administered once daily for 3 days with 2 formulations provided according to body weight and without regard to food intake. Daily doses for pyronaridine–artesunate tablets (180:60 mg) were 1 (≥20 to <24 kg), 2 (≥24 to <45 kg), 3 (≥45 to <65 kg), or 4 (≥65 kg); and for granule sachets (60:20 mg) were 1 (≥5 to <8 kg), 2 (≥8 to <15 kg), or 3 (≥15 to <20 kg). To reflect real-world practice, patients could be included more than once in the study, following a 28-day washout period between consecutive pyronaridine–artesunate treatments. Patients received the first pyronaridine–artesunate dose under supervision and were provided with the remaining doses to be taken unsupervised on the subsequent 2 consecutive days.

### Procedures

At enrollment on day 0, eligible patients underwent physical examination, with demographic data and medical history noted.

Blood samples (5 mL) were collected for hematology, clinical chemistry, and confirmation of malaria diagnosis. Blood samples were stored for retrospective analysis of baseline clinical chemistry and viral hepatitis assessment: hepatitis A, B, C, and E (anti-HAV IgM, anti-HBc IgM, HBsAg, hepatitis C RNA, and hepatitis E IgM antibody) and hepatitis delta antibody if positive for hepatitis B. All laboratory analysis was performed at the site where the patient was recruited by technically certified staff, and results were validated by external independent laboratories. Standard quality control procedures were in place, including daily internal controls and the quality control process assessed by the clinical monitors.

Malaria was diagnosed using a rapid diagnostic test (RDT) or Giemsa-stained thick blood smear. The RDT was consistent with local treatment guidelines and used according to the manufacturer’s instructions by staff trained according to the local standard operating procedure (S1 Methods). Microscopic diagnosis was performed in accordance with standard protocols by qualified microscopists [22], with 10% of slides undergoing quality control checks by an independent microscopist. Blood spots were obtained at screening and stored on filter paper for polymerase chain reaction (PCR) genotyping. Patients were visited by a community health worker (CHW) on days 7 and 28 who inquired regarding any signs or symptoms of malaria. In addition, patients were requested to present to the health center should they have malaria symptoms at any time during follow-up. Any patient who presented with suspected malaria was clinically examined and malaria confirmed by microscopy. At follow-up day 28, blood samples were collected from all patients to confirm parasite clearance by microscopy. In the case of *P*. *falciparum* recurrence, PCR genotyping was used to differentiate between recrudescence and reinfection by comparing blood spot samples taken at baseline versus recurrence, using published criteria [23].

Adverse events were assessed without knowledge of baseline ALT/AST. Patients were to present at the clinic should any adverse event occur within 28 days of pyronaridine–artesunate dosing. Home follow-up visits were conducted by CHWs on days 7 and 28 to assess treatment adherence, clinical condition, adverse events, and concomitant medications. CHWs were trained before the study and retrained during the study by clinical investigators in the assessment of safety and tolerability using questionnaire forms. In addition, investigators accompanied CHWs to field visits to assure quality and consistency in adverse event assessment. All clinical information provided by CHWs was assessed by clinical investigators to ensure accurate medical recording of all safety and tolerability signs. CHWs were provided with pictorial information specifically identifying the signs and symptoms of liver injury. Furthermore, any potential hepatotoxic signs or symptoms noted by CHWs were specifically discussed with the study doctor, with patients referred to the clinic if necessary.

Hepatic events prompted blood sampling for clinical chemistry and hematology and a full hepatitis panel. Assessment at the health facility was triggered by serious or severe adverse events or adverse events of special interest, i.e., hepatotoxicity (jaundice, dark urine, putty/mastic stool, worsening of fatigue, nausea, vomiting, anorexia, abdominal pain, itching, rash, spontaneous bruising, or appearance of red spots), serious liver reactions, and hypersensitivity (flushing, wheals/urticaria, breathlessness, faintness, or a fall in blood pressure). Female participants had a urinary pregnancy test at day 28, with provision in the case of pregnancy for extended follow-up until the birth or other outcome.

### Outcomes and trial populations

The safety population included all enrolled patients who received at least 1 dose of study medication for any malaria episode. The primary outcome was the incidence of hepatic events in the safety population following pyronaridine–artesunate treatment of malaria patients with baseline ALT or AST >2×ULN, defined as clinical signs and symptoms of possible hepatotoxicity, i.e., fatigue, nausea, abdominal pain, itching, or signs of jaundice (dark urine, putty or mastic colored stools, and yellowing of the whites of the eyes or skin), associated with a rise in ALT/AST >2× the baseline value.

Safety outcomes were the incidence of adverse events in the safety population overall and categorized by normal/abnormal/unknown baseline ALT/AST, any underlying hepatic disease identified on the hepatitis panel, HIV status (where known), age, sex, weight, and nutritional status. Malnutrition was defined for patients <5 years old as a mid-upper arm circumference <115 mm, in patients aged ≥5 years to ≤19 years using the World Health Organization age- and sex-specific body mass index (BMI)-for-age z-scores [24], and for adults >19 years old as a BMI <18.5 kg/m^2^.

Treatment adherence was self-assessed by patients and also estimated by CHWs based on empty packaging provided by patients versus the number of tablets/sachets dispensed.

Effectiveness was evaluated on day 28 in the intention-to-treat and per-protocol populations, with cure defined as the absence of microbiologically confirmed malaria without previous failure. Cure rates were reported unadjusted and PCR adjusted for *P*. *falciparum* reinfection. Day 28 effectiveness subgroup analysis was planned for by country, dosing (granules versus tablets), malnutrition, and age. Additional outcomes were time between malaria episodes and the frequency of repeat episodes. The intention-to-treat population included all malaria episodes treated with at least 1 dose of study medication with confirmed positive parasitemia at baseline. The per-protocol population included all malaria episodes in the intention-to-treat population in patients who were treated with a full course of study medication (adherence per patient self-assessment), had a day 28 effectiveness endpoint, did not vomit after study drug administration (except where vomiting occurred after the first dose and dosing was successfully repeated), and who had no prior or concomitant medication, which had an antimalarial effect and so could interfere with the treatment outcome. All outcomes and trial populations were prespecified (S1 Protocol and SAP).

### Statistical analysis

Sample size was calculated based on the primary objective, i.e., the incidence of protocol-defined hepatic events in malaria patients with baseline ALT/AST >2×ULN. The screening rate in previous studies for malaria patients with baseline ALT/AST >2×ULN was 1.4% [25]. To detect 1 protocol-defined hepatic event in patients with baseline ALT/AST >2×ULN with a probability of at least 80% (81.6%), 120 malaria episodes occurring in patients with a baseline ALT/AST value >2×ULN were required. Thus, to observe 1 severe hepatic event, at least 8,572 malaria episodes were required (i.e., 1.4% of 8,572 = 120). There was no country-specific recruitment target, and the trial was to continue until observation of at least 120 malaria episodes in patients with elevated baseline ALT/AST. As a secondary endpoint, a multivariate logistic regression model was planned in order to compare the incidence of hepatic events in patients with elevated baseline ALT/AST values versus those with normal values. However, as the primary endpoint did not occur in any patient during the trial, this analysis was not conducted.

Descriptive statistics were used for analysis of all other outcomes, with 95% CIs calculated (Clopper–Pearson). To better mimic the clinical reality, patients could be included more than once in the study. For the outcome of clinical effectiveness, each malaria episode was treated as an independent event, with no adjustment for repeated measures. This was to better reflect the endpoint of operational effectiveness, i.e., to include patient-related as well as drug-related contributors and to be consistent with previous analyses of randomized controlled trials of repeated treatment with pyronaridine–artesunate [11,25]. For the determination of cure rates, patients with a missing parasitological assessment at day 28 were considered as failures in the intention-to-treat population and excluded from the per-protocol population. For further details, please see S1 Protocol and SAP. Statistical analysis used SAS (version 8.2) and GraphPad Prism (version 8.4.3). Adverse events were coded using MedDRA (version 22).

## Results

### Patients

**Fig 1:**
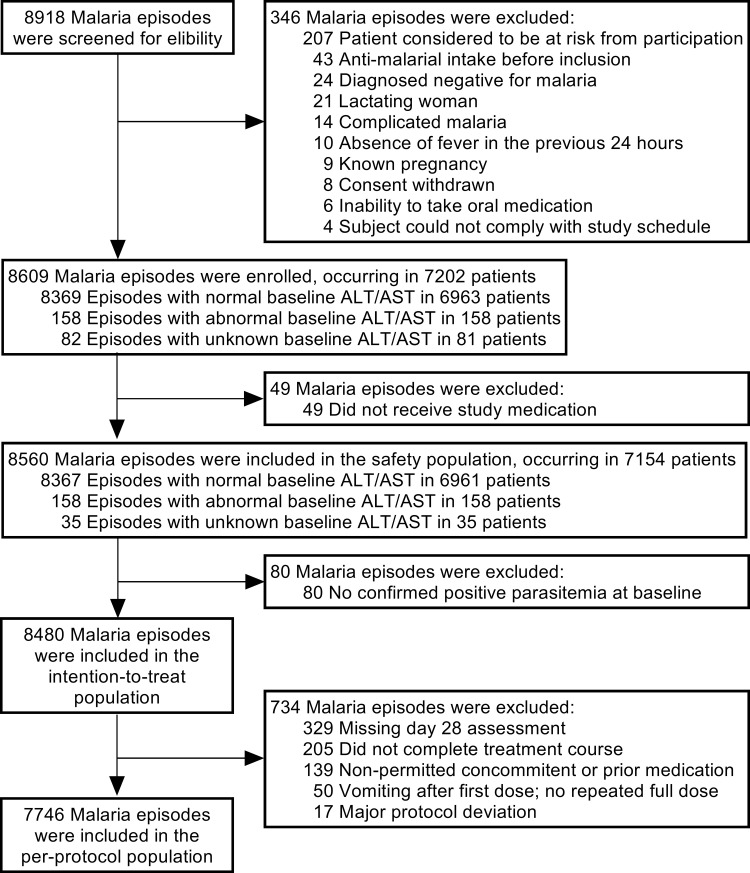
Screening and analysis populations. There may have been more than 1 reason for exclusion from enrollment into the study or exclusion from a study population. Normal LFTs were ALT or AST ≤2×ULN and abnormal values were ALT or AST >2×ULN at baseline. “Patient considered to be at risk from participation” included patients that investigators considered inappropriate for trial inclusion, mainly because of low hemoglobin or an inability to draw blood for assessments. Note that no patient with clinically evident preexisting liver injury was excluded from the screening population. ALT, alanine aminotransferase; AST, aspartate aminotransferase; LFT, liver function test; ULN, upper limit of normal.

Of the 8,609 malaria episodes that met study eligibility criteria, 8,560 were treated with at least 1 dose of study medication, comprising the safety population (Fig 1). Of the 1.9% (158/8,560) of malaria episodes occurring in patients with baseline ALT/AST elevations >2×ULN, 19.0% (30/158) were positive for viral hepatitis. The safety population included similar proportions of males and females; most patients had 1 malaria episode, but the maximum was 9 episodes (Table 1). Repeated malaria episodes accounted for 16.7% (1,414/8,480) of all episodes in the intention-to-treat population (Table 1). Time between repeated malaria episodes tended to decrease for each subsequent episode (S1 Fig). Based on patient self-assessment, treatment adherence was achieved in 94.5% (8,085/8,560) of malaria episodes versus 81.5% (6,974/8,560) based on empty medication packaging presented to CHWs (Table 1).

**Table 1:** Baseline demographic and clinical characteristics in the safety population.

**Characteristic by patient**	**Normal baseline ALT/AST** **(*N =* 6,961)**	**Abnormal baseline ALT/AST** **(*N* = 158)**	**Unknown baseline ALT/AST** **(*N* = 35)**	**Total (*N* = 7,154)**
Male sex—no. (%)	3,461 (49.7)	89 (56.3)	19 (54.3)	3,569 (49.9)
Mean age (SD)—y	14.0 ± 14.6	9.3 ± 13.6	10.8 ± 13.0	13.9 ± 14.6
<1 y—no. (%)	124 (1.8)	9 (5.7)	1 (2.9)	134 (1.9)
≥1 y to <5 y—no. (%)	1,621 (23.3)	77 (48.7)	13 (37.1)	1,711 (23.9)
≥5 to <18 y—no. (%)	3,543 (50.9)	51 (32.3)	15 (42.9)	3,609 (50.4)
≥18 y—no. (%)	1,673 (24.0)	21 (13.3)	6 (17.1)	1,700 (23.8)
Mean body weight (SD)—kg	33.1 ± 20.9	23.5 ± 17.8	26.0 ± 19.4	32.8 ± 20.9
≥5 to <20 kg—no. (%)	2,517 (36.2)	97 (61.4)	20 (57.1)	2,634 (36.8)
≥20 kg—no. (%)	4,444 (63.8)	61 (38.6)	15 (42.9)	4,520 (63.2)
Malnourished—no. (%)	365 (5.2)	3 (1.9)	2 (5.7)	370 (5.2)
Nonmalnourished—no. (%)	6,596 (94.8)	155 (98.1)	33 (94.3)	6,784 (94.8)
Country—no. (%)				
Ivory Coast	1,200 (17.2)	14 (8.9)	1 (2.9)	1,215 (17.0)
Cameroon	776 (11.1)	20 (12.7)	1 (2.9)	797 (11.1)
Democratic Republic of Congo	2,785 (40.0)	64 (40.5)	10 (28.6)	2,859 (40.0)
Republic of Congo	607 (8.7)	23 (14.6)	5 (14.3)	635 (8.9)
Gabon	1,593 (22.9)	37 (23.4)	18 (51.4)	1,648 (23.0)
Number of malaria episodes per patient—no. (%)				
1	5,885 (84.5)	130 (82.3)	22 (62.9)	6,037 (84.4)
2	811 (11.7)	17 (10.8)	12 (34.3)	840 (11.7)
3	162 (2.3)	6 (3.8)	1 (2.9)	169 (2.4)
4	71 (1.0)	4 (2.5)	0	75 (1.0)
5	20 (0.3)	1 (0.6)	0	21 (0.3)
6	7 (0.1)	0	0	7 (0.1)
7	3 (<0.1)	0	0	3 (<0.1)
8	1 (<0.1)	0	0	1 (<0.1)
9	1 (<0.1)	0	0	1 (<0.1)
**Characteristic by malaria episode**	**Normal baseline ALT/AST** **(*N* = 8,367)**	**Abnormal baseline ALT/AST** **(*N* = 158)**	**Unknown baseline ALT/AST** **(*N =* 35)**	**Total (*N =* 8,560)**
Mean baseline ALT (SD) [range]—IU/L	12.8 (8.2) [0.1, 95.0]	100.7 (146.4) [1.8, 1,103.4]	–	14.4 (24.5)^a^ [0.1, 1,103.4]
Mean baseline AST (SD) [range]—IU/L	28.7 (12.2) [1.0, 163.0]	182.6 (271.4)^b^ [20.0, 1,952.3]	–	31.5 (43.9)^c^ [1.0, 1,952.3]
Episodes in which first dose was vomited—no. (%)	199 (2.4)	5 (3.2)	10 (28.6)	214 (2.5)
Episodes that received a repeated first dose—no. (%)	189 (2.3)	5 (3.2)	10 (28.6)	204 (2.4)
Patient-reported adherence—no. (%)	7,925 (94.7)	145 (91.8)	15 (42.9)	8,085 (94.5)
Adherence assessed by CHWs—no. (%)^d^	6,837 (81.7)	126 (79.7)	11 (31.4)	6,974 (81.5)

ALT, alanine aminotransferase; AST, aspartate aminotransferase; CHW, community health worker; LFT, liver function test; SD, standard deviation; ULN, upper limit of normal.

Normal LFTs were ALT or AST ≤2×ULN, and abnormal values were AST or ALT >2×ULN at baseline. –, no observations.

^a^*N =* 8,525.

^b^*N* = 157.

^c^*N* = 8,524.

^d^Adherence was assessed by CHWs based on the number of empty blisters/sachets presented.

### Hepatic safety

There were no malaria episodes with protocol-defined hepatic events in any patient in the safety population. No further planned secondary analyses of the primary endpoint could, therefore, be conducted.

### Safety

**Fig 2:**
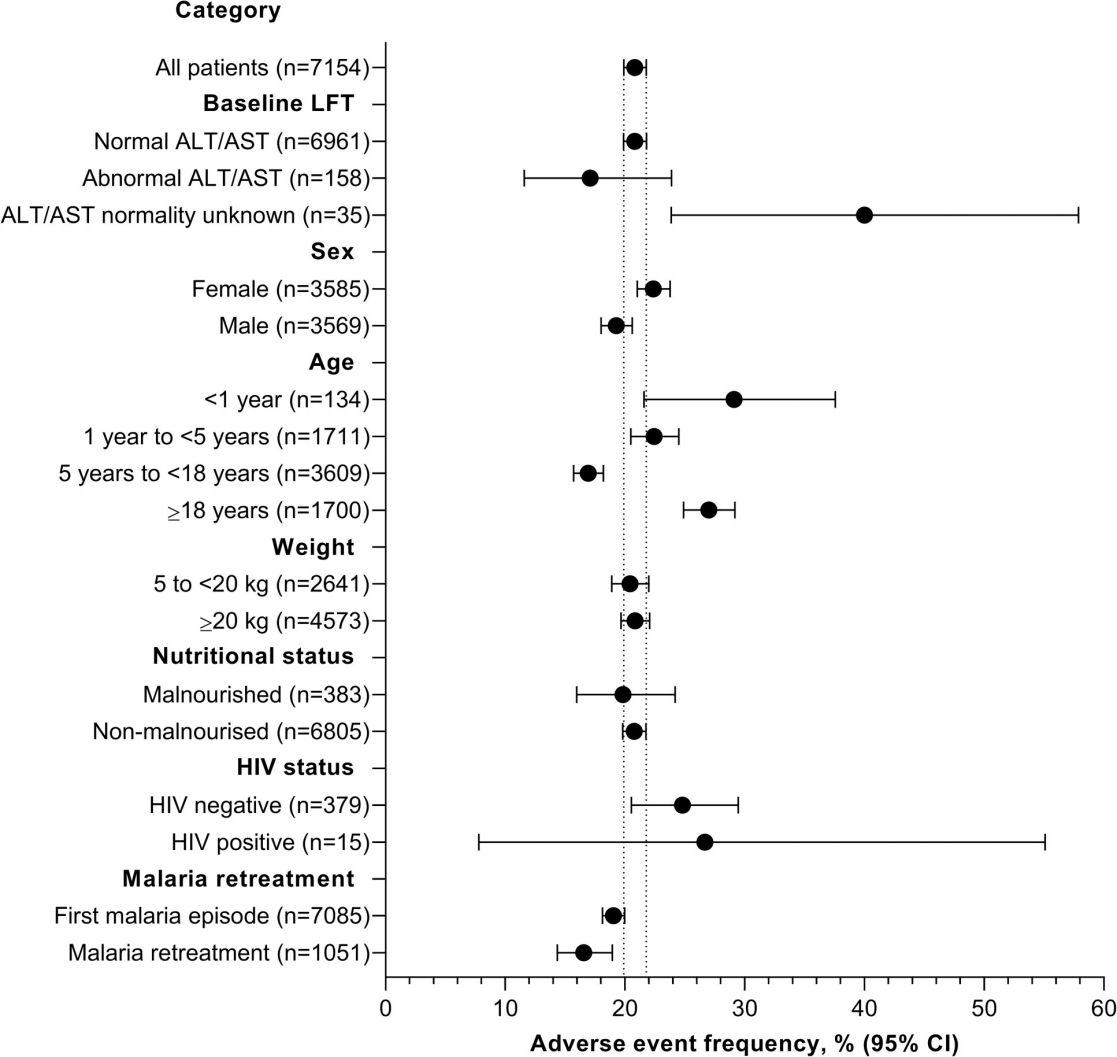
Frequency of adverse events of any cause subgroup analysis in the safety population. Data are shown for the number of patients. Patients may have been categorized differently for different malaria episodes. Categorical data were not available for all patients. ALT, alanine aminotransferase; AST, aspartate aminotransferase; CI, confidence interval; LFT, liver function test.

Adverse events of any cause occurred in 20.8% (1,490/7,154) of patients, most frequently pyrexia (5.1% [366/7,154]) and vomiting (4.2% [303/7,154]) (Table 2, S1 Table). There was no difference in adverse event frequency between patients with normal versus abnormal baseline ALT/AST values (Fig 2), although those with unknown ALT/AST values experienced more adverse events (40.0% [14/35] 95% CI, 25.6 to 56.4).

Adverse event frequency was not affected by sex, weight, nutritional status, HIV status, or first versus repeated malaria episode (Fig 2). The nature of adverse events and their incidence was comparable for first versus repeated episodes (S2 Table). Four of the 30 patients positive for viral hepatitis had adverse events (13.3%).

Higher adverse event rates were noted for patients aged under 1 year (29.1% [39/134] 95% CI, 21.6 to 37.6) and those ≥18 years of age (27.0% [459/1,700] 95% CI, 24.9 to 29.2) versus those aged 5 to <18 years (16.9% [611/3,609] 95% CI, 15.7 to 18.2) (Fig 2, S3 and S4 Tables). In particular, vomiting was associated with age, occurring in 11.2% [15/134] of children under 1 year old, 7.7% (131/1,711) of those aged 1 to <5 years, 3.1% (112/3,609) of children and adolescents 5 years to 18 years, and in just 2.8% (47/1,700) of adults (S3 Table). The frequency of pyrexia and cough also tended to decrease with increasing age (S3 Table).

**Table 2:** Most common adverse events of any cause in patients treated with pyronaridine–artesunate for acute uncomplicated malaria (safety population).

Adverse event, *n* (%)	Normal baseline ALT/AST (*N =* 6,961)	Abnormal baseline ALT/AST (*N* = 158)	Unknown baseline ALT/AST (*N* = 35)	Total (*N* = 7,154)
Patients with any adverse event	1,449 (20.8)	27 (17.1)	14 (40.0)	1,490 (20.8)
Pyrexia	361 (5.2)	4 (2.5)	1 (2.9)	366 (5.1)
Vomiting	285 (4.1)	8 (5.1)	10 (28.6)	303 (4.2)
Headache	201 (2.9)	0	1 (2.9)	202 (2.8)
Cough	100 (1.4)	3 (1.9)	3 (1.9)	103 (1.4)
Diarrhea	79 (1.1)	2 (1.3)	0	81 (1.1)
Abdominal pain	74 (1.1)	0	0	74 (1.0)
Dizziness	65 (0.9)	0	0	65 (0.9)
Fatigue	65 (0.9)	0	0	65 (0.9)
Asthenia	48 (0.7)	1 (0.6)	0	49 (0.7)
Influenza	43 (0.6)	3 (1.9)	0	46 (0.6)
Decreased appetite	42 (0.6)	0	0	42 (0.6)
Influenza-like illness	40 (0.6)	1 (0.6)	0	41 (0.6)
Pruritus	40 (0.6)	0	0	40 (0.6)
Anemia	26 (0.4)	1 (0.6)	2 (5.7)	29 (0.4)
Nausea	31 (0.4)	0	0	31 (0.4)
Rhinorrhea	23 (0.3)	0	0	23 (0.3)
Malaria	16 (0.2)	1 (0.6)	1 (2.9)	18 (0.3)
Rash	22 (0.3)	0	0	22 (0.3)
Vertigo	17 (0.2)	0	0	17 (0.2)
Arthralgia	16 (0.2)	0	0	16 (0.2)
Abdominal pain upper	14 (0.2)	0	0	14 (0.2)
Acarodermatitis	13 (0.2)	0	1 (2.9)	14 (0.2)
Nasopharyngitis	12 (0.2)	0	0	12 (0.2)
Myalgia	12 (0.2)	0	0	12 (0.2)
Hyperhidrosis	12 (0.2)	0	0	12 (0.2)
Bronchitis	11 (0.2)	0	0	11 (0.2)

Adverse events occurring in >0.1% of patients; a complete list of adverse events is shown in S1 Table. Patients may have had more than 1 adverse event. Normal LFTs were ALT or AST ≤2×ULN, and abnormal values were AST or ALT >2×ULN at baseline.

ALT, alanine aminotransferase; AST, aspartate aminotransferase; LFT, liver function test; ULN, upper limit of normal.

There were 9.6% (685/7,154) of patients with adverse events considered related to pyronaridine–artesunate treatment, most commonly vomiting (3.2% [228/7,154]) and headache (1.0% [72/7,154]) (S5 Table). Treatment was discontinued early owing to adverse events in 0.8% (54/7,154) of patients, mostly because of vomiting (0.5% [37/7,154]) (S6 Table). Severe adverse events occurred in 0.4% (32/7,154) of patients and life-threatening events in 0.1% (10/7,154) (S7 Table).

Serious adverse events were reported in 0.4% (29/7,154) of patients, with the most frequent being malaria (0.1% [9/7,154]) and anemia (0.1% [7/7,154]) (S8 Table). Four patients had serious adverse events considered possibly related to pyronaridine–artesunate treatment (epistaxis, gastroenteritis, and 2 cases of anemia).

There were 3 deaths during the study (diarrhea, drowning, and severe malaria), none of which were considered related to pyronaridine–artesunate treatment. The case of severe malaria occurred in a 6-year-old girl from the Democratic Republic of Congo who was enrolled with acute uncomplicated malaria, presenting with asthenia and vomiting. She was diagnosed with severe malaria 1.5 hours following the first dose of pyronaridine–artesunate and admitted to hospital after symptoms of asthenia worsened. Pyronaridine–artesunate was discontinued, and the patient treated with intravenous quinine. She also received intravenous phenobarbital and diazepam for convulsions and cefotaxime and gentamycin for suspected meningitis, but her condition rapidly worsened, and she unfortunately died later the same day. As the patient died of severe malaria within 24 hours of her first dose of pyronaridine–artesunate, it is usual to consider the cause as disease progression, and so the death was not attributed to drug treatment. The death from diarrhea occurred in a 66-year-old man who received 6 pyronaridine–artesunate tablets over 3 days, who died 29 days after the first dose of study medication from severe diarrhea, with no further information available. The other death was that of a 9-year-old boy who drowned in a river 12 days after the first administration of pyronaridine–artesunate.

An adverse event of special interest occurred in 9 patients (0.1%, *N =* 7,154); in all cases, this was hypersensitivity occurring only in 1 episode: 6 occurred in the first episode (6/7,085; 0.09% 95% CI, 0.03 to 0.18), and the other 3 occurred for the first time in episodes 2, 3, and 4, respectively (3/1,051; 0.3%; 95% CI, 0.06 to 0.8). All patients were aged ≥5 years with normal baseline ALT/AST, 4 had urticaria, and 1 each had maculo-papular rash, papular rash, hypersensitivity (symptomatology of flushing and wheals), lip swelling, and eye pruritis; all were of mild-to-moderate intensity.

CHWs identified 595 cases that required discussion of adverse events with the investigational site, with 165 referrals. An additional 223 cases self-referred. Of these, further blood testing was clinically indicated in 7 patients, with no concerning results (S9 Table). Eleven pregnancies were confirmed during the study. Three were terminated for social reasons. The remaining 8 (6 of which were exposed to study treatment during the first trimester) progressed normally with spontaneous deliveries, except for 1 case of uterine hypertonus at full term, requiring cesarean section.

### Effectiveness

*P*. *falciparum* was the predominant pathogen, present in 8,478 of 8,480 malaria episodes (intention-to-treat population). *Plasmodium ovale* (*n =* 70) and *Plasmodium malariae* (*n* = 55) occurred as mixed *Plasmodium* infections; 1 *P*. *ovale*/*P*. *malariae* coinfection and 1 *Plasmodium vivax* mono-infection were identified (S10 Table). Adjusting for *P*. *falciparum* reinfection, treatment effectiveness at day 28 was 98.6% ([7,369/7,746] 95% CI 98.3 to 98.9) in the per-protocol population and 90.9% ([7,705/8,478] 95% CI 90.2 to 91.5) in the intention-to-treat population. The unadjusted cure rate in the per-protocol population was 93.2% ([7,221/7,746] 95% CI 92.6 to 93.8) and 85.9% ([7,285/8,480] 95% CI 85.1 to 86.6) in the intention-to-treat population (S11 Table).

**Fig 3:**
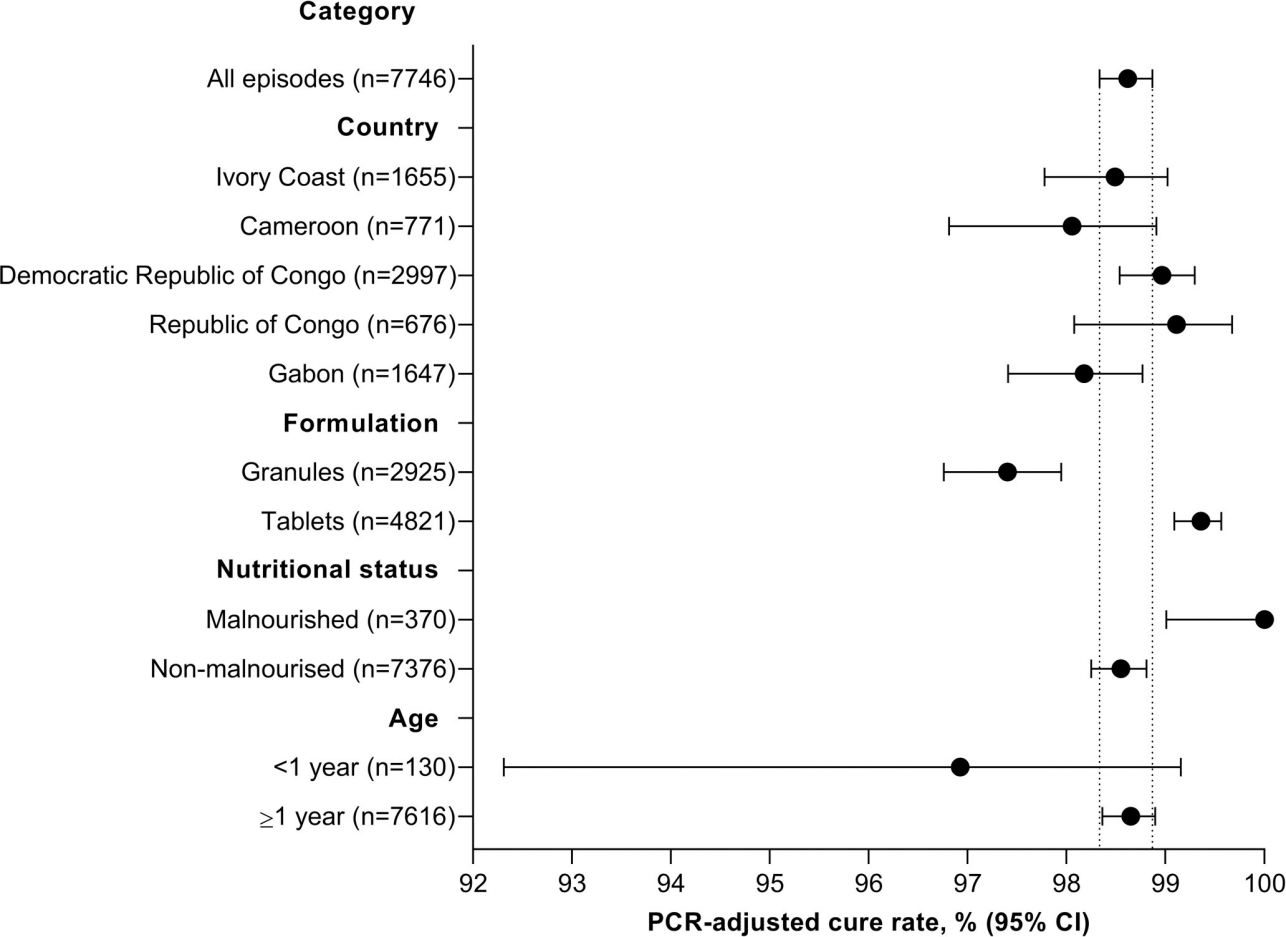
Day 28 PCR-adjusted treatment effectiveness in the per-protocol population subgroup analysis. Data are shown for the number of malaria episodes. CI, confidence interval; PCR, polymerase chain reaction.

Subgroup analyses of the day 28 PCR-adjusted cure rate in the per-protocol population are shown in Fig 3 and S11 Table. Cure rates across the 5 participating countries exceeded 98%. The cure rate was lower for the granule formulation (97.4% [2,849/2,925] 95% CI 96.8 to 97.9) versus tablets (99.4% [4,790/4,821] 95% CI 99.1 to 99.6) and lower in infants (<1 year old; 96.9% [126/130] 95% CI, 92.3 to 99.2) versus older children and adults (98.6% [7,513/7,616] 95% CI, 98.4 to 98.9), though CIs overlapped. In malnourished patients, the cure rate was 100% (370/370).

## Discussion

This study was conducted within the Central Africa Network on Tuberculosis, HIV/AIDS, and Malaria (CANTAM). There were no hepatic events in 8,560 episodes of uncomplicated *P*. *falciparum* malaria treated with pyronaridine–artesunate, 158 of which occurred in patients with baseline ALT or AST abnormalities. Tolerability was consistent with previous studies, and treatment effectiveness at day 28 was 98.6%.

Pyronaridine–artesunate efficacy and safety have been already demonstrated in multicenter, randomized, controlled clinical trials conducted for regulatory purposes in a selected patient population. In those studies, raised ALT >5×ULN was more frequently observed with pyronaridine–artesunate versus comparators, but clinical signs and symptoms of hepatotoxicity were not manifest and all patients recovered without sequelae or intervention [6,11,14–18,25]. However, the exclusion from these Phase II/III trials of individuals with elevated baseline liver enzymes left uncertainty regarding the clinical relevance of the biochemical findings in patients with underlying hepatic dysfunction. The aim of the current study was to generate data to inform clinical practice and provide evidence for decision-making by Ministries of Health and malaria control programs. Additionally, the study was conducted following EMA requirements to collect safety data to support scale-up of pyronaridine–artesunate access in countries where routine pharmacovigilance is limited. Thus, the design reflected the reality of malaria treatment in Central and West Africa to investigate pyronaridine–artesunate as an operational antimalarial. The study protocol specified a patient population based on the approved prescribing information for pyronaridine–artesunate and so can be considered representative of the wider patient population that might be treated with the drug. Patients underwent prescreening procedures consistent with those conducted under normal clinical practice. Although samples for clinical biochemistry were taken at baseline, these were only available retrospectively and collected to verify that patients with elevated ALT and AST had actually been recruited in sufficient numbers to provide the necessary power for the primary analysis of protocol-defined hepatic event incidence. Of the 8,560 malaria episodes followed, 1.9% (158) occurred in patients with baseline ALT/AST elevations >2×ULN. In fact, no hepatic events occurred in any patient, and the study exceeded the recruitment target, demonstrating with 89.2% power that none of 158 patients with elevated baseline ALT/AST experienced a protocol-defined hepatic event.

There are some key limitations to this study as designed. Firstly, this study aimed to assess whether there was any clinical evidence of hepatotoxicity following pyronaridine–artesunate administration according to the approved prescribing information. There is no requirement to measure ALT/AST in patients without signs or symptoms of liver dysfunction prior to treatment in normal clinical practice. Thus, the current study was explicitly designed to identify clinical hepatic events only. Although this reflects normal practice and resource availability in the study setting, not all patients with hepatitis will manifest clinical symptoms, and our study does not exclude the possibility of occurrences of subclinical liver injury. The use of the retrospective analysis of baseline blood samples was employed to confirm that patients with AST/ALT >2×ULN had, in fact, been recruited and to allow comparison with a matched cohort of patients with normal baseline ALT/AST (secondary endpoint). Postbaseline clinical biochemistry was only conducted where clinically indicated, for example, for biochemical confirmation of any clinically suspected signs of hepatotoxicity. In fact, postbaseline biochemistry for any reason was indicated in only 7 patients. Consequently, no conclusions can be drawn on the impact of pyronaridine–artesunate on postbaseline ALT/AST levels for patients with elevated baseline values. Secondly, in line with the prescribing information for pyronaridine–artesunate, patients with clinical signs or symptoms of hepatic injury (such as nausea and/or abdominal pain associated with jaundice) or known severe liver disease (i.e., decompensated cirrhosis, Child–Pugh stage B or C) were excluded [19]. Thus, no conclusions can be reached regarding the safety of pyronaridine–artesunate in patients with preexisting severe liver injury, and ethical considerations preclude the evaluation of patients in clinical trials who have contraindications to treatment. In fact, although the prespecified exclusion criteria are easily recognized in normal clinical practice, no patients were excluded from the study for hepatic injury or severe liver disease. This is probably because malaria tends to occur in younger patients, and the preexisting liver disease must be severe enough to be clinically evident. One further limitation is that because there were no instances of the primary endpoint, i.e., protocol-defined hepatic events, a number of the planned secondary analyses could not be conducted, most notably the cohort comparison with patients who had normal baseline ALT/AST [20] and a sub-analysis of the potential influence of concomitant medications on the incidence of hepatic events (S1 Protocol and SAP).

Randomized controlled clinical trials of pyronaridine–artesunate provide an extensive safety database compared to a number of different antimalarial drugs, and the safety profile is well documented [6,11,14–18,25]. However, previous study populations were selected using more restrictive inclusion and exclusion criteria and had extensive follow-up and clinical monitoring. Safety and tolerability assessed under real-world clinical conditions in a clinically relevant population provide a valuable assessment of risk–benefit for prescribers and patients, particularly where pharmacovigilance is limited [20,21]. In the current study, only 0.8% (54/7,154) of patients discontinued treatment owing to an adverse event. Adverse event frequency (20.8% [1,490/7,154]) was lower than observed in previous randomized controlled clinical trials (56.5% [2,348/4,157]), as was the frequency of serious adverse events (0.4% [29/7,154] versus 1.0% [41/4,157]) [11,14]. This is explained by less frequent follow-up (days 7 and 28) in the current study than in the previous trials (days 1 to 3, 7, 14, 21, and 28). Also, postbaseline hematological and biochemical data were not routinely collected, so out-of-normal range values without clinical symptoms were not captured. Adverse event frequency was lower in patients aged ≥5 to 18 years than in younger children, mainly because of more frequent vomiting and pyrexia in children <1 year old. A similar pattern was observed in a previous study conducted in West Africa [11]. Another similarity between these 2 studies is that the frequency of adverse events tended to decrease on malaria retreatment [11]. Overall, the most common adverse events observed were consistent with previous studies and with the symptoms of malaria, and there were no unexpected adverse events based on the known safety profile of pyronaridine–artesunate [6,11,14–18,25].

The pharmacodynamic outcome of this trial was clinical effectiveness under real-world conditions, whereas previous randomized clinical trials reported clinical efficacy as defined using the standard WHO outcome of adequate clinical and parasitological response (ACPR) [22]. Across all 5 countries included in the study, pyronaridine–artesunate treatment effectiveness was ≥98%. This assessment was based on the per-protocol population, which included patients with adherence assessed from patient reporting, rather than the lower adherence rates reported by CHWs. Thus, the clinical effectiveness of pyronaridine–artesunate might be expected to be lower than its efficacy following fully supervised therapy in randomized clinical trials. However, this was not the case. Overall effectiveness with the tablet formulation in the per-protocol population, adjusted for reinfection by PCR, was 99.3% (4,790/4,821] 95% CI 99.1 to 99.6) and was comparable with >99% ACPR reported in randomized controlled clinical trials using this formulation [11,14,15,18]. Effectiveness of the pyronaridine–artesunate pediatric granule formulation was lower (97.4% [2,849/2,925] 95% CI 96.8 to 97.9) than for the tablet formulation, but comparable with ACPR reported from a previous randomized controlled trial in African children using the granule formulation (97.1% [329/339] 95% CI 94.6 to 98.6) [16]. In both instances, the lower effectiveness/efficacy of the granule versus the tablet was associated with a high rate of vomiting observed in younger children [16]. In the present study, vomiting occurred in 7.9% (146/1,845) of children under 5 years old versus only 3.1% (112/3,609) in those aged 5 to 18 years. Similarly, effectiveness tended to be lower in children under 1 year old, with 11.2% (15/134) of these infants experiencing vomiting. It is not possible within this trial to confirm the causality of the lower effectiveness observed for young children who received the granule formulation. Based on previous data, drug exposure is comparable between the tablet and granule formulations when all doses are fully supervised [26]. However, the association with vomiting and difficulties in administering complete doses of oral medication, even with a specific pediatric granule formulation, are likely to result in lower drug exposures in young children. Also, as young children lack the semi-immunity to malaria that adolescents and adults acquire in this endemic region, they may also be more sensitive to underdosing caused by vomiting. Consequently, the finding that antimalarial effectiveness in children is lower than in adults is not unexpected, though for pyronaridine–artesunate to achieve high levels of treatment effectiveness under operational conditions, comparable to the efficacy observed in randomized clinical trials, is encouraging [16].

In the per-protocol population, the difference between PCR-adjusted effectiveness (98.6% [95% CI 98.3, 98.9]) and unadjusted effectiveness (93.2% [95% CI 92.6, 93.8]) suggests that around 5% of patients were reinfected within 28 days of pyronaridine–artesunate treatment. Pyronaridine has a half-life of about 14 to 18 days [19], and posttreatment protection with pyronaridine–artesunate is limited [11]. Also, this population is likely to include patients with suboptimal adherence. In this case, even if drug exposure was sufficient to clear the initial infection, the period of posttreatment prophylaxis may have been reduced. Given that malaria transmission is still substantial in the study areas in general and the observation that individual patients had multiple malaria episodes during this study, this reinfection rate is not unexpected and consistent with previous findings [11].

In summary, in this large clinical trial, conducted in a real-world setting in a representative population of African malaria patients, pyronaridine–artesunate had excellent tolerability, safety, and effectiveness, and no clinically evident hepatic events occurred in patients with or without ALT/AST elevations at baseline. The study supports pyronaridine–artesunate as an operationally valuable therapy for the treatment of acute uncomplicated *P*. *falciparum* malaria.

## Supporting information

S1 STROBE Checklist STROBE Statement—Checklist of items that should be included in reports of cohort studies. (PDF)

S1 Protocol and SAP This supporting information contains the original protocol (version 7.0), final protocol (version 9.0), summary of changes from version 7.0 to version 8.0 (summary 2) and from version 8.0 to version 9.0 (summary 3), and the original statistical analysis plan and amendments to the plan. (PDF)

S1 Methods Rapid diagnostic tests. (PDF)

S1 Table Adverse events of any cause. (PDF)

S2 Table Adverse events of any cause following repeated pyronaridine–artesunate treatment. (PDF)

S3 Table Adverse events of any cause by age. (PDF)

S4 Table Adverse events of any cause by category. (PDF)

S5 Table Adverse events considered related to pyronaridine–artesunate. (PDF)

S6 Table Adverse events leading to early discontinuation of pyronaridine–artesunate. (PDF)

S7 Table Severe and life-threatening adverse events of any cause. (PDF)

S8 Table Serious adverse events of any cause. (PDF)

S9 Table Laboratory values at baseline and changes at day 7 and day 28. (PDF)

S10 Table Pyronaridine–artesunate day 28 unadjusted cure rate in African patients with acute uncomplicated malaria by *Plasmodium* species at baseline. (PDF)

S11 Table Pyronaridine–artesunate day 28 cure rate in African patients with acute uncomplicated malaria by category. (PDF)

S1 Fig Mean number of days between malaria episodes treated with pyronaridine–artesunate (intention-to-treat population). (PDF)

## Abbreviations

ACPR: adequate clinical and parasitological response
ACT: artemisinin-based combination therapy
ALT: alanine aminotransferase
AST: aspartate aminotransferase
BMI: body mass index
CANTAM: Central Africa Network on Tuberculosis, HIV/AIDS, and Malaria
CHW: community health worker
CI: confidence interval
EMA: European Medicines Agency
ICH: International Council for Harmonization
PCR: polymerase chain reaction
RDT: rapid diagnostic test
RR: relative risk
SD: standard deviation
STROBE: Strengthening the Reporting of Observational Studies in Epidemiology
ULN: upper limit of normal
