# Supplementary material for: Pyronaridine–artesunate real-world safety, tolerability, and effectiveness in malaria patients in 5 African countries: A single-arm, open-label, cohort event monitoring study
Source: PLoS Med. 2021 Jun 15;18(6):e1003669. doi: 10.1371/journal.pmed.1003669 (PMC8205155; doi:10.1371/journal.pmed.1003669)
Supplement: S1 STROBE Checklist — (PDF) [file pmed.1003669.s001.pdf]

S1 Checklist.

STROBE Statement—Checklist of items that should be included in reports of ***cohort studies***

	Item No	Recommendation	Page number
**Title and abstract**	1	(a) Indicate the study's design with a commonly used term in the title or the abstract	Abstract methods and findings
		(*b*) Provide in the abstract an informative and balanced summary of what was done and what was found	Abstract methods and findings
**Introduction**			
Background/rationale	2	Explain the scientific background and rationale for the investigation being reported	Introduction para 1-5
Objectives	3	State specific objectives, including any pre-specified hypotheses	Introduction para 5-6
**Methods**			
Study design	4	Present key elements of study design early in the paper	Methods study design para 1
Setting	5	Describe the setting, locations, and relevant dates, including periods of recruitment, exposure, follow-up, and data collection	Methods study design para 1
Participants	6	(*a*) Give the eligibility criteria, and the sources and methods of selection of participants. Describe methods of follow-up	Methods patients para 1
		(*b*) For matched studies, give matching criteria and number of exposed and unexposed	Not applicable (NA)
Variables	7	Clearly define all outcomes, exposures, predictors, potential confounders, and effect modifiers. Give diagnostic criteria, if applicable	Methods treatment para 1<br>Methods outcomes and trial populations para 1-4
Data sources/ measurement	8*	For each variable of interest, give sources of data and details of methods of assessment (measurement). Describe comparability of assessment methods if there is more than one group	Methods procedures para 1-5
Bias	9	Describe any efforts to address potential sources of bias	Methods procedures para 2-3

Study size	10	Explain how the study size was arrived at	Methods statistical analysis para 1
Quantitative variables	11	Explain how quantitative variables were handled in the analyses. If applicable, describe which groupings were chosen and why	Methods statistical analysis para 2
Statistical methods	12	(*a*) Describe all statistical methods, including those used to control for confounding	Methods statistical analysis para 1-2
		(*b*) Describe any methods used to examine subgroups and interactions	Methods statistical analysis para 1-2
		(*c*) Explain how missing data were addressed	Methods statistical analysis para 2
		(*d*) If applicable, explain how loss to follow-up was addressed	Methods statistical analysis para 2
		(*e*) Describe any sensitivity analyses	NA
**Results**			
Participants	13*	(a) Report numbers of individuals at each stage of study—eg numbers potentially eligible, examined for eligibility, confirmed eligible, included in the study, completing follow-up, and analysed	Figure 1
		(b) Give reasons for non-participation at each stage	Figure 1
		(c) Consider use of a flow diagram	Figure 1
Descriptive data	14*	(a) Give characteristics of study participants (eg demographic, clinical, social) and information on exposures and potential confounders	Table 1
		(b) Indicate number of participants with missing data for each variable of interest	Table 1, Figure 1
		(c) Summarise follow-up time (eg, average and total amount)	Figure 1
Outcome data	15*	Report numbers of outcome events or summary measures over time	Table 2, Figure 2, Figure 3,

			Supplementary Tables S1 to S10 and S1 Fig
Main results	16	(*a*) Give unadjusted estimates and, if applicable, confounder-adjusted estimates and their precision (eg, 95% confidence interval). Make clear which confounders were adjusted for and why they were included	Table 2, Figure 2, Figure 3, Supplementary Tables S1 to S10 and S1 Fig
		(*b*) Report category boundaries when continuous variables were categorized	NA
		(*c*) If relevant, consider translating estimates of relative risk into absolute risk for a meaningful time period	NA
Other analyses	17	Report other analyses done—eg analyses of subgroups and interactions, and sensitivity analyses	Fig 2 and Fig 3
**Discussion**			
Key results	18	Summarise key results with reference to study objectives	Discussion para 2
Limitations	19	Discuss limitations of the study, taking into account sources of potential bias or imprecision. Discuss both direction and magnitude of any potential bias	Discussion para 3
Interpretation	20	Give a cautious overall interpretation of results considering objectives, limitations, multiplicity of analyses, results from similar studies, and other relevant evidence	Discussion para 4
Generalisability	21	Discuss the generalisability (external validity) of the study results	Discussion para 7
**Other information**			
Funding	22	Give the source of funding and the role of the funders for the present study and, if applicable, for the original study on which the present article is based	Funding statement

*Give information separately for exposed and unexposed groups.

Note: An Explanation and Elaboration article discusses each checklist item and gives methodological background and published examples of transparent reporting. The STROBE checklist is best used in conjunction with this article (freely available on the Web sites of PLoS Medicine at http://www.plosmedicine.org/, Annals of Internal Medicine at http://www.annals.org/, and Epidemiology at http://www.epidem.com/). Information on the STROBE Initiative is available at http://www.strobe-statement.org.
