# Supplementary material for: Pyronaridine–artesunate real-world safety, tolerability, and effectiveness in malaria patients in 5 African countries: A single-arm, open-label, cohort event monitoring study
Source: PLoS Med. 2021 Jun 15;18(6):e1003669. doi: 10.1371/journal.pmed.1003669 (PMC8205155; doi:10.1371/journal.pmed.1003669)
Supplement: S1 Protocol and SAP — (PDF) [file pmed.1003669.s002.pdf]

Pyronaridine-artesunate real-world safety, tolerability, and effectiveness: a single arm, open-label, cohort event monitoring study in African malaria patients

Gaston Tona Lutete, *et al.*

This supplement contains the following items:

1. Original protocol (version 7.0), final protocol (version 9.0), summary of changes from version 7.0 to version 8.0 (summary 2) and from version 8.0 to version 9.0 (summary 3).

Version 7.0 is the first version of the protocol under which patients were recruited. Version 9.0 is the final version of the protocol under which patients were recruited. Previous versions were in draft form for discussion among investigators and were not implemented.

2. Original statistical analysis plan and amendments to the plan.

POST EMA-POSITIVE OPINION

PRODUCT: Pyramax
(pyronaridine-artesunate)

PHASE IIIB/IV COHORT EVENT MONITORING STUDY TO EVALUATE, IN REAL LIFE SETTING, THE SAFETY AND TOLERABILITY IN MALARIA PATIENTS OF THE FIXED-DOSE ARTEMISININ-BASED COMBINATION THERAPY PYRAMAX (PYRONARIDINE-ARTESUNATE)

STUDY NUMBER: SP-C-021-15

STUDY NAME: Phase IIIb/IV Safety Assessment of Pyramax

FINAL VERSION 7.0 DATE: 25/01/2017

CONFIDENTIAL

NAMES AND ADDRESSES:
Principal Investigators:

Cameroon: Dr Jude D. Bigoga , PhD

[redacted]

Ivory Coast: Dr. Serge-Brice Assi, MD, PhD

[redacted]

Congo: Dr Felix Koukouikila- Koussounda, PhD

[redacted]

Democratic Republic of Congo: Prof Gaston Tona Lutete

[redacted]

Gabon: Dr Ghyslain Mombo-Ngoma, MD

[redacted]

Other Responsible Parties:

Clinical monitoring

Family Health International Inc
[redacted]

Data Management and Statistical Analyses

ICON plc
[redacted]

**COORDINATING PRINCIPAL INVESTIGATOR**	Name :<br>Address:<br>Mob:<br>Tel:<br>E-mail:	Assoc Prof Dr Michael Ramharter<br>[redacted]
**PROJECT COORDINATOR**	Name:<br>Address:<br><br><br><br>Mob:<br>E-mail:	Prof Francine NTOUMI, PhD<br>[redacted]
**SPONSOR**<br><br>**SHIN POONG**	Name:<br>Address:<br><br>Mob:<br>Tel:<br>Fax:<br>E-mail:	Mr. Jangsik Shin<br>[redacted]
**FUNDER**<br><br>**MMV**	Name:<br><br>Address:<br><br>Mob:<br>Tel:<br>Fax:<br>E-mail:	Dr Stephan Duparc<br>[redacted]

Protocol Approval and Authorisation

[redacted]

Dr Robert Miller, MD
Pyramax QPPV

________________________ Date: ____________

Mr Jangsik Shin
Shin Poong Pharmaceutical Co., Ltd
Sponsor Pyramax Manager

________________________ Date: ____________

Assoc. Prof. Dr. Michael Ramharter, MD
Coordinating Principal Investigator

________________________ Date: ____________

Dr Isabelle Borghini PhD
Medicines for Malaria Venture
[redacted]

Dr Stephan Duparc, MD
Medicines for Malaria Venture
Chief Medical Officer

________________________ Date: ____________

Prof Francine Ntoumi, PhD
Central African Network on Tuberculosis, AIDS/HIV & Malaria
Project Coordinator

________________________ Date: ____________

Professor Stephen Allen, MD
Liverpool School of Tropical Medicine
DSMB Chairman

________________________ Date: ____________

Protocol Approval and Authorisation
Protocol Number: SP-C-021-15

Date:

Dr Robert Miller, MD

[redacted]

Mr Jangsik Shin
Shin Poong Pharmaceutical Co., Ltd
Sponsor Pyramax Manager

Date:

Assoc. Prof. Dr. Michael Ramharter, MD
Coordinating Principal Investigator

Date:

Dr Isabelle Borghini PhD
Medicines for Malaria Venture
Project Clinical Lead

Date:

Dr Stephan Duparc, MD
Medicines for Malaria Venture
Chief Medical Officer

Date:

Prof Francine Ntoumi, PhD
Central African Network on Tuberculosis, AIDS/HIV & Malaria
Project Coordinator

Date:

Professor Stephen Allen, MD
Liverpool School of Tropical Medicine
DSMB Chairman

Date:

Version 7.0 Date 25/01/2017: Property of SHIN POONG/MMV- strictly confidential

Protocol Approval and Authorisation
Protocol Number: SP-C-021-15

Date:

Dr Robert Miller, MD
Pyramax QPPV

Date:

Mr Jangsik Shin
Shin Poong Pharmaceutical Co., Ltd

[redacted]

Assoc. Prof. Dr. Michael Ramharter, MD
Coordinating Principal Investigator

Date:

Dr Isabelle Borghini PhD
Medicines for Malaria Venture
Project Clinical Lead

Date:

Dr Stephan Duparc, MD
Medicines for Malaria Venture
Chief Medical Officer

Date:

Prof Francine Ntoumi, PhD
Central African Network on Tuberculosis, AIDS/HIV & Malaria
Project Coordinator

Date:

Professor Stephen Allen, MD
Liverpool School of Tropical Medicine
DSMB Chairman

Date:

Protocol Approval and Authorisation
Protocol Number: SP-C-021-15

______________________________ Date: ______________

Dr Robert Miller, MD
Pyramax QPPV

______________________________ Date: ______________

Mr Jangsik Shin
Shin Poong Pharmaceutical Co., Ltd
Sponsor Pyramax Manager

______________________________ Date: ______________

Assoc. Prof. Dr. Michael Ramharter, MD
Coordinating Principal Investigator

[redacted]

Dr Isabelle Borghini PhD
Medicines for Malaria Venture
Project Clinical Lead

______________________________ Date: ______________

Dr Stephan Duparc, MD
Medicines for Malaria Venture
Chief Medical Officer

______________________________ Date: ______________

Prof Francine Ntoumi, PhD
Central African Network on Tuberculosis, AIDS/HIV & Malaria
Project Coordinator

______________________________ Date: ______________

Professor Stephen Allen, MD
Liverpool School of Tropical Medicine
DSMB Chairman

______________________________ Date: ______________

Protocol Approval and Authorisation

Protocol Number: SP-C-021-15

Date:

Dr Robert Miller, MD
Pyramax QPPV

Date:

Mr Jangsik Shin
Shin Poong Pharmaceutical Co., Ltd
Sponsor Pyramax Manager

Date:

Assoc. Prof. Dr. Michael Ramharter, MD
Coordinating Principal Investigator

Date:

Dr Isabelle Borghini PhD
Medicines for Malaria Venture
Project Clinical Lead

Date:

Dr Stephan Duparc, MD
Medicines for Malaria Venture
[redacted]

Prof Francine Ntoumi, PhD
Central African Network on Tuberculosis, AIDS/HIV & Malaria
Project Coordinator

[redacted]
Liverpool School of Tropical Medicine
DSMB Chairman

Date:

STUDY PROTOCOL AGREEMENT FORM

I, undersigned, ,………………………………………………………………, hereby certify that I have examined the protocol of the above referenced study for SHIN POONG and that I have thoroughly discussed the objectives of this study as well as the contents of this protocol with the Sponsor's representatives.

I agree to keep the contents of this protocol confidential and not to disclose it to a third party and to use it only for the purposes of this study.

I agree to perform this study according to this protocol and to meet the objectives, to comply with the ethical rules and to ensure patient safety.

I have well understood that in case the Sponsor may decide to prematurely end or to suspend this study at any time and for any reason, I would be informed of this decision in writing. Conversely, in case I would decide to prematurely end or to suspend this study, I commit to immediately inform the Sponsor of this decision in writing.

INVESTIGATOR FOR (Country):

NAME:

DATE:

Signature: ________________________________

TABLE OF CONTENTS

1. LIST OF ABBREVIATIONS 10

2. SUMMARY 12

STUDY FLOW CHART: 18

3. INTRODUCTION AND RATIONALE 19
3.1 Summary of current situation 19
3.2 Pyramax (pyronaridine-artesunate) 19
3.3 Malaria in Cameroon, Republic of Congo, DRC, Gabon and Ivory Coast 21
3.4 Project rationale 24
3.5 Proposed Sites participating in the study 24

4. STUDY OBJECTIVES 25
4.1 Primary objective 25
4.2 Secondary objectives 25

5. DESCRIPTION OF THE STUDY 26
5.1 Description of the study design 26
5.2 Study duration 27
5.3 Drug prescription and dosing 27
5.4 Evaluation criteria 28

6. STUDY POPULATION AND PATIENT SELECTION 29
6.1 Sample size 29
6.2 Selection criteria 29
6.2.1 Inclusion criteria 29
6.2.2 Exclusion criteria 29
6.3 Enrolment procedure 30
6.3.1 Investigational Sites 30
6.3.2 Patient selection 30
6.3.3 Patient numbering 30

7. STUDY PROCEDURE AND DATA COLLECTION 31
7.1 Visit planning 31
7.2 Collected data 31
7.2.1 Registration at the clinical study centre 31
7.2.2 Examination and data collection at inclusion 31
7.2.3 Follow-up visits/contacts and clinical tolerability follow-up questionnaire 32
7.3 Patients who did not receive follow-up visits/contacts or who have prematurely discontinued treatment 34
7.3.1 Premature treatment discontinuation 34
7.3.2 Patient lost to follow-up 34
7.4 Thick blood smear and blood spot logistics 35
7.5 Hepatitis panel logistics: 35
7.6 Vital signs and Physical examination 36

8. SUBJECT SAFETY 36
8.1 Training 36
8.2 Monitoring of adverse events 36
8.3 Definition of Adverse Event and Serious Adverse Event 37
8.4 Obligation of AE notification 37
8.5 Adverse event of special interest 39
8.6 Pregnancy 40
8.7 Sponsor obligations 41
8.7.1 Safety 41
8.7.2 Quality Assurance 41

9. STATISTICAL METHODS 41
9.1 Data analysis 41
9.2 Determination of sample size 42

10. DATA MANAGEMENT 43
10.1 Collection and validation of data 43
10.2 Quality control of data on site 43
10.3 Quality control of the preparation of thick blood smear slides 43
10.4 Quality control of thick blood smear sample reading 43
10.5 Data entry 44
10.6 Data cleaning and data base locking 44

11. TASKS AND RESPONSIBILITIES 44
11.1. Study specific DSMB 44
11.1.1. Responsibilities of the study specific DSMB 44
11.2. Responsibilities of the clinical staff of the health facilities 45
11.3. Responsibilities of the Sponsor 46

12. ETHICAL AND REGULATION ASPECTS 46
12.1. Regulations 46
12.2. Informed consent 47
12.3. Expected risks 47
12.4. Data protection and confidentiality 47
12.5. Insurance 48
12.6. Premature termination of the study 48
12.7. Competent authority inspections 48

13. PROTOCOL AMENDMENTS 49

14. DOCUMENTATION AND UTILISATION OF STUDY RESULTS 49
14.1. Properties and use of the study data and results 49
14.2 Publications 49

15. REFERENCES 50

16. ANNEXES..53
ANNEX 1..53
Declaration of Helsinki..53
ANNEX 2..59
Definition of Severe Malaria..59
ANNEX 3..60
Guidance for the evaluation of the intensity of clinical signs..60
ANNEX 4..63
Adverse Events of Special Interests: Drugs and the Liver..63

1. LIST OF ABBREVIATIONS

ACT	Artemisinin-based Combination Therapy
AE	Adverse Event
AESI	Adverse Event of Special Interest
AL	Artemether-lumefantrine
ALT	Alanine aminotransferase
An	*Anopheles*
AS	Artesunate
AS/AQ	Artesunate/amodiaquine
AST	Aspartate aminotransferase
BUN	Blood Urea Nitrogen
CANTAM	Central African Network on Tuberculosis, AIDS and Malaria
CEM	Cohort Event Monitoring
CEMREL	Centre de Recherches Médicales de Lambaréné
CHW	Community Health Worker
CMV	Cytomegalovirus
CNPV	National Pharmacovigilance Center of DRC
CPK	Creatinine phosphokinase
CRF	Case Report Form
DILI	Drug Induced Liver Injury
DOT	Direct Observational Treatment
DRC	Democratic Republic of Congo
eCRF	Electronic Case Report Form
EBV	Epstein Barr Virus
EMA	European Medicines Agency
HAV	Hepatitis A Virus
Hb	Haemoglobin
HBc	Hepatitis B core antigen
HBsAg	Hepatitis B surface Antigen
HCV	Hepatitis C Virus
HIV	Human immunodeficiency virus
IgM	Immunoglobulin M
IPTp	Intermittent Preventive Treatment in pregnancy
ITNs/LLINs	Insecticide Treated Nets / Long Lasting Insecticidal Nets
IRS	Indoor Residual Spraying
LDH	Lactate Dehydrogenase
LFTs	Liver function tests
MedDRA	Medical dictionary for Regulatory Activities
NMCP	National Malaria Control Programme
PCR	polymerase chain reaction
P. falciparum	*Plasmodium falciparum*
PI	Principal Investigator
QPPV	(Pyramax) Qualified Person for Pharmacovigilance
RDT	Rapid Diagnostic Test
RNA	Ribonucleic acid

SAE	Serious Adverse Event
SDV	Source Data Verification
SmPC	Summary of Product Characteristics
SP	Sulfadoxine-Pyrimethamine
SPPV	Shin Poong Pharmacovigilance
STM	Study Team Member
SUSAR	Suspected Unexpected Serious Adverse Reaction
ULN	Upper Limit of Normal
UPC-PV	Unité de Pharmacologie Clinique et Pharmacovigilance
WHO	World Health Organisation

2. SUMMARY

PRODUCT: Pyramax **Study N°: SP/CANTAM-C-021-15**

**Title**	Phase IIIb/IV Cohort Event Monitoring study to evaluate, in real life setting, the safety and tolerability in malaria patients of the fixed-dose Artemisinin-based Combination Therapy Pyramax (pyronaridine-artesunate).
**Location of the study**	The study will be performed in public health facilities of the CANTAM network in Central Africa and in public health facilities in West Africa where *Pyramax* will be used as treatment of uncomplicated malaria episodes, including repeat episodes.
**Objectives**	The main objectives of the study are to assess the safety of *Pyramax* particularly in patients with underlying liver function abnormalities, in patients who have co-morbid conditions, such as HIV, and also in very small children (<1 year of age)<br><br>**Primary**:<br>Evaluation and identification of the hepatic safety events of *Pyramax* in a sub group of patients enrolled with LFTs >2xULN from blood taken immediately prior to treatment without any clinical signs or symptoms of hepatotoxicity and with signs and symptoms of uncomplicated malaria confirmed by a Rapid Diagnostic Test (RDT) or microscopy (thick blood smear).<br><br>**Main Secondary:**<br>Comparison of the clinical hepatic safety of *Pyramax* between a cohort of patients enrolled with LFTs >2xULN and a cohort of patients enrolled with normal LFTs matched for demographic characteristics.<br><br>**Other Secondary**:<br>- Evaluation of the safety and tolerability in patients with normal and abnormal LFTs at inclusion according to any possible hepatic underlying disease based on the finding of the hepatitis panel and according to their HIV status (where known), their nutritional status, their age (children < 1 year of age in comparison to the rest of the treated population) and their weight.<br>- Evaluation of the safety and tolerability in retreated patients with a special focus on the hepatic safety.<br>- Evaluation of the potential for hypersensitivity reactions<br>- Evaluation of the relationship between the occurrence of hepatic related adverse events with or without LFT abnormalities and the administration of concomitant medications (in particular paracetamol, herbal medicines and antiretroviral drug).<br>- Evaluation of the efficacy and compliance of Pyramax when used under usual conditions (including unsupervised medication intake) in patients with signs and symptoms of uncomplicated malaria confirmed by a RDT or microscopy (thick blood smear).
**Design and duration of the study**	This is a non-comparative Cohort Event Monitoring study. All the patients for whom a diagnosis of uncomplicated malaria (according to the WHO criteria) is suspected, will undergo an RDT/microscopy. If the presence of malaria is confirmed and the patient (or parent/ guardian in case of children) provides

	his/her informed consent, he/she will be assigned a unique ID number and will be enrolled in the study. Blood will be taken for a blood spot for PCR analysis, for retrospective LFT assessment as well as for a retrospective viral hepatitis assessment to be done only in patients with abnormal LFTs. A blood sample will be taken for haematology (Hb mandatory). The first dose of *Pyramax* will be given under Direct Observational Treatment (DOT) conditions, but patients will take their second and third doses of *Pyramax* under usual conditions (including unsupervised medication intake). Patients will be also required to attend the health care facilities if they have any clinical signs or symptoms of hepatotoxicity.<br><br>A cohort of at least 2% of children who are <1 year of age will also be included for monitoring of liver function.<br><br>The patients will be visited at home at Day 7 ±1 day, by a community health worker (CHW), in order to capture the adverse events (AEs) experienced. In the case of signs or symptoms of hepatotoxicity or hepatic related AEs, the patients will be referred to the health facilities and blood will be taken on this occasion for LFT assessment and haematology (Hb mandatory). If a patient cannot be directly reached, the CHW must organize an appointment with this patient before Day 10. Special procedures will be followed in case of serious adverse events (SAEs) and/or severe adverse events and events classified as being of special interest (AESI) (see specific section).<br><br>At Day 28 ±2 days, all patients will be seen by a CHW for a final assessment, including blood taken for thick blood smear and spot. In the case of signs or symptoms of hepatotoxicity or hepatic related AEs occurring since the previous Day 7 visit, the patient will be referred to the health facilities and blood will be taken for LFTs and haematology (Hb mandatory) assessment.<br><br>Female patients must communicate to their village CHW or the study team if they get pregnant just before or after the start of the *Pyramax* treatment and for 2 months post-treatment. All pregnancies must be documented on the manufacturer's *Pyramax* Pregnancy Register. In these cases, the evolution of the pregnancy will be monitored with visits at 3, 6 and 9 months and at 7 days after the delivery. Information on the drugs taken during the pregnancy as well as AEs/SAEs/AESIs and the health status of the newborn(s) will be collected.
**Drug prescription and dosing**	Patients will be instructed to take one *Pyramax* dose every 24 hours over a period of three days, i.e. at Day 0, then after 24 hours (Day 1) and after 48 hours (Day 2) from the first administration.<br><br>The dose will be based on patient body weight, as specified in the summary of product characteristics and in the Investigator's Brochure. Two strengths and formulations of *Pyramax* will be provided to facilitate the dosing in adults, children, and young infants:<br>• Tablets of 180:60 mg of pyronaridine and artesunate (patients ≥20kg),<br>• Granules in sachets of 60:20 mg of pyronaridine and artesunate (patients 5-<20kg).
**Population**<br>Selection criteria	**Inclusion Criteria**<br>• Uncomplicated malaria (*Plasmodia* of any species) diagnosed as per national policies and in line with WHO recommendations:<br>o Fever or history of fever in the previous 24 h and/or the presence of anaemia, for which pallor of the palms appears to be the most

	reliable sign in young children.<br>o Confirmation of malaria by a parasitological diagnosis (RDT or Microscopy (thick blood smear). analysis).<br>• Weight ≥5 kg - < 20 kg (granules); ≥20 kg (tablets).<br>• Ability to take an oral medication.<br>• Ability and willingness to participate based on signed informed consent (a parent or a guardian has to sign for children below 18 years old) and on signed assent form for minors that could be required per national regulations in each participating country.<br>• The patient has to comply with all scheduled follow-up visits.<br><br>**Exclusion Criteria**<br>• Patients with clinical signs or symptoms of hepatic injury (such as nausea, abdominal pain associated with jaundice) or known severe liver disease (i.e. decompensated cirrhosis, Child-Pugh stage 3 or 4).<br>• Known allergy to artemisinin and/or to pyronaridine.<br>• Known pregnancy.<br>• Lactating women should be excluded if other anti-malarial treatments are available.<br>• Complicated malaria as per WHO definition (Annex 2)<br>• Patients that the investigator considers would be at particular risk if receiving an anti-malarial or if participating in the study.<br>• Patients having been treated with *Pyramax* in the previous 28 days.
Number of patients	Based on the primary objective, 120 malaria episodes in patients with baseline raised AST/ALT value >2xULN will be needed for a 81.6% probability to observe one severe hepatic event in this population. Given the screening rate in previous studies of 1.4% for malaria patients with AST/ALT >2xULN, recruitment will require an estimate of at least 8,572 malaria episodes. To mimic reality, patients can be included more than once in the study.
Number of sites	Public health facilities in four medical research centres with adjacent satellite sites of the CANTAM network in Central Africa and one centre in Ivory Coast. Additional sites may be added by consent of Coordinating PI, Sponsor and MMV.
**Study Flow Chart**	Reported below.
**Evaluation criteria**	**Primary:**<br>A safety analysis will be performed retrospectively on a sub-group of patients identified with abnormal baseline LFTs. In this sub-population, clinical hepatic safety will be assessed through the specific analysis of hepatic adverse events captured by the CHW at Day 7 ± 1 day or at any unforeseen visits in case of signs or symptoms of hepatotoxicity or hepatic related AEs, after confirmation by biochemical (LFT) assessment.<br><br>**Main Secondary:**<br>• Comparative analysis of the clinical hepatic safety (signs or symptoms of hepatotoxicity or hepatic related AEs, confirmed biologically) will be made between patients having abnormal LFTs (AST/ALT >2xULN) at enrolment versus a cohort of patients with normal LFTs matched for demographic characteristics.

	<u>**Other Secondary**</u>:<br>• A safety analysis will be performed in patients with normal and abnormal LFTs at inclusion according to any possible hepatic underlying disease based on the finding of the hepatitis panel and according to their HIV status (where known), their nutritional status, their age (children < 1 year of age in comparison to the rest of the treated population) and their weight.<br>• Safety (including hepatic safety) in retreated patients.<br>• Incidence of hypersensitivity reactions<br>o At any unforeseen visits in case of signs or symptoms of hepatotoxicity or hepatic related AEs, special efforts will be made to assure and record adequate follow-up of serious and/or severe adverse events and of the adverse events of special interest (see specific section).<br>o Biological/LFTs assessment (AST/ALT bilirubin total and conjugated, alkaline phosphatases). Assessment of baseline LFT results where signs or symptoms of hepatotoxicity or hepatic related AEs were reported at Day 28 or at any unforeseen visit.<br>• The relationship between the occurrence of hepatic related adverse events with or without LFT abnormalities and the administration of concomitant medications (in particular paracetamol, herbal medicines and antiretroviral drugs) will be evaluated.<br>• Crude Day 28 cure rate by species and PCR adjusted cure rate for Day 28 cure rate for *P. falciparum*<br>• Time between malaria episodes and frequency of repeat episodes before and after Day 28<br>• Compliance will be assessed by counting the number of tablets or sachets of granules dispensed but not taken, when the CHW visits the patient at home.
**AEs of special interest (AESIs)**	In case of an AESI confirmed by the study physician, the Sponsor and *Pyramax* QPPV shall be informed within 24 hours ([redacted], even if the event does not satisfy any condition of seriousness. Notification will occur through the use of an AESI form.<br><br>**1) Hepatic** AESIs should be reported as follows:<br><br>**a) Patients with normal LFTs at baseline:**<br>- Present with fatigue, nausea, abdominal pain, itching or signs of jaundice such as:<br>o dark urine,<br>o putty or mastic coloured stools,<br>o jaundice (yellowing of the whites of the eyes or skin).<br>and<br>- ALT or AST >3 x ULN<br><br>**b) Patients with baseline ALT/AST >2xULN**<br>- Present with fatigue, nausea, abdominal pain itching or signs of jaundice such as:<br>o dark urine,<br>o putty or mastic coloured stools,<br>o jaundice (yellowing of the whites of the eyes or skin).<br><br>and

	- ALT/AST >2 x baseline value<br>In all these cases, patients shall be directed to the designated health facilities for evaluation and recording of all relevant information. If necessary, the health worker will contact the study team member or PI / co-PI.<br>In addition to the LFT assessment at baseline for retrospective assessment, LFTs will be performed as soon as the patient arrives at the health facilities at any unforeseen visit and at Day 28+/2, if required because of clinical signs or symptoms of possible hepatotoxicity or hepatic related AEs.<br>In the event of biological signs of hepatotoxicity associated with clinical symptoms/suspected Drug Induced Liver Injury (DILI), blood will need to be taken/stored to perform the following additional tests (hepatitis panel):<br>• Hepatitis A, B, C (Anti-HAV IgM, Anti-HBc IgM, HBsAg, and hepatitis C RNA),<br>• Hepatitis E IgM antibody,<br>• Cytomegalovirus (CMV) testing polymerase chain reaction (PCR) testing,<br>• pp65 antigen, or IgM antibody,<br>• Epstein Barr virus (EBV) viral capsid antigen IgM antibody,<br>• Serum creatinine phosphokinase (CPK) and lactate dehydrogenase (LDH).<br>• Serum transferrin saturation and serum ferritin (diagnosis of hemochromatosis).<br>• Liver's biosynthetic capacity: albumin and prothrombin time (+ prealbumin, serum ceruloplasmin, procollagen III peptide, α-1-antitrypsin and α-feto protein when possible).<br>2) **Hypersensitivity** AESIs should be reported if patients show signs of hypersensitivity soon after treatment with Pyramax (particularly on repeat treatment) such as:<br>- Flushing<br>- The appearance of wheals / urticaria<br>- Breathlessness<br>- Faintness and/or fall in blood pressure
**Sample size**	Based on the primary objective, 120 malaria episodes in patients with baseline AST or ALT value >2xULN will be needed for a 81.6% probability to observe one severe hepatic event in this population, defined as:<br>• Appearance of clinical signs and symptoms of possible hepatotoxicity associated with a rise in ALT/AST >2 x baseline value.<br>Given the screening rate in previous studies of 1.4% for malaria patients with baseline AST/ALT > 2 x ULN, recruitment will require an estimate of at least 8,572 malaria episodes. To mimic reality, patients can be included more than once in the study.
**Statistical analysis**	The statistical analyses will be performed using an appropriate software package such as SAS , version 8.2 or later versions, SAS Institute, NC, Cary, USA.<br>A detailed statistical analysis plan will be generated within three months of study

	start. This plan may be revised during the course of the study in order to take into account protocol amendments, if any, and to address potential issues occurring during the study, that could affect the planned analyses. A final, approved, statistical analysis plan will be in place before the database lock.<br><br>**Analysis (All patients)**<br>The statistical analysis will be mainly descriptive. The estimates of the incidence of adverse events will be based on crude rates. All estimates will be complemented with appropriate 95% confidence intervals.<br><br>Adverse events will be coded in accordance with standard medical dictionary terminology (MedDRA) before database lock.<br><br>Incidences will be computed on the following classes of AEs:<br>▪ All AEs<br>▪ Serious AEs<br>▪ AEs of special interest (AESIs)<br>▪ AEs which caused early discontinuation of *Pyramax*<br>▪ AEs related to *Pyramax* treatment<br><br>Specific analyses will be carried-out on the AESIs. Correlation analyses will be carried-out in order to verify if their occurrence is more frequent with the previous/concomitant use of other drugs (specific classes to be determined in the statistical analysis plan), including antimalarials, in the presence of a co-morbidity (specific classes to be determined in the statistical analysis plan), or in the presence of parasitaemia.<br><br>Compliance will be computed for each patient as the ratio between the number of tablets or sachets actually taken and the number of tablets or sachets that the patient should have taken. Two groups of patients shall be described: compliant = 100% treatment compliance and non-compliant = other cases. The safety profile of compliant vs non-compliant patients will be compared.
**Safety reporting**	Safety data as well as any pregnancies will be reported to the Manufacturer and Sponsor Shin Poong [redacted] and will be managed as per national guidelines. In addition safety data will be reported by the Sponsor to the WHO collaborating centre for pharmacovigilance through the National Pharmacovigilance Center of DRC (CNPV).
**Ethical issues**	The protocol will be conducted in compliance with the Declaration of Helsinki, and the directives in the respective countries, in particular concerning the submission to the Ethics Committees and the protection of personal data.
**Dates**	Trial duration: Estimated to be approximately 18 to 24 months after study start, depending on enrolment being completed over 1 or 2 malaria seasons. However, the study will not be terminated until at least 120 malaria episodes in patients with baseline LFTs >2xULN are enrolled.

STUDY FLOW CHART:

**STUDY EXAMINATION**	Screening/ Inclusion Day 0/**V1** (health centre)	Day 7+/- 1 (home visit)	Day 28 +/-2 (home visit)	**Unforeseen Visit**
Age / sex / weight / height	X			
Vital signs (pulse, blood pressure)	X			
Body Temperature	X	X	X	X
Malaria symptoms / Hepatic symptoms / Other symptoms	X	X	X	X
Rapid Diagnostic Test (RDT)* or thick blood smear	X		X	X
Informed Consent	X			
Medical history	X			
Physical examination	X			
Previous medications (last month)	X			
Blood spot for PCR	X		X	X
Blood sample taken for AST/ALT, Total Bilirubin+/- Conjugated Bilirubin, Alkaline phosphatase and haematology (haemoglobin mandatory)	X[¶]	X([#])	X([#])	X([#])
Viral hepatitis assessment (hepatitis A, B, C, delta (if hepatitis B positive) and E)	X[a]			
Hepatitis panel		X([#])	X([#])	X([#])
Inclusion/Exclusion	X			
Prescription of *Pyramax*	X			
AE recording	X (only SAEs)	X	X	X
Concomitant medications	X	X	X	X
Compliance assessment		X		
Reported Pregnancy	X	X	X	X

*RDT only possible at D0, thick blood smear mandatory at D28.
¶ LFT samples will be only analyzed retrospectively
[a] Blood for viral hepatits assessment will be drawn from all patients at baseline. For patients retrospectively found with baseline AST or ALT >2xULN, the blood for viral hepatitis assessment will be sent to the central laboratory for analysis, while for all other patients (i.e. with AST and ALT ≤2xULN) the blood for viral hepatitis assessment will be destroyed.

In the case of clinical signs of possible hepatotoxicity/DILI, LFTs & Hb assessment will be performed as soon as the patient arrives at the health centre and blood for a full hepatitis panel will be taken in case of AESI or SAE related to hepatotoxicity.

3. INTRODUCTION AND RATIONALE

3.1 Summary of current situation

In response to the breakout and the spread of *Plasmodium* strains resistant to classical anti-malarials, the WHO recommends the use of artemisinin-based combination therapies (ACTs) in the treatment of uncomplicated malaria episodes.

New ACTs are entering the African market and will be used by the public health care system. Until now, these ACTs were prescribed to a limited number of patients and most of the time within strictly regulated clinical studies with patients meeting restrictive selection criteria. The collection of safety data and the risk evaluation based upon observational data are critical in order to evaluate the risk/benefit profile of a product through its life cycle and to inform the best use of medicines. In addition, the impact of the introduction of a particular ACT into the public health care system on the evolution of its efficacy and on the malaria morbidity and mortality is unknown and is worth investigating (1, 2, 3, 4, 5).

3.2 Pyramax (pyronaridine-artesunate)

Pyronaridine and artesunate are antimalarial agents with a history of clinical use, and artesunate has been used clinically in combination with other drugs. The action of artesunate is a rapid knock-down of the parasites, after which, the drug is quickly cleared as it has a short systemic half-life. Pyronaridine is also effective in the short-term but has an intermediate blood half-life thus providing a more sustained schizonticidal effect.

Artesunate, has been partnered with the established antimalarial agent pyronaridine to develop a three-day oral therapy *Pyramax*, for use in infants, children and adults to treat acute, uncomplicated malaria. The aim of the fixed dose combination of pyronaridine and artesunate is to provide a rapid reduction in parasitaemia with a short term regimen, thereby improving compliance and reducing the risk of recrudescence through the slower elimination of pyronaridine. This fixed combination of pyronaridine and artesunate has been studied extensively in both healthy volunteers and uncomplicated malaria patients and has EMA Positive Opinion under the article 58 procedure.

The safety database for the *Pyramax* (PA) clinical programme includes 4121 subjects who received at least 1 dose, administered either as the fixed-dose co-formulation or as pyronaridine tetraphosphate (PP) + artesunate (AS), or pyronaridine alone (Phase I), across four Phase I, two Phase II, five Phase III studies and a longitudinal, repeat dose Phase IIIb study. This safety database includes the subgroup of children treated with *Pyramax* granules in three studies, where the resulting safety profiles were comparable and consistent for both adult and paediatric formulations.

Pyramax treatment was generally well tolerated with the vast majority of adverse events being of mild or moderate intensity, with headache and gastrointestinal symptoms occurring most frequently.

The only notable safety finding more prevalent in subjects treated with *Pyramax* is associated with significant transient liver transaminase elevations in a small minority of subjects. The

early onset (Day 3-7) and rapid resolution of the liver transaminase elevations are consistent with a direct, low level toxicity seen. In the patient studies, the potential risk of liver injury, associated with this finding, was assessed taking into account subjects who had increases >3x ULN (3.4%), >5xULN (1.4%), and >10x ULN (0.4%) for transaminases, as well as those subjects who might qualify as Hy's law candidates (ALT >3xULN and total bilirubin >2xULN). Review of the data by an independent safety review board concluded that the risk of progressive liver injury, especially for a 3-day course of treatment, was very low. This was supported further by the fact that serious idiosyncratic hepatotoxicity typically begins weeks or months after starting therapy. Of note, all the raised values observed with *Pyramax* treatment returned to normal, the vast majority being normal at Day 28 or earlier (the precise timing confounded by timing of the visit dates). Overall, changes in liver function tests were mild with a small number of moderate cases (based on peak total bilirubin levels) as per the criteria of the Drug Induced Liver Injury Network. Furthermore there were no cases of liver failure, no encephalopathy, no evidence of coagulopathy and no evidence of a delayed effect.

Repeat dosing with *Pyramax* in Thai subjects with a single administration repeated after washout of 8 weeks showed no rises in transaminases after first or second dosing. Interim data from a longitudinal patient study conducted in West Africa where patients are retreated with *Pyramax* (tablets and granules) or comparator as many times as required over a 2-year period has shown that in the first 316 patients retreated at least once there is no increased risk in terms of liver function or adverse events on repeat dosing with periods between treatments of 27 and 307 days.

The Investigators should be familiar with the *Pyramax* Investigator Brochure (Version 8, 2016), in which the clinical and non-clinical studies are further described (6, 7, 8, 9, 10, 11, 12, 13, 14, 15, 16, 17).

3.2.1. Risk/benefit

Pyramax is a new option for the treatment of acute uncomplicated *P. falciparum* and *P. vivax* malaria, with a simple, once-a-day for three days regimen, and with no food restriction. The cumulative safety of pyronaridine-artesunate has been evaluated in over 4500 subjects and has shown that treatment was generally well tolerated. In order to get further assurance on safety, patients previously excluded or poorly represented in the Phase II/III clinical studies program will be included in the present cohort event monitoring study, such as patients with significantly raised liver enzymes, before treatment as well as patients with co-morbidities (e.g. HIV, hepatitis, or severe malnutrition).

For a full description of potential risks associated with Pyramax treatment, please refer to the EMA-approved Summary of Product Characteristics for *Pyramax* Tablets and *Pyramax* Granules (in particular to Sections 4.4 Special warnings and precautions for use, 4.6 Fertility, pregnancy and lactation and 4.8 Undesirable effects).

3.3 Malaria in Cameroon, Republic of Congo, DRC, Gabon and Ivory Coast

Cameroon

- **Yaoundé**

Malaria is endemic throughout Cameroon where it is responsible for 30% morbidity, 29% hospital consultations, 38% of cases among gravid women, 49% hospitalization and 45% deaths among children under five. Mfou, a forest area located in the Mefou and Afamba Division, Centre region of Cameroon (4°27'N and 11°38'E) has about 85 000 inhabitants, most of them living below the poverty line. The climate is equatorial with four seasons comprising two rainy seasons (March-June; August-October) and two dry seasons (November-February; June-July), which are of unequal durations (long and short season respectively). It has an annual average temperature of 22-25°C with rainfall averaging 1700-2500 mm/year. Houses are poorly constructed and close to bushes, increasing anopheles-human contact. Malaria is holoendemic in this locality and transmission is perennial, peaks generally during the rainy season and the rainy-dry transition period with about 25 infectious bites/year. The prevalence of malaria in the population is 77% and 40.6% among school aged children. *Plasmodium falciparum* is predominant parasite species responsible for over 97% all infections. The major vectors are *Anopheles gambiae* and *Anopheles funestus*.

- **Study sites**

The Principal site and Satellite sites are overseen by the Principal Investigator located at University of Yaoundé.
One satellite site is located at Etoug ebe, in the Mfoundi division, centre region of Cameroon (3°51'N and 11°31'E). It has a very particular relief consisting of hills and marshy valleys with average altitude of 760 m.
A further satellite site is based at Mfou District Hospital.

Nkolbisson is a region of Yaoundé and is the site location for the University of Yaoundé. It has four seasons made up of two rainy seasons (March-June; August-October) and two dry seasons (November-February; June-July). These seasons are of unequal durations (long and short season respectively). The annual average temperature is 25°C. With a population of about 200 000 inhabitants, a density of 200 inhabitants per km2, Nkolbisson is composed of almost all the ethnic groups of the country. The rainfall varies between 1500 mm and 2000 mm per year. Just like Mfou climatically, malaria is holoendemic with a perennial mode of transmission that peaks generally during the rainy season and the rainy-dry transition period. Malaria is main cause of consultation with huge burden among children under 5 years old (86.5%). *P. falciparum* is predominant malaria parasite species accounting for almost all the malaria cases. This parasite is responsible for the severe forms of malaria with *An. gambiae* being the predominant vector.

Republic of Congo

- **Study sites**

The Principal site and Satellite sites are overseen by the Principal Investigator who is located at Centre de Santé FCRM Massisia. The satellite sites are Centre de Santé de Madibou, Brazzaville and Hôpital de Base de Makélékélé, Brazzaville.

The study site is in Madibou a suburban area located in Makélékélé, one of the seven districts of Brazzaville. According to the data obtained from the General Census of Population in 2007, there were 1,373,382 inhabitants in Brazzaville, and Makélékélé, one of its seven districts, had 298,292 inhabitants. In this study area, Ngoko, Kinsana, and Mayala are part of Madibou; Ntietie, Malanda Yabi, Collinaud, and Nkoutou are part of Mbouono. A public health facility is located in Madibou which refers some patients to the district hospital in Makélékélé, located in the centre of the district. In the 1980s, malaria transmission had been described as intense and perennial, with an entomological inoculation rate of 200–1,000 infective bites/person/year. Since then, there has been no update with more recent entomological studies. Of 17,636 inhabitants in the study area, 3,058 (17.3%) were children under 10 years of age.

The *Pyramax* study will be conducted also at the hospital of Makélékélé (Southern area). The prevalence of clinical malaria infection ranges currently between 12 and 25% and 20 patients per day could be recruited during the study period.

Democratic Republic of Congo

- **Study sites**

The Principal site and Satellite sites are overseen by the Principal Investigator who is located at Universite de Kinshasa (UoKPV) and the satellite sites are based at Institut Médicale Évangélique de Kimpesé (IMEK) in Kimpese (bas Congo), Centre Hospitalier Mont-Amba (CHMA) in Kinshasa and Maternite de Binza (MB) in Kinshasa Delvaux.

In the region of Kinshasa, two seasons (rainy and dry) are accounted for and the rainy season lasts for 8 months. Malaria transmission is stable almost all the year. Malaria infection is high, around 54000 cases were reported last year with predominance in children under 5 years old (around 50%).

Country	Site	0 - 5 years	> 5 years	Pregnant women	Microscopy Positive	RDT positive
Democratic Republic of Congo	Kinshasa<br>Delvaux (Urban site)	5266	9254	7063	2886	2422
	Kinshasa<br>Kingasani (semi-rural site)<br>Institut Médicale Évangélique de Kimpesé<br>Kimpesé, Bas-Congo<br><br>Centre Hospitalier Mont-Amba<br>Université de Kinshasa	4817	6553	815	3742	3776

(18, 19, 20, 21, 22, 23, 24).

Gabon

- **Lambaréné**

Current WHO reports indicate that the entire population of Gabon (estimated at 1,670,000 in 2013) live in malaria endemic regions. Malaria transmission is perennial with a hyper-endemic transmission pattern. *P. falciparum* is responsible for 95% of human malaria cases and *P. ovale* (wallikeri and curtisi) and *P. malariae* are prevalent as mono- and co-infections throughout the country. Main vectors are *An. gambiae* and *An. funestus*.

Malaria incidence per 1000 persons/year is estimated at 228 (25). Children are the main patient population suffering from malaria in Gabon. Since 2003 first line treatments of *P. falciparum* malaria is AS/AQ and as a secondary option AL. ACTs are free of charge in the public sector since 2003. Since 2009 patients should receive a diagnostic test before treatment. ITNs/LLINs are recommended to all age groups since 2007, IRS is recommended since 2013, and IPTp is used to prevent malaria during pregnancy since 2003.

- Study site

The Principal Investigator is based at Albert Schweitzer Hospital; Lambaréné.
In 2014 malaria was responsible for 26% of hospital consultations among children (0-14 years of age) at a sentinel centre in central Gabon (Albert Schweitzer Hospital; Lambaréné 2014). Children <1 year of age constitute approximately 9% of paediatric malaria cases at this sentinel hospital in Lambaréné, Gabon. Children aged 1-5 years and 6-14 years constitute each 45.5% of paediatric malaria patients attending the hospital (Lambaréné 2014).

Ivory Coast

- **Agboville**

Ivory Coast has a high prevalence of parasitic infectious diseases including malaria, which is a significant public health problem.
Malaria is the leading cause of hospitalisation and consultation in the country's health centres. In 2010, 43% of morbidity in health care centres was due to malaria (NMCP, 2010 situational analysis).
Children under 5 years of age suffer 1 to 6 malaria's episodes per year, with an average of 3 malaria attacks per child. The number of attacks for adults is 1 to 3 episodes per person and per year, especially in rural areas (26).
Malaria accounts for 33% of all causes of death in the hospitals. (NMCP 2004 data)
The entomological inoculation rate varies by region from 150 to 400 infective bites per person and per year (27).
Ivory Coast, as most of the African countries, followed the WHO recommendations and changed its management policy of uncomplicated malaria. The protocol, ratified in January 2007 in Ivory Coast, recommends using ACTs for the treatment of uncomplicated malaria and supports the use of both ASAQ and artemether-lumefantrine as 1st line therapy.

- **Study sites**

The Principal site and Satellite sites are overseen by the Principal Investigator who is located at Institute Pierre Richet (IPR) and the satellite sites : Agboville, Azaguié Health Center, Centre de Sante Urbain d`Azaguie (CSUA) and Centre de Sante Urbin de Grand Morie (CSUGM).

The Pyramax study will be conducted in the health district of Agboville. This district is not far from Abidjan and is organized around several health centres for proper health coverage. The region has a stable malaria endemic setting reflecting the situation of southern Ivory Coast. The Health Centre of Azaguié was chosen initially to perform the prospective collection of safety data in real life. Additional centres at Grand Morié, Aboudé-Mandeké and Rubino may be opened in the event of difficulty of recruiting or in the case of closure of a center in one of the other countries participating in the study. According to the NMCP data, more than 2,000 malaria episodes could be included in this center in one year.

3.4 Project rationale

The project will take place in four Central African countries and in one West African country and it has dual objectives.

The first is to collect safety data in real-life conditions on the use of a medicine for which the safety profile is well known only through classical clinical studies. The second is to develop new methods for actively performing pharmacovigilance, which will subsequently be used for other anti-malarial drugs, and drugs in other therapeutic classes in sub-Saharan Africa.

This programme is being implemented in close collaboration with the National Malaria Control Programmes (NMCP) of each of the five countries and the Pharmacovigilance Department of the Sponsor. The collected data will be regularly submitted to an independent Drug Safety Monitoring Board (DSMB) committee in charge of safety monitoring, as well as to the Health Authorities and will be communicated by the Sponsor to the WHO collaborating Pharmacovigilance centre in Uppsala via the National Pharmacovigilance Center of DRC who act as a regional pharmacovigilance service in this project.

3.5 Proposed Sites participating in the study

1) Cameroon: University of Yaoundé 1, Yaoundé and the satellite sites Mfou District Hospital and Etoug-Ebe Baptist Health Centre (EBHC)
2) Republic of Congo, Centre de santé FCRM – Massisia and the satellite sites: Centre de santé de Madibou , Brazzaville and Hôpital de base de Makélékélé, Brazzaville
3) DRC : Universite de Kinshasa (UoKPV) and the satellite sites Institut Médicale Évangélique de Kimpesé (IMEK) in Kimpese (bas Congo), Centre Hospitalier Mont-Amba (CHMA) in Kinshasa and Maternite de Binza (MB) in Kinshasa Delvaux.
4) Gabon, Albert Schweitzer Hospital at Lambaréné (CERMEL),
5) Ivory Coast, Institute Pierre Richet (IPR) and the satellite sites : Agboville, Azaguié Health Center, Centre de Sante Urbain d`Azaguie (CSUA) and Centre de Sante Urbin de Grand Morie (CSUGM).

Other sites can be introduced by the Sponsor in conjunction with PI and MMV to optimise the execution of the study and achieve the required sample size.

4. STUDY OBJECTIVES

The main objectives of the study are to assess the safety of *Pyramax* particularly in patients with underlying liver function abnormalities, in patients who have co-morbid conditions, such as HIV (where known), and also in very small children (<1 year of age).

4.1 Primary objective

Evaluation and identification of the hepatic safety events of *Pyramax* in a subgroup of patients enrolled with LFTs >2xULN from blood taken immediately prior to treatment without any clinical signs or symptoms of hepatotoxicity and with signs and symptoms of uncomplicated malaria confirmed by a Rapid Diagnostic Test (RDT) or microscopy (thick blood smear).

4.2 Secondary objectives

- 4.2.1 Main secondary

Comparison of the clinical hepatic safety of *Pyramax* between a cohort of patients enrolled with LFTs >2xULN and a cohort of patients enrolled with normal LFTs matched for demographic characteristics.

- 4.2.2 Other secondary
  - Evaluation of the safety and tolerability in patients with normal and abnormal LFTs at inclusion according to any possible hepatic underlying disease based on the finding of the hepatitis panel and according to their HIV status (where known), their nutritional status, their age (children < 1 year of age in comparison to the rest of the treated population) and their weight.
  - Evaluation of the safety and tolerability in retreated patients with a special focus on the hepatic safety.
  - Evaluation of the potential for hypersensitivity.
  - Evaluation of the relationship between the occurrence of hepatic related adverse events with or without LFT abnormalities and the administration of concomitant medications (in particular paracetamol, herbal medicines and antiretroviral drug).
  - Evaluation of the efficacy and compliance of *Pyramax* when used under usual conditions (including unsupervised medication intake) in patients with signs and symptoms of uncomplicated malaria confirmed by a RDT or microscopy (thick blood smear).

5. DESCRIPTION OF THE STUDY

5.1 Description of the study design

This is a non-comparative Cohort Event Monitoring study of 8,572 malaria episodes to be conducted in Central and West Africa. Treatment is with *Pyramax* as per the product label and Investigator Brochure. Each patient will be provided with 3 days oral treatment with *Pyramax* to be administered on days 0, 1 & 2.

Total study duration for a single patient episode is 28±2 days.

A cohort of at least 2% of children who are <1 year of age will be included.

5.1.1. Study assessments

Screening/Inclusion/Day 0:
Patients for whom a diagnosis of uncomplicated malaria (according to WHO criteria) is suspected, will undergo an RDT or microscopy. If the presence of malaria is confirmed and the patient meets all inclusion criteria and none of the exclusion criteria and the patient (or parent/ guardian in case of children) provides his/her informed consent, he/she will be assigned a unique ID number and will be enrolled in the study. Blood will be taken for PCR analysis (blood spot), for retrospective LFTs (& Hb?) assessment as well as for a retrospective viral hepatitis assessment to be done only in those patients with abnormal baseline LFTs (AST or ALT >2 x ULN). A blood sample will be taken for Haematology (Hb mandatory).

The first dose of *Pyramax* will be given under Direct Observational Treatment (DOT) conditions, and patients will take their second and third doses of *Pyramax* under usual conditions (including unsupervised medication intake). Patients will be also informed of the requirement to attend the health care facilities if they have any clinical signs or symptoms of hepatotoxicity.

Day 7 or any unforeseen visit
The patients will be visited at home at Day 7 ± 1 day, by a community health worker (CHW), in order to capture the adverse events (AEs) experienced. The patient's body temperature will be taken. In the case of signs or symptoms of hepatotoxicity or hepatic related AEs, the patients will be referred to the health facilities and blood will be taken on this occasion for LFTs and haematology (minimum Hb) assessment as well as for a full hepatitis panel. These investigations will be performed immediately. If a patient cannot be directly reached, the CHW will have to organise an appointment with this patient before Day 10. Special procedures will be followed in case of serious adverse events (SAEs) and/or severe adverse events and events classified as being of special interest (AESI) (see specific section).

At any unforeseen visit, in addition to the procedures above, malaria diagnostics and a blood spot for PCR analysis will be undertaken.

Day 28

At Day 28 ±2 days, all patients will be seen by a CHW for a final assessment. At this occasion, blood will be taken for thick blood smear and spot. In the case of signs or symptoms of hepatotoxicity or hepatic related AEs occurring since the previous Day 7 visit, the patient will be referred to the health facilities and blood will be taken for LFTs and haematology (minimum Hb) assessment as well as for a full hepatitis panel.

Female patients must communicate to the local CHW or the study team if they get pregnant just before or after the start of the *Pyramax* treatment and for 2 months post-treatment. All pregnancies must be documented on the manufacturer's *Pyramax* Pregnancy Register. In these cases, the evolution of the pregnancy will be monitored with visits at 3, 6 and 9 months and 7 days after the delivery. Information on the drugs taken during the pregnancy as well as AEs/SAEs/AESIs and the health status of the newborn(s) will be collected.

5.2 Study duration

The study is anticipated to last approximately 18 to 24 months and will end when 1) the total number of patients (n=8,572) is reached and 2) at least 120 episodes of malaria in patients with raised liver enzymes at baseline have been recruited and 3) at least 2% (n=172) children below the age of 1 year will have been recruited into the study.

5.3 Drug prescription and dosing

The patients will be instructed to take one *Pyramax* (pyronaridine-artesunate) dose orally every 24 hours over a period of three consecutive days, i.e. at Day 0, then after 24 hours (Day 1) and after 48 hours (Day 2) from the first administration.

The dose will be based on body weight. Two formulations of *Pyramax* will be provided to facilitate the dosing in young infants, children and adults: 60:20mg granules presented in sachets and 180:60mg tablets of pyronaridine and artesunate respectively. For this study, *Pyramax* tablets are sent as bulk supplies to site in commercial blister packs of 90 tablets. Within the pack there are 10 blisters packs with 9 tablets in each.
Only the first dose will be administered under supervision in the clinic, the second and third doses will be taken by the subject at home.

**Body weight**	**No. of Sachets (granule formulation) per day**
5 to < 8 kg	1
8 to < 15 kg	2
15 to < 20 kg	3
	**Number of Tablets per day**
20 to < 24 kg	1

24 to <45 kg	2
45 to < 65 kg	3
≥65kg	4

Pyramax will be provided free of charge by the Sponsor, Shin Poong Pharmaceutical Co, Ltd.

5.4 Evaluation criteria

Primary evaluation criterion:
A safety analysis will be performed retrospectively on a sub-group of patients identified with abnormal baseline LFTs (AST/ALT >2xULN). In this sub-population, clinical hepatic safety will be assessed through the specific analysis of hepatic adverse events captured by the CHW at D7 ± 1 day or at any unforeseen visits in case of signs or symptoms of hepatotoxicity or hepatic related AEs, after confirmation by biochemistry assessment.

Main secondary evaluation criterion:
Comparative analysis of the clinical hepatic safety (signs or symptoms of hepatotoxicity or hepatic related AEs confirmed biologically) will be made between patients having abnormal LFTs (AST/ALT >2xULN) at enrolment versus a cohort of patients with normal LFTs matched for demographic characteristics.

Other secondary evaluation criteria
- A safety analysis will be performed in patients with normal and abnormal LFTs at inclusion according to any possible hepatic underlying disease based on the finding of the hepatitis panel and according to their HIV status (where known), their nutritional status, their age (children < 1 year of age in comparison to the rest of the treated population) and their weight.
- Safety (including hepatic safety) in retreated patients.
- Incidence of hypersensitivity reactions.
    - At any unforeseen visits in case of signs or symptoms of hepatotoxicity or hepatic related AEs, special efforts will be made to assure and record adequate follow-up of serious and/or severe adverse events and of the adverse events of special interest (see specific section).
    - Biological/LFTs assessment (AST/ALT bilirubin total and conjugated, alkaline phosphatases). Assessment of baseline LFT results where signs or symptoms of hepatotoxicity or hepatic related AEs were reported at Day 28 or at any unforeseen visit.
- The relationship between the occurrence of hepatic related adverse events with or without LFT abnormalities and the administration of concomitant medications (in particular paracetamol, herbal medicines and antiretroviral drugs) will be evaluated.
- Crude Day 28 cure rate by species and PCR-adjusted cure rate for Day 28 cure rate for *P. falciparum.*
- Time between malaria episodes and frequency of repeat episodes before and after Day 28.
- Compliance will be assessed by interview and counting the number of tablets or sachets of granules dispensed but not taken, when the CHW visits the patient at home.

6. STUDY POPULATION AND PATIENT SELECTION

6.1 Sample size

Based on the primary objective, 120 malaria episodes in patients with baseline raised AST/ALT value >2xULN will be needed for a 81.6% probability to observe one severe hepatic event in this population, defined as:

- Appearance of clinical signs and symptoms of possible hepatotoxicity associated with a rise in ALT/AST >2 x baseline value.

Given the screening rate in previous studies of 1.4% for malaria patients with AST/ALT >2xULN, recruitment will require an estimate of at least 8,572 malaria episodes. To mimic reality, patients can be included more than once in the study. At each new malaria episode, the patient will be assigned the same 8 first digits of his ID number, followed by E01, E02 etc, to illustrate repeated treatment 1, 2 etc.

The study duration is estimated to be approximately 18 to 24 months after study start, depending on enrolment being completed over 1 or 2 malaria seasons. However, the study will not be terminated until at least 120 malaria episodes in patients with LFTs >2xULN are enrolled and until at least 2% (n=172) children below the age of 1 year will be recruited.

6.2 Selection criteria

6.2.1 Inclusion criteria

- Uncomplicated malaria (*Plasmodia* of any species) diagnosed as per national policies and in line with WHO recommendations:
  - o Fever or history of fever in the previous 24 h and/or the presence of anaemia, for which pallor of the palms appears to be the most reliable sign in young children.
  - o Confirmation of malaria by a parasitological diagnosis (RDT or Microscopy (thick blood smear)).
- Weight ≥5 kg - <20 kg (granules); ≥20 kg (tablets).
- Ability to take an oral medication.
- Ability and willingness to participate based on signed informed consent (a parent or a guardian has to sign for children below 18 years old) and on signed assent form for minors that could be required per national regulations in each participating country.
- The patient has to comply with all scheduled follow-up visits.

6.2.2 Exclusion criteria

- Patients with clinical signs or symptoms of hepatic injury (such as nausea, abdominal pain associated with jaundice) or known severe liver disease (i.e. decompensated cirrhosis, Child-Pugh stage 3 or 4).
- Known allergy to artemisinin and/or to pyronaridine.

- Known pregnancy.
- Lactating women should be excluded if other anti-malarial treatments are available.
- Complicated malaria as per WHO 2012 definition (Annex 2).
- Patients that the investigator considers would be at particular risk if receiving an anti-malarial or if participating in the study.
- Patients having been treated with Pyramax in the previous 28 days.

6.3 Enrolment procedure

6.3.1 Investigational Sites

The Study will be conducted at selected health facilities from the following countries: Cameroon, Republic of Congo, DRC, Gabon and Ivory Coast. A list of sites is provided in section 3.5.
Additional sites can be added at the discretion of the Sponsor, MMV and PI if required.

6.3.2 Patient selection

At least 8,572 episodes of malaria are needed in the study. To mimic reality, patients can be included more than once in the study. A washout period of at least 28 days must be maintained between two consecutive treatments with *Pyramax*. A subset of 2% of children under the age of 1 year will be recruited.

A patient will be included in the study where he/she meets the selection criteria of the protocol and agrees to participate. In particular, to enter the study, the patient should accept: (i) that a small amount of blood is taken for the preparation of a blood spot, as well as for a LFTs assessment (ii) that the CHW visit him/her at home, (iii) that the data collected during those visits are registered and analysed and finally (iv) that, in case an SAE and/or an AE classified as severe and/or an AESI (see section 8.5) should occur he/she is to contact the CHW immediately.

6.3.3 Patient numbering

Each patient will be identified at the study site by a 10 digits number, referred to as patient ID number. The first digit is the country number, the following two digits identify the centre and the next four digits identify the patient enrolment number. A letter E will follow and the last two digits will indicate the malaria episode for which the patient is being treated, starting with 01 for the first time the patient is treated in the protocol. Each number will be unique and will not be assigned to another patient, including any premature discontinuations. At each subsequent malaria episode, the patient will be assigned the same first 8 digits of the ID, up to the letter "E" , followed by the corresponding Episode number, to illustrate repeated treatment (02, 03 etc….).

7. STUDY PROCEDURE AND DATA COLLECTION

7.1 Visit planning

Inclusion in the study and the required assessments for inclusion will be performed at the study centre or at one of the associated health facilities, on Day 0 during the consultation.

The first follow-up home visit with the patient will be performed by a trained CHW on Day 7 (± 1 day) after administration of the first *Pyramax* dose.

In addition, patients will be requested, in the event of any unusual significant symptom experienced during the 28 days following the treatment prescription, to visit their village CHW or contact directly the health agent who followed him/her during the study.

In case of an occurrence of an SAE, or an AE classified as severe or an AESI (see section 8.5), or if requiring medical care, the patient will be transferred to the study designated health facility by the village CHW in order to receive care for his/her symptoms (this will be an unforeseen visit) and to provide more detailed information on the event.

7.2 Collected data

7.2.1 Registration at the clinical study centre

Each patient visiting one of the designated study health facilities, screened for suspected uncomplicated or complicated malaria, regardless of their inclusion in the study, will be formally registered by the Study Team Member (STM) who has examined him/her. The date of consultation, the age and sex of the patient, the village or neighbourhood he/she lives in, the recent history of any anti-malarial treatment intake, the weight, the clinical signs, the diagnosis, the prescribed treatment (dose and duration) and any other medications within 7 days will be reported in this document. Where applicable the reason for not participating in the study (screen failure) will be documented. For patients consenting to the study, their unique patient ID will be recorded in the registry.

For the patients included in the study, the dates on which the patient's chart was provided to the CHW (by the STM) on which the CHW consigned the completed chart with data referring the follow-up contact at Day 7 (±1 day) at the STM will be included in the registry; those dates will be reported for traceability purposes.

7.2.2 Examination and data collection at inclusion

Where the patient agrees to participate in the study, the following procedures will be followed (Day 0):

- An informed consent form will have to be signed and will be filed with the medical records.
- The STM will assign a patient ID number to the patient, and record the following data:
    - For the Site Registry: last name, first name, full address (village/neighbourhood), registration number and patient ID number.

- o <u>In the Case Report Form (CRF*)</u>: the other information collected during this visit, i.e.:
    - Demographic data: patient ID number, initials, age, sex, weight, height and, vital signs (pulse and blood pressure), body temperature.
    - Physical examination will include general appearance, skin, head and neck, eyes, ears, nose, throat, lymph node palpation, lungs, heart, chest, abdomen, neurological function.
    - History of anti-malarial drugs taken during the 4 weeks before inclusion.
    - Any other treatment including traditional treatments, taken during the 2 weeks before inclusion.
    - Concomitant treatments including traditional medication and treatments prescribed when visiting the centre.
    - Malaria and associated disease symptoms to be reported.
    - Confirmation that a blood sample for malaria diagnosis (RDT or thick blood smear and blood spot for PCR), for haematology (Hb mandatory), for blood chemistry (at least AST, ALT, Bilirubin total and conjugated, ALP) and for viral hepatitis assessment has been drawn.
    - Review of inclusion and exclusion criteria, and eligibility for the study.
    - *Pyramax* treatment administration time. Dosage will be defined according to the weight of the patient. The patient will be instructed to take the medication with water according to the administration instructions. For all patients the first dose of the study drug will be directly administered at the facility and time of administration will be documented.

* The term CRF is used generically, the actual data will also be recorded in electronic format or eCRF. The detail will be described in the separate Data Capture and Management Plan. The terms CRF and eCRF are therefore used interchangeably.

7.2.3 Follow-up visits/contacts and clinical tolerability follow-up questionnaire

On Day 7 (± 1 day) (after administration of the first *Pyramax* dose) the CHW will visit the patient at home. He/she will then record the following data:

- His/her own 4 digit identification number (first two digits are the number of the health centre he/she is attached to, followed by a 2 digit number which will be assigned to him/her when the study starts).
- Date of the visit/contact.
- Clinical condition (recovered status or continuation of the illnesses).
- Body temperature will be taken.
- Compliance with the prescribed *Pyramax* treatment and in case the correct number of tablets or sachets of *Pyramax* was not taken, an explanation of the reason why the treatment was not followed should be provided.
- Description of the symptoms experienced by the patient in case of a positive answer to the question: "*Have you noticed any unusual event/symptom since your last visit at the centre?"*. Collect date of first manifestation of this/these event(s), symptom(s), intensity, treatment dis-/continuation, and evolution, in the specific section of the patient chart dedicated to the collection of Adverse Events.
- Pregnancy status will also be enquired about.
- Current treatments

In case, during his/her contact, the CHW assess that an event that has occurred is serious and/or severe, or detects an adverse event of special interest (see section 8.5), he/she should contact the STM and transfer the patient immediately to the health facility to be examined. If necessary in the opinion of the STM, he/she should contact the investigators so that they can visit the patient and characterise the status of the event and the causality. If the adverse event belongs to the hepatic category, after transfer to the health facility, blood will be drawn for chemistry (at least AST, ALT, Bilirubin total and conjugated, ALP) and haematology (Hb mandatory) and a full hepatitis panel. In that event, a specific adverse event report form will be completed by the STM or the investigators.
In addition, a thick blood smear will be prepared as well as a blood spot for future analysis.
In case any treatment is prescribed by a STM staff or the study doctor, it will be reported in the CRF and on the patient chart.

All the patients will be instructed to refer to the CHW or the designated STM should any adverse event occur within the 28 day after the start of the *Pyramax* administration.
In addition the regional hospital will be alerted of the ongoing study, and there will be a collection on a regular basis of any data related to patients who are treated in regional hospitals who have taken part in the study.

Female patients will be encouraged to communicate to the study team if they get pregnant within a period of two months after the start of the *Pyramax* treatment. In these cases, information on the evolution of the pregnancy will be collected at 3, 6 and 9 months and at 7 days after the delivery. Information on the drugs taken during the pregnancy as well as AEs/SAEs/AESIs and the health status of the newborn/s will be collected and reported in the patient chart and will be transmitted to the Manufacturer for documentation on the *Pyramax* pregnancy register.

On Day 28 (±2 day) the CHW will visit the patient for a final study visit at home. He/she will report:

- His/her own 4 digit identification number (first two digits are the number of the health centre he/she is attached to, followed by a 2 digit number which will be assigned to him/her when the study starts).
- Date of the visit/contact.
- Clinical condition (recovered status or continuation of the illnesses).
- Body temperature will be taken.
- Description of the symptoms experienced by the patient in case of a positive answer to the question: "*Have you noticed any unusual event/symptom since your last visit to the centre?"*. Collect date of first manifestation of this/these event(s), symptom(s), intensity, treatment dis-/continuation, and evolution, in the specific section of the patient chart dedicated to the collection of Adverse Events.
- Drops of blood will be taken for thick blood smear and spot for PCR.
- Pregnancy status will also be enquired about
- Current treatments

If the adverse event belongs to the hepatic category, after transfer to the health facility, blood should be drawn for chemistry (at least AST, ALT, Bilirubin total and conjugated, ALP) and haematology (Hb mandatory) and a full hepatitis panel.

Should the patient present at the clinic or make contact with the CHW, it might be required to conduct an unscheduled unforeseen visit to report:

- Date of the visit/contact.
- Clinical condition (recovered status or continuation of the illnesses).
- Body temperature will be recorded
- Description of the symptoms experienced by the patient in case of a positive answer to the question: "*Have you noticed any unusual event/symptom since your last visit to the centre?"*. Collect date of first manifestation of this/these event(s), symptom(s), intensity, treatment dis-/continuation, and evolution, in the specific section of the patient chart dedicated to the collection of Adverse Events.
- Drops of blood will be taken for thick blood smear and spot for PCR.
- .Pregnancy status will be checked.
- Current treatments.

7.3 Patients who did not receive follow-up visits/contacts or who have prematurely discontinued treatment

7.3.1 Premature treatment discontinuation

The reasons for any premature treatment discontinuation will be collected by the CHW or STM staff during his/her visits/contacts and if the reason for stopping the treatment is the occurrence of an adverse event, this will be described on the specific section of the patient chart and treated depending on its category (see section 8).

A patient can be withdrawn from the study for any of the following reasons:

- Death
- Loss to follow-up
- Withdrawal of consent
- The investigator or Sponsor believes (eg, for safety or tolerability reasons, such as a diagnosis of severe malaria or cerebral complications, or other adverse events) that it is in the best interest of the subject to discontinue treatment.

There will be no replacement for patients with premature discontinuation.

7.3.2 Patient lost to follow-up

The study staff will take all appropriate measures to perform each patient's follow-up visits. However, some patients may be lost to follow up. This information will be recorded in the patient's chart. A patient will be considered lost to follow up if he cannot be visited by the CHW before Day 10.

If a subject is lost to follow-up, all reasonable efforts must be made by the study site personnel to contact the subject and to determine endpoint status and the reason for discontinuation/withdrawal.

7.4 Thick blood smear and blood spot logistics

Blood slides will be performed and blood spots collected on Day 28 for all the participants. At the inclusion (Day 0) visit a thick blood smear can be replaced by RDT. All thick blood smear slides will be stained and stored for independent confirmation of presence or absence of malaria parasites. They will be examined in the laboratory by qualified personnel and according to the laboratory's Standard Operating Procedures. The results will be available for inclusion in the final study database.

7.5 Hepatitis panel logistics:

Viral hepatitis assessment at baseline:
On Day 0, after inclusion and before treatment, blood for viral hepatitis assessment will be drawn from all patients.
Viral hepatitis assessment shall include: hepatitis A, B, C, delta (if hepatitis B positive) and E.
Blood for viral hepatitis assessment will be kept frozen at -20°C.
For patients retrospectively found with baseline AST or ALT >2xULN, the frozen blood for viral hepatitis assessment will be sent to the central lab for analysis immediately. For all other patients (i.e. with AST and ALT <2xULN) the blood for baseline viral hepatitis assessment will be destroyed.

Full hepatitis panel:
During the study at D7, D28 or at any unforeseen visit, in case the CHW detects possible clinical signs or symptoms of hepatotoxicity, the patient will be referred to the health centre. At the health centre, blood will be drawn for at least AST, ALT, ALP, total and conjugated bilirubin and for a full hepatitis panel:

- Hepatitis A, B, C (Anti-HAV IgM, Anti-HBc IgM, HBsAg, and hepatitis C RNA),
- Hepatitis E IgM antibody,
- Cytomegalovirus (CMV) testing polymerase chain reaction (PCR) testing,
- pp65 antigen, or IgM antibody,
- Epstein Barr virus (EBV) viral capsid antigen IgM antibody,
- Serum creatinine phosphokinase (CPK) and lactate dehydrogenase (LDH),
- Alkaline phosphatase (if not already collected),
- Serum transferrin saturation and serum ferritin (diagnosis of hemochromatosis),
- Liver biosynthetic capacity: albumin and prothrombin time (+ prealbumin, serum ceruloplasmin, procollagen III peptide, α-1-antitrypsin and α-feto protein, when possible).

For patients with AST or ALT >3xULN (when baseline was normal) or for patients with AST or ALT >2x baseline value (when baseline value for AST or ALT >2xULN) the blood for full hepatitis panel should be sent as soon as possible to the central lab for analysis.

For all other patients, i.e. patients with AST and ALT <3xULN (when baseline was normal) or with AST and ALT <2x baseline value (when baseline value for AST or ALT >2xULN) the blood for the full hepatitis panel will be destroyed.

Version 7.0 Date 25/01/2017: Property of SHIN POONG/MMV- strictly confidential

7.6 Vital signs and Physical examination

Vitals signs include body temperature, weight, pulse rate and blood pressure. Blood pressure and pulse rate will be measured after the patient has rested for 3 minutes. The physical examination will include general appearance, skin, head and neck, eyes, ears, nose, throat, lymph node palpation, lungs, heart, chest, abdomen, neurological function.

8. SUBJECT SAFETY

8.1 Training

Before the start of the study, the following training programmes will be completed:

The health facility study staff will be trained on the technique of thick blood smear preparation and staining according to the local Standard Operating Procedure on the use and reading of the RDT, on the blood spots preparation and on the detection of signs or symptoms of hepatotoxicity and hypersensitivity reactions.

The medical staff of the clinical study centres as well as the CHW and the health care provider (HCP) will be trained on the tolerability profile of *Pyramax*, on pharmacovigilance procedures (adverse events recording, adverse events management etc.) and on other study specific procedures (registry maintenance, CRF completion, non-directional/open patient questioning, filling data sheets), data management systems and tools. The medical staff of the referral hospitals will additionally be trained on the detection of hepatotoxic events. GCP training will be provided as appropriate.

8.2 Monitoring of adverse events

Patient charts (completely filled in with the contact visit at Day 7 (±1 day), and with any AE(s) reported in the 28 days after the start of the treatment) will be captured in the CRF by the Principal Investigator (PI) of the study centre via the electronic capture system. Chart review meetings shall be organised regularly, during which the PI, his/her team and the people responsible for the pharmacovigilance in the country participating in the study shall review AE data (intensity, causality, date of event manifestation in relation to the start of treatment and the initial parasite species identified on the patient). Given the absence of pharmacovigilance systems in most countries involved in the study, the experience of Shin Poong Pharmacovigilance (SPPV) and national pharmacovigilance center of DRC (CNPV) will be used in this study. The principal investigator in each country and his team will make a first analysis of the safety reports received in order to assess completeness. Safety data should be entered immediately in the eCRF. Safety reports are reviewed by Shin Poong Pharmacovigilance (SPPV) for quality control. Designated personnel of CNPV / UPC-PV will have access to the trial database. They will have access to the electronic version of the CRF and can address a request to the principal investigator of the site concerned to add any further information needed for the analysis. CNPV will conduct the final review of the CRF for any patients who have had an adverse event, then CNPV will code each adverse event reported according to standard medical terminology (MedDRA). The PI will make a causality assessment. SPPV/CNPV/PI should be informed about any new data on a patient for whom a

causality link had already been established to reconsider its analysis and if necessary reassess the causality. Among these new data will be included any data acquired from the hepatitis panel results, where appropriate, the recurrence of the same adverse event in a patient who is retreated after a subsequent episode of uncomplicated malaria, etc. Data shall only be modified following an official query procedure. Safety data will be transmitted to the WHO Collaborating Centre for Pharmacovigilance via the CNPV of DRC to achieve a quality control of the review and to provide necessary feedback to CNPV and SPPV.
The SAE reporting must follow notification requirements in Section 8.4. SAEs and AESIs will be coded using the latest version of MedDRA by SPPV and will reconcile all SAEs in the SPPV database with those in the CNPV database on a regular basis.

An independent study-specific Data Safety Monitoring Board (DSMB) will conduct periodic safety reviews (see Section 11).

8.3 Definition of Adverse Event and Serious Adverse Event

An **Adverse Event** (AE) is a sign, symptom, syndrome, disease or biological anomaly suffered by a patient or a subject participating in a clinical study and receiving a medicinal product. This term does not imply a causal relationship with the concerned treatment. Clinical signs typical of an acute malaria episode will not be considered AEs unless the healthcare personnel considers these events as exceptional due to their evolution, their seriousness, or another factor related to these events (cf. annex 3 for grading of AEs).

A **Serious Adverse Event** (SAE) is an adverse event which:

- causes death or
- is life-threatening or
- necessitates hospitalisation or prolongs hospitalisation or
- results in persistent or significant disability/incapacity or
- is a congenital defect or malformation
- is another medically important event
- constitutes a possible Hy's Law case following the definition given in Annex 4.

A decision on medical and scientific grounds is required to assess whether an immediate notification of an event is warranted in other situations, such as medically important events which are not life-threatening, fatal or cause hospitalization, but could endanger the patient or required an intervention to prevent one of the above conditions developing.

Remark: Examples of such events are intensive care in the emergency room or at home to treat a bronchospasm; a haematological dyscrasia or convulsions not causing hospitalisation, or the development of drug addiction or abuse.

8.4 Obligation of AE notification

Adverse Events

The AEs, regardless of their seriousness and causal relationship to the study drug, arising between the first administration of study medication and the last study visit (as per the protocol), must all be recorded on the patient CRF (AE recording section). When possible, the

Version 7.0 Date 25/01/2017: Property of SHIN POONG/MMV- strictly confidential

symptoms must be regrouped within a single syndrome or diagnosis. The healthcare personnel shall have to specify the date of manifestation of the event, its intensity, final evolution, the measures taken and the treatment undertaken (if any).

Serious Adverse Events

In case of SAEs independent of causality, the healthcare personnel must immediately contact the PI / Investigator for validation of the seriousness and determination of the causality. Subsequently, the procedure described below must be followed:

- SEND (within 24 hours and fax or pdf scanned documents) the signed and dated copy of the “Adverse Event form” and the form “SAE complementary information” to SPPV (Tel/Fax[redacted])]
    - SPPV will send notification to CNPV-UPC
- CALL immediately (the same day) the medical monitor Dr. Selidji Todagbe Agnandji, responsible for safety in case of death or life-threatening events.
- CALL the local responsible person for pharmacovigilance of the project and where appropriate Shin Poong QPPV.
- INFORM within 48 hours the Ethic Committees of the occurrence of any serious adverse event as per local regulation.
- The follow-up of each fatal or life-threatening AE must be provided to the medical monitor and the SPPV within the same timeline as the initial report (within 24 hours and preferably by email).
- Attach to the chart the photocopy of all available results and examinations which were undertaken (and their date). Analysis results must be accompanied by the laboratory normal ranges. Special consideration shall be taken to ensure patient anonymity, and to the correct completion of the patient’s study specific identifier in the copies of the source documents provided to the Sponsor.

Follow-up of Adverse Events

The healthcare personnel must take all appropriate measures to protect the safety of the patients. Personnel must ensure to document follow-up of the evolution of each adverse event (clinical, biological or other) until resolution or until the stabilization of the patient’s status.

All new relevant information concerning the initial SAE shall be recorded on a form “SAE follow-up information form” by the nursing staff of the health centre, and shall be validated by the PI/co-PI who shall transfer the form to the local person/physician responsible for the pharmacovigilance of the project and the Shin Poong PV.

In case of a serious adverse event the patient must be followed until complete resolution and normalization of all analysis results, or until chronicity of the patient’s status. This can imply that the follow-up of the patient must continue beyond the period of follow-up per protocol, and that additional investigations could be requested by the Sponsor.

Version 7.0 Date 25/01/2017: Property of SHIN POONG/MMV- strictly confidential

8.5 Adverse event of special interest

An adverse event of special interest (AESI) is an adverse event for which on-going monitoring is appropriate within the context of the study. These events necessitate complementary examinations in order to characterize and understand them.

AESIs in this study can be related to:

- Hepatotoxicity
- Hypersensitivity

The study team, as well as the relevant referral facilities, should be trained to take particular notice of symptoms/signs suggestive of the AESIs in this study.

In case of an AESI confirmed by the study physician, the *Pyramax* QPPV shall be informed within 24 hours, even if the event does not satisfy any condition of seriousness. Notification will occur through the use of an AESI form to [redacted]

Hepatic AESIs should be reported as follows:

a) For patients with normal LFTs at baseline:

- Present with fatigue, nausea, abdominal pain, itching or signs of jaundice such as:
    - dark urine,
    - putty or mastic coloured stools,
    - jaundice (yellowing of the whites of the eyes or skin).

and

- ALT or AST >3 x ULN

b) For patients with baseline ALT/AST >2xULN:

- Present with fatigue, nausea, abdominal pain itching or signs of jaundice such as:
    - dark urine,
    - putty or mastic coloured stools,
    - jaundice (yellowing of the whites of the eyes or skin).

and

- ALT/AST >2 x baseline value

In all these cases, patients shall be directed to the designated health facilities for evaluation and recording of all relevant information. If necessary, the health worker will contact the study team member or PI / co-PI.

In addition to the immediate assessment of baseline LFT for retrospective assessment, LFTs will be performed as soon as the patient arrives at the health facilities at any unforeseen visit and at Day 28, if required because of clinical signs or symptoms of possible hepatotoxicity or hepatic related AEs.

In the event of biological signs of hepatotoxicity associated with clinical symptoms/suspected Drug Induced Liver Injury (DILI), blood will need to be taken to perform the following additional tests (full hepatitis panel): This blood sample shall be sent as soon as possible to the central lab for further analysis.

- Hepatitis A, B, C (Anti-HAV IgM, Anti-HBc IgM, HBsAg, and hepatitis C RNA),
- Hepatitis E IgM antibody,
- Cytomegalovirus (CMV) testing polymerase chain reaction (PCR) testing,
- pp65 antigen, or IgM antibody,
- Epstein Barr virus (EBV) viral capsid antigen IgM antibody,
- Serum creatinine phosphokinase (CPK) and lactate dehydrogenase (LDH),
- Alkaline phosphatase (if not already collected),
- Serum transferrin saturation and serum ferritin (diagnosis of hemochromatosis),
- Liver biosynthetic capacity: albumin and prothrombin time (+ prealbumin, serum ceruloplasmin, procollagen III peptide, α-1-antitrypsin and α-feto protein, when possible).

Hypersensitivity AESIs should be reported if patients show signs of hypersensitivity soon after treatment with Pyramax (particularly on repeat treatment) such as:

- Flushing
- The appearance of wheals / urticaria
- Breathlessness
- Faintness and/or fall in blood pressure

These events should be reported within 24 hours of knowledge to SPPV (Tel/Fax[redacted])]. The mechanism for reporting is the same as for an SAE; however, these will NOT be considered as SAEs unless they fulfil serious criteria as defined in Section 8.3.

8.6 Pregnancy

In case of pregnancy on initial presentation, the anti-malarial treatment shall be the one recommended by the NMCP. The patient will not be included in this study.

Female patients will be encouraged to communicate to their village CHW if they become pregnant within a period of two months after the start of the Pyramax treatment.

The evolution of the pregnancy will be monitored with visits at 3, 6 and 9 months and at 7 days after the delivery. Information on the drugs taken during the pregnancy as well as AEs/AESIs/SAEs and the health status of the newborn/s will be collected.

Pregnancy is not an adverse event unless the outcome of the pregnancy fulfils one of the serious criteria as defined in Section 8.3. Pregnancy should be reported by using the appropriate pregnancy report form within 24 hours of knowledge to SPPV (Tel/Fax [redacted])].

8.7 Sponsor obligations

8.7.1 Safety

Throughout the study, Shin Poong Pharmacovigilance (SPPV) shall report expeditiously all unexpected SAE's which are reasonably related to the study drug (SUSARs), to each local responsible person for pharmacovigilance and those who are responsible to inform the local Ethics Committees in accordance with their procedures, to the investigators and to EMA through Eudravigilance portal. SPPV, through the local investigators, will ensure submission of the reported events to the competent authorities in accordance with local regulations.

Shin Poong Pharmacovigilance (SPPV) will collaborate closely with the National Pharmacovigilance Center of DRC (CNPV-UPC) who act as a regional pharmacovigilance service and who report to the WHO collaborating Pharmacovigilance centre in Uppsala.

Shin Poong will also report expeditiously all expected SAE's which are reasonably related to the study drug, to the local responsible person for pharmacovigilance. The latter will submit the reported events to the competent authorities in accordance with local regulations. Shin Poong will also report to European Medicines Agency as required.

Any AE not listed as being an expected event in the SmPC and in this protocol shall be considered to be unexpected.

8.7.2 Quality Assurance

Steps to be taken to ensure the accuracy and reliability of data include the selection of qualified investigators and appropriate study sites, review of protocol procedures with the investigators and associated personnel before the study, periodic monitoring visits by the assigned study monitors on behalf of the Sponsor, and direct transmission of clinical laboratory data from central laboratories into the study database.
Written instructions will be provided for collection, preparation, and shipment of blood samples and for thick blood smear procedures (study manual).

Guidelines for eCRF completion will be provided and reviewed with study personnel before the start of the study.

CRFs will be reviewed for accuracy and completeness during on-site monitoring visits by the study monitor, any discrepancies will be resolved with the investigators or designee, as appropriate. Following ongoing transfers of the data into the clinical study database further validation and checks will be performed by Data Management and any queries will be communicated to the investigator or designee for subsequent resolution

9. STATISTICAL METHODS

9.1 Data analysis

The statistical analyses will be performed using an appropriate software package such as SAS , version 8.2 or later versions, SAS Institute, NC, Cary, USA.

A detailed statistical analysis plan will be generated within three months of study start. This plan may be revised during the course of the study in order to take into account protocol amendments, if any, and to address potential issues occurring during the study, that could affect the planned analyses. A final, approved, statistical analysis plan will be in place before the database lock.

A DSMB analysis plan will be generated on commencement of the study to address the requirements of the DSMB as outlined in the DSMB Charter.

Analysis (All patients)
The statistical analysis will be mainly descriptive. The estimates of the incidence of adverse events will be based on crude rates. All estimates will be complemented with appropriate 95% confidence intervals.

Adverse events will be coded in accordance with standard medical dictionary terminology (MedDRA) before database lock.

Incidences will be computed on the following classes of AEs:

- All AEs
- Serious AEs
- AEs of special interest (AESIs)
- AEs which caused early discontinuation of *Pyramax*
- AEs related to *Pyramax* treatment

Specific analyses will be carried-out on the AESIs. Correlation analyses will be carried-out in order to verify if their occurrence is more frequent with the previous/concomitant use of other drugs (specific classes to be determined in the statistical analysis plan), including antimalarials, in the presence of a co-morbidity (specific classes to be determined in the statistical analysis plan), or in presence of parasitaemia.

Compliance will be computed for each patient as the ratio between the number of tablets or sachets actually taken and the number of tablets or sachets that the patient should have taken. Two groups of patients shall be described: compliant = 100% treatment compliance and non-compliant = other cases. The safety profile of compliant vs non-compliant patients will be compared.

9.2 Determination of sample size

The sample size computation for the number of patients from the general malaria population who will present with AST or ALT >2xULN is based on a background incidence of 1.4% as seen in previous studies in a similar region. One hundred and twenty (120) malaria episodes in patients with AST or ALT >2xULN will need to be recruited to be able to detect one event with a probability of 81.6%. Severe hepatic event is defined as:

- Appearance of clinical signs and symptoms of possible hepatotoxicity associated with a rise in ALT/AST >2 x baseline value.

10. DATA MANAGEMENT

10.1 Collection and validation of data

Details of the data management processes will be documented in the Data Capture and Management Plan.

Screening data on all patients who present at the site will be captured on a specific screening registry (Section 7.2.1).

Patient data should be captured in the patient record at each site and documented in the study specific CRF.

Frequency of visit and responsibilities of the study monitor will be described in the Monitoring Plan. Source data verification (SDV) will be conducted on all informed consent forms, eligibility criteria, primary endpoint and AEs, SAEs and AESIs by the study monitor during monitoring visits.

Direct access to source documentation (medical records) must be allowed for the purpose of verifying that the data recorded in the eCRF are consistent with the original source data.

The study coordinator shall organise regular capture of these data by each study centre together with the results of the thick blood smears/RDT and blood spot of those patients.

Interim data will be assessed under the DSMB Charter by the independent DSMB.

10.2 Quality control of data on site

The plan for source data verification will be described in the Data Capture and Management Plan.

10.3 Quality control of the preparation of thick blood smear slides

The sample preparation technique will be quality controlled by one of the trainers. This person shall visit each site during the course of the study to verify the microscopic slides.

These visits shall be frequent at the start of the study, then may decrease in frequency if the quality of the samples is satisfactory. In case anomalies are detected during these study visits, or during the examination of the samples, the concerned Health Agent shall receive additional training.

A provisional schedule or calendar of supervision visits shall be initiated at the end of the training phase.

10.4 Quality control of thick blood smear sample reading

10% of the microscopic slides shall be quality controlled by a second qualified microscopist, who is independent of the centre where the initial microscopy took place. The procedures for quality control will be detailed in an ad hoc specification protocol.

Version 7.0 Date 25/01/2017: Property of SHIN POONG/MMV- strictly confidential

10.5 Data entry

The Data Capture and Management Plan will describe the data entry processes and controls.

10.6 Data cleaning and data base locking

The Data Capture and Management Plan will describe the data cleaning and database locking procedures.

At the end of the study the centrally aggregated data, will be verified according to the above mentioned documented procedures. Once declared clean, the database will be locked before data analysis is performed.

11. TASKS AND RESPONSIBILITIES

11.1. Study specific DSMB

This study specific DSMB is an independent board governed by a specific charter which identifies the frequency of meetings and methodology of reporting to the EMA via Shin Poong Pharmacovigilance. The minutes of the Open Sessions of the DSMB are to be made available to the EMA.

This DSMB shall be in charge of the regular review (not more than every four months) of the listings of the adverse events collected during the study. The panel shall also receive the notification of serious adverse events, adverse events classified as severe and adverse events of special interest.

In case a signal is identified by the DSMB, the *Pyramax* QPPV shall be contacted as soon as possible.
In a more general manner, all decisions taken by this DSMB shall be documented in writing and shall be transmitted to the Sponsor for the project located in the Republic of Korea and attached to the final study report.

MMV and Shin Poong delegates may be co-opted as non-voting members to the DSMB.

11.1.1. Responsibilities of the study specific DSMB

This study specific DSMB is composed of scientific experts in the field of pharmacovigilance and/or tropical medicine, hepatology and of one statistician. If required, it shall be possible to consult *ad hoc* experts in other fields (haematology, cardiology, dermatology etc.), who can join committee meetings.

The DSMB shall be involved in the approval of this protocol and its amendments, and be regularly updated on study progress, in particular on the number of inclusions. The DSMB shall receive data and reports issued, as required by the DSMB Charter, and prepared by Shin Poong Pharmacovigilance, as well as notifications of serious adverse events. If clear signals

of safety concerns are present, the DSMB can recommend halting the inclusion of further patients. The decision to halt the study shall be made after discussion and agreement between the members of the DSMB, Shin Poong and MMV. The *Pyramax* QPPV will be available at all times to discuss any safety findings in the context of the whole *Pyramax* safety database to aid in interpretation of any emerging safety signals.

The DSMB will have meetings or telephone conferences, prior to the start of the study to review the DSMB Charter, and then in accordance with the schedule provided in the Charter over the course of the study. At meetings the DSMB will review adverse events, consider and determine any possible safety signals, and discussion recommendations on the progress of the study. Finally, the DSMB shall meet for the release of the final results.

In between, conference calls can be organized at the request of the Sponsor, of an investigator, or of a member of one of the local pharmacovigilance monitoring committees.

All the decisions taken by the study specific DSMB shall be documented in writing; they will be transmitted to the Sponsor with the PIs of all the sites on copy and attached to the final study report. Any further local reporting will be in line with local regulations.

11.2. Responsibilities of the clinical staff of the health facilities

The staff shall be responsible to performing the study in accordance with this protocol and in accordance with the legislation and international guidelines under the direction of the local PI.

They are responsible for obtaining an informed consent prior to inclusion in the study, for completing the study documents (screening registry and patient CRF) and for recording all relevant data in relation to the study. Each staff member shall ensure that the information reported in the document is precise and accurate.

They must inform the patient of all relevant aspects of the study, including the information in the patient information sheet. All this information shall be provided to the patient in layman's terms. Patient confidentiality is paramount.

Prior to study inclusion, the informed consent form shall have to be personally completed (first name, surname), dated and signed by the patient, the patient's parent(s) or a guardian authorised representative. The person who has conveyed the information on the study to the patient shall also sign and date the informed consent form approved by the Ethics Committee. The informed consent forms will be translated into local languages for the benefit of those who do not understand English or French.

In case of patients unable to read and sign the patient information sheet and informed consent form, these documents will be read and explained to the patient in local language in the presence of a witness. The patient or the parent(s)/guardian in case of children below 18 years old, shall put her/his fingerprint on the informed consent form and the witness shall also sign the consent form to confirm that the patient has consented willingly.

A copy of the information sheet and the signed consent form shall be handed over to the patient or the parent/guardian.

11.3. Responsibilities of the Sponsor

The study Sponsor's responsibility is toward study teams at the study site and the health authorities and shall take all reasonable measures to ensure the good conduct of the study with regards to ethics, protocol compliance, integrity and validity of the information recorded in the patient CRF and registry as well as with regards to the availability of the adequate resources to ensure appropriate conduct of the study. In this respect, the principal function of the study management team is to help the investigator and the Sponsor to maintain a high level of ethical, scientific, technical and regulatory standards for all study-related aspects of ethics, regulations and administrative rules.

12. ETHICAL AND REGULATION ASPECTS

12.1. Regulations

The study shall be conducted in compliance with the text of the Declaration of Helsinki adopted by the 18th World Medical Association Assembly in 1964, and with its amendments (Annex 1).
The study will seek approval from the local institutional review boards / ethics committees (IRBs/ECs). This study will be undertaken only after the IEC/IRB has given full approval of the final protocol, amendments (if any), the informed consent form, applicable recruiting materials and the Sponsor has received a copy of this approval. This approval letter must be dated and must clearly identify the IEC/IRB and the documents and approved versions being approved. This study shall be conducted in accordance with the principles of the Good Clinical Practices (US, (4) & European, (5)).

The study shall be conducted in compliance with the international and national laws and regulations in effect, and in accordance with the applicable directives in Cameroon, Congo, DRC, Gabon and Ivory Coast in particular concerning the submission to the Ethics Committee and the protection of personal data.

Study related documents (protocol, case report from, informed consent form) shall be submitted to the National Ethics Committees or to the Institutions from the participating countries. Upon signature of the protocol, the Investigator accepts to respect the instructions and procedures described in the protocol, as well as the Good Clinical Practices and Good Laboratory Practices, to which he/she conforms.
The Investigator shall obtain from the patient or his/her legal representative, a signed (fingerprint and signature from a witness for patients unable to read and write), written consent. Assent will be obtained for minors able to understand the study procedures, according to the regulations in each of the participating countries. If informed consent is not obtained, the patient will not be enrolled. Furthermore, patients enrolled shall be entirely taken charge of for the treatment of their malaria for the duration of the study.

To ensure anonymity of study participants and confidentiality of the information collected, an identification number will be attributed to each participant at the time of study entry. Case

report forms and diaries will be kept in a locked room by the Sponsor. Only medical doctors having taken part in the study and the Sponsor will have access to these documents.

12.2. Informed consent

The investigator is responsible for ensuring that informed consent is obtained from each patient or legal representative and for obtaining the appropriate signatures and dates on the informed consent document prior to the performance of any protocol procedures and prior to the administration of study medication.

The Investigator shall explain to each study participant or his/her legal representative the nature of the study, its objective, the procedures involved, its risks and potential benefits and any discomfort it may generate. The patient must be informed that his/her participation is entirely voluntary, that he/she can withdraw from the study at any time and that withdrawal will not affect his/her subsequent medical treatment nor his relationship with the treating physician. The patient or his/her legal representative will sign on the informed consent sheet after having read and voluntarily agreed to it. A translation in local language for subjects unable to read and understand it will be available. Where the patient or his legal tutor are unable to read, in this case, an impartial witness should be present during the entire informed consent discussion. After inclusion, the patient may elect to withdraw from the study when he/she so wishes. The same level of attention will be dispensed to the patient.

12.3. Expected risks

Risks related to study medication

The study drug used in this study may cause headaches, nausea, vomiting, stomach ache, a slowing down of the heart rate (bradycardia), a decrease in the number of white blood cells, red blood cells (anemia) or blood platelets, an increase in the blood liver enzymes and a decrease in blood sugar levels (hypoglycemia).

These signs are generally moderate though they can vary from patient to patient. Risks that are currently unknown may exist.

Pyramax has not been studied in certain patient populations (for example in patients presenting with elevations in blood levels of liver enzymes). The uncertainty regarding the safety of use in such groups of patients is a foreseeable risk of participation in this study.

The safety of *Pyramax* in pregnant women has not been established. It is therefore not recommended in pregnant women. Women will be invited to inform the community health worker of any pregnancy occurring during the 2 months following the start of the *Pyramax* treatment. Pregnancy will be followed-up with visits at 3, 6 and 9 months and 7 days after delivery.

12.4. Data protection and confidentiality

The personal data of the patients which could be included in a Sponsor database or study database shall be treated in accordance with all local laws and regulations.

The investigator and the concerned personnel of the health centres and the study sites must keep all study documentation confidential and must take all necessary measures to prevent accidental or premature destruction of these documents.

The regulations or national laws in force on patient record keeping shall be applied.
At the time of archiving or management of the personal data pertaining to the nursing staff and/or patients, the Sponsor shall take all appropriate measures to secure and protect these data against access by a third non-authorised person.

12.5. Insurance

The Sponsor certifies to have subscribed for this study under its sponsorship, an insurance covering the responsibility of the investigator and his team, which is in agreement with the local laws and recommendations. The Sponsor's insurance shall not dismiss the investigator and his collaborators of their obligation to have their own civil liability insurance in line with the laws in force.

A copy of the insurance certificate shall be available for provision to investigators and / or ethics committees who would request it.

12.6. Premature termination of the study

The Sponsor can decide at any time and for whatever reason to prematurely suspend or interrupt the study. The decision and the justification shall be communicated in writing to the study specific DSMB.

The local authorities, EC and competent authorities shall have to be informed in line with local legislation.

The EMA will be advised of any decision and justification to prematurely terminate the study.

12.7. Competent authority inspections

Shin Poong and delegated responsible parties as well as the principal investigators accept to grant direct access to study source dossiers to auditors/inspectors for review, with the understanding that these people are bound by professional secrecy and shall not disclose any identity or medical information of a personal nature.

They shall undertake any effort in support of the audits and inspections by facilitating the access to equipment, data and necessary documents.

The confidentiality of the verified data and the protection of the patients shall be respected during these inspections.

All results and all information resulting from these inspections by the regulatory authorities shall be immediately communicated to the Sponsor.

Shin Poong and delegated responsible parties as well as the principal investigators shall take the appropriate measures in order to lead the corrective actions to all problems identified during the audits or inspections.

13. PROTOCOL AMENDMENTS

Each change to the protocol will be reported in a written amendment which will be signed by both the Principal Investigators and the Sponsor. The signed amendment will be added to the protocol.

Following the national legislation, the protocol amendment may require a regulatory submission (for example to the Ethics Committee) before implementation. Sometimes, an amendment may result in changes to the informed consent form. The Sponsor/Principal Investigators must receive an approval/favourable opinion from the Ethics Committee on the revised informed consent form before use.

14. DOCUMENTATION AND UTILISATION OF STUDY RESULTS

14.1. Properties and use of the study data and results

All results, data, documents and inventions obtained, directly or indirectly, from the trial, will be owned by the Sponsor unless a law or local regulation states otherwise. The Sponsor can use or exploit all results for their own use without any limitation of it's industrial property (territory, area, duration) in consultation with the study centres. The full data base will be the property of Shin Poong and will be utilized for producing the final study report as well as a safety report to be submitted to the European Medicine Agency. The investigators will have the right to participate with Shin Poong and MMV in the publication of such results.

14.2 Publications

A scientific committee will be formed, comprising the Sponsor, MMV and Coordinating Principal Investigator and others as agreed by these parties, with the responsibility for the presentations and/or publications of the results. The results of the study will be submitted to the Committee before each publication. Each subsequent presentation or publication should be approved by the scientific board.

The final decision on the publication of a manuscript/summary/presentation will be taken by the scientific committee after notification of the Sponsor in order to allow for an internal review and the possibility of providing comments. Each manuscript, summary, presentation will be submitted to the Shin Poong and MMV for internal review and possible comments at least 45 days before the submission to the journal and at least 20 days before the submssion of the summary. The Sponsor may request that their name and/or the name of one of their employees is present or not present on the publication. The Sponsor may delay each publication or communication during a limited time frame in order to protect the confidentiality or the proprietary information present in the document.

15. REFERENCES

1. World Health Organisation: World Malaria Report 2014 [http://www.who.int/malaria/publications/world_malaria_report_2014/en/].

2. World Health Organisation: Guidelines for the treatment of malaria (third edition), 2015 [http://www.who.int/malaria/publications/atoz/9789241549127/en/].

3. World Health Organisation: WHO List of Prequalified Medicinal Products [http://apps.who.int/prequal/query/ProductRegistry.aspx?list=ma].

4. World Health Organisation: A practical handbook on pharmacovigilance of antimalarial medicines, WHO/PSM/QSM/2007.8. [http://apps.who.int/medicinedocs/documents/s16881e/s16881e.pdf].

5. World Health Organization: A Practical Handbook, Third Edition, 2012. Management of Severe Malaria [http://apps.who.int/medicinedocs/documents/s20170en/s20170en.pdf].

6. Committee for Medicinal Products for Human Use (CHMP). Summary of opinion: Pyramax (pyronaridine tetraphosphate / artesunate) 2012. [http://www.ema.europa.eu/docs/en_GB/document_library/Other/2012/02/WC500122945.pdf].

7. Investigator Brochure PYRAMAX Pyronaridine Artesunate. Edition 8, January 2016.

8. Temple R. Hepatotoxicity Through the Years: Impact on the FDA, presented 2/12/2001 [http:www.fda.gov/downloads/Drugs/ScienceResearch/ResearchAreas/ucm122149.pdf].

9. Reuben A. Hy’s Law. Hepatology. 2004; 39:574-8.

10. Croft SL, Duparc S, Arbe-Barnes SJ, et al. Review of pyronaridine anti-malarial properties and product characteristics. Malar J 2012; 11: 270.

11. Ramharter M, Kurth F, Schreier AC, Nemeth J, Glasenapp I, Bélard S, Schlie M, Kammer J, Koumba PK, Cisse B, Mordmüller B, Lell B, Issifou S, Oeuvray C, Fleckenstein L, Kremsner PG. Fixed-dose pyronaridine-artesunate combination for treatment of uncomplicated falciparum malaria in pediatric patients in Gabon. J Infect Dis. 2008 Sep 15;198(6):911-9.

12. Tshefu AK, Gaye O, Kayentao K, Thompson R, Bhatt KM, Sesay SS, Bustos DG, Tjitra E, Bedu-Addo G, Borghini-Fuhrer I, Duparc S, Shin CS, Fleckenstein L; Pyronaridine-artesunate Study Team. Efficacy and safety of a fixed-dose oral combination of pyronaridine-artesunate compared with artemether-lumefantrine in

children and adults with uncomplicated *Plasmodium falciparum* malaria: a randomised non-inferiority trial. Lancet. 2010 Apr 24;375(9724):1457-67.

13. Kayentao K, Doumbo OK, Penali LK, et al. Pyronaridine-artesunate granules versus artemether-lumefantrine crushed tablets in children with *Plasmodium falciparum* malaria: a randomized controlled trial. Malar J 2012; 11: 364.

14. Poravuth Y, Socheat D, Rueangweerayut R, et al. Pyronaridine-artesunate versus chloroquine in patients with acute *Plasmodium vivax* malaria: a randomized, double-blind, non-inferiority trial. PLoS One 2011; 6(1).

15. Rueangweerayut R, Phyo AP, Uthaisin C, et al. Pyronaridine-artesunate versus mefloquine plus artesunate for malaria. N Engl J Med 2012; 366(14): 1298-309.

16. Duparc S, Borghini-Fuhrer I, Craft CJ, et al. Safety and efficacy of pyronaridine-artesunate in uncomplicated acute malaria: an integrated analysis of individual patient data from six randomized clinical trials. Malar J 2013; 12: 70.

17. Sagara I, Beavogui AH, Zongo I, Soulama I, Borghini-Fuhrer I, Fofana B, C Daouda, Somé AF, Coulibaly AS, Traore OB, Dara N, Kabore MJT, Thera I, Compaore YD, Sylla MM, Nikiema F, Diallo MS, Dicko A, Gil JP, Borrmann S, Duparc S, Miller RM, Doumbo OK, Shin J, Bjorkman A, Ouedraogo JB, Sirima SB and Djimdé AA. Safety and efficacy of retreatments with pyronaridine-artesunate in African malaria patients. Lancet Infect Dis 2016; 16: 189-198.

18. Anchang-Kimbi JK, Achidi EA, Apinjoh TO, Mugri RN, Chi HF, Tata RB, Nkegoum B, Mendimi JM, Sverremark-Ekström E, Troye-Blomberg M. (2014). Antenatal care visit attendance, intermittent preventive treatment during pregnancy (IPTp) and malaria parasitaemia at delivery. Malaria Journal, 13(1): 162. doi: 10.1186/1475-2875-13-162.

19. Tobias O. Apinjoh, Judith K. Anchang-Kimbi, Regina N. Mugri, Clarisse Njua-Yafi, Rolland B. Tata, Hanesh F. Chi, Delphine A. Tangoh, Beatrice T. Loh and Eric A. Achidi (2015) Determinants of Infant Susceptibility to Malaria During the First Year of Life in South Western Cameroon. Open Forum Infectious Diseases, DOI: 10.1093/ofid/ofv012

20. Tobias O Apinjoh, Judith K Anchang-Kimbi, Regina N Mugri, Robert N Vuchu, Delphine A Tangoh, Hanesh F Chi, Rolland B Tata, Charles Njumkeng, Clarisse Njua-Yafi and Eric A Achidi. (2015). The effect of Insecticide Treated Nets (ITNs) on Plasmodium falciparum infection in rural and semi-urban communities in the South West Region of Cameroon. Plos One. 10(2):e0116300.

21. Mathieu Ndounga, Pembe Mayengue Issamou, Prisca Nadine Casimiro, Félix Koukouikila-Koussounda, Michel Bitemo, Brunelle Diassivy Matondo, Lee Aymar Ndounga Diakou, Leonardo K Basco, Francine Ntoumi. Amodiaquine-artesunate versus artemether-lumefantrine for the treatment of acute uncomplicated malaria in

Congolese children under 10 years old living in a suburban area: a randomized study. Malaria Journal (In Press).

22. Francine Ntoumi, Jeannhey C.Vouvoungui, Rod Ibara, Miguel Landry, Anissa Sidibé. 2013. Malaria burden and case management in the Republic of Congo: Limited use and application of Rapid Diagnostic Tests results. BMC Public Health. Feb 14;13(1):135.

23. Zeukeng F, Tchinda VHM, Bigoga JD, Seumen CHT, Ndzi ES, Abonweh G, et al. (2014) Co-infections of Malaria and Geohelminthiasis in Two Rural Communities of Nkassomo and Vian in the Mfou Health District, Cameroon. PLoS Negl Trop Dis 8(10)

24. Tchinda GG, Atashili J, Achidi EA, Kamga HL, Njunda AL, Ndumbe PM (2012) Impact of Malaria on Hematological Parameters in People Living with HIV/AIDS Attending the Laquintinie Hospital in Douala, Cameroon. PLoS ONE 7

25. Malaria Atlas Project 2015 http://www.map.ox.ac.uk/

26. Henry M-C, C. Rogier, I. Nzeyimana, et al., Trop. Med. Int. Health 2003. Inland Valley rice production systems and malaria infection and disease in the Savannah of Côte d'Ivoire, TM&IH, Volume 8, Issue 5, May 2003, Pages 449–458

27. Ministère de la Santé et de l'Hygiène Publique. Institut Pierre Richet / Institut National de Santé Publique. 2009. Rapport sur le Profil entomologique du paludisme en Côte d'Ivoire (1956-2009). PP67.

16. ANNEXES

Annex 1: Declaration of Helsinki
Annex 2: Definition of Severe Malaria
Annex 3: Guidance for gradation of clinical symptoms
Annex 4: Adverse Events of Special Interests: Drugs and the Liver

ANNEX 1

Declaration of Helsinki

WORLD MEDICAL ASSOCIATION DECLARATION OF HELSINKI
Ethical Principles for Medical Research Involving Human Subjects

Adopted by the 18th WMA General Assembly, Helsinki, Finland, June 1964, and amended by the:
29th WMA General Assembly, Tokyo, Japan, October 1975
35th WMA General Assembly, Venice, Italy, October 1983
41st WMA General Assembly, Hong Kong, September 1989
48th WMA General Assembly, Somerset West, Republic of South Africa, October 1996
52nd WMA General Assembly, Edinburgh, Scotland, October 2000
53rd WMA General Assembly, Washington 2002 (Note of Clarification on paragraph 29 added)
55th WMA General Assembly, Tokyo 2004 (Note of Clarification on Paragraph 30 added)
59th WMA General Assembly, Seoul, October 2008

A. INTRODUCTION

1. The World Medical Association (WMA) has developed the Declaration of Helsinki as a statement of ethical principles for medical research involving human subjects**,** including research on identifiable human material and data.

   The Declaration is intended to be read as a whole and each of its constituent paragraphs should not be applied without consideration of all other relevant paragraphs.

2. Although the Declaration is addressed primarily to physicians, the WMA encourages other participants in medical research involving human subjects to adopt these principles.

3. It is the duty of the physician to promote and safeguard the health of patients, including those who are involved in medical research. The physician's knowledge and conscience are dedicated to the fulfilment of this duty.

4. The Declaration of Geneva of the WMA binds the physician with the words, “The health of my patient will be my first consideration,” and the International Code of

Medical Ethics declares that, “A physician shall act in the patient's best interest when providing medical care.”

5. Medical progress is based on research that ultimately must include studies involving human subjects. Populations that are underrepresented in medical research should be provided appropriate access to participation in research.

6. In medical research involving human subjects, the well-being of the individual research subject must take precedence over all other interests.

7. The primary purpose of medical research involving human subjects is to understand the causes, development and effects of diseases and improve preventive, diagnostic and therapeutic interventions (methods, procedures and treatments). Even the best current interventions must be evaluated continually through research for their safety, effectiveness, efficiency, accessibility and quality.

8. In medical practice and in medical research, most interventions involve risks and burdens.

9. Medical research is subject to ethical standards that promote respect for all human subjects and protect their health and rights. Some research populations are particularly vulnerable and need special protection. These include those who cannot give or refuse consent for themselves and those who may be vulnerable to coercion or undue influence.

10. Physicians should consider the ethical, legal and regulatory norms and standards for research involving human subjects in their own countries as well as applicable international norms and standards. No national or international ethical, legal or regulatory requirement should reduce or eliminate any of the protections for research subjects set forth in this Declaration.

B. PRINCIPLES FOR ALL MEDICAL RESEARCH

11. It is the duty of physicians who participate in medical research to protect the life, health, dignity, integrity, right to self-determination, privacy, and confidentiality of personal information of research subjects.

12. Medical research involving human subjects must conform to generally accepted scientific principles, be based on a thorough knowledge of the scientific literature, other relevant sources of information, and adequate laboratory and, as appropriate, animal experimentation. The welfare of animals used for research must be respected.

13. Appropriate caution must be exercised in the conduct of medical research that may harm the environment.

14. The design and performance of each research study involving human subjects must be clearly described in a research protocol. The protocol should contain a statement of the ethical considerations involved and should indicate how the principles in this

Declaration have been addressed. The protocol should include information regarding funding, sponsors, institutional affiliations, other potential conflicts of interest, incentives for subjects and provisions for treating and/or compensating subjects who are harmed as a consequence of participation in the research study. The protocol should describe arrangements for post-study access by study subjects to interventions identified as beneficial in the study or access to other appropriate care or benefits.

15. The research protocol must be submitted for consideration, comment, guidance and approval to a research ethics committee before the study begins. This committee must be independent of the researcher, the Sponsor and any other undue influence. It must take into consideration the laws and regulations of the country or countries in which the research is to be performed as well as applicable international norms and standards but these must not be allowed to reduce or eliminate any of the protections for research subjects set forth in this Declaration. The committee must have the right to monitor ongoing studies. The researcher must provide monitoring information to the committee, especially information about any serious adverse events. No change to the protocol may be made without consideration and approval by the committee.

16. Medical research involving human subjects must be conducted only by individuals with the appropriate scientific training and qualifications. Research on patients or healthy volunteers requires the supervision of a competent and appropriately qualified physician or other health care professional. The responsibility for the protection of research subjects must always rest with the physician or other health care professional and never the research subjects, even though they have given consent.

17. Medical research involving a disadvantaged or vulnerable population or community is only justified if the research is responsive to the health needs and priorities of this population or community and if there is a reasonable likelihood that this population or community stands to benefit from the results of the research.

18. Every medical research study involving human subjects must be preceded by careful assessment of predictable risks and burdens to the individuals and communities involved in the research in comparison with foreseeable benefits to them and to other individuals or communities affected by the condition under investigation.

19. Every clinical trial must be registered in a publicly accessible database before recruitment of the first subject.

20. Physicians may not participate in a research study involving human subjects unless they are confident that the risks involved have been adequately assessed and can be satisfactorily managed. Physicians must immediately stop a study when the risks are found to outweigh the potential benefits or when there is conclusive proof of positive and beneficial results.

21. Medical research involving human subjects may only be conducted if the importance of the objective outweighs the inherent risks and burdens to the research subjects.

22. Participation by competent individuals as subjects in medical research must be voluntary. Although it may be appropriate to consult family members or community leaders, no competent individual may be enrolled in a research study unless he or she freely agrees.

23. Every precaution must be taken to protect the privacy of research subjects and the confidentiality of their personal information and to minimize the impact of the study on their physical, mental and social integrity.

24. In medical research involving competent human subjects, each potential subject must be adequately informed of the aims, methods, sources of funding, any possible conflicts of interest, institutional affiliations of the researcher, the anticipated benefits and potential risks of the study and the discomfort it may entail, and any other relevant aspects of the study. The potential subject must be informed of the right to refuse to participate in the study or to withdraw consent to participate at any time without reprisal. Special attention should be given to the specific information needs of individual potential subjects as well as to the methods used to deliver the information. After ensuring that the potential subject has understood the information, the physician or another appropriately qualified individual must then seek the potential subject's freely-given informed consent, preferably in writing. If the consent cannot be expressed in writing, the non-written consent must be formally documented and witnessed.

25. For medical research using identifiable human material or data, physicians must normally seek consent for the collection, analysis, storage and/or reuse. There may be situations where consent would be impossible or impractical to obtain for such research or would pose a threat to the validity of the research. In such situations the research may be done only after consideration and approval of a research ethics committee.

26. When seeking informed consent for participation in a research study the physician should be particularly cautious if the potential subject is in a dependent relationship with the physician or may consent under duress. In such situations the informed consent should be sought by an appropriately qualified individual who is completely independent of this relationship.

27. For a potential research subject who is incompetent, the physician must seek informed consent from the legally authorized representative. These individuals must not be included in a research study that has no likelihood of benefit for them unless it is intended to promote the health of the population represented by the potential subject, the research cannot instead be performed with competent persons, and the research entails only minimal risk and minimal burden.

28. When a potential research subject who is deemed incompetent is able to give assent to decisions about participation in research, the physician must seek that assent in addition to the consent of the legally authorized representative. The potential subject's dissent should be respected.

29. Research involving subjects who are physically or mentally incapable of giving consent, for example, unconscious patients, may be done only if the physical or mental condition that prevents giving informed consent is a necessary characteristic of the research population. In such circumstances the physician should seek informed consent from the legally authorized representative. If no such representative is available and if the research cannot be delayed, the study may proceed without informed consent provided that the specific reasons for involving subjects with a condition that renders them unable to give informed consent have been stated in the research protocol and the study has been approved by a research ethics committee. Consent to remain in the research should be obtained as soon as possible from the subject or a legally authorized representative.

30. Authors, editors and publishers all have ethical obligations with regard to the publication of the results of research. Authors have a duty to make publicly available the results of their research on human subjects and are accountable for the completeness and accuracy of their reports. They should adhere to accepted guidelines for ethical reporting. Negative and inconclusive as well as positive results should be published or otherwise made publicly available. Sources of funding, institutional affiliations and conflicts of interest should be declared in the publication. Reports of research not in accordance with the principles of this Declaration should not be accepted for publication.

C. ADDITIONAL PRINCIPLES FOR MEDICAL RESEARCH COMBINED WITH MEDICAL CARE

31. The physician may combine medical research with medical care only to the extent that the research is justified by its potential preventive, diagnostic or therapeutic value and if the physician has good reason to believe that participation in the research study will not adversely affect the health of the patients who serve as research subjects.

32. The benefits, risks, burdens and effectiveness of a new intervention must be tested against those of the best current proven intervention, except in the following circumstances:
    - The use of placebo, or no treatment, is acceptable in studies where no current proven intervention exists; or
    - Where for compelling and scientifically sound methodological reasons the use of placebo is necessary to determine the efficacy or safety of an intervention and the patients who receive placebo or no treatment will not be subject to any risk of serious or irreversible harm. Extreme care must be taken to avoid abuse of this option.

33. At the conclusion of the study, patients entered into the study are entitled to be informed about the outcome of the study and to share any benefits that result from it, for example, access to interventions identified as beneficial in the study or to other appropriate care or benefits.

34. The physician must fully inform the patient which aspects of the care are related to the research. The refusal of a patient to participate in a study or the patient's decision to withdraw from the study must never interfere with the patient-physician relationship.

35. In the treatment of a patient, where proven interventions do not exist or have been ineffective, the physician, after seeking expert advice, with informed consent from the patient or a legally authorized representative, may use an unproven intervention if in the physician's judgement it offers hope of saving life, re-establishing health or alleviating suffering. Where possible, this intervention should be made the object of research, designed to evaluate its safety and efficacy. In all cases, new information should be recorded and, where appropriate, made publicly available.

ANNEX 2

Definition of Severe Malaria

WORLD HEALTH ORGANISATION CRITERIA 2014

Clinical features of severe malaria in children in high transmission area:

Group 1	Prostrate children (prostration is the inability to sit upright in a child normally able to do so or to drink in the case of children too young to sit). Three subgroups of increasing severity should be distinguished:<br>Prostrate but fully conscious<br>Prostrate with impaired consciousness but not in deep coma<br>Coma (the inability to localise a painful stimulus)<br>Respiratory distress (acidotic breathing):<br>Mild – sustained nasal flaring and/or mild intercostal indrawing (recession)<br>Severe – the presence of either marked indrawing (recession) of the bony structure of the lower chest wall or deep (acidotic) breathing<br>Shock compensated or decompensated (see definition above)
Group 2	Children who, although able to be treated with oral antimalarials, require supervised management because of the risk of clinical deterioration but who show none of the features of group 1 (above)*. These include children with any of the following:<br>Haemoglobin <5 g/dl or haematocrit < 15%<br>2 or more convulsions within a 24-h period<br>Haemoglobinuria (blackwater)<br>Jaundice
Group 3	Children who require parenteral treatment because of persistent vomiting but who lack any specific clinical or laboratory features of groups 1 or 2 (above)

*If parasite counts are immediately available, a parasitaemia over 10% should be included in group 2.
Children are defined as <12 years old.

Clinical features of severe malaria in adults:

Group 1	Adults at increased risk of dying immediately who require parenteral antimalarials and appropriate supportive therapy<br>Prostrated or obtunded adults (prostration is the inability to sit or to drink). Four subgroups of increasing severity should be distinguished:<br>Prostrate but fully conscious<br>Prostrate with impaired consciousness but not in deep coma (GCS > 11)<br>Confusion and agitation (GCS > 11)<br>Coma (the inability to localise a painful stimulus) (GCS < 11)<br>Respiratory distress (acidotic breathing)<br>Mild – sustained nasal flaring and/or mild intercostal indrawing (recession)<br>Severe – the presence of either marked indrawing (recession) of the bony structure of the lower chest wall or deep (acidotic) breathing<br>Shock (hypotension:systolic BP < 80 mmHg)<br>Anuria<br>Significant upper gastrointestinal haemorrhage
Group 2	Adults who, although able to be treated with oral ACTs, require supervised management because of the risk of clinical deterioration but who show none of the features of group 1 (above)*. This group includes adults with any of the following:<br>Haemoglobin <7 g/dl or haematocrit <20%<br>One or more convulsions within a 24-h period<br>Haemoglobinuria (blackwater)<br>Jaundice
Group 3	Adults who require parenteral treatment because of persistent vomiting but who lack any specific clinical or laboratory features of groups 1 or 2 (above)

*If parasite counts are immediately available a parasitaemia over 4% should be included in group 2.

ANNEX 3

Guidance for the evaluation of the intensity of clinical signs

	Grade 1<br>MILD	Grade 2<br>MODERATE	Grade 3<br>SEVERE	Grade 4<br>LIFE-THREATENING
**Fever in the following 24h**	N/A	Yes	N/A	N/A
**Weakness**	Small decrease in activity, keeps playing	Moderate decrease in activity, has difficulty to play	No activities, stopped playing	Lethargy
**Muscular or articular pain***	Localised pain, weak intensity	Diffuse pain, weak intensity	Real weakness; limited functions	N/A
**Cephalgia***	Weak, not requiring treatment	Fluctuating, moderate, requiring treatment	Severe, responding to an initial narcotic treatment	Refractory, requiring a repeated narcotic treatment
**Anorexia**	Reduced appetite but still eating solid foods	Reduced appetite, avoiding all solid foods	Refusing breast milk, very reduced appetite, taking neither liquids nor solids<br>(< 2 years ≤ 12 h;<br>> 2 years ≤ 24 h)	Refusing breast milk, very reduced appetite, taking neither liquids nor solids<br>(< 2 years ≤ 12 h;<br>> 2 years ≤ 24 h)
**Nausea***	Small discomfort; continues to ingest normally	Moderate discomfort; ingestion significantly reduced; certain activities are limited	Severe discomfort; no significant ingestion, limited activities	Minimal ingestion of liquids
**Vomiting**	Transient vomiting	Moderate or occasional vomiting	Orthostatic hypotension requiring an infusion	Shock for which hospitalisation for infusion is required
**Abdominal pain***	Weak	Moderate, no treatment required	Moderate to severe – treatment required	Severe – hospitalisation for treatment
**Diarrhea**	Transient; 3-4 liquid stools /day	5–7 liquid stools /day	Orthostatic hypotension or >7 liquid stools /day or infusion required	Shock for which hospitalisation for infusion is required
**Cough**	Transient, no treatment required	Continuous, requiring a treatment	Irrepressible	Cyanosis, violent cough, very difficult breathing
**Pruritus**	Pruritus without rash	Pruritus with rash or Pruritus without eruptions disturbing the sleep	Moderate urticaria	Severe urticaria, anaphylaxis, Quincke's Oedema
**Tinnitus***	Weak	Moderate	Severe including hearing loss	N/A
**Behavioural changes**	Minor concentration difficulties; confusion or minor agitation, normal daily activities; no treatment required	Moderate confusion or agitation; daily activities slightly impacted; minimal treatment required	Severe confusion or agitation; requiring assistance with daily activities; treatment required	Toxic psychosis; hospitalisation required
**Flu symptoms**	Minor nasal congestion, minor rhinitis, without cough	Moderate nasal congestion, moderate rhinitis, with cough	N/A<br>(if severe, classify the symptoms individually)	N/A (in life-threatening circumstances, classify the symptoms individually)

*** Applicable only to children ≥ 3 ans. Respond N/A if younger or for those not able to respond.**

Reference – Based upon the WHO toxicity grading scale for determining the severity of adverse events

	Grade 1 MILD	Grade 2 MODERATE	Grade 3 SEVERE	Grade 4 LIFE-THREATENIING
**Convulsion**	N/A	N/A	Localised or general	Epileptic state
**Temperature* (tympanic)**	38.0-38.4°C	38,5-40.0°C	> 40.0°C	Persistent fever, equal to >40° during more than 5 days
**Dehydration ****	Normal skin to touch, hydrated mucosa, tears are present, normal eyes, flat fontanel, consolable, regular pulse, normal micturition	Dry skin, dry mucosa, sunken eyes, lack of tears, soft fontanel, irritable, slightly accelerated pulse, reduced micturition	Moist and cold skin, dried skin, completely sunken eyes, no tears, sunken fontanel, lethargic, rapid pulse, no micturition	
**Facial oedema**	Present, minor swelling of the eyes	Moderate swelling of the eyes and the face	Severe swelling of the eyes, the face and the mucosa – impossible to open the eyes	The respiratory system is affected
**Icterus**	Mild subconjunctival icterus	Moderate subconjunctival icterus, mucosa moderately yellow	Severe subconjunctival icterus and icterus of the skin	N/A
**Thorax**	Slightly accelerated breathing (with regard to age and temperature), evanescent or localised rhonci	Moderately accelerated breathing, diffuse or persistent rhonchi	Rapid breathing (< 2 months > 60, 2-12 months > 50, 1-5 years > 40, adults > 30)* dilatation and retraction of the nostrils	Cyanosis
**Abdomen**	Normal abdominal sounds, slight local sensibility and/or hepatomegaly exceeding the costal margin with 2-4 cm and/or palpable spleen and/or presence of umbilical hernia	Slightly abnormal abdominal signs or moderate or diffuse sensibility and/or mild or moderate hepatomegaly (exceeding the costal margin with 4-6 cm) and/or grade 4 splenomegaly (palpable spleen until midway between the navel and the public symphysis	Very abnormal abdominal sounds, pain and resistance during palpation and/or hepatomegaly exceeding the costal margin > 6 cm and/or splenomegaly grade 5 (palpable spleen at beyond midway between the navel and the public symphysis)	No abdominal sound. Contracture
**Skin†**	Localised skin eruption, erythema or pruritus	Desquamation, diffuse maculopapular eruption	Vesicles, moist desquamation or ulceration	Exfoliative dermatitis, implication of mucosa or multiform erythema or suspicion of Stevens Johnson or a necrosis requiring a surgical intervention

Version 7.0 Date 25/01/2017: Property of SHIN POONG/MMV- strictly confidential

	Grade 1<br>MILD	Grade 2<br>MODERATE	Grade 3<br>SEVERE	Grade 4<br>LIFE-THREATENIING
**Hearing**	< 4 years : N/A<br>> 4 years: unilateral reduction of the hearing capacity	< 4 years: N/A<br>> 4 years: severe bilateral or unilateral reduction of the hearing capacity	< 4 years: Any reduction of the hearing capacity<br>> 4 ANS : severe bilateral reduction of the hearing capacity	N/A
**Attempt to pick-up tablets**	Difficulty in grasping the tablet though capable	Cannot grasp the tablet without dropping the tablet	Cannot grasp the tablet	N/A
**Other symptoms/ signs (not described elsewhere)**	No treatment; only monitoring	May require a minimal intervention and monitoring	Requires medical care and possible hospitalisation	Requires active medical care or hospitalisation

***Reference – DMID Toxicity Table for Children, May 2001, drug fever (rectal)**
**** Reference - The Harriet Lane Handbook, 15th edition, 2000**
† Reference - WHO toxicity grading scale for determining the severity of adverse events

ANNEX 4

Adverse Events of Special Interests: Drugs and the Liver

Checklist for Serious Liver Reactions:

The following liver reactions have to be considered as potentially serious:

Possible Hy's law case is defined as a subject with any value of ALT or AST >3xULN together WITH an increase in bilirubin to a value > 2xULN (>35% direct) and NOT associated to an ALP value > 2xULN.

Other definitions of hepatic adverse events of special interest (AESIs):

For patients with normal LFTs at baseline:

- Present with fatigue, nausea, abdominal pain itching or signs of jaundice such as:
    - dark urine,
    - putty or mastic coloured stools,
    - jaundice (yellowing of the whites of the eyes or skin).

and

- ALT or AST >3 x ULN.

For patients with baseline ALT/AST >2xULN:

- Present with fatigue, nausea, abdominal pain itching or signs of jaundice such as:
    - dark urine,
    - putty or mastic coloured stools,
    - jaundice (yellowing of the whites of the eyes or skin).

and

- ALT/AST >2 x baseline value.

Procedures to follow if possible Hy's law criteria are met:

- Stop the study medication and concomitant medications if they are not medically necessary
- Report the event as an SAE.
- Alert the Sponsor (via the study Medical Monitor or the Pharmacovigilance (Shin Poong QPPV) contacts) immediately.
- All efforts must be done to obtain a hepatologist consultation.
- In addition to the hepatitis panel, obtain samples for:
    - quantitative hepatitis B DNA and hepatitis delta antibody (if positive for hepatitis B surface antigen at screening),
    - serum acetaminophen adduct HPLC assay,
    - antinuclear antibody, anti-smooth muscle antibody, Type 1 anti-liver kidney microsomal antibodies and quantitative IgG or gamma-globulin.
- Follow the instructions below for all events meeting any definition of liver biochemistry event of special concern.

Instructions in the event of any of the following: (i) a case of possible Hy's Law, or (ii) meeting definition of a biochemistry event of special interest

- Report the AE to the Sponsor, and as an SAE if the event is a possible Hy's law, or meets SAE criteria (via the study Medical Monitor and Shin Poong QPPV)
- Collect if not scheduled urine, blood, or relevant biological fluids for additional diagnostic tests
- Follow up of liver chemistries albumin and prothrombin time (+ prealbumin, serum ceruloplasmin, procollagen III peptide, α-1-antitrypsin and α-feto protein when possible) twice weekly or more frequently if considered medically indicated until values normalize or substantively improve.
- If medically indicated request liver imaging (ultrasound, computerized tomography or magnetic resonance imaging)
- If liver biopsy obtained, request full report be forwarded to the Sponsor
- Obtain additional consent for above if necessary
- Obtain if needed additional history and review of medical records
- Ask again for details, in particular any concomitant meds, OTC meds, herbal remedies, prior exposure, previous episodes etc and discuss them with Sponsor
- Obtain documentation for all protocol- required or non-protocol required laboratory values, ECG tracings, pathology reports until resolution
- Ensure appropriate medical attention, including move to hospital Emergency Department or ICU, specialist consultation, Liver Transplant Unit etc as deemed medically necessary
- Arrange appropriate follow up for investigations
- Sponsor, Investigator and Hepatology Consultant to review aggregate data from available sources for underlying trends
- Contact the Sponsor safety department (via Medical Monitor) or other internal and external experts for advice as needed

Documentation needed in the event of an elevation of special concern in one or more liver function tests

If laboratory values are returned that meet the above-described criteria of Serious Liver Reactions, the investigator should attempt to document the following in the investigator comment log of the CRF prior to contacting the Sponsor:

- The subject's age, gender and weight.
- The date (and visit number) on which the blood sample was obtained.
- The Randomization date as well as the dose level that the subject is currently taking, and the duration of exposure to that dose level. The exact date on which the subject took the last dose

of the study medication and an objective assessment should be obtained of the subject's compliance with the study medication.

- The specific abnormal laboratory values, as well as those of each of the other LFTs noted above (regardless of value) and, when relevant, results of isoenzyme or fractionation analyses. The investigator should also document the corresponding laboratory values at screening/baseline.
- Other notable abnormalities in laboratory values (*e.g.,* complete blood count, eosinophilia, or electrolyte abnormalities, Serum transferrin saturation and serum ferritin if present).
- The dates and nature of any relevant adverse events (*e.g.,* jaundice) that occurred since Randomization, with particular attention to hypotension, fever, rash, hepatitis symptoms (e.g. appearance or worsening of fatigue, nausea, anorexia, nausea, emesis, abdominal pain), or other adverse events that might have occurred in close proximity to the elevation in the laboratory value(s) of interest.
- Any associated physical findings (including results of any exams or evaluations, including heart rate, blood pressure, temperature, and abdominal exam).
- The use of concomitant medications (*e.g.,* ARVs, paracetamol/acetaminophen, herbal products) since randomization, as well as the dates of exposure to the concomitant medication(s). Please include any nutritional supplements, vitamins and/or herbal preparations that the subject might have taken during this time frame.
- A statement concerning whether the subject has consumed alcohol since the time of Randomization, with a description of frequency and intensity, if relevant. A blood alcohol level should be obtained if the subject's history and/or clinical presentation suggest proximal use or intoxication with alcohol.
- Any history on the subject's part of prior elevations in any of the relevant laboratory values. Provide actual dates and laboratory values.
- Any history on the subject's part of a past or recent history of exposure to known factors that can cause, or are associated with, elevations in liver function tests. Examples of these factors include alcohol abuse and/or dependence, hepatitis (infectious or chemical), infectious mononucleosis, gallbladder disease, liver disease of any kind, jaundice, myocardial infarction, heart failure and/or episodes of hypotension.
- Any family history of hepatitis (from any cause) or hepatotoxicity from medications.

The investigator is asked to contact the Medical Monitor if there are any questions about the most appropriate course of action, and/or if there are questions as to whether the subject should be referred to a specialist for further evaluation.

It is anticipated that the investigator will follow any subject with clinically significant elevations of one or more liver function tests until there is clear evidence that the value(s) have stabilized and/or normalized. In addition, an explanation should be provided for any subjects that are lost to follow up.

POST EMA-POSITIVE OPINION

PRODUCT: Pyramax
(pyronaridine-artesunate)

PHASE IIIB/IV COHORT EVENT MONITORING STUDY TO EVALUATE, IN REAL LIFE SETTING, THE SAFETY AND TOLERABILITY IN MALARIA PATIENTS OF THE FIXED-DOSE ARTEMISININ-BASED COMBINATION THERAPY PYRAMAX (PYRONARIDINE-ARTESUNATE)

STUDY NUMBER: SP-C-021-15

STUDY NAME: Phase IIIb/IV Safety Assessment of Pyramax

FINAL VERSION 9.0 DATE: 15 March 2019

CONFIDENTIAL

NAMES AND ADDRESSES:
Principal Investigators:

Cameroon: Dr Jude D. Bigoga , PhD
[redacted]

Ivory Coast: Dr. Serge-Brice Assi, MD, PhD
[redacted]

Congo: Dr Felix Koukouikila- Koussounda, PhD
[redacted]

Democratic Republic of Congo: Prof Gaston Tona Lutete
[redacted]

Gabon: Dr Ghyslain Mombo-Ngoma, MD
[redacted]

Other Responsible Parties:

Clinical monitoring
Family Health International Inc
[redacted]

Central Laboratory (Viral Hepatitis Assessment and Full Hepatitis Panel)
Covance Central Laboratory Services SA
[redacted]

Central Laboratory (Re-appearing parasitemia)
Swiss Tropical and Public Health Institute
[redacted]

Data Management and Statistical Analyses
ICON plc
[redacted]

Electronic Trial Master File
PhlexGlobal
[redacted]

Version 9.0 Date 15/03/2019: Property of SHIN POONG/MMV- strictly confidential

**COORDINATING PRINCIPAL INVESTIGATOR**	Name :<br>Address:<br><br><br><br>Mob:<br>Tel:<br>E-mail:	Prof. Dr Michael Ramharter<br>[redacted]
**PROJECT COORDINATOR**	Name:<br>Address:<br><br><br>Tel:<br>E-mail:	Prof. Francine NTOUMI, PhD<br>[redacted]
**SPONSOR**<br><br>**SHIN POONG**	Name:<br>Address:<br><br>Mob:<br>Tel:<br>Fax:<br>E-mail:	Mr Jangsik Shin<br>[redacted]
**FUNDER**<br><br>**MMV**	Name:<br><br>Address:<br><br>Mob:<br>Tel:<br>Fax:<br>E-mail:	Dr Stephan Duparc<br>[redacted]
**MEDICAL MONITOR**	Name:<br>Address:<br>Mob:<br>Tel:<br>E-mail:	Selidji Todagbe Agnandji<br>[redacted]

[redacted]

STUDY PROTOCOL AGREEMENT FORM

I, undersigned, ,………………………………………………………………, hereby certify that I have examined the protocol of the above referenced study for SHIN POONG and that I have thoroughly discussed the objectives of this study as well as the contents of this protocol with the Sponsor's representatives.

I agree to keep the contents of this protocol confidential and not to disclose it to a third party and to use it only for the purposes of this study.

I agree to perform this study according to this protocol and to meet the objectives, to comply with the ethical rules and to ensure patient safety.

I have well understood that in case the Sponsor may decide to prematurely end or to suspend this study at any time and for any reason, I would be informed of this decision in writing. Conversely, in case I would decide to prematurely end or to suspend this study, I commit to immediately inform the Sponsor of this decision in writing.

INVESTIGATOR FOR (Country):

NAME:

DATE:

Signature: ________________________________

TABLE OF CONTENTS

1. LIST OF ABBREVIATIONS....................10

2. SUMMARY....................12

STUDY FLOW CHART:....................18

3. INTRODUCTION AND RATIONALE....................19
3.1 Summary of current situation....................19
3.2 Pyramax (pyronaridine-artesunate)....................19
3.3 Malaria in Cameroon, Republic of Congo, DRC, Gabon and Ivory Coast....................20
3.4 Project rationale....................23
3.5 Proposed Sites participating in the study....................24

4. STUDY OBJECTIVES....................24
4.1 Primary objective....................24
4.2 Secondary objectives....................24

5. DESCRIPTION OF THE STUDY....................25
5.1 Description of the study design....................25
5.2 Study duration....................26
5.3 Drug prescription and dosing....................26
5.4 Evaluation criteria....................27

6. STUDY POPULATION AND PATIENT SELECTION....................28
6.1 Sample size....................28
6.2 Selection criteria....................28
6.2.1 Inclusion criteria....................28
6.2.2 Exclusion criteria....................29
6.3 Enrolment procedure....................29
6.3.1 Investigational Sites....................29
6.3.2 Patient selection....................29
6.3.3 Patient numbering....................30

7. STUDY PROCEDURE AND DATA COLLECTION....................30
7.1 Visit planning....................30
7.2 Collected data....................30
7.2.1 Registration at the clinical study centre....................30
7.2.2 Examination and data collection at inclusion....................31
7.2.3 Follow-up visits/contacts and clinical tolerability follow-up questionnaire....................31
7.3 Patients who did not receive follow-up visits/contacts or who have prematurely discontinued treatment....................33
7.3.1 Premature treatment discontinuation....................33
7.3.2 Patient lost to follow-up....................33
7.4 Thick blood smear and blood spot logistics....................34
7.5 Hepatitis panel logistics:....................34
7.6 Vital signs and Physical examination....................35

8. SUBJECT SAFETY....................35

8.1 Training....35
8.2 Monitoring of adverse events....35
8.3 Definition of Adverse Event and Serious Adverse Event....36
8.4 Obligation of AE notification....36
8.5 Adverse event of special interest....38
8.6 Pregnancy....39
8.7 Sponsor obligations....40
8.7.1 Safety....40
8.7.2 Quality Assurance....40

9. STATISTICAL METHODS....41
9.1 Data analysis....41
9.2 Determination of sample size....41

10. DATA MANAGEMENT....42
10.1 Collection and validation of data....42
10.2 Quality control of data on site....42
10.3 Quality control of the preparation of thick blood smear slides....42
10.4 Quality control of thick blood smear sample reading....43
10.5 Data entry....43
10.6 Data cleaning and data base locking....43

11. TASKS AND RESPONSIBILITIES....43
11.1 Study specific DSMB....43
11.1.1 Responsibilities of the study specific DSMB....43
11.2 Responsibilities of the clinical staff of the health facilities....44
11.3 Responsibilities of the Sponsor....45

12. ETHICAL AND REGULATION ASPECTS....45
12.1 Regulations....45
12.2 Informed consent....46
12.3 Expected risks....46
12.4 Data protection and confidentiality....46
12.5 Insurance....47
12.6 Premature termination of the study....47
12.7 Competent authority inspections....47

13. PROTOCOL AMENDMENTS....48

14. DOCUMENTATION AND UTILISATION OF STUDY RESULTS....48
14.1 Properties and use of the study data and results....48
14.2 Publications....48

15. REFERENCES....49

16. ANNEXES....52
ANNEX 1....52
Declaration of Helsinki....52
ANNEX 2....57
Definition of Severe Malaria....57

WORLD HEALTH ORGANISATION CRITERIA 2014 57
ANNEX 3 57
Guidance for the evaluation of the intensity of clinical signs 57
ANNEX 4 61
Adverse Events of Special Interests: Drugs and the Liver 61

1. LIST OF ABBREVIATIONS

ACT	Artemisinin-based Combination Therapy
AE	Adverse Event
AESI	Adverse Event of Special Interest
AL	Artemether-lumefantrine
ALT	Alanine aminotransferase
An	*Anopheles*
AS	Artesunate
ASAQ	Artesunate-amodiaquine
AST	Aspartate aminotransferase
CANTAM	Central African Network on Tuberculosis, AIDS and Malaria
CERMEL	Centre de Recherches Médicales de Lambaréné
CHW	Community Health Worker
CMV	Cytomegalovirus
CNPV	National Pharmacovigilance Center of DRC
CPK	Creatinine phosphokinase
CRF	Case Report Form
DILI	Drug Induced Liver Injury
DOT	Direct Observational Treatment
DRC	Democratic Republic of Congo
eCRF	Electronic Case Report Form
EBV	Epstein Barr Virus
EMA	European Medicines Agency
HAV	Hepatitis A Virus
Hb	Haemoglobin
HBc	Hepatitis B core antigen
HBsAg	Hepatitis B surface Antigen
HCV	Hepatitis C Virus
HIV	Human immunodeficiency virus
IgM	Immunoglobulin M
IPTp	Intermittent Preventive Treatment in pregnancy
ITNs/LLINs	Insecticide Treated Nets / Long Lasting Insecticidal Nets
IRS	Indoor Residual Spraying
LDH	Lactate Dehydrogenase
LFTs	Liver function tests
MedDRA	Medical dictionary for Regulatory Activities
NMCP	National Malaria Control Programme
PCR	Polymerase chain reaction
P. falciparum	*Plasmodium falciparum*
PI	Principal Investigator
QPPV	(*Pyramax*) Qualified Person for Pharmacovigilance
RDT	Rapid Diagnostic Test
RNA	Ribonucleic acid
SAE	Serious Adverse Event
SDV	Source Data Verification
SmPC	Summary of Product Characteristics
SP	Sulfadoxine-pyrimethamine

Version 9.0 Date 15/03/2019: Property of SHIN POONG/MMV- strictly confidential

SPPV	Shin Poong Pharmacovigilance
STM	Study Team Member
SUSAR	Suspected Unexpected Serious Adverse Reaction
ULN	Upper Limit of Normal
UPC-PV	Unité de Pharmacologie Clinique et Pharmacovigilance
WHO	World Health Organisation

2. SUMMARY

PRODUCT: Pyramax **Study N°: SP/CANTAM-C-021-15**

**Title**	Phase IIIb/IV Cohort Event Monitoring study to evaluate, in real life setting, the safety and tolerability in malaria patients of the fixed-dose Artemisinin-based Combination Therapy Pyramax (pyronaridine-artesunate).
**Location of the study**	The study will be performed in public health facilities of the CANTAM network in Central Africa and in public health facilities in West Africa where *Pyramax* will be used as treatment of uncomplicated malaria episodes, including repeat episodes.
**Objectives**	The main objectives of the study are to assess the safety of *Pyramax* particularly in patients with underlying liver function abnormalities, in patients who have co-morbid conditions, such as HIV, and also in very small children (<1 year of age)<br><br>**Primary**:<br>Evaluation and identification of the hepatic safety events of *Pyramax* in a sub group of patients enrolled with LFTs >2xULN from blood taken immediately prior to treatment without any clinical signs or symptoms of hepatotoxicity and with signs and symptoms of uncomplicated malaria confirmed by a Rapid Diagnostic Test (RDT) or microscopy (thick blood smear).<br><br>**Main Secondary:**<br>Comparison of the clinical hepatic safety of *Pyramax* between a cohort of patients enrolled with LFTs >2xULN and a cohort of patients enrolled with normal LFTs matched for demographic characteristics.<br><br>**Other Secondary**:<br>- Evaluation of the safety and tolerability in patients with normal and abnormal LFTs at inclusion according to any possible hepatic underlying disease based on the finding of the hepatitis panel and according to their HIV status (where known), their nutritional status, their age (children < 1 year of age in comparison to the rest of the treated population) and their weight.<br>- Evaluation of the safety and tolerability in retreated patients with a special focus on the hepatic safety.<br>- Evaluation of the potential for hypersensitivity reactions<br>- Evaluation of the relationship between the occurrence of hepatic related adverse events with or without LFT abnormalities and the administration of concomitant medications (in particular paracetamol, herbal medicines and antiretroviral drug).<br>- Evaluation of the efficacy and compliance of *Pyramax* when used under usual conditions (including unsupervised medication intake) in patients with signs and symptoms of uncomplicated malaria confirmed by a RDT or microscopy (thick blood smear).
**Design and duration of the study**	This is a non-comparative Cohort Event Monitoring study. All the patients for whom a diagnosis of uncomplicated malaria (according to the WHO criteria) is suspected, will undergo an RDT/microscopy. If the presence of malaria is confirmed and the patient (or parent/ guardian in case of children) provides his/her informed consent, he/she will be assigned a unique ID number and will be enrolled in the study. Blood will be taken for blood spots for PCR analysis, for retrospective LFT assessment as well as for a retrospective viral hepatitis assessment to be done

	only in patients with abnormal LFTs. A blood sample will be taken for haematology (Hb mandatory). The first dose of *Pyramax* will be given under Direct Observational Treatment (DOT) conditions, but patients will take their second and third doses of *Pyramax* under usual conditions (including unsupervised medication intake). Patients will be also required to attend the health care facilities if they have any clinical signs or symptoms of hepatotoxicity.<br><br>A cohort of approximately 2% of children who are <1 year of age will also be included for monitoring of liver function.<br><br>The patients will be visited at home at Day 7 ±1 day, by a community health worker (CHW), in order to capture the adverse events (AEs) experienced. In the case of signs or symptoms of hepatotoxicity or hepatic related AEs, the patients will be referred to the health facilities and blood will be taken on this occasion for LFT assessment, haematology (Hb mandatory), and where appropriate for full hepatitis panel assessment. If a patient cannot be directly reached, the CHW must organize an appointment with this patient before Day 10. Special procedures will be followed in case of serious adverse events (SAEs) and/or severe adverse events and events classified as being of special interest (AESI) (see specific section).<br><br>At Day 28 ±2 days, all patients will be seen by a CHW for a final assessment, including blood taken for thick blood smears and spots. In the case of signs or symptoms of hepatotoxicity or hepatic related AEs occurring since the previous Day 7 visit, the patient will be referred to the health facilities and blood will be taken for LFT assessment, haematology (Hb mandatory), and where appropriate for full hepatitis panel assessment.<br><br>Female patients must communicate to their village CHW or the study team if they get pregnant just before or after the start of the *Pyramax* treatment and for 2 months post-treatment. All pregnancies must be documented on the manufacturer's *Pyramax* Pregnancy Register. In these cases, the evolution of the pregnancy will be monitored with visits at 3, 6 and 9 months and at 7 days after the delivery. Information on the drugs taken during the pregnancy as well as AEs/SAEs/AESIs and the health status of the newborn(s) will be collected. On the last day of follow-up (usually Day 28 ±2 or in case of early termination, on the respective day), a urine pregnancy test will be performed for all women of child-bearing potential, defined as women who have experienced menarche and who are not permanently sterile or postmenopausal (defined as 12 consecutive months with no menses without an alternative medical cause). The investigator shall decide whether this is applicable or not based on the medical history of the patient, and on customs and practices of the region.
**Drug prescription and dosing**	Patients will be instructed to take one *Pyramax* dose every 24 hours over a period of three days, i.e. at Day 0, then after 24 hours (Day 1) and after 48 hours (Day 2) from the first administration.<br><br>The dose will be based on patient body weight, as specified in the summary of product characteristics and in the Investigator's Brochure. Two strengths and formulations of *Pyramax* will be provided to facilitate the dosing in adults, children, and young infants:<br>• Tablets of 180:60 mg of pyronaridine and artesunate (patients ≥20kg),<br>• Granules in sachets of 60:20 mg of pyronaridine and artesunate (patients 5-<20kg).
**Population**<br>Selection criteria	**Inclusion Criteria**<br>• Uncomplicated malaria (*Plasmodia* of any species) diagnosed as per national policies and in line with WHO recommendations:

	○ Fever or history of fever in the previous 24 h and/or the presence of anaemia, for which pallor of the palms appears to be the most reliable sign in young children.<br>○ Confirmation of malaria by a parasitological diagnosis (RDT or Microscopy (thick blood smear) analysis).<br>• Weight ≥5 kg - < 20 kg (granules); ≥20 kg (tablets).<br>• Ability to take an oral medication.<br>• Ability and willingness to participate based on signed informed consent (a parent or a guardian has to sign for children below 18 years old) and on signed assent form for minors that could be required per national regulations in each participating country.<br>• The patient has to comply with all scheduled follow-up visits.<br><br>**Exclusion Criteria**<br>• Patients with clinical signs or symptoms of hepatic injury (such as nausea, abdominal pain associated with jaundice) or known severe liver disease (i.e. decompensated cirrhosis, Child-Pugh stage 3 or 4).<br>• Known allergy to artemisinin and/or to pyronaridine.<br>• Known pregnancy.<br>• Lactating women should be excluded if other anti-malarial treatments are available.<br>• Complicated malaria as per WHO definition (Annex 2)<br>• Patients that the investigator considers would be at particular risk if receiving an anti-malarial or if participating in the study.<br>• Patients having been treated with *Pyramax* in the previous 28 days.
Number of patients	Based on the primary objective, 120 malaria episodes in patients with baseline raised AST/ALT value >2xULN will be needed for a 81.6% probability to observe one severe hepatic event in this population. Given the screening rate in previous studies of 1.4% for malaria patients with AST/ALT >2xULN, recruitment will require an estimate of at least 8,572 malaria episodes. To mimic reality, patients can be included more than once in the study.
Number of sites	Public health facilities in four medical research centres with adjacent satellite sites of the CANTAM network in Central Africa and one centre in Ivory Coast. Additional sites may be added by consent of Coordinating PI, Sponsor and MMV.
**Study Flow Chart**	Reported below.
**Evaluation criteria**	**Primary:**<br>A safety analysis will be performed retrospectively on a sub-group of patients identified with abnormal baseline LFTs. In this sub-population, clinical hepatic safety will be assessed through the specific analysis of hepatic adverse events captured by the CHW at Day 7 ± 1 day or at any unforeseen visits in case of signs or symptoms of hepatotoxicity or hepatic related AEs, after confirmation by biochemical (LFT) assessment.<br><br>**Main Secondary:**<br>• Comparative analysis of the clinical hepatic safety (signs or symptoms of hepatotoxicity or hepatic related AEs, confirmed biologically) will be made between patients having abnormal LFTs (AST/ALT >2xULN) at enrolment versus a cohort of patients with normal LFTs matched for demographic characteristics.

	<u>**Other Secondary**</u>:<br>• A safety analysis will be performed in patients with normal and abnormal LFTs at inclusion according to any possible hepatic underlying disease based on the finding of the hepatitis panel and according to their HIV status (where known), their nutritional status, their age (children < 1 year of age in comparison to the rest of the treated population) and their weight.<br>• Safety (including hepatic safety) in retreated patients.<br>• Incidence of hypersensitivity reactions<br>o At any unforeseen visits in case of signs or symptoms of hepatotoxicity or hepatic related AEs, special efforts will be made to assure and record adequate follow-up of serious and/or severe adverse events and of the adverse events of special interest (see specific section).<br>o Biological/LFTs assessment (AST/ALT bilirubin total and conjugated, and alkaline phosphatases). Assessment of baseline LFT results where signs or symptoms of hepatotoxicity or hepatic related AEs were reported at Day 28 or at any unforeseen visit.<br>• The relationship between the occurrence of hepatic related adverse events with or without LFT abnormalities and the administration of concomitant medications (in particular paracetamol, herbal medicines and antiretroviral drugs) will be evaluated.<br>• Crude Day 28 cure rate by species and PCR-adjusted cure rate for Day 28 cure rate for *P. falciparum*<br>• Time between malaria episodes and frequency of repeat episodes before and after Day 28.<br>• Compliance will be assessed by counting the number of tablets or sachets of granules dispensed but not taken, when the CHW visits the patient at home.
**AEs of special interest (AESIs)**	In case of an AESI confirmed by the study physician, the Sponsor and *Pyramax* QPPV shall be informed within 24 hours ([redacted] even if the event does not satisfy any condition of seriousness. Notification will occur through the use of an AESI form.<br><br>**1) Hepatic** AESIs should be reported as follows:<br><br>**a) Patients with normal LFTs at baseline:**<br>- Present with fatigue, nausea, abdominal pain, itching or signs of jaundice such as:<br>o dark urine,<br>o putty or mastic coloured stools,<br>o jaundice (yellowing of the whites of the eyes or skin),<br>and<br>- ALT or AST >3 x ULN<br><br>**b) Patients with baseline ALT/AST >2xULN**<br>- Present with fatigue, nausea, abdominal pain itching or signs of jaundice such as:<br>o dark urine,<br>o putty or mastic coloured stools,<br>o jaundice (yellowing of the whites of the eyes or skin),<br>and<br>- ALT/AST >2 x baseline value

	In all these cases, patients shall be directed to the designated health facilities for evaluation and recording of all relevant information. If necessary, the health worker will contact the study team member or PI / co-PI.<br><br>In addition to the LFT assessment at baseline for retrospective assessment, LFTs will be performed as soon as the patient arrives at the health facilities at any unforeseen visit and at Day 28+/2, if required because of clinical signs or symptoms of possible hepatotoxicity or hepatic related AEs.<br><br>In the event of biological signs of hepatotoxicity associated with clinical symptoms/suspected Drug Induced Liver Injury (DILI), blood will need to be taken/stored to perform the following additional tests (full hepatitis panel):<br><br>• Hepatitis A, B, C (Anti-HAV IgM, Anti-HBc IgM, HBsAg, and hepatitis C RNA),<br>• Hepatitis E IgM antibody,<br>• Cytomegalovirus (CMV) testing polymerase chain reaction (PCR) testing,<br>• pp65 antigen, or IgM antibody,<br>• Epstein Barr virus (EBV) viral capsid antigen IgM antibody,<br>• Serum creatinine phosphokinase (CPK) and lactate dehydrogenase (LDH).<br>• Serum transferrin saturation and serum ferritin (diagnosis of hemochromatosis).<br>• Liver's biosynthetic capacity: albumin and prothrombin time (+ prealbumin, serum ceruloplasmin, procollagen III peptide, α-1-antitrypsin and α-feto protein when possible).<br><br>**2) Hypersensitivity** AESIs should be reported if patients show signs of hypersensitivity soon after treatment with *Pyramax* (particularly on repeat treatment), such as:<br>- Flushing<br>- The appearance of wheals / urticaria<br>- Breathlessness<br>- Faintness and/or fall in blood pressure
**Sample size**	Based on the primary objective, 120 malaria episodes in patients with baseline AST or ALT value >2xULN will be needed for a 81.6% probability to observe one severe hepatic event in this population, defined as:<br><br>• Appearance of clinical signs and symptoms of possible hepatotoxicity associated with a rise in ALT/AST >2 x baseline value.<br><br>Given the screening rate in previous studies of 1.4% for malaria patients with baseline AST/ALT > 2 x ULN, recruitment will require an estimate of at least 8,572 malaria episodes. To mimic reality, patients can be included more than once in the study.
**Statistical analysis**	The statistical analyses will be performed using an appropriate software package such as SAS , version 8.2 or later versions, SAS Institute, NC, Cary, USA.<br><br>A detailed statistical analysis plan will be generated within three months of study start. This plan may be revised during the course of the study in order to take into account protocol amendments, if any, and to address potential issues occurring during the study, that could affect the planned analyses. A final, approved, statistical analysis plan will be in place before the database lock.<br><br>**Analysis (All patients)**

	The statistical analysis will be mainly descriptive. The estimates of the incidence of adverse events will be based on crude rates. All estimates will be complemented with appropriate 95% confidence intervals.<br><br>Adverse events will be coded in accordance with standard medical dictionary terminology (MedDRA) before database lock.<br><br>Incidences will be computed on the following classes of AEs:<br>▪ All AEs<br>▪ Serious AEs<br>▪ AEs of special interest (AESIs)<br>▪ AEs which caused early discontinuation of *Pyramax*<br>▪ AEs related to *Pyramax* treatment<br><br>Specific analyses will be carried-out on the AESIs. Correlation analyses will be carried-out in order to verify if their occurrence is more frequent with the previous/concomitant use of other drugs (specific classes to be determined in the statistical analysis plan), including antimalarials, in the presence of a co-morbidity (specific classes to be determined in the statistical analysis plan), or in the presence of parasitemia.<br><br>Compliance will be computed for each patient as the ratio between the number of tablets or sachets actually taken and the number of tablets or sachets that the patient should have taken. Two groups of patients shall be described: compliant = 100% treatment compliance and non-compliant = other cases. The safety profile of compliant vs non-compliant patients will be compared.
**Safety reporting**	Safety data as well as any pregnancies will be reported to the Manufacturer and Sponsor Shin Poong ([redacted] and will be managed as per national guidelines. In addition safety data will be reported by the Sponsor to the WHO collaborating centre for pharmacovigilance through the National Pharmacovigilance Center of DRC (CNPV).
**Ethical issues**	The protocol will be conducted in compliance with the Declaration of Helsinki, and the directives in the respective countries, in particular concerning the submission to the Ethics Committees and the protection of personal data.
**Dates**	Trial duration: Estimated to be approximately 18 to 24 months after study start, depending on enrolment being completed over 1 or 2 malaria seasons. However, the study will not be terminated until at least 120 malaria episodes in patients with baseline LFTs >2xULN are enrolled.

Version 9.0 Date 15/03/2019: Property of SHIN POONG/MMV- strictly confidential

STUDY FLOW CHART:

**STUDY EXAMINATION**	Screening/ Inclusion Day 0/**V1** (health centre)	Day 7+/- 1 (home visit)	Day 28 +/-2 (home visit)	**Unforeseen Visit**
Age / sex / weight / height	X			
Vital signs (pulse, blood pressure)	X			
Body Temperature	X	X	X	X
Malaria symptoms / Hepatic symptoms / Other symptoms	X	X	X	X
Rapid Diagnostic Test (RDT)* or thick blood smear	X		X	X
Informed Consent	X			
Medical history	X			
Physical examination	X			
Previous medications (last month)	X			
Blood spots for PCR	X		X	X
Blood sample taken for AST/ALT, Total Bilirubin+/- Conjugated Bilirubin, Alkaline phosphatase and haematology (haemoglobin mandatory)	X[¶]	X(#)	X(#)	X(#)
Viral hepatitis assessment (hepatitis A, B, C, delta (if hepatitis B positive) and E)	X[a]			
Full hepatitis panel		X(#)	X(#)	X(#)
Inclusion/Exclusion	X			
Prescription of *Pyramax*	X			
AE recording	X (only SAEs)	X	X	X
Concomitant medications	X	X	X	X
Compliance assessment		X		
Reported Pregnancy	X	X	X	X
Urine pregnancy test			X(∞)	X(∞)

*RDT only possible at D0, thick blood smear mandatory at D28.

¶ LFT samples will be only analyzed retrospectively

[a] Blood for viral hepatitis assessment will be drawn from all patients at baseline. For patients retrospectively found with baseline AST or ALT >2xULN, the blood for viral hepatitis assessment will be sent to the central laboratory for analysis, while for all other patients (i.e. with AST and ALT $\leq$2xULN) the blood for viral hepatitis assessment will be destroyed.

In the case of clinical signs of possible hepatotoxicity/DILI, LFTs & Hb assessment will be performed as soon as the patient arrives at the health centre and blood for a full hepatitis panel will be taken in case of AESI or SAE related to hepatotoxicity.

∞ A urine pregnancy test will be performed for all women of child-bearing potential, on the last day of follow-up (usually Day 28 ±2 or in case of early termination, on the respective day), at home or at the health facility, if the patient is transferred to the health facility.

Women of child-bearing potential are defined as women who have experienced menarche and who are not permanently sterile or postmenopausal (defined as 12 consecutive months with no menses without an alternative medical cause). The investigator shall decide whether this is applicable or not based on the medical history of the patient, and on customs and practices of the region.

3. INTRODUCTION AND RATIONALE

3.1 Summary of current situation

In response to the breakout and the spread of *Plasmodium* strains resistant to classical anti-malarials, the WHO recommends the use of artemisinin-based combination therapies (ACTs) in the treatment of uncomplicated malaria episodes.

New ACTs are entering the African market and will be used by the public health care system. Until now, these ACTs were prescribed to a limited number of patients and most of the time within strictly regulated clinical studies with patients meeting restrictive selection criteria. The collection of safety data and the risk evaluation based upon observational data are critical in order to evaluate the risk/benefit profile of a product through its life cycle and to inform the best use of medicines. In addition, the impact of the introduction of a particular ACT into the public health care system on the evolution of its efficacy and on the malaria morbidity and mortality is unknown and is worth investigating (1, 2, 3, 4, 5).

3.2 Pyramax (pyronaridine-artesunate)

Pyronaridine and artesunate are antimalarial agents with a history of clinical use, and artesunate has been used clinically in combination with other drugs. The action of artesunate is a rapid knock-down of the parasites, after which, the drug is quickly cleared as it has a short systemic half-life. Pyronaridine is also effective in the short-term but has an intermediate blood half-life thus providing a more sustained schizonticidal effect.

Artesunate, has been partnered with the established antimalarial agent pyronaridine to develop a three-day oral therapy *Pyramax*, for use in infants, children and adults to treat acute, uncomplicated malaria. The aim of the fixed dose combination of pyronaridine and artesunate is to provide a rapid reduction in parasitaemia with a short term regimen, thereby improving compliance and reducing the risk of recrudescence through the slower elimination of pyronaridine. This fixed combination of pyronaridine and artesunate has been studied extensively in both healthy volunteers and uncomplicated malaria patients and has EMA Positive Opinion under the article 58 procedure.

The safety database for the *Pyramax* (PA) clinical programme includes 4121 subjects who received at least 1 dose, administered either as the fixed-dose co-formulation or as pyronaridine tetraphosphate (PP) + artesunate (AS), or pyronaridine alone (Phase I), across four Phase I, two Phase II, five Phase III studies and a longitudinal, repeat dose Phase IIIb study. This safety database includes the subgroup of children treated with *Pyramax* granules in three studies, where the resulting safety profiles were comparable and consistent for both adult and paediatric formulations.

Pyramax treatment was generally well tolerated with the vast majority of adverse events being of mild or moderate intensity, with headache and gastrointestinal symptoms occurring most frequently.

The only notable safety finding more prevalent in subjects treated with *Pyramax* is associated with significant transient liver transaminase elevations in a small minority of subjects. The early onset (Day 3-7) and rapid resolution of the liver transaminase elevations are consistent with a direct, low level toxicity seen. In the patient studies, the potential risk of liver injury, associated

with this finding, was assessed taking into account subjects who had increases >3x ULN (3.4%), >5xULN (1.4%), and >10x ULN (0.4%) for transaminases, as well as those subjects who might qualify as Hy's law candidates (ALT >3xULN and total bilirubin >2xULN). Review of the data by an independent safety review board concluded that the risk of progressive liver injury, especially for a 3-day course of treatment, was very low. This was supported further by the fact that serious idiosyncratic hepatotoxicity typically begins weeks or months after starting therapy. Of note, all the raised values observed with *Pyramax* treatment returned to normal, the vast majority being normal at Day 28 or earlier (the precise timing confounded by timing of the visit dates). Overall, changes in liver function tests were mild with a small number of moderate cases (based on peak total bilirubin levels) as per the criteria of the Drug Induced Liver Injury Network. Furthermore there were no cases of liver failure, no encephalopathy, no evidence of coagulopathy and no evidence of a delayed effect.

Repeat dosing with *Pyramax* in Thai subjects with a single administration repeated after washout of 8 weeks showed no rises in transaminases after first or second dosing. Interim data from a longitudinal patient study conducted in West Africa where patients are retreated with *Pyramax* (tablets and granules) or comparator as many times as required over a 2-year period has shown that in the first 316 patients retreated at least once there is no increased risk in terms of liver function or adverse events on repeat dosing with periods between treatments of 27 and 307 days.

The Investigators should be familiar with the *Pyramax* Investigator Brochure (Version 8, 2016), in which the clinical and non-clinical studies are further described (6, 7, 8, 9, 10, 11, 12, 13, 14, 15, 16, 17).

3.2.1. Risk/benefit

Pyramax is a new option for the treatment of acute uncomplicated *P. falciparum* and *P. vivax* malaria, with a simple, once-a-day for three days regimen, and with no food restriction. The cumulative safety of pyronaridine-artesunate has been evaluated in over 4500 subjects and has shown that treatment was generally well tolerated. In order to get further assurance on safety, patients previously excluded or poorly represented in the Phase II/III clinical studies program will be included in the present cohort event monitoring study, such as patients with significantly raised liver enzymes, before treatment as well as patients with co-morbidities (e.g. HIV, hepatitis, or severe malnutrition).

For a full description of potential risks associated with *Pyramax* treatment, please refer to the EMA-approved Summary of Product Characteristics for *Pyramax* Tablets and *Pyramax* Granules (in particular to Sections 4.4 Special warnings and precautions for use, 4.6 Fertility, pregnancy and lactation and 4.8 Undesirable effects).

3.3 Malaria in Cameroon, Republic of Congo, DRC, Gabon and Ivory Coast

Cameroon

- **Yaoundé**

Malaria is endemic throughout Cameroon where it is responsible for 30% morbidity, 29% hospital consultations, 38% of cases among gravid women, 49% hospitalization and 45% deaths

among children under five. Mfou, a forest area located in the Mefou and Afamba Division, Centre region of Cameroon (4°27'N and 11°38'E) has about 85 000 inhabitants, most of them living below the poverty line. The climate is equatorial with four seasons comprising two rainy seasons (March-June; August-October) and two dry seasons (November-February; June-July), which are of unequal durations (long and short season respectively). It has an annual average temperature of 22-25°C with rainfall averaging 1700-2500 mm/year. Houses are poorly constructed and close to bushes, increasing anopheles-human contact. Malaria is holoendemic in this locality and transmission is perennial, peaks generally during the rainy season and the rainy-dry transition period with about 25 infectious bites/year. The prevalence of malaria in the population is 77% and 40.6% among school aged children. *Plasmodium falciparum* is predominant parasite species responsible for over 97% all infections. The major vectors are *Anopheles gambiae* and *Anopheles funestus*.

- **Study sites**

The Principal site and Satellite site are overseen by the Principal Investigator located at University of Yaoundé.
The satellite site is based at Mfou District Hospital.

Nkolbisson is a region of Yaoundé and is the site location for the University of Yaoundé. It has four seasons made up of two rainy seasons (March-June; August-October) and two dry seasons (November-February; June-July). These seasons are of unequal durations (long and short season respectively). The annual average temperature is 25°C. With a population of about 200 000 inhabitants, a density of 200 inhabitants per km^2, Nkolbisson is composed of almost all the ethnic groups of the country. The rainfall varies between 1500 mm and 2000 mm per year. Just like Mfou climatically, malaria is holoendemic with a perennial mode of transmission that peaks generally during the rainy season and the rainy-dry transition period. Malaria is main cause of consultation with huge burden among children under 5 years old (86.5%). *P. falciparum* is predominant malaria parasite species accounting for almost all the malaria cases. This parasite is responsible for the severe forms of malaria with *An. gambiae* being the predominant vector.

<u>Republic of Congo</u>

- **Study sites**

The Principal site and Referring sites are overseen by the Principal Investigator who is located at Centre de Santé FCRM Massisia. Referring sites are Centre de Santé de Madibou, Brazzaville and Hôpital de Base de Makélékélé, Brazzaville.

The study site is in Madibou a suburban area located in Makélékélé, one of the seven districts of Brazzaville. According to the data obtained from the General Census of Population in 2007, there were 1,373,382 inhabitants in Brazzaville, and Makélékélé, one of its seven districts, had 298,292 inhabitants. In this study area, Ngoko, Kinsana, and Mayala are part of Madibou; Ntietie, Malanda Yabi, Collinaud, and Nkoutou are part of Mbouono. A public health facility is located in Madibou which refers some patients to the district hospital in Makélékélé, located in the centre of the district. In the 1980s, malaria transmission had been described as intense and perennial, with an entomological inoculation rate of 200–1,000 infective bites/person/year. Since then, there has been no update with more recent entomological studies. Of 17,636 inhabitants in the study area, 3,058 (17.3%) were children under 10 years of age.

The *Pyramax* study will be conducted also at the hospital of Makélékélé (Southern area). The prevalence of clinical malaria infection ranges currently between 12 and 25% and 20 patients per day could be recruited during the study period.

Democratic Republic of Congo

- **Study sites**

The Principal site and Satellite sites are overseen by the Principal Investigator who is located at Université de Kinshasa (UoKPV) and the satellite sites are based at Institut Médicale Évangélique de Kimpesé (IMEK) in Kimpesé (Bas Congo), and Centre Hospitalier Mont-Amba (CHMA) in Kinshasa.

In the region of Kinshasa, two seasons (rainy and dry) are accounted for and the rainy season lasts for 8 months. Malaria transmission is stable almost all the year. Malaria infection is high, around 54000 cases were reported last year with predominance in children under 5 years old (around 50%).

Country	Site	0 - 5 years	> 5 years	Pregnant women	Microscopy Positive	RDT positive
Democratic Republic of Congo	Kinshasa<br>Delvaux (Urban site)	5266	9254	7063	2886	2422
	Kinshasa<br>Kingasani (semi-rural site)<br>Institut Médicale Évangélique de Kimpesé<br>Kimpesé, Bas-Congo<br><br>Centre Hospitalier Mont-Amba<br>Université de Kinshasa	4817	6553	815	3742	3776

(18, 19, 20, 21, 22, 23, 24).

Gabon

- **Lambaréné**

Current WHO reports indicate that the entire population of Gabon (estimated at 1,670,000 in 2013) live in malaria endemic regions. Malaria transmission is perennial with a hyper-endemic transmission pattern. *P. falciparum* is responsible for 95% of human malaria cases and *P. ovale* (wallikeri and curtisi) and *P. malariae* are prevalent as mono- and co-infections throughout the country. Main vectors are *An. gambiae* and *An. funestus*.

Malaria incidence per 1000 persons/year is estimated at 228 (25). Children are the main patient population suffering from malaria in Gabon. Since 2003 first line treatments of *P. falciparum* malaria is ASAQ and as a secondary option AL. ACTs are free of charge in the public sector since 2003. Since 2009 patients should receive a diagnostic test before treatment. ITNs/LLINs are recommended to all age groups since 2007, IRS is recommended since 2013, and IPTp is used to prevent malaria during pregnancy since 2003.

Version 9.0 Date 15/03/2019: Property of SHIN POONG/MMV- strictly confidential

- **Study site**

The Principal Investigator is based at Albert Schweitzer Hospital; Lambaréné.
In 2014 malaria was responsible for 26% of hospital consultations among children (0-14 years of age) at a sentinel centre in central Gabon (Albert Schweitzer Hospital; Lambaréné 2014). Children <1 year of age constitute approximately 9% of paediatric malaria cases at this sentinel hospital in Lambaréné, Gabon. Children aged 1-5 years and 6-14 years constitute each 45.5% of paediatric malaria patients attending the hospital (Lambaréné 2014).

Ivory Coast

- **Agboville**

Ivory Coast has a high prevalence of parasitic infectious diseases including malaria, which is a significant public health problem.
Malaria is the leading cause of hospitalisation and consultation in the country's health centres. In 2010, 43% of morbidity in health care centres was due to malaria (NMCP, 2010 situational analysis).
Children under 5 years of age suffer 1 to 6 malaria's episodes per year, with an average of 3 malaria attacks per child. The number of attacks for adults is 1 to 3 episodes per person and per year, especially in rural areas (26).
Malaria accounts for 33% of all causes of death in the hospitals. (NMCP 2004 data)
The entomological inoculation rate varies by region from 150 to 400 infective bites per person and per year (27).
Ivory Coast, as most of the African countries, followed the WHO recommendations and changed its management policy of uncomplicated malaria. The protocol, ratified in January 2007 in Ivory Coast, recommends using ACTs for the treatment of uncomplicated malaria and supports the use of both ASAQ and artemether-lumefantrine as 1st line therapy.

- **Study sites**

The Principal site and Satellite sites are overseen by the Principal Investigator who is located at Institute Pierre Richet (IPR) and the satellite sites : Agboville, Centre de Santé Urbain d'Azaguié (CSUA) and Centre de Santé Urbain de Grand Morié (CSUGM).

The *Pyramax* study will be conducted in the health district of Agboville. This district is not far from Abidjan and is organized around several health centres for proper health coverage. The region has a stable malaria endemic setting reflecting the situation of southern Ivory Coast. The Health Centre of Azaguié was chosen initially to perform the prospective collection of safety data in real life. Additional centres at Grand Morié, Aboudé-Mandeké and Rubino may be opened in the event of difficulty of recruiting or in the case of closure of a center in one of the other countries participating in the study. According to the NMCP data, more than 2,000 malaria episodes could be included in this center in one year.

3.4 Project rationale

The project will take place in four Central African countries and in one West African country and it has dual objectives.

The first is to collect safety data in real-life conditions on the use of a medicine for which the safety profile is well known only through classical clinical studies. The second is to develop

new methods for actively performing pharmacovigilance, which will subsequently be used for other anti-malarial drugs, and drugs in other therapeutic classes in sub-Saharan Africa.

This programme is being implemented in close collaboration with the National Malaria Control Programmes (NMCP) of each of the five countries and the Pharmacovigilance Department of the Sponsor. The collected data will be regularly submitted to an independent Drug Safety Monitoring Board (DSMB) committee in charge of safety monitoring, as well as to the Health Authorities and will be communicated by the Sponsor to the WHO collaborating Pharmacovigilance centre in Uppsala via the National Pharmacovigilance Center of DRC who act as a regional pharmacovigilance service in this project.

3.5 Proposed Sites participating in the study

1) Cameroon: University of Yaoundé 1, Yaoundé and the satellite site Mfou District Hospital.
2) Republic of Congo, Centre de santé FCRM – Massisia and the referring sites: Centre de santé de Madibou , Brazzaville and Hôpital de base de Makélékélé, Brazzaville.
3) DRC : Université de Kinshasa (UoKPV) and the satellite sites Institut Médicale Évangélique de Kimpesé (IMEK) in Kimpesé (Bas Congo), and Centre Hospitalier Mont-Amba (CHMA) in Kinshasa.
4) Gabon, Albert Schweitzer Hospital at Lambaréné (CERMEL).
5) Ivory Coast, Institute Pierre Richet (IPR) and the satellite sites : Agboville, Centre de Santé Urbain d`Azaguié (CSUA) and Centre de Santé Urbain de Grand Morié (CSUGM).

Other sites can be introduced by the Sponsor in conjunction with PI and MMV to optimise the execution of the study and achieve the required sample size.

4. STUDY OBJECTIVES

The main objectives of the study are to assess the safety of *Pyramax* particularly in patients with underlying liver function abnormalities, in patients who have co-morbid conditions, such as HIV (where known), and also in very small children (<1 year of age).

4.1 Primary objective

Evaluation and identification of the hepatic safety events of *Pyramax* in a subgroup of patients enrolled with LFTs >2xULN from blood taken immediately prior to treatment without any clinical signs or symptoms of hepatotoxicity and with signs and symptoms of uncomplicated malaria confirmed by a Rapid Diagnostic Test (RDT) or microscopy (thick blood smear).

4.2 Secondary objectives

- 4.2.1 Main secondary

Comparison of the clinical hepatic safety of *Pyramax* between a cohort of patients enrolled with LFTs >2xULN and a cohort of patients enrolled with normal LFTs matched for demographic characteristics.

- 4.2.2 Other secondary
  - Evaluation of the safety and tolerability in patients with normal and abnormal LFTs at inclusion according to any possible hepatic underlying disease based on the finding of the hepatitis panel and according to their HIV status (where known), their nutritional status, their age (children < 1 year of age in comparison to the rest of the treated population) and their weight.
  - Evaluation of the safety and tolerability in retreated patients with a special focus on the hepatic safety.
  - Evaluation of the potential for hypersensitivity.
  - Evaluation of the relationship between the occurrence of hepatic related adverse events with or without LFT abnormalities and the administration of concomitant medications (in particular paracetamol, herbal medicines and antiretroviral drug).
  - Evaluation of the efficacy and compliance of *Pyramax* when used under usual conditions (including unsupervised medication intake) in patients with signs and symptoms of uncomplicated malaria confirmed by a RDT or microscopy (thick blood smear).

5. DESCRIPTION OF THE STUDY

5.1 Description of the study design

This is a non-comparative Cohort Event Monitoring study of 8,572 malaria episodes to be conducted in Central and West Africa. Treatment is with *Pyramax* as per the product label and Investigator Brochure. Each patient will be provided with 3 days oral treatment with *Pyramax* to be administered on days 0, 1 & 2.

Total study duration for a single patient episode is 28±2 days.

A cohort of approximately 2% of children who are <1 year of age will be included.

5.1.1. Study assessments

Screening/Inclusion/Day 0:
Patients for whom a diagnosis of uncomplicated malaria (according to WHO criteria) is suspected, will undergo an RDT or microscopy. If the presence of malaria is confirmed and the patient meets all inclusion criteria and none of the exclusion criteria and the patient (or parent/ guardian in case of children) provides his/her informed consent, he/she will be assigned a unique ID number and will be enrolled in the study. Blood will be taken for PCR analysis (blood spot), for retrospective LFT assessment as well as for a retrospective viral hepatitis assessment to be done only in those patients with abnormal baseline LFTs (AST or ALT >2 x ULN). A blood sample will be taken for Haematology (Hb mandatory).

The first dose of *Pyramax* will be given under Direct Observational Treatment (DOT) conditions, and patients will take their second and third doses of *Pyramax* under usual conditions (including unsupervised medication intake). Patients will be also informed of the

requirement to attend the health care facilities if they have any clinical signs or symptoms of hepatotoxicity.

Day 7 or any unforeseen visit
The patients will be visited at home at Day 7 ± 1 day, by a community health worker (CHW), in order to capture the adverse events (AEs) experienced. The patient's body temperature will be taken. In the case of signs or symptoms of hepatotoxicity or hepatic related AEs, the patients will be referred to the health facilities and blood will be taken on this occasion for LFTs and haematology (Hb mandatory) assessment as well as for a full hepatitis panel. These investigations will be performed immediately. If a patient cannot be directly reached, the CHW will have to organise an appointment with this patient before Day 10. Special procedures will be followed in case of serious adverse events (SAEs) and/or severe adverse events and events classified as being of special interest (AESI) (see specific section).

At any unforeseen visit, in addition to the procedures above, malaria diagnostics and blood spot PCR analysis will be undertaken.

Day 28
At Day 28 ±2 days, all patients will be seen by a CHW for a final assessment. At this occasion, blood will be taken for thick blood smear and blood spots. In the case of signs or symptoms of hepatotoxicity or hepatic related AEs occurring since the previous Day 7 visit, the patient will be referred to the health facilities and blood will be taken for LFTs and haematology (Hb mandatory) assessment as well as for a full hepatitis panel.

Female patients must communicate to the local CHW or the study team if they get pregnant just before or after the start of the *Pyramax* treatment and for 2 months post-treatment. All pregnancies must be documented on the manufacturer's *Pyramax* Pregnancy Register. In these cases, the evolution of the pregnancy will be monitored with visits at 3, 6 and 9 months and 7 days after the delivery. Information on the drugs taken during the pregnancy as well as AEs/SAEs/AESIs and the health status of the newborn(s) will be collected. A urine pregnancy test will be performed for all women of child-bearing potential, on the last day of follow-up (usually Day 28 ±2 or in case of early termination, on the respective day), at home or at the health facility, if the patient is transferred to the health facility. Women of child-bearing potential are defined as women who have experienced menarche and who are not permanently sterile or postmenopausal (defined as 12 consecutive months with no menses without an alternative medical cause). The investigator shall decide whether this is applicable or not based on the medical history of the patient, and on customs and practices of the region.

5.2 Study duration

The study is anticipated to last approximately 18 to 24 months and will end when 1) the total number of malaria episodes (n=8,572) is reached and 2) at least 120 episodes of malaria in patients with raised liver enzymes at baseline have been recruited.

5.3 Drug prescription and dosing

The patients will be instructed to take one *Pyramax* (pyronaridine-artesunate) dose orally every 24 hours over a period of three consecutive days, i.e. at Day 0, then after 24 hours (Day 1) and after 48 hours (Day 2) from the first administration.

Version 9.0 Date 15/03/2019: Property of SHIN POONG/MMV- strictly confidential

The dose will be based on body weight. Two formulations of *Pyramax* will be provided to facilitate the dosing in young infants, children and adults: 60:20mg granules presented in sachets and 180:60mg tablets of pyronaridine and artesunate respectively. For this study, *Pyramax* tablets are sent as bulk supplies to site in commercial blister packs of 90 tablets. Within the pack there are 10 blisters packs with 9 tablets in each.
Only the first dose will be administered under supervision in the clinic, the second and third doses will be taken by the subject at home.

**Body weight**	**No. of Sachets (granule formulation) per day**
5 to < 8 kg	1
8 to < 15 kg	2
15 to < 20 kg	3
	**Number of Tablets per day**
20 to < 24 kg	1
24 to <45 kg	2
45 to < 65 kg	3
≥65kg	4

Pyramax will be provided free of charge by the Sponsor, Shin Poong Pharmaceutical Co, Ltd.

5.4 Evaluation criteria

Primary evaluation criterion:
A safety analysis will be performed retrospectively on a sub-group of patients identified with abnormal baseline LFTs (AST/ALT >2xULN). In this sub-population, clinical hepatic safety will be assessed through the specific analysis of hepatic adverse events captured by the CHW at D7 ± 1 day or at any unforeseen visits in case of signs or symptoms of hepatotoxicity or hepatic related AEs, after confirmation by biochemistry assessment.

Main secondary evaluation criterion:
Comparative analysis of the clinical hepatic safety (signs or symptoms of hepatotoxicity or hepatic related AEs confirmed biologically) will be made between patients having abnormal LFTs (AST/ALT >2xULN) at enrolment versus a cohort of patients with normal LFTs matched for demographic characteristics.

Other secondary evaluation criteria
- A safety analysis will be performed in patients with normal and abnormal LFTs at inclusion according to any possible hepatic underlying disease based on the finding of the hepatitis panel and according to their HIV status (where known), their nutritional status, their age (children <1 year of age in comparison to the rest of the treated population) and their weight.
- Safety (including hepatic safety) in retreated patients.

- Incidence of hypersensitivity reactions
    - o At any unforeseen visits in case of signs or symptoms of hepatotoxicity or hepatic related AEs, special efforts will be made to assure and record adequate follow-up of serious and/or severe adverse events and of the adverse events of special interest (see specific section).
    - o Biological/LFTs assessment (AST/ALT bilirubin total and conjugated, and alkaline phosphatases). Assessment of baseline LFT results where signs or symptoms of hepatotoxicity or hepatic related AEs were reported at Day 28 or at any unforeseen visit.
- The relationship between the occurrence of hepatic related adverse events with or without LFT abnormalities and the administration of concomitant medications (in particular paracetamol, herbal medicines and antiretroviral drugs) will be evaluated.
- Crude Day 28 cure rate by species and PCR-adjusted cure rate. for Day 28 cure rate for *P. falciparum*
- Time between malaria episodes and frequency of repeat episodes before and after Day 28. Compliance will be assessed by interview and counting the number of tablets or sachets of granules dispensed but not taken, when the CHW visits the patient at home.

6. STUDY POPULATION AND PATIENT SELECTION

6.1 Sample size

Based on the primary objective, 120 malaria episodes in patients with baseline raised AST/ALT value >2xULN will be needed for a 81.6% probability to observe one severe hepatic event in this population, defined as:

- Appearance of clinical signs and symptoms of possible hepatotoxicity associated with a rise in ALT/AST >2 x baseline value.

Given the screening rate in previous studies of 1.4% for malaria patients with AST/ALT >2xULN, recruitment will require an estimate of at least 8,572 malaria episodes. To mimic reality, patients can be included more than once in the study. At each new malaria episode, the patient will be assigned the same 8 first digits of his ID number, followed by E01, E02, etc, to illustrate repeated treatment 1, 2, etc.

The study duration is estimated to be approximately 18 to 24 months after study start, depending on enrolment being completed over 1 or 2 malaria seasons. However, the study will not be terminated until at least 120 malaria episodes in patients with LFTs >2xULN are enrolled.

6.2 Selection criteria

6.2.1 Inclusion criteria

- Uncomplicated malaria (*Plasmodia* of any species) diagnosed as per national policies and in line with WHO recommendations:
    - o Fever or history of fever in the previous 24 h and/or the presence of anaemia, for which pallor of the palms appears to be the most reliable sign in young children.

  - o Confirmation of malaria by a parasitological diagnosis (RDT or Microscopy (thick blood smear)).
- Weight ≥5 kg - <20 kg (granules); ≥20 kg (tablets).
- Ability to take an oral medication.
- Ability and willingness to participate based on signed informed consent (a parent or a guardian has to sign for children below 18 years old) and on signed assent form for minors that could be required per national regulations in each participating country.
- The patient has to comply with all scheduled follow-up visits.

6.2.2 Exclusion criteria

- Patients with clinical signs or symptoms of hepatic injury (such as nausea, abdominal pain associated with jaundice) or known severe liver disease (i.e. decompensated cirrhosis, Child-Pugh stage 3 or 4).
- Known allergy to artemisinin and/or to pyronaridine.
- Known pregnancy.
- Lactating women should be excluded if other anti-malarial treatments are available.
- Complicated malaria as per WHO 2012 definition (Annex 2).
- Patients that the investigator considers would be at particular risk if receiving an anti-malarial or if participating in the study.
- Patients having been treated with *Pyramax* in the previous 28 days.

6.3 Enrolment procedure

6.3.1 Investigational Sites

The Study will be conducted at selected health facilities from the following countries: Cameroon, Republic of Congo, DRC, Gabon and Ivory Coast. A list of sites is provided in section 3.5.
Additional sites can be added at the discretion of the Sponsor, MMV and PI if required.

6.3.2 Patient selection

At least 8,572 episodes of malaria are needed in the study. To mimic reality, patients can be included more than once in the study. A washout period of at least 28 days must be maintained between two consecutive treatments with *Pyramax*. A subset of approximately 2% of children under the age of 1 year will be recruited.

A patient will be included in the study where he/she meets the selection criteria of the protocol and agrees to participate. In particular, to enter the study, the patient should accept: (i) that a small amount of blood is taken for the preparation of blood spots, as well as for a LFTs assessment (ii) that the CHW visit him/her at home, (iii) that the data collected during those visits are registered and analysed and finally (iv) that, in case an SAE and/or an AE classified as severe and/or an AESI (see section 8.5) should occur he/she is to contact the CHW immediately.

Version 9.0 Date 15/03/2019: Property of SHIN POONG/MMV- strictly confidential

6.3.3 Patient numbering

Each patient will be identified at the study site by a 10 digits number, referred to as patient ID number. The first digit is the country number, the following two digits identify the centre and the next four digits identify the patient enrolment number. A letter E will follow and the last two digits will indicate the malaria episode for which the patient is being treated, starting with 01 for the first time the patient is treated in the protocol. Each number will be unique and will not be assigned to another patient, including any premature discontinuations. At each subsequent malaria episode, the patient will be assigned the same first 8 digits of the ID, up to the letter "E" , followed by the corresponding Episode number, to illustrate repeated treatment (02, 03, etc).

7. STUDY PROCEDURE AND DATA COLLECTION

7.1 Visit planning

Inclusion in the study and the required assessments for inclusion will be performed at the study centre or at one of the associated health facilities, on Day 0 during the consultation.

The first follow-up home visit with the patient will be performed by a trained CHW on Day 7 (± 1 day) after administration of the first *Pyramax* dose.

In addition, patients will be requested, in the event of any unusual significant symptom experienced during the 28 days following the treatment prescription, to visit their village CHW or contact directly the health agent who followed him/her during the study.

In case of an occurrence of an SAE, or an AE classified as severe or an AESI (see section 8.5), or if requiring medical care, the patient will be transferred to the study designated health facility by the village CHW in order to receive care for his/her symptoms (this will be an unforeseen visit) and to provide more detailed information on the event.

7.2 Collected data

7.2.1 Registration at the clinical study centre

Each patient visiting one of the designated study health facilities, screened for suspected uncomplicated or complicated malaria, regardless of their inclusion in the study, will be formally registered by the Study Team Member (STM) who has examined him/her. The date of consultation, the age and sex of the patient, the village or neighbourhood he/she lives in, the recent history of any anti-malarial treatment intake, the weight, the clinical signs, the diagnosis, the prescribed treatment (dose and duration) and any other medications within 7 days will be reported in this document. Where applicable the reason for not participating in the study (screen failure) will be documented. For patients consenting to the study, their unique patient ID will be recorded in the registry.

For the patients included in the study, the dates on which the patient's chart was provided to the CHW (by the STM) on which the CHW consigned the completed chart with data referring the follow-up contact at Day 7 (±1 day) at the STM will be included in the registry; those dates will be reported for traceability purposes.

7.2.2 Examination and data collection at inclusion

Where the patient agrees to participate in the study, the following procedures will be followed (Day 0):

- An informed consent form will have to be signed and will be filed with the medical records.
- The STM will assign a patient ID number to the patient, and record the following data:
  - o For the Site Registry: last name, first name, full address (village/neighborhood), registration number and patient ID number.
  - o In the Case Report Form (CRF*): the other information collected during this visit, i.e.:
    - Demographic data: patient ID number, initials, age, sex, weight, height and, vital signs (pulse and blood pressure), body temperature.
    - Physical examination will include general appearance, skin, head and neck, eyes, ears, nose, throat, lymph node palpation, lungs, heart, chest, abdomen, neurological function.
    - History of anti-malarial drugs taken during the 4 weeks before inclusion.
    - Any other treatment including traditional treatments, taken during the 2 weeks before inclusion.
    - Concomitant treatments including traditional medication and treatments prescribed when visiting the centre.
    - Malaria and associated disease symptoms to be reported.
    - Confirmation that a blood sample for malaria diagnosis (RDT or thick blood smear and blood spot for PCR), for haematology (Hb mandatory), for blood chemistry (at least AST, ALT, Bilirubin total and conjugated, ALP) and for viral hepatitis assessment has been drawn.
    - Review of inclusion and exclusion criteria, and eligibility for the study.
    - *Pyramax* treatment administration time. Dosage will be defined according to the weight of the patient. The patient will be instructed to take the medication with water according to the administration instructions. For all patients the first dose of the study drug will be directly administered at the facility and time of administration will be documented.

* The term CRF is used generically, the actual data will also be recorded in electronic format or eCRF. The detail will be described in the separate Data Capture and Management Plan. The terms CRF and eCRF are therefore used interchangeably.

7.2.3 Follow-up visits/contacts and clinical tolerability follow-up questionnaire

On Day 7 (± 1 day) (after administration of the first *Pyramax* dose) the CHW will visit the patient at home. He/she will then record the following data:

- His/her own 4 digit identification number (first two digits are the number of the health centre he/she is attached to, followed by a 2 digit number which will be assigned to him/her when the study starts).
- Date of the visit/contact.
- Clinical condition (recovered status or continuation of the illnesses).
- Body temperature will be taken.
- Compliance with the prescribed *Pyramax* treatment and in case the correct number of tablets or sachets of *Pyramax* was not taken, an explanation of the reason why the treatment was not followed should be provided.

- Description of the symptoms experienced by the patient in case of a positive answer to the question: "*Have you noticed any unusual event/symptom since your last visit at the centre?"*. Collect date of first manifestation of this/these event(s), symptom(s), intensity, treatment dis-/continuation, and evolution, in the specific section of the patient chart dedicated to the collection of Adverse Events.
- Pregnancy status will also be enquired about.
- Current treatments

In case, during his/her contact, the CHW assess that an event that has occurred is serious and/or severe, or detects an adverse event of special interest (see section 8.5), he/she should contact the STM and transfer the patient immediately to the health facility to be examined. If necessary in the opinion of the STM, he/she should contact the investigators so that they can visit the patient and characterise the status of the event and the causality. If the adverse event belongs to the hepatic category, after transfer to the health facility, blood will be drawn for chemistry (at least AST, ALT, Bilirubin total and conjugated, ALP) and haematology (Hb mandatory) and a full hepatitis panel. In that event, a specific adverse event report form will be completed by the STM or the investigators.
In addition, thick blood smears will be prepared as well as blood spots for future analysis.
In case any treatment is prescribed by a STM staff or the study doctor, it will be reported in the CRF and on the patient chart.

All the patients will be instructed to refer to the CHW or the designated STM should any adverse event occur within the 28 day after the start of the *Pyramax* administration.
In addition the regional hospital will be alerted of the ongoing study, and there will be a collection on a regular basis of any data related to patients who are treated in regional hospitals who have taken part in the study.

Female patients will be encouraged to communicate to the study team if they get pregnant within a period of two months after the start of the *Pyramax* treatment. In these cases, information on the evolution of the pregnancy will be collected at 3, 6 and 9 months and at 7 days after the delivery. Information on the drugs taken during the pregnancy as well as AEs/SAEs/AESIs and the health status of the newborn/s will be collected and reported in the patient chart and will be transmitted to the Manufacturer for documentation on the *Pyramax* pregnancy register.

On Day 28 (±2 day) the CHW will visit the patient for a final study visit at home. He/she will report:

- His/her own 4 digit identification number (first two digits are the number of the health centre he/she is attached to, followed by a 2 digit number which will be assigned to him/her when the study starts).
- Date of the visit/contact.
- Clinical condition (recovered status or continuation of the illnesses).
- Body temperature will be taken.
- Description of the symptoms experienced by the patient in case of a positive answer to the question: "*Have you noticed any unusual event/symptom since your last visit to the centre/contact with CHW?"*. Collect date of first manifestation of this/these event(s), symptom(s), intensity, treatment dis-/continuation, and evolution, in the specific section of the patient chart dedicated to the collection of Adverse Events.
- Drops of blood will be taken for thick blood smears and spots for PCR.

- Any pregnancy: a urine pregnancy test will be performed for all women of child-bearing potential.
- Current treatments.

If the adverse event belongs to the hepatic category, after transfer to the health facility, blood should be drawn for chemistry (at least AST, ALT, Bilirubin total and conjugated, ALP) and haematology (Hb mandatory) and a full hepatitis panel.
Should the patient present at the clinic or make contact with the CHW, it might be required to conduct an unscheduled unforeseen visit to report:

- Date of the visit/contact.
- Clinical condition (recovered status or continuation of the illnesses).
- Body temperature will be recorded
- Description of the symptoms experienced by the patient in case of a positive answer to the question: "*Have you noticed any unusual event/symptom since your last visit to the centre/contact with CHW?".* Collect date of first manifestation of this/these event(s), symptom(s), intensity, treatment dis-/continuation, and evolution, in the specific section of the patient chart dedicated to the collection of Adverse Events.
- Drops of blood will be taken for thick blood smears and spots for PCR.
- Pregnancy status.
- In case of early termination, a urine pregnancy test will be performed for all women of child-bearing potential.
- Current treatments.

7.3 Patients who did not receive follow-up visits/contacts or who have prematurely discontinued treatment

7.3.1 Premature treatment discontinuation

The reasons for any premature treatment discontinuation will be collected by the CHW or STM staff during his/her visits/contacts and if the reason for stopping the treatment is the occurrence of an adverse event, this will be described on the specific section of the patient chart and treated depending on its category (see section 8).

A patient can be withdrawn from the study for any of the following reasons:

· Death
· Loss to follow-up
· Withdrawal of consent
· The investigator or Sponsor believes (e.g, for safety or tolerability reasons, such as a diagnosis of severe malaria or cerebral complications, or other adverse events) that it is in the best interest of the subject to discontinue treatment.

There will be no replacement for patients with premature discontinuation.

7.3.2 Patient lost to follow-up

The study staff will take all appropriate measures to perform each patient's follow-up visits. However, some patients may be lost to follow up. This information will be recorded in the

patient's chart. A patient will be considered lost to follow up if he cannot be visited by the CHW before Day 10.
All reasonable efforts must be made by the study site personnel to contact the subject for the Day 28 visit, to determine the endpoint status and the reason for discontinuation/withdrawal until a reasonable period.

7.4 Thick blood smear and blood spot logistics

Blood slides will be performed and blood spots collected on Day 28 for all the participants. At the inclusion (Day 0) visit, a thick blood smear can be replaced by RDT. All thick blood smear slides will be stained and stored for independent confirmation of presence or absence of malaria parasites. They will be examined in the laboratory by qualified personnel and according to the laboratory's Standard Operating Procedures. The results will be available for inclusion in the final study database.

7.5 Hepatitis panel logistics:

Viral hepatitis assessment at baseline:
On Day 0, after inclusion and before treatment, blood for viral hepatitis assessment will be drawn from all patients.
Viral hepatitis assessment shall include: hepatitis A, B, C, delta (if hepatitis B positive) and E. Blood for viral hepatitis assessment will be kept frozen at -20°C.
For patients retrospectively found with baseline AST or ALT >2xULN, the frozen blood for viral hepatitis assessment will be sent to the central lab for analysis immediately. For all other patients (i.e. with AST and ALT <2xULN) the blood for baseline viral hepatitis assessment will be destroyed.

Full hepatitis panel:
During the study at D7, D28 or at any unforeseen visit, in case the CHW detects possible clinical signs or symptoms of hepatotoxicity, the patient will be referred to the health centre. At the health centre, blood will be drawn for at least AST, ALT, ALP, total and conjugated bilirubin and for a full hepatitis panel:

- Hepatitis A, B, C (Anti-HAV IgM, Anti-HBc IgM, HBsAg, and hepatitis C RNA),
- Hepatitis E IgM antibody,
- Cytomegalovirus (CMV) testing polymerase chain reaction (PCR) testing,
- pp65 antigen, or IgM antibody,
- Epstein Barr virus (EBV) viral capsid antigen IgM antibody,
- Serum creatinine phosphokinase (CPK) and lactate dehydrogenase (LDH),
- Alkaline phosphatase (if not already collected),
- Serum transferrin saturation and serum ferritin (diagnosis of hemochromatosis),
- Liver biosynthetic capacity: albumin and prothrombin time (+ prealbumin, serum ceruloplasmin, procollagen III peptide, α-1-antitrypsin and α-feto protein, when possible).

For patients with AST or ALT >3xULN (when baseline was normal) or for patients with AST or ALT >2x baseline value (when baseline value for AST or ALT >2xULN) the blood for full hepatitis panel should be sent as soon as possible to the central lab for analysis.

For all other patients, i.e. patients with AST and ALT <3xULN (when baseline was normal) or with AST and ALT <2x baseline value (when baseline value for AST or ALT >2xULN) the blood for the full hepatitis panel will be destroyed.

7.6 Vital signs and Physical examination

Vitals signs include body temperature, weight, pulse rate and blood pressure. Blood pressure and pulse rate will be measured after the patient has rested for 3 minutes. The physical examination will include general appearance, skin, head and neck, eyes, ears, nose, throat, lymph node palpation, lungs, heart, chest, abdomen, neurological function.

8. SUBJECT SAFETY

8.1 Training

Before the start of the study, the following training programmes will be completed:

The health facility study staff will be trained on the technique of thick blood smear preparation and staining according to the local Standard Operating Procedure on the use and reading of the RDT, on the blood spots preparation and on the detection of signs or symptoms of hepatotoxicity and hypersensitivity reactions.

The medical staff of the clinical study centres as well as the CHW and the health care provider (HCP) will be trained on the tolerability profile of *Pyramax*, on pharmacovigilance procedures (adverse events recording, adverse events management etc.) and on other study specific procedures (registry maintenance, CRF completion, non-directional/open patient questioning, filling data sheets), data management systems and tools. The medical staff of the referral hospitals will additionally be trained on the detection of hepatotoxic events. GCP training will be provided as appropriate.

8.2 Monitoring of adverse events

Patient charts (completely filled in with the contact visit at Day 7 (±1 day), and with any AE(s) reported in the 28 days after the start of the treatment) will be captured in the CRF by the Principal Investigator (PI) of the study centre via the electronic capture system. Chart review meetings shall be organised regularly, during which the PI, his/her team and the people responsible for the pharmacovigilance in the country participating in the study shall review AE data (intensity, causality, date of event manifestation in relation to the start of treatment and the initial parasite species identified on the patient). Given the absence of pharmacovigilance systems in most countries involved in the study, the experience of Shin Poong Pharmacovigilance (SPPV) and national pharmacovigilance center of DRC (CNPV) will be used in this study. The principal investigator in each country and his team will make a first analysis of the safety reports received in order to assess completeness. Safety data should be entered immediately in the eCRF. Safety reports are reviewed by Shin Poong Pharmacovigilance (SPPV) for quality control. The medical monitor will have access to the trial database. He will have access to the electronic version of the CRF and can address a request to the principal investigator of the site concerned to add any further information needed for the analysis. The medical monitor will conduct the final review of the CRF for any patients who have had an adverse event. The eCRF provider will code each adverse event reported according

to standard medical terminology (MedDRA) and the medical monitor will then review the list of coded terms. The PI will make a causality assessment. SPPV/CNPV/PI should be informed about any new data on a patient for whom a causality link had already been established to reconsider its analysis and if necessary reassess the causality. Among these new data will be included any data acquired from the hepatitis panel results, where appropriate, the recurrence of the same adverse event in a patient who is retreated after a subsequent episode of uncomplicated malaria, etc. Data shall only be modified following an official query procedure. Safety data will be transmitted to the WHO Collaborating Centre for Pharmacovigilance via the CNPV of DRC to achieve a quality control of the review and to provide necessary feedback to CNPV and SPPV.
The SAE reporting must follow notification requirements in Section 8.4. SAEs and AESIs will be coded using the latest version of MedDRA by SPPV and will reconcile all SAEs in the SPPV database with those in the CNPV database on a regular basis.

An independent study-specific Data Safety Monitoring Board (DSMB) will conduct periodic safety reviews (see Section 11).

8.3 Definition of Adverse Event and Serious Adverse Event

An **Adverse Event** (AE) is a sign, symptom, syndrome, disease or biological anomaly suffered by a patient or a subject participating in a clinical study and receiving a medicinal product. This term does not imply a causal relationship with the concerned treatment. Clinical signs typical of an acute malaria episode will not be considered AEs unless the healthcare personnel considers these events as exceptional due to their evolution, their seriousness, or another factor related to these events (cf. annex 3 for grading of AEs).

A **Serious Adverse Event** (SAE) is an adverse event which:

- causes death or
- is life-threatening or
- necessitates hospitalisation or prolongs hospitalisation or
- results in persistent or significant disability/incapacity or
- is a congenital defect or malformation
- is another medically important event
- constitutes a possible Hy's Law case following the definition given in Annex 4.

A decision on medical and scientific grounds is required to assess whether an immediate notification of an event is warranted in other situations, such as medically important events which are not life-threatening, fatal or cause hospitalization, but could endanger the patient or required an intervention to prevent one of the above conditions developing.

Remark: Examples of such events are intensive care in the emergency room or at home to treat a bronchospasm; a haematological dyscrasia or convulsions not causing hospitalisation, or the development of drug addiction or abuse.

8.4 Obligation of AE notification

Adverse Events

The AEs, regardless of their seriousness and causal relationship to the study drug, arising between the first administration of study medication and the last study visit (as per the protocol),

must all be recorded on the patient CRF (AE recording section). When possible, the symptoms must be regrouped within a single syndrome or diagnosis. The healthcare personnel shall have to specify the date of manifestation of the event, its intensity, final evolution, the measures taken and the treatment undertaken (if any).

Serious Adverse Events

In case of SAEs independent of causality, the healthcare personnel must immediately contact the PI / Investigator for validation of the seriousness and determination of the causality. Subsequently, the procedure described below must be followed:

- SEND (within 24 hours and fax or pdf scanned documents) the signed and dated copy of the "Adverse Event form" and the form "SAE complementary information" to SPPV (Tel/Fax[redacted])]
    - SPPV will send notification to CNPV-UPC
- CALL immediately (the same day) the medical monitor Dr. Selidji Todagbe Agnandji, responsible for safety in case of death or life-threatening events.
- CALL the local responsible person for pharmacovigilance of the project and where appropriate Shin Poong QPPV.
- INFORM within 48 hours the Ethic Committees of the occurrence of any serious adverse event as per local regulation.
- The follow-up of each fatal or life-threatening AE must be provided to the medical monitor and the SPPV within the same timeline as the initial report (within 24 hours and preferably by email).
- Attach to the chart the photocopy of all available results and examinations which were undertaken (and their date). Analysis results must be accompanied by the laboratory normal ranges. Special consideration shall be taken to ensure patient anonymity, and to the correct completion of the patient's study specific identifier in the copies of the source documents provided to the Sponsor.

Follow-up of Adverse Events

The healthcare personnel must take all appropriate measures to protect the safety of the patients. Personnel must ensure to document follow-up of the evolution of each adverse event (clinical, biological or other) until resolution or until the stabilization of the patient's status.

All new relevant information concerning the initial SAE shall be recorded on a form "SAE follow-up information form" by the nursing staff of the health centre, and shall be validated by the PI/co-PI who shall transfer the form to the local person/physician responsible for the pharmacovigilance of the project and the Shin Poong PV.

In case of a serious adverse event the patient must be followed until complete resolution and normalization of all analysis results, or until chronicity of the patient's status. This can imply that the follow-up of the patient must continue beyond the period of follow-up per protocol, and that additional investigations could be requested by the Sponsor.

8.5 Adverse event of special interest

An adverse event of special interest (AESI) is an adverse event for which on-going monitoring is appropriate within the context of the study. These events necessitate complementary examinations in order to characterize and understand them.

AESIs in this study can be related to:

- Hepatotoxicity
- Hypersensitivity

The study team, as well as the relevant referral facilities, should be trained to take particular notice of symptoms/signs suggestive of the AESIs in this study.

In case of an AESI confirmed by the study physician, the *Pyramax* QPPV shall be informed within 24 hours, even if the event does not satisfy any condition of seriousness. Notification will occur through the use of an AESI form to [redacted]

Hepatic AESIs should be reported as follows:

a) For patients with normal LFTs at baseline:

- Present with fatigue, nausea, abdominal pain, itching or signs of jaundice such as:
    - dark urine,
    - putty or mastic coloured stools,
    - jaundice (yellowing of the whites of the eyes or skin),

and

- ALT or AST >3 x ULN

b) For patients with baseline ALT/AST >2xULN:

- Present with fatigue, nausea, abdominal pain itching or signs of jaundice such as:
    - dark urine,
    - putty or mastic coloured stools,
    - jaundice (yellowing of the whites of the eyes or skin),

and

- ALT/AST >2 x baseline value

In all these cases, patients shall be directed to the designated health facilities for evaluation and recording of all relevant information. If necessary, the health worker will contact the study team member or PI / co-PI.

In addition to the immediate assessment of baseline LFT for retrospective assessment, LFTs will be performed as soon as the patient arrives at the health facilities at any unforeseen visit and at Day 28, if required because of clinical signs or symptoms of possible hepatotoxicity or hepatic related AEs.

In the event of biological signs of hepatotoxicity associated with clinical symptoms/suspected Drug Induced Liver Injury (DILI), blood will need to be taken to perform the following

additional tests (full hepatitis panel): This blood sample shall be sent as soon as possible to the central lab for further analysis.

- Hepatitis A, B, C (Anti-HAV IgM, Anti-HBc IgM, HBsAg, and hepatitis C RNA),
- Hepatitis E IgM antibody,
- Cytomegalovirus (CMV) testing polymerase chain reaction (PCR) testing,
- pp65 antigen, or IgM antibody,
- Epstein Barr virus (EBV) viral capsid antigen IgM antibody,
- Serum creatinine phosphokinase (CPK) and lactate dehydrogenase (LDH),
- Alkaline phosphatase (if not already collected),
- Serum transferrin saturation and serum ferritin (diagnosis of hemochromatosis),
- Liver biosynthetic capacity: albumin and prothrombin time (+ prealbumin, serum ceruloplasmin, procollagen III peptide, α-1-antitrypsin and α-feto protein, when possible).

Hypersensitivity AESIs should be reported if patients show signs of hypersensitivity soon after treatment with *Pyramax* (particularly on repeat treatment) such as:

- Flushing
- The appearance of wheals / urticaria
- Breathlessness
- Faintness and/or fall in blood pressure

These events should be reported within 24 hours of knowledge to SPPV (Tel/Fax[redacted])]. The mechanism for reporting is the same as for an SAE; however, these will NOT be considered as SAEs unless they fulfil serious criteria as defined in Section 8.3.

8.6 Pregnancy

In case of pregnancy on initial presentation, the anti-malarial treatment shall be the one recommended by the NMCP. The patient will not be included in this study.

Female patients will be encouraged to communicate to their village CHW if they become pregnant within a period of two months after the start of the *Pyramax* treatment.

The evolution of the pregnancy will be monitored with visits at 3, 6 and 9 months and at 7 days after the delivery. Information on the drugs taken during the pregnancy as well as AEs/AESIs/SAEs and the health status of the newborn/s will be collected.

Pregnancy is not an adverse event unless the outcome of the pregnancy fulfils one of the serious criteria as defined in Section 8.3. Pregnancy should be reported by using the appropriate pregnancy report form within 24 hours of knowledge to SPPV (Tel/Fax[redacted])].

A urine pregnancy test will be performed for all women of child-bearing potential, on the last day of follow-up (usually Day 28 ±2 or in case of early termination, on the respective day), at home or at the health facility, if the patient is transferred at the health facility. Women of child-bearing potential are defined as women who have experienced menarche and who are not permanently sterile or postmenopausal, defined as 12 consecutive months with no menses

without an alternative medical cause. The investigator shall decide whether this is applicable or not based on the medical history of the patient.

8.7 Sponsor obligations

8.7.1 Safety

Throughout the study, Shin Poong Pharmacovigilance (SPPV) shall report expeditiously all unexpected SAE's which are reasonably related to the study drug (SUSARs), to each local responsible person for pharmacovigilance and those who are responsible to inform the local Ethics Committees in accordance with their procedures, to the investigators and to EMA through Eudravigilance portal. SPPV, through the local investigators, will ensure submission of the reported events to the competent authorities in accordance with local regulations.

Shin Poong Pharmacovigilance (SPPV) will collaborate closely with the National Pharmacovigilance Center of DRC (CNPV-UPC) who act as a regional pharmacovigilance service and who report to the WHO collaborating Pharmacovigilance centre in Uppsala.

Shin Poong will also report expeditiously all expected SAE's which are reasonably related to the study drug, to the local responsible person for pharmacovigilance. The latter will submit the reported events to the competent authorities in accordance with local regulations. Shin Poong will also report to European Medicines Agency as required.

Any AE not listed as being an expected event in the SmPC and in this protocol shall be considered to be unexpected.

8.7.2 Quality Assurance

Steps to be taken to ensure the accuracy and reliability of data include the selection of qualified investigators and appropriate study sites, review of protocol procedures with the investigators and associated personnel before the study, periodic monitoring visits by the assigned study monitors on behalf of the Sponsor, and direct transmission of clinical laboratory data from central laboratories into the study database.
Written instructions will be provided for collection, preparation, and shipment of blood samples and for thick blood smear procedures (study manual).

Guidelines for eCRF completion will be provided and reviewed with study personnel before the start of the study.

CRFs will be reviewed for accuracy and completeness during on-site monitoring visits by the study monitor, any discrepancies will be resolved with the investigators or designee, as appropriate. Following ongoing transfers of the data into the clinical study database further validation and checks will be performed by Data Management and any queries will be communicated to the investigator or designee for subsequent resolution

9. STATISTICAL METHODS

9.1 Data analysis

The statistical analyses will be performed using an appropriate software package such as SAS , version 8.2 or later versions, SAS Institute, NC, Cary, USA.
A detailed statistical analysis plan will be generated within three months of study start. This plan may be revised during the course of the study in order to take into account protocol amendments, if any, and to address potential issues occurring during the study, that could affect the planned analyses. A final, approved, statistical analysis plan will be in place before the database lock.

A DSMB analysis plan will be generated on commencement of the study to address the requirements of the DSMB as outlined in the DSMB Charter.

Analysis (All patients)
The statistical analysis will be mainly descriptive. The estimates of the incidence of adverse events will be based on crude rates. All estimates will be complemented with appropriate 95% confidence intervals.

Adverse events will be coded in accordance with standard medical dictionary terminology (MedDRA) before database lock.

Incidences will be computed on the following classes of AEs:

- All AEs
- Serious AEs
- AEs of special interest (AESIs)
- AEs which caused early discontinuation of *Pyramax*
- AEs related to *Pyramax* treatment

Specific analyses will be carried-out on the AESIs. Correlation analyses will be carried-out in order to verify if their occurrence is more frequent with the previous/concomitant use of other drugs (specific classes to be determined in the statistical analysis plan), including antimalarials, in the presence of a co-morbidity (specific classes to be determined in the statistical analysis plan), or in presence of parasitemia.

Compliance will be computed for each patient as the ratio between the number of tablets or sachets actually taken and the number of tablets or sachets that the patient should have taken. Two groups of patients shall be described: compliant = 100% treatment compliance and non-compliant = other cases. The safety profile of compliant vs non-compliant patients will be compared.

9.2 Determination of sample size

The sample size computation for the number of patients from the general malaria population who will present with AST or ALT >2xULN is based on a background incidence of 1.4% as seen in previous studies in a similar region. One hundred and twenty (120) malaria episodes in patients with AST or ALT >2xULN will need to be recruited to be able to detect one event with a probability of 81.6%. Severe hepatic event is defined as:

- Appearance of clinical signs and symptoms of possible hepatotoxicity associated with a rise in ALT/AST >2 x baseline value.

10. DATA MANAGEMENT

10.1 Collection and validation of data

Details of the data management processes will be documented in the Data Capture and Management Plan.

Screening data on all patients who present at the site will be captured on a specific screening registry (Section 7.2.1).

Patient data should be captured in the patient record at each site and documented in the study specific CRF.

Frequency of visit and responsibilities of the study monitor will be described in the Monitoring Plan. Source data verification (SDV) will be conducted on all informed consent forms, eligibility criteria, primary endpoint and AEs, SAEs and AESIs by the study monitor during monitoring visits.

Direct access to source documentation (medical records) must be allowed for the purpose of verifying that the data recorded in the eCRF are consistent with the original source data.

The study coordinator shall organise regular capture of these data by each study centre together with the results of the thick blood smears/RDTs and blood spots of those patients.

Interim data will be assessed under the DSMB Charter by the independent DSMB.

10.2 Quality control of data on site

The plan for source data verification will be described in the Data Capture and Management Plan.

10.3 Quality control of the preparation of thick blood smear slides

The sample preparation technique will be quality controlled by one of the trainers. This person shall visit each site during the course of the study to verify the microscopic slides.

These visits shall be frequent at the start of the study, then may decrease in frequency if the quality of the samples is satisfactory. In case anomalies are detected during these study visits, or during the examination of the samples, the concerned Health Agent shall receive additional training.

A provisional schedule or calendar of supervision visits shall be initiated at the end of the training phase.

Version 9.0 Date 15/03/2019: Property of SHIN POONG/MMV- strictly confidential

10.4 Quality control of thick blood smear sample reading

10% of the microscopic slides shall be quality controlled by a second qualified microscopist, who is independent of the centre where the initial microscopy took place. The procedures for quality control will be detailed in an ad hoc specification protocol.

10.5 Data entry

The Data Capture and Management Plan will describe the data entry processes and controls.

10.6 Data cleaning and data base locking

The Data Capture and Management Plan will describe the data cleaning and database locking procedures.

At the end of the study the centrally aggregated data, will be verified according to the above mentioned documented procedures. Once declared clean, the database will be locked before data analysis is performed.

11. TASKS AND RESPONSIBILITIES

11.1 Study specific DSMB

This study specific DSMB is an independent board governed by a specific charter which identifies the frequency of meetings and methodology of reporting to the EMA via Shin Poong Pharmacovigilance. The minutes of the Open Sessions of the DSMB are to be made available to the EMA.

This DSMB shall be in charge of the regular review (not more than every four months) of the listings of the adverse events collected during the study. The panel shall also receive the notification of serious adverse events, adverse events classified as severe and adverse events of special interest.

In case a signal is identified by the DSMB, the *Pyramax* QPPV shall be contacted as soon as possible.
In a more general manner, all decisions taken by this DSMB shall be documented in writing and shall be transmitted to the Sponsor for the project located in the Republic of Korea and attached to the final study report.

MMV and Shin Poong delegates may be co-opted as non-voting members to the DSMB.

11.1.1 Responsibilities of the study specific DSMB

This study specific DSMB is composed of scientific experts in the field of pharmacovigilance and/or tropical medicine, hepatology and of one statistician. If required, it shall be possible to consult *ad hoc* experts in other fields (haematology, cardiology, dermatology, etc.), who can join committee meetings.

The DSMB shall be involved in the approval of this protocol and its amendments, and be regularly updated on study progress, in particular on the number of inclusions. The DSMB shall

receive data and reports issued, as required by the DSMB Charter, and prepared by Shin Poong Pharmacovigilance, as well as notifications of serious adverse events. If clear signals of safety concerns are present, the DSMB can recommend halting the inclusion of further patients. The decision to halt the study shall be made after discussion and agreement between the members of the DSMB, Shin Poong and MMV. The *Pyramax* QPPV will be available at all times to discuss any safety findings in the context of the whole *Pyramax* safety database to aid in interpretation of any emerging safety signals.

The DSMB will have meetings or telephone conferences, prior to the start of the study to review the DSMB Charter, and then in accordance with the schedule provided in the Charter over the course of the study. At meetings the DSMB will review adverse events, consider and determine any possible safety signals, and discussion recommendations on the progress of the study. Finally, the DSMB shall meet for the release of the final results.

In between, conference calls can be organized at the request of the Sponsor, of an investigator, or of a member of one of the local pharmacovigilance monitoring committees.

All the decisions taken by the study specific DSMB shall be documented in writing; they will be transmitted to the Sponsor with the PIs of all the sites on copy and attached to the final study report. Any further local reporting will be in line with local regulations.

11.2 Responsibilities of the clinical staff of the health facilities

The staff shall be responsible to performing the study in accordance with this protocol and in accordance with the legislation and international guidelines under the direction of the local PI.

They are responsible for obtaining an informed consent prior to inclusion in the study, for completing the study documents (screening registry and patient CRF) and for recording all relevant data in relation to the study. Each staff member shall ensure that the information reported in the document is precise and accurate.

They must inform the patient of all relevant aspects of the study, including the information in the patient information sheet. All this information shall be provided to the patient in layman's terms. Patient confidentiality is paramount.

Prior to study inclusion, the informed consent form shall have to be personally completed (first name, surname), dated and signed by the patient, the patient's parent(s) or a guardian authorised representative. The person who has conveyed the information on the study to the patient shall also sign and date the informed consent form approved by the Ethics Committee. The informed consent forms will be translated into local languages for the benefit of those who do not understand English or French.

In case of patients unable to read and sign the patient information sheet and informed consent form, these documents will be read and explained to the patient in local language in the presence of a witness. The patient or the parent(s)/guardian in case of children below 18 years old, shall put her/his fingerprint on the informed consent form and the witness shall also sign the consent form to confirm that the patient has consented willingly.

A copy of the information sheet and the signed consent form shall be handed over to the patient or the parent/guardian.

11.3 Responsibilities of the Sponsor

The study Sponsor's responsibility is toward study teams at the study site and the health authorities and shall take all reasonable measures to ensure the good conduct of the study with regards to ethics, protocol compliance, integrity and validity of the information recorded in the patient CRF and registry as well as with regards to the availability of the adequate resources to ensure appropriate conduct of the study. In this respect, the principal function of the study management team is to help the investigator and the Sponsor to maintain a high level of ethical, scientific, technical and regulatory standards for all study-related aspects of ethics, regulations and administrative rules.

12. ETHICAL AND REGULATION ASPECTS

12.1 Regulations

The study shall be conducted in compliance with the text of the Declaration of Helsinki adopted by the 18th World Medical Association Assembly in 1964, and with its amendments (Annex 1).
The study will seek approval from the local institutional review boards / ethics committees (IRBs/ECs). This study will be undertaken only after the IEC/IRB has given full approval of the final protocol, amendments (if any), the informed consent form, applicable recruiting materials and the Sponsor has received a copy of this approval. This approval letter must be dated and must clearly identify the IEC/IRB and the documents and approved versions being approved. This study shall be conducted in accordance with the principles of the Good Clinical Practices (US, (4) & European, (5)).

The study shall be conducted in compliance with the international and national laws and regulations in effect, and in accordance with the applicable directives in Cameroon, Congo, DRC, Gabon and Ivory Coast in particular concerning the submission to the Ethics Committee and the protection of personal data.

Study related documents (protocol, case report from, informed consent form) shall be submitted to the National Ethics Committees or to the Institutions from the participating countries. Upon signature of the protocol, the Investigator accepts to respect the instructions and procedures described in the protocol, as well as the Good Clinical Practices and Good Laboratory Practices, to which he/she conforms.
The Investigator shall obtain from the patient or his/her legal representative, a signed (fingerprint and signature from a witness for patients unable to read and write), written consent. Assent will be obtained for minors able to understand the study procedures, according to the regulations in each of the participating countries. If informed consent is not obtained, the patient will not be enrolled. Furthermore, patients enrolled shall be entirely taken charge of for the treatment of their malaria for the duration of the study.

To ensure anonymity of study participants and confidentiality of the information collected, an identification number will be attributed to each participant at the time of study entry. Case report forms and diaries will be kept in a locked room by the Sponsor. Only medical doctors having taken part in the study and the Sponsor will have access to these documents.

Version 9.0 Date 15/03/2019: Property of SHIN POONG/MMV- strictly confidential

12.2 Informed consent

The investigator is responsible for ensuring that informed consent is obtained from each patient or legal representative and for obtaining the appropriate signatures and dates on the informed consent document prior to the performance of any protocol procedures and prior to the administration of study medication.

The Investigator shall explain to each study participant or his/her legal representative the nature of the study, its objective, the procedures involved, its risks and potential benefits and any discomfort it may generate. The patient must be informed that his/her participation is entirely voluntary, that he/she can withdraw from the study at any time and that withdrawal will not affect his/her subsequent medical treatment nor his relationship with the treating physician. The patient or his/her legal representative will sign on the informed consent sheet after having read and voluntarily agreed to it. A translation in local language for subjects unable to read and understand it will be available. Where the patient or his legal tutor are unable to read, in this case, an impartial witness should be present during the entire informed consent discussion. After inclusion, the patient may elect to withdraw from the study when he/she so wishes. The same level of attention will be dispensed to the patient.

12.3 Expected risks

Risks related to study medication

The study drug used in this study may cause headaches, nausea, vomiting, stomach ache, a slowing down of the heart rate (bradycardia), a decrease in the number of white blood cells, red blood cells (anemia) or blood platelets, an increase in the blood liver enzymes and a decrease in blood sugar levels (hypoglycemia).

These signs are generally moderate though they can vary from patient to patient. Risks that are currently unknown may exist.

Pyramax has not been studied in certain patient populations (for example in patients presenting with elevations in blood levels of liver enzymes). The uncertainty regarding the safety of use in such groups of patients is a foreseeable risk of participation in this study.

The safety of *Pyramax* in pregnant women has not been established. It is therefore not recommended in pregnant women. Women will be invited to inform the community health worker of any pregnancy occurring during the 2 months following the start of the *Pyramax* treatment. Pregnancy will be followed-up with visits at 3, 6 and 9 months and 7 days after delivery. On the last day of follow-up (usually Day 28 ±2 or in case of early termination, on the respective day), a urine pregnancy test will be performed for all women of child-bearing potential.

12.4 Data protection and confidentiality

The personal data of the patients which could be included in a Sponsor database or study database shall be treated in accordance with all local laws and regulations.

The investigator and the concerned personnel of the health centres and the study sites must keep all study documentation confidential and must take all necessary measures to prevent accidental or premature destruction of these documents.

The regulations or national laws in force on patient record keeping shall be applied.

At the time of archiving or management of the personal data pertaining to the nursing staff and/or patients, the Sponsor shall take all appropriate measures to secure and protect these data against access by a third non-authorised person.

12.5 Insurance

The Sponsor certifies to have subscribed for this study under its sponsorship, an insurance covering the responsibility of the investigator and his team, which is in agreement with the local laws and recommendations. The Sponsor's insurance shall not dismiss the investigator and his collaborators of their obligation to have their own civil liability insurance in line with the laws in force.

A copy of the insurance certificate shall be available for provision to investigators and / or ethics committees who would request it.

12.6 Premature termination of the study

The Sponsor can decide at any time and for whatever reason to prematurely suspend or interrupt the study. The decision and the justification shall be communicated in writing to the study specific DSMB.

The local authorities, EC and competent authorities shall have to be informed in line with local legislation.

The EMA will be advised of any decision and justification to prematurely terminate the study.

12.7 Competent authority inspections

Shin Poong and delegated responsible parties as well as the principal investigators accept to grant direct access to study source dossiers to auditors/inspectors for review, with the understanding that these people are bound by professional secrecy and shall not disclose any identity or medical information of a personal nature.

They shall undertake any effort in support of the audits and inspections by facilitating the access to equipment, data and necessary documents.

The confidentiality of the verified data and the protection of the patients shall be respected during these inspections.

All results and all information resulting from these inspections by the regulatory authorities shall be immediately communicated to the Sponsor.

Shin Poong and delegated responsible parties as well as the principal investigators shall take the appropriate measures in order to lead the corrective actions to all problems identified during the audits or inspections.

13. PROTOCOL AMENDMENTS

Each change to the protocol will be reported in a written amendment which will be signed by both the Principal Investigators and the Sponsor. The signed amendment will be added to the protocol.

Following the national legislation, the protocol amendment may require a regulatory submission (for example to the Ethics Committee) before implementation. Sometimes, an amendment may result in changes to the informed consent form. The Sponsor/Principal Investigators must receive an approval/favourable opinion from the Ethics Committee on the revised informed consent form before use.

14. DOCUMENTATION AND UTILISATION OF STUDY RESULTS

14.1 Properties and use of the study data and results

All results, data, documents and inventions obtained, directly or indirectly, from the trial, will be owned by the Sponsor unless a law or local regulation states otherwise. The Sponsor can use or exploit all results for their own use without any limitation of it's industrial property (territory, area, duration) in consultation with the study centres. The full data base will be the property of Shin Poong and will be utilized for producing the final study report as well as a safety report to be submitted to the European Medicine Agency. The investigators will have the right to participate with Shin Poong and MMV in the publication of such results.

14.2 Publications

A scientific committee will be formed, comprising the Sponsor, MMV and Coordinating Principal Investigator and others as agreed by these parties, with the responsibility for the presentations and/or publications of the results. The results of the study will be submitted to the Committee before each publication. Each subsequent presentation or publication should be approved by the scientific board.

The final decision on the publication of a manuscript/summary/presentation will be taken by the scientific committee after notification of the Sponsor in order to allow for an internal review and the possibility of providing comments. Each manuscript, summary, presentation will be submitted to the Shin Poong and MMV for internal review and possible comments at least 45 days before the submission to the journal and at least 20 days before the submssion of the summary. The Sponsor may request that their name and/or the name of one of their employees is present or not present on the publication. The Sponsor may delay each publication or communication during a limited time frame in order to protect the confidentiality or the proprietary information present in the document.

Version 9.0 Date 15/03/2019: Property of SHIN POONG/MMV- strictly confidential

15. REFERENCES

1. World Health Organisation: World Malaria Report 2014 [http://www.who.int/malaria/publications/world_malaria_report_2014/en/].

2. World Health Organisation: Guidelines for the treatment of malaria (third edition), 2015 [http://www.who.int/malaria/publications/atoz/9789241549127/en/].

3. World Health Organisation: WHO List of Prequalified Medicinal Products [http://apps.who.int/prequal/query/ProductRegistry.aspx?list=ma].

4. World Health Organisation: A practical handbook on pharmacovigilance of antimalarial medicines, WHO/PSM/QSM/2007.8. [http://apps.who.int/medicinedocs/documents/s16881e/s16881e.pdf].

5. World Health Organization: A Practical Handbook, Third Edition, 2012. Management of Severe Malaria [http://apps.who.int/medicinedocs/documents/s20170en/s20170en.pdf].

6. Committee for Medicinal Products for Human Use (CHMP). Summary of opinion: Pyramax (pyronaridine tetraphosphate / artesunate) 2012. [http://www.ema.europa.eu/docs/en_GB/document_library/Other/2012/02/WC500122945.pdf].

7. Investigator Brochure PYRAMAX Pyronaridine Artesunate. Edition 8, January 2016.

8. Temple R. Hepatotoxicity Through the Years: Impact on the FDA, presented 2/12/2001 [http:www.fda.gov/downloads/Drugs/ScienceResearch/ResearchAreas/ucm122149.pdf].

9. Reuben A. Hy's Law. Hepatology. 2004; 39:574-8.

10. Croft SL, Duparc S, Arbe-Barnes SJ, et al. Review of pyronaridine anti-malarial properties and product characteristics. Malar J 2012; 11: 270.

11. Ramharter M, Kurth F, Schreier AC, Nemeth J, Glasenapp I, Bélard S, Schlie M, Kammer J, Koumba PK, Cisse B, Mordmüller B, Lell B, Issifou S, Oeuvray C, Fleckenstein L, Kremsner PG. Fixed-dose pyronaridine-artesunate combination for treatment of uncomplicated falciparum malaria in pediatric patients in Gabon. J Infect Dis. 2008 Sep 15;198(6):911-9.

12. Tshefu AK, Gaye O, Kayentao K, Thompson R, Bhatt KM, Sesay SS, Bustos DG, Tjitra E, Bedu-Addo G, Borghini-Fuhrer I, Duparc S, Shin CS, Fleckenstein L; Pyronaridine-artesunate Study Team. Efficacy and safety of a fixed-dose oral combination of pyronaridine-artesunate compared with artemether-lumefantrine in children and adults with uncomplicated *Plasmodium falciparum* malaria: a randomised non-inferiority trial. Lancet. 2010 Apr 24;375(9724):1457-67.

Version 9.0 Date 15/03/2019: Property of SHIN POONG/MMV- strictly confidential

13. Kayentao K, Doumbo OK, Penali LK, et al. Pyronaridine-artesunate granules versus artemether-lumefantrine crushed tablets in children with *Plasmodium falciparum* malaria: a randomized controlled trial. Malar J 2012; 11: 364.

14. Poravuth Y, Socheat D, Rueangweerayut R, et al. Pyronaridine-artesunate versus chloroquine in patients with acute *Plasmodium vivax* malaria: a randomized, double-blind, non-inferiority trial. PLoS One 2011; 6(1).

15. Rueangweerayut R, Phyo AP, Uthaisin C, et al. Pyronaridine-artesunate versus mefloquine plus artesunate for malaria. N Engl J Med 2012; 366(14): 1298-309.

16. Duparc S, Borghini-Fuhrer I, Craft CJ, et al. Safety and efficacy of pyronaridine-artesunate in uncomplicated acute malaria: an integrated analysis of individual patient data from six randomized clinical trials. Malar J 2013; 12: 70.

17. Sagara I, Beavogui AH, Zongo I, Soulama I, Borghini-Fuhrer I, Fofana B, C Daouda, Somé AF, Coulibaly AS, Traore OB, Dara N, Kabore MJT, Thera I, Compaore YD, Sylla MM, Nikiema F, Diallo MS, Dicko A, Gil JP, Borrmann S, Duparc S, Miller RM, Doumbo OK, Shin J, Bjorkman A, Ouedraogo JB, Sirima SB and Djimdé AA. Safety and efficacy of retreatments with pyronaridine-artesunate in African malaria patients. Lancet Infect Dis 2016; 16: 189-198.

18. Anchang-Kimbi JK, Achidi EA, Apinjoh TO, Mugri RN, Chi HF, Tata RB, Nkegoum B, Mendimi JM, Sverremark-Ekström E, Troye-Blomberg M. (2014). Antenatal care visit attendance, intermittent preventive treatment during pregnancy (IPTp) and malaria parasitaemia at delivery. Malaria Journal, 13(1): 162. doi: 10.1186/1475-2875-13-162.

19. Tobias O. Apinjoh, Judith K. Anchang-Kimbi, Regina N. Mugri, Clarisse Njua-Yafi, Rolland B. Tata, Hanesh F. Chi, Delphine A. Tangoh, Beatrice T. Loh and Eric A. Achidi (2015) Determinants of Infant Susceptibility to Malaria During the First Year of Life in South Western Cameroon. Open Forum Infectious Diseases, DOI: 10.1093/ofid/ofv012

20. Tobias O Apinjoh, Judith K Anchang-Kimbi, Regina N Mugri, Robert N Vuchu, Delphine A Tangoh, Hanesh F Chi, Rolland B Tata, Charles Njumkeng, Clarisse Njua-Yafi and Eric A Achidi. (2015). The effect of Insecticide Treated Nets (ITNs) on Plasmodium falciparum infection in rural and semi-urban communities in the South West Region of Cameroon. Plos One. 10(2):e0116300.

21. Mathieu Ndounga, Pembe Mayengue Issamou, Prisca Nadine Casimiro, Félix Koukouikila-Koussounda, Michel Bitemo, Brunelle Diassivy Matondo, Lee Aymar Ndounga Diakou, Leonardo K Basco, Francine Ntoumi. Amodiaquine-artesunate versus artemether-lumefantrine for the treatment of acute uncomplicated malaria in Congolese children under 10 years old living in a suburban area: a randomized study. Malaria Journal (In Press).

22. Francine Ntoumi, Jeannhey C.Vouvoungui, Rod Ibara, Miguel Landry, Anissa Sidibé. 2013. Malaria burden and case management in the Republic of Congo: Limited use

and application of Rapid Diagnostic Tests results. BMC Public Health. Feb 14;13(1):135.

23. Zeukeng F, Tchinda VHM, Bigoga JD, Seumen CHT, Ndzi ES, Abonweh G, et al. (2014) Co-infections of Malaria and Geohelminthiasis in Two Rural Communities of Nkassomo and Vian in the Mfou Health District, Cameroon. PLoS Negl Trop Dis 8(10)

24. Tchinda GG, Atashili J, Achidi EA, Kamga HL, Njunda AL, Ndumbe PM (2012) Impact of Malaria on Hematological Parameters in People Living with HIV/AIDS Attending the Laquintinie Hospital in Douala, Cameroon. PLoS ONE 7

25. Malaria Atlas Project 2015 http://www.map.ox.ac.uk/

26. Henry M-C, C. Rogier, I. Nzeyimana, et al., Trop. Med. Int. Health 2003. Inland Valley rice production systems and malaria infection and disease in the Savannah of Côte d'Ivoire, TM&IH, Volume 8, Issue 5, May 2003, Pages 449–458

27. Ministère de la Santé et de l'Hygiène Publique. Institut Pierre Richet / Institut National de Santé Publique. 2009. Rapport sur le Profil entomologique du paludisme en Côte d'Ivoire (1956-2009). PP67.

16. ANNEXES

Annex 1: Declaration of Helsinki
Annex 2: Definition of Severe Malaria
Annex 3: Guidance for gradation of clinical symptoms
Annex 4: Adverse Events of Special Interests: Drugs and the Liver

ANNEX 1

Declaration of Helsinki

WORLD MEDICAL ASSOCIATION DECLARATION OF HELSINKI
Ethical Principles for Medical Research Involving Human Subjects

Adopted by the 18th WMA General Assembly, Helsinki, Finland, June 1964, and amended by the:
29th WMA General Assembly, Tokyo, Japan, October 1975
35th WMA General Assembly, Venice, Italy, October 1983
41st WMA General Assembly, Hong Kong, September 1989
48th WMA General Assembly, Somerset West, Republic of South Africa, October 1996
52nd WMA General Assembly, Edinburgh, Scotland, October 2000
53rd WMA General Assembly, Washington 2002 (Note of Clarification on paragraph 29 added)
55th WMA General Assembly, Tokyo 2004 (Note of Clarification on Paragraph 30 added)
59th WMA General Assembly, Seoul, October 2008

A. INTRODUCTION

1. The World Medical Association (WMA) has developed the Declaration of Helsinki as a statement of ethical principles for medical research involving human subjects, including research on identifiable human material and data.

   The Declaration is intended to be read as a whole and each of its constituent paragraphs should not be applied without consideration of all other relevant paragraphs.

2. Although the Declaration is addressed primarily to physicians, the WMA encourages other participants in medical research involving human subjects to adopt these principles.

3. It is the duty of the physician to promote and safeguard the health of patients, including those who are involved in medical research. The physician's knowledge and conscience are dedicated to the fulfilment of this duty.

4. The Declaration of Geneva of the WMA binds the physician with the words, “The health of my patient will be my first consideration,” and the International Code of Medical Ethics declares that, “A physician shall act in the patient's best interest when providing medical care.”

5. Medical progress is based on research that ultimately must include studies involving human subjects. Populations that are underrepresented in medical research should be provided appropriate access to participation in research.

6. In medical research involving human subjects, the well-being of the individual research subject must take precedence over all other interests.

7. The primary purpose of medical research involving human subjects is to understand the causes, development and effects of diseases and improve preventive, diagnostic and therapeutic interventions (methods, procedures and treatments). Even the best current interventions must be evaluated continually through research for their safety, effectiveness, efficiency, accessibility and quality.

8. In medical practice and in medical research, most interventions involve risks and burdens.

9. Medical research is subject to ethical standards that promote respect for all human subjects and protect their health and rights. Some research populations are particularly vulnerable and need special protection. These include those who cannot give or refuse consent for themselves and those who may be vulnerable to coercion or undue influence.

10. Physicians should consider the ethical, legal and regulatory norms and standards for research involving human subjects in their own countries as well as applicable international norms and standards. No national or international ethical, legal or regulatory requirement should reduce or eliminate any of the protections for research subjects set forth in this Declaration.

B. PRINCIPLES FOR ALL MEDICAL RESEARCH

11. It is the duty of physicians who participate in medical research to protect the life, health, dignity, integrity, right to self-determination, privacy, and confidentiality of personal information of research subjects.

12. Medical research involving human subjects must conform to generally accepted scientific principles, be based on a thorough knowledge of the scientific literature, other relevant sources of information, and adequate laboratory and, as appropriate, animal experimentation. The welfare of animals used for research must be respected.

13. Appropriate caution must be exercised in the conduct of medical research that may harm the environment.

14. The design and performance of each research study involving human subjects must be clearly described in a research protocol. The protocol should contain a statement of the ethical considerations involved and should indicate how the principles in this Declaration have been addressed. The protocol should include information regarding funding, sponsors, institutional affiliations, other potential conflicts of interest, incentives for subjects and provisions for treating and/or compensating subjects who are harmed as a consequence of participation in the research study. The protocol should describe arrangements for post-study access by study subjects to interventions identified as beneficial in the study or access to other appropriate care or benefits.

15. The research protocol must be submitted for consideration, comment, guidance and approval to a research ethics committee before the study begins. This committee must be independent of the researcher, the Sponsor and any other undue influence. It must take into consideration the laws and regulations of the country or countries in which the research is to be performed as well as applicable international norms and standards but these must not be allowed to reduce or eliminate any of the protections for research subjects set forth in this Declaration. The committee must have the right to monitor ongoing studies. The researcher must provide monitoring information to the committee, especially information about any serious adverse events. No change to the protocol may be made without consideration and approval by the committee.

16. Medical research involving human subjects must be conducted only by individuals with the appropriate scientific training and qualifications. Research on patients or healthy volunteers requires the supervision of a competent and appropriately qualified physician or other health care professional. The responsibility for the protection of research subjects must always rest with the physician or other health care professional and never the research subjects, even though they have given consent.

17. Medical research involving a disadvantaged or vulnerable population or community is only justified if the research is responsive to the health needs and priorities of this population or community and if there is a reasonable likelihood that this population or community stands to benefit from the results of the research.

18. Every medical research study involving human subjects must be preceded by careful assessment of predictable risks and burdens to the individuals and communities involved in the research in comparison with foreseeable benefits to them and to other individuals or communities affected by the condition under investigation.

19. Every clinical trial must be registered in a publicly accessible database before recruitment of the first subject.

20. Physicians may not participate in a research study involving human subjects unless they are confident that the risks involved have been adequately assessed and can be satisfactorily managed. Physicians must immediately stop a study when the risks are found to outweigh the potential benefits or when there is conclusive proof of positive and beneficial results.

21. Medical research involving human subjects may only be conducted if the importance of the objective outweighs the inherent risks and burdens to the research subjects.

22. Participation by competent individuals as subjects in medical research must be voluntary. Although it may be appropriate to consult family members or community leaders, no competent individual may be enrolled in a research study unless he or she freely agrees.

23. Every precaution must be taken to protect the privacy of research subjects and the confidentiality of their personal information and to minimize the impact of the study on their physical, mental and social integrity.

24. In medical research involving competent human subjects, each potential subject must be adequately informed of the aims, methods, sources of funding, any possible conflicts of interest, institutional affiliations of the researcher, the anticipated benefits and potential risks of the study and the discomfort it may entail, and any other relevant aspects of the study. The potential subject must be informed of the right to refuse to participate in the study or to withdraw consent to participate at any time without reprisal. Special attention should be given to the specific information needs of individual potential subjects as well as to the methods used to deliver the information. After ensuring that the potential subject has understood the information, the physician or another appropriately qualified individual must then seek the potential subject's freely-given informed consent, preferably in writing. If the consent cannot be expressed in writing, the non-written consent must be formally documented and witnessed.

25. For medical research using identifiable human material or data, physicians must normally seek consent for the collection, analysis, storage and/or reuse. There may be situations where consent would be impossible or impractical to obtain for such research or would pose a threat to the validity of the research. In such situations the research may be done only after consideration and approval of a research ethics committee.

26. When seeking informed consent for participation in a research study the physician should be particularly cautious if the potential subject is in a dependent relationship with the physician or may consent under duress. In such situations the informed consent should be sought by an appropriately qualified individual who is completely independent of this relationship.

27. For a potential research subject who is incompetent, the physician must seek informed consent from the legally authorized representative. These individuals must not be included in a research study that has no likelihood of benefit for them unless it is intended to promote the health of the population represented by the potential subject, the research cannot instead be performed with competent persons, and the research entails only minimal risk and minimal burden.

28. When a potential research subject who is deemed incompetent is able to give assent to decisions about participation in research, the physician must seek that assent in addition to the consent of the legally authorized representative. The potential subject's dissent should be respected.

29. Research involving subjects who are physically or mentally incapable of giving consent, for example, unconscious patients, may be done only if the physical or mental condition that prevents giving informed consent is a necessary characteristic of the research population. In such circumstances the physician should seek informed consent from the legally authorized representative. If no such representative is available and if the research cannot be delayed, the study may proceed without informed consent provided that the specific reasons for involving subjects with a condition that renders them unable to give informed consent have been stated in the research protocol and the study has been approved by a research ethics committee. Consent to remain in the research should be obtained as soon as possible from the subject or a legally authorized representative.

30. Authors, editors and publishers all have ethical obligations with regard to the publication of the results of research. Authors have a duty to make publicly available the results of their research on human subjects and are accountable for the completeness and accuracy of their reports. They should adhere to accepted guidelines for ethical reporting. Negative and inconclusive as well as positive results should be published or otherwise made publicly available. Sources of funding, institutional affiliations and conflicts of interest should be declared in the publication. Reports of research not in accordance with the principles of this Declaration should not be accepted for publication.

C. ADDITIONAL PRINCIPLES FOR MEDICAL RESEARCH COMBINED WITH MEDICAL CARE

31. The physician may combine medical research with medical care only to the extent that the research is justified by its potential preventive, diagnostic or therapeutic value and if the physician has good reason to believe that participation in the research study will not adversely affect the health of the patients who serve as research subjects.

32. The benefits, risks, burdens and effectiveness of a new intervention must be tested against those of the best current proven intervention, except in the following circumstances:
    - The use of placebo, or no treatment, is acceptable in studies where no current proven intervention exists; or
    - Where for compelling and scientifically sound methodological reasons the use of placebo is necessary to determine the efficacy or safety of an intervention and the patients who receive placebo or no treatment will not be subject to any risk of serious or irreversible harm. Extreme care must be taken to avoid abuse of this option.

33. At the conclusion of the study, patients entered into the study are entitled to be informed about the outcome of the study and to share any benefits that result from it, for example, access to interventions identified as beneficial in the study or to other appropriate care or benefits.

34. The physician must fully inform the patient which aspects of the care are related to the research. The refusal of a patient to participate in a study or the patient's decision to withdraw from the study must never interfere with the patient-physician relationship.

35. In the treatment of a patient, where proven interventions do not exist or have been ineffective, the physician, after seeking expert advice, with informed consent from the patient or a legally authorized representative, may use an unproven intervention if in the physician's judgement it offers hope of saving life, re-establishing health or alleviating suffering. Where possible, this intervention should be made the object of research, designed to evaluate its safety and efficacy. In all cases, new information should be recorded and, where appropriate, made publicly available.

ANNEX 2

Definition of Severe Malaria

WORLD HEALTH ORGANISATION CRITERIA 2014

Clinical features of severe malaria in children in high transmission area:

Group 1	Prostrate children (prostration is the inability to sit upright in a child normally able to do so or to drink in the case of children too young to sit). Three subgroups of increasing severity should be distinguished:<br>Prostrate but fully conscious<br>Prostrate with impaired consciousness but not in deep coma<br>Coma (the inability to localise a painful stimulus)<br>Respiratory distress (acidotic breathing):<br>Mild – sustained nasal flaring and/or mild intercostal indrawing (recession)<br>Severe – the presence of either marked indrawing (recession) of the bony structure of the lower chest wall or deep (acidotic) breathing<br>Shock compensated or decompensated (see definition above)
Group 2	Children who, although able to be treated with oral antimalarials, require supervised management because of the risk of clinical deterioration but who show none of the features of group 1 (above)*. These include children with any of the following:<br>Haemoglobin <5 g/dl or haematocrit < 15%<br>2 or more convulsions within a 24-h period<br>Haemoglobinuria (blackwater)<br>Jaundice
Group 3	Children who require parenteral treatment because of persistent vomiting but who lack any specific clinical or laboratory features of groups 1 or 2 (above)

*If parasite counts are immediately available, a parasitaemia over 10% should be included in group 2.
Children are defined as <12 years old.

Clinical features of severe malaria in adults:

Group 1	Adults at increased risk of dying immediately who require parenteral antimalarials and appropriate supportive therapy<br>Prostrated or obtunded adults (prostration is the inability to sit or to drink). Four subgroups of increasing severity should be distinguished:<br>Prostrate but fully conscious<br>Prostrate with impaired consciousness but not in deep coma (GCS > 11)<br>Confusion and agitation (GCS > 11)<br>Coma (the inability to localise a painful stimulus) (GCS < 11)<br>Respiratory distress (acidotic breathing)<br>Mild – sustained nasal flaring and/or mild intercostal indrawing (recession)<br>Severe – the presence of either marked indrawing (recession) of the bony structure of the lower chest wall or deep (acidotic) breathing<br>Shock (hypotension:systolic BP < 80 mmHg)<br>Anuria<br>Significant upper gastrointestinal haemorrhage
Group 2	Adults who, although able to be treated with oral ACTs, require supervised management because of the risk of clinical deterioration but who show none of the features of group 1 (above)*. This group includes adults with any of the following:<br>Haemoglobin <7 g/dl or haematocrit <20%<br>One or more convulsions within a 24-h period<br>Haemoglobinuria (blackwater)<br>Jaundice
Group 3	Adults who require parenteral treatment because of persistent vomiting but who lack any specific clinical or laboratory features of groups 1 or 2 (above)

*If parasite counts are immediately available a parasitaemia over 4% should be included in group 2.

ANNEX 3

Guidance for the evaluation of the intensity of clinical signs

	Grade 1	Grade 2	Grade 3	Grade 4

	MILD	MODERATE	SEVERE	LIFE-THREATENING
**Fever in the following 24h**	N/A	Yes	N/A	N/A
**Weakness**	Small decrease in activity, keeps playing	Moderate decrease in activity, has difficulty to play	No activities, stopped playing	Lethargy
**Muscular or articular pain***	Localised pain, weak intensity	Diffuse pain, weak intensity	Real weakness; limited functions	N/A
**Cephalgia***	Weak, not requiring treatment	Fluctuating, moderate, requiring treatment	Severe, responding to an initial narcotic treatment	Refractory, requiring a repeated narcotic treatment
**Anorexia**	Reduced appetite but still eating solid foods	Reduced appetite, avoiding all solid foods	Refusing breast milk, very reduced appetite, taking neither liquids nor solids (< 2 years ≤ 12 h; > 2 years ≤ 24 h)	Refusing breast milk, very reduced appetite, taking neither liquids nor solids (< 2 years ≤ 12 h; > 2 years ≤ 24 h)
**Nausea***	Small discomfort; continues to ingest normally	Moderate discomfort; ingestion significantly reduced; certain activities are limited	Severe discomfort; no significant ingestion, limited activities	Minimal ingestion of liquids
**Vomiting**	Transient vomiting	Moderate or occasional vomiting	Orthostatic hypotension requiring an infusion	Shock for which hospitalisation for infusion is required
**Abdominal pain***	Weak	Moderate, no treatment required	Moderate to severe – treatment required	Severe – hospitalisation for treatment
**Diarrhea**	Transient; 3-4 liquid stools /day	5–7 liquid stools /day	Orthostatic hypotension or >7 liquid stools /day or infusion required	Shock for which hospitalisation for infusion is required
**Cough**	Transient, no treatment required	Continuous, requiring a treatment	Irrepressible	Cyanosis, violent cough, very difficult breathing
**Pruritus**	Pruritus without rash	Pruritus with rash or Pruritus without eruptions disturbing the sleep	Moderate urticaria	Severe urticaria, anaphylaxis, Quincke's Oedema
**Tinnitus***	Weak	Moderate	Severe including hearing loss	N/A
**Behavioural changes**	Minor concentration difficulties; confusion or minor agitation, normal daily activities; no treatment required	Moderate confusion or agitation; daily activities slightly impacted; minimal treatment required	Severe confusion or agitation; requiring assistance with daily activities; treatment required	Toxic psychosis; hospitalisation required
**Flu symptoms**	Minor nasal congestion, minor rhinitis, without cough	Moderate nasal congestion, moderate rhinitis, with cough	N/A (if severe, classify the symptoms individually)	N/A (in life-threatening circumstances, classify the symptoms individually)
*** Applicable only to children ≥ 3 ans. Respond N/A if younger or for those not able to respond.**				

Reference – Based upon the WHO toxicity grading scale for determining the severity of adverse events

	Grade 1 MILD	Grade 2 MODERATE	Grade 3 SEVERE
**Convulsion**	N/A	N/A	Localised or general
**Temperature* (tympanic)**	38.0-38.4°C	38,5-40.0°C	> 40.0°C

**Dehydration** **	Normal skin to touch, hydrated mucosa, tears are present, normal eyes, flat fontanel, consolable, regular pulse, normal micturition	Dry skin, dry mucosa, sunken eyes, lack of tears, soft fontanel, irritable, slightly accelerated pulse, reduced micturition	Moist and cold skin, dried skin, completely sunken eyes, no tears, sunken fontanel, lethargic, rapid pulse, no micturition	
**Facial oedema**	Present, minor swelling of the eyes	Moderate swelling of the eyes and the face	Severe swelling of the eyes, the face and the mucosa – impossible to open the eyes	The respiratory system is affected
**Icterus**	Mild subconjunctival icterus	Moderate subconjunctival icterus, mucosa moderately yellow	Severe subconjunctival icterus and icterus of the skin	N/A
**Thorax**	Slightly accelerated breathing (with regard to age and temperature), evanescent or localised rhonci	Moderately accelerated breathing, diffuse or persistent rhonchi	Rapid breathing (< 2 months > 60, 2-12 months > 50, 1-5 years > 40, adults > 30)* dilatation and retraction of the nostrils	Cyanosis
**Abdomen**	Normal abdominal sounds, slight local sensibility and/or hepatomegaly exceeding the costal margin with 2-4 cm and/or palpable spleen and/or presence of umbilical hernia	Slightly abnormal abdominal signs or moderate or diffuse sensibility and/or mild or moderate hepatomegaly (exceeding the costal margin with 4-6 cm) and/or grade 4 splenomegaly (palpable spleen until midway between the navel and the public symphysis	Very abnormal abdominal sounds, pain and resistance during palpation and/or hepatomegaly exceeding the costal margin > 6 cm and/or splenomegaly grade 5 (palpable spleen at beyond midway between the navel and the public symphysis)	No abdominal sound. Contracture
**Skin†**	Localised skin eruption, erythema or pruritus	Desquamation, diffuse maculopapular eruption	Vesicles, moist desquamation or ulceration	Exfoliative dermatitis, implication of mucosa or multiform erythema or suspicion of Stevens Johnson or a necrosis requiring a surgical intervention

	**Grade 4** **LIFE-THREATENIING**
tion ty ' the	N/A

col Amendment Number 2 - Approval and Authorisation

ol Number: SP-C-021-15

[redacted]

**Attempt to pick-up tablets**	Difficulty in grasping the tablet though capable	Cannot grasp the tablet without dropping the tablet	Cannot grasp the tablet	N/A
**Other symptoms/ signs (not described elsewhere)**	No treatment; only monitoring	May require a minimal intervention and monitoring	Requires medical care and possible hospitalisation	Requires active medical care or hospitalisation

***Reference – DMID Toxicity Table for Children, May 2001, drug fever (rectal)**
**** Reference - The Harriet Lane Handbook, 15th edition, 2000**
† Reference - WHO toxicity grading scale for determining the severity of adverse events

ANNEX 4

Adverse Events of Special Interests: Drugs and the Liver

Checklist for Serious Liver Reactions:

The following liver reactions have to be considered as potentially serious:

Possible Hy's law case is defined as a subject with any value of ALT or AST >3xULN together WITH an increase in bilirubin to a value > 2xULN (>35% direct) and NOT associated to an ALP value > 2xULN.

Other definitions of hepatic adverse events of special interest (AESIs):

For patients with normal LFTs at baseline:

- Present with fatigue, nausea, abdominal pain itching or signs of jaundice such as:
    - o dark urine,
    - o putty or mastic coloured stools,
    - o jaundice (yellowing of the whites of the eyes or skin),

and

- ALT or AST >3 x ULN.

For patients with baseline ALT/AST >2xULN:

- Present with fatigue, nausea, abdominal pain itching or signs of jaundice such as:
    - o dark urine,
    - o putty or mastic coloured stools,
    - o jaundice (yellowing of the whites of the eyes or skin),

and

- ALT/AST >2 x baseline value.

Procedures to follow if possible Hy's law criteria are met:

- Stop the study medication and concomitant medications if they are not medically necessary
- Report the event as an SAE.
- Alert the Sponsor (via the study Medical Monitor or the Pharmacovigilance (Shin Poong QPPV) contacts) immediately.
- All efforts must be done to obtain a hepatologist consultation.
- In addition to the hepatitis panel, obtain samples for:
    - o quantitative hepatitis B DNA and hepatitis delta antibody (if positive for hepatitis B surface antigen at screening),
    - o serum acetaminophen adduct HPLC assay,
    - o antinuclear antibody, anti-smooth muscle antibody, Type 1 anti-liver kidney microsomal antibodies and quantitative IgG or gamma-globulin.
- Follow the instructions below for all events meeting any definition of liver biochemistry event of special concern.

Instructions in the event of any of the following: (i) a case of possible Hy's Law, or (ii) meeting definition of a biochemistry event of special interest

- Report the AE to the Sponsor, and as an SAE if the event is a possible Hy's law, or meets SAE criteria (via the study Medical Monitor and Shin Poong QPPV)
- Collect if not scheduled urine, blood, or relevant biological fluids for additional diagnostic tests
- Follow up of liver chemistries albumin and prothrombin time (+ prealbumin, serum ceruloplasmin, procollagen III peptide, α-1-antitrypsin and α-feto protein when possible) twice weekly or more frequently if considered medically indicated until values normalize or substantively improve.
- If medically indicated request liver imaging (ultrasound, computerized tomography or magnetic resonance imaging)
- If liver biopsy obtained, request full report be forwarded to the Sponsor
- Obtain additional consent for above if necessary
- Obtain if needed additional history and review of medical records
- Ask again for details, in particular any concomitant meds, OTC meds, herbal remedies, prior exposure, previous episodes, etc, and discuss them with Sponsor
- Obtain documentation for all protocol- required or non-protocol required laboratory values, ECG tracings, pathology reports until resolution
- Ensure appropriate medical attention, including move to hospital Emergency Department or ICU, specialist consultation, Liver Transplant Unit, etc, as deemed medically necessary
- Arrange appropriate follow up for investigations
- Sponsor, Investigator and Hepatology Consultant to review aggregate data from available sources for underlying trends
- Contact the Sponsor safety department (via Medical Monitor) or other internal and external experts for advice as needed

Documentation needed in the event of an elevation of special concern in one or more liver function tests

If laboratory values are returned that meet the above-described criteria of Serious Liver Reactions, the investigator should attempt to document the following in the investigator comment log of the CRF prior to contacting the Sponsor:

- The subject's age, gender and weight.
- The date (and visit number) on which the blood sample was obtained.
- The Randomization date as well as the dose level that the subject is currently taking, and the duration of exposure to that dose level. The exact date on which the subject took the last dose of the study medication and an objective assessment should be obtained of the subject's compliance with the study medication.

- The specific abnormal laboratory values, as well as those of each of the other LFTs noted above (regardless of value) and, when relevant, results of isoenzyme or fractionation analyses. The investigator should also document the corresponding laboratory values at screening/baseline.
- Other notable abnormalities in laboratory values (*e.g.,* complete blood count, eosinophilia, or electrolyte abnormalities, Serum transferrin saturation and serum ferritin if present).
- The dates and nature of any relevant adverse events (*e.g.,* jaundice) that occurred since Randomization, with particular attention to hypotension, fever, rash, hepatitis symptoms (e.g. appearance or worsening of fatigue, nausea, anorexia, nausea, emesis, abdominal pain), or other adverse events that might have occurred in close proximity to the elevation in the laboratory value(s) of interest.
- Any associated physical findings (including results of any exams or evaluations, including heart rate, blood pressure, temperature, and abdominal exam).
- The use of concomitant medications (*e.g.,* ARVs, paracetamol/acetaminophen, herbal products) since randomization, as well as the dates of exposure to the concomitant medication(s). Please include any nutritional supplements, vitamins and/or herbal preparations that the subject might have taken during this time frame.
- A statement concerning whether the subject has consumed alcohol since the time of Randomization, with a description of frequency and intensity, if relevant. A blood alcohol level should be obtained if the subject's history and/or clinical presentation suggest proximal use or intoxication with alcohol.
- Any history on the subject's part of prior elevations in any of the relevant laboratory values. Provide actual dates and laboratory values.
- Any history on the subject's part of a past or recent history of exposure to known factors that can cause, or are associated with, elevations in liver function tests. Examples of these factors include alcohol abuse and/or dependence, hepatitis (infectious or chemical), infectious mononucleosis, gallbladder disease, liver disease of any kind, jaundice, myocardial infarction, heart failure and/or episodes of hypotension.
- Any family history of hepatitis (from any cause) or hepatotoxicity from medications.

The investigator is asked to contact the Medical Monitor if there are any questions about the most appropriate course of action, and/or if there are questions as to whether the subject should be referred to a specialist for further evaluation.

It is anticipated that the investigator will follow any subject with clinically significant elevations of one or more liver function tests until there is clear evidence that the value(s) have stabilized and/or normalized. In addition, an explanation should be provided for any subjects that are lost to follow up.

CLINICAL STUDY PROTOCOL AMENDMENT NUMBER 2 - GENERAL

Sponsor:	Shin Poong Pharmaceuticals Co. Ltd
Protocol Number:	SP-C-021-15
Study Title:	PHASE IIIB/IV COHORT EVENT MONITORING STUDY TO EVALUATE, IN REAL LIFE SETTING, THE SAFETY AND TOLERABILITY IN MALARIA PATIENTS OF THE FIXED-DOSE ARTEMISININ-BASED COMBINATION THERAPY PYRAMAX (PYRONARIDINE-ARTESUNATE)

Refers to the Current Approved Protocol Version 7.0, 25 January 2017

Date: 13 September 2018

1 INTRODUCTION

The purpose of this amendment is to include in the current protocol:

1) Urinary pregnancy test at Day 28 ±2 or earlier in case of patient early termination, in order to increase the chance to detect early pregnancies that would have started just before or during the treatment period. A few numbers of pregnancies have been reported up to now. The inclusion of pregnancy test for all women of child-bearing potential at the end of the study could confirm this reality or could gather additional important information regarding the treatment of pregnant women in first trimester with PYRAMAX®.

In addition to the amendment of study procedures, following minor changes are included

1) Addresses were updated for specification and including new information.
2) Contact information for other responsible parties, including central laboratories, monitoring and electronic TMF vendor was included.
3) Clarification was added, to indicate the analysis of the full hepatitis panel at day 7, day 28 and unforeseen visits, if appropriate.
4) When the protocol Version 7.0 was designed, six months before the initiation, other sites were anticipated to take part of the study. Respective information was updated to reflect the sites that are currently active and enrol patients for the study.
5) In the context of the haematology, "minimum" was changed to "mandatory" to clarify that HB analysis is the one obligatory test, beside other tests, deemed necessary by the investigator.
6) The reporting and revision process for Adverse Events was refined.

Changes/additions to the text are marked in **yellow**, with addition in bold and deletion in strikethrough.

2 CHANGES MADE TO THE PROTOCOL BY THIS AMENDMENT

**Section Location**	Original Text (Final protocol version 7.0, dated 25 January 2017)	Amended Text (Amended protocol dated 13 September 2018)	Rationale
**NAMES AND ADDRESSES**<br>**Principal Investigators**<br>**(p. 2)**	Dr. Serge-Brice Assi, MD, PhD<br>[redacted]	Dr. Serge-Brice Assi, MD, PhD<br>[redacted]	Typo

	Dr Felix Koukouikila- Koussounda, PhD<br>[redacted]<br><br>Prof Gaston Tona Lutete<br>[redacted]	Dr Felix Koukouikila- Koussounda, PhD<br>[redacted]<br><br>Prof Gaston Tona Lutete<br>[redacted]	Addresses updated
**NAMES AND ADDRESSES**<br>**Other Responsible Parties**<br>**(p. 3)**		**Central Laboratory (Viral Hepatitis Assessment and Full Hepatitis Panel)**<br>**Covance Central Laboratory Services SA**<br>[redacted]<br><br>**Central Laboratory (Re-appearing parasitemia)**<br>**Swiss Tropical and Public Health Institute**<br>[redacted]<br><br>**Electronic Trial Master File**<br>**PhlexGlobal**<br>[redacted]	Other Responsible Parties added

**NAMES AND ADDRESSES**<br>**COORDINATING PRINCIPAL INVESTIGATOR**<br>**(p. 4)**	Assoc Prof Dr Michael Ramharter<br>[redacted]	~~Assoc~~ Prof Dr Michael Ramharter<br>[redacted]	Addresses, emails and phone number updated
**NAMES AND ADDRESSES**<br>**PROJECT COORDINATOR**<br>**(p. 4)**	Prof Francine NTOUMI, PhD<br>[redacted]	Prof Francine NTOUMI, PhD<br>**Fondation Congolaise pour la Recherche Médicale,** [redacted]	Addresses, emails and phone number updated
**NAMES AND ADDRESSES**<br>**Medical Monitor**<br>**(p. 4)**		**Selidji Todagbe Agnandji**<br>**CERMEL,** [redacted]	Name of Medical Monitor and contact details added
**Protocol Approval and Authorisation**<br>**(p. 5)**	Assoc. Prof. Dr. Michael Ramharter, MD	~~Assoc.~~ Prof. Dr. Michael Ramharter, MD	Updated
**1. SUMMARY**<br>**LIST OF**	BUN<br>CEM	~~BUN~~<br>~~CEM~~	Abbreviations deleted (not

**ABBREVATIONS**<br>**(p. 10-11)**	CEMREL<br>SP Sulfadoxine-Pyrimethamine	~~CEMREL~~**CERMEL**<br>SP Sulfadoxine-~~P~~**p**yrimethamine	included in the protocol), corrected
**2. SUMMARY**<br>**Design and duration of the study**<br>**(p. 13)**	………………..<br>The patients will be visited at home at Day 7 ±1 day, by a community health worker (CHW), in order to capture the adverse events (AEs) experienced. In the case of signs or symptoms of hepatotoxicity or hepatic related AEs, the patients will be referred to the health facilities and blood will be taken on this occasion for LFT assessment and haematology (Hb mandatory). If a patient cannot be directly reached, the CHW must organize an appointment with this patient before Day 10. Special procedures will be followed in case of serious adverse events (SAEs) and/or severe adverse events and events classified as being of special interest (AESI) (see specific section).<br><br>At Day 28 ±2 days, all patients will be seen by a CHW for a final assessment, including blood taken for thick blood smear and spot. In the case of signs or symptoms of hepatotoxicity or hepatic related AEs occurring since the previous Day 7 visit, the patient will be referred to the health facilities and blood will be taken for LFTs and haematology (Hb mandatory) assessment.<br><br>Female patients must communicate to their village CHW or the study team if they get pregnant just before or after the start of the *Pyramax* treatment and for 2 months post-treatment. All pregnancies must be documented on the manufacturer's *Pyramax* Pregnancy Register. In these cases, the evolution of the pregnancy will be monitored with visits at 3, 6	………………..<br>The patients will be visited at home at Day 7 ±1 day, by a community health worker (CHW), in order to capture the adverse events (AEs) experienced. In the case of signs or symptoms of hepatotoxicity or hepatic related AEs, the patients will be referred to the health facilities and blood will be taken on this occasion for LFT assessment, ~~and~~ haematology (Hb mandatory), **and where appropriate for full hepatitis panel assessment**. If a patient cannot be directly reached, the CHW must organize an appointment with this patient before Day 10. Special procedures will be followed in case of serious adverse events (SAEs) and/or severe adverse events and events classified as being of special interest (AESI) (see specific section).<br><br>At Day 28 ±2 days, all patients will be seen by a CHW for a final assessment, including blood taken for thick blood smear and spot. In the case of signs or symptoms of hepatotoxicity or hepatic related AEs occurring since the previous Day 7 visit, the patient will be referred to the health facilities and blood will be taken for LFT~~s~~ **assessment**, ~~and~~ haematology (Hb mandatory), **and where appropriate for full hepatitis panel** assessment.<br><br>Female patients must communicate to their village CHW or the study team if they get pregnant just before or after the start of the *Pyramax* treatment and for 2 months post-treatment. All pregnancies must be documented on the manufacturer's *Pyramax* Pregnancy Register. In these cases, the evolution of	Reworded for clarity<br><br>Reworded for clarity

	and 9 months and at 7 days after the delivery. Information on the drugs taken during the pregnancy as well as AEs/SAEs/AESIs and the health status of the newborn(s) will be collected. On the last day of follow-up (usually Day 28 ±2 days or in case of earlier withdrawal then on the respective day), a urine pregnancy test will be performed on all women of child bearing potential.	the pregnancy will be monitored with visits at 3, 6 and 9 months and at 7 days after the delivery. Information on the drugs taken during the pregnancy as well as AEs/SAEs/AESIs and the health status of the newborn(s) will be collected. On the last day of follow-up (usually Day 28 ±2 days or in case of earlier withdrawal then on the respective day), a urine pregnancy test will be performed on all women of child bearing potential. **On the last day of follow-up (usually Day 28 ±2 or in case of early termination, on the respective day), a urine pregnancy test will be performed for all women of child-bearing potential, defined as women who have experienced menarche and who are not permanently sterile or postmenopausal (defined as 12 consecutive months with no menses without an alternative medical cause). The investigator shall decide whether this is applicable or not based on the medical history of the patient, and on customs and practices of the region.**	Urinary pregnancy test added to increase the chance to detect early pregnancies
**STUDY FLOW CHART**<br>**(p. 18)**<br><br>**Flow chart footnote**	Hepatitis panel	**Full h**~~H~~epatitis panel<br>**Urine pregnancy test (at Day28 ±2 and Unforeseen Visit**<br><br>**∞ A urine pregnancy test will be performed for all women of child-bearing potential, on the last day of follow-up (usually Day 28 ±2 or in case of early termination, on the respective day), at home or at the health facility, if the patient is transferred to the health facility.**<br>**Women of child-bearing potential are defined as women who have experienced menarche and who are not permanently sterile or postmenopausal (defined as 12 consecutive months with no menses without an alternative medical cause). The investigator shall decide**	Typos<br><br>Urinary pregnancy test added to increase the chance to detect early pregnancies

		**whether this is applicable or not based on the medical history of the patient, and on customs and practices of the region.**	
**3.3 Malaria in Cameroon, Republic of Congo, DRC, Gabon and Ivory Coast**<br>**(p. 21-24)**	Cameroon – Study sites<br>The Principal site and Satellite sites are overseen by the Principal Investigator located at University of Yaoundé.<br>One satellite site is located at Etoug ebe, in the Mfoundi division, centre region of Cameroon (3°51'N and 11°31'E). It has a very particular relief consisting of hills and marshy valleys with average altitude of 760 m.<br>A further satellite site is based at Mfou District Hospital.<br>………………..<br>Republic of Congo – Study sites<br>The Principal site and Satellite sites are overseen by the Principal Investigator who is located at Centre de Santé FCRM Massisia. The satellite sites are Centre de Santé de Madibou, Brazzaville and Hôpital de Base de Makélékélé, Brazzaville.<br>………………..<br>Democratic Republic of Congo – Study sites<br>The Principal site and Satellite sites are overseen by the Principal Investigator who is located at Universite de Kinshasa (UoKPV) and the satellite sites are based at Institut Médicale Évangélique de Kimpesé (IMEK) in Kimpese (bas Congo), Centre Hospitalier Mont-Amba (CHMA) in Kinshasa and Maternite de Binza (MB) in Kinshasa Delvaux.<br>………………..	Cameroon – Study sites<br>The Principal site and Satellite sites are overseen by the Principal Investigator located at University of Yaoundé.<br>~~One satellite site is located at Etoug ebe, in the Mfoundi division, centre region of Cameroon (3°51'N and 11°31'E). It has a very particular relief consisting of hills and marshy valleys with average altitude of 760 m.~~<br>~~A further~~ **The** satellite site is based at Mfou District Hospital.<br>………………..<br>Republic of Congo – Study sites<br>The Principal site and ~~Satellite~~ **Referring** sites are overseen by the Principal Investigator who is located at Centre de Santé FCRM Massisia. ~~The satellite~~**Referring** sites are Centre de Santé de Madibou, Brazzaville and Hôpital de Base de Makélékélé, Brazzaville.<br>………………..<br>Democratic Republic of Congo – Study sites<br>The Principal site and Satellite sites are overseen by the Principal Investigator who is located at Universite de Kinshasa (UoKPV) and the satellite sites are based at Institut Médicale Évangélique de Kimpesé (IMEK) in Kimpese (bas Congo), **and** Centre Hospitalier Mont-Amba (CHMA) in Kinshasa. ~~and Maternite de Binza (MB) in Kinshasa Delvaux.~~<br>………………..	Deleted: site is not active the study<br><br>Site is referring patients to the principal site<br><br>Deleted: site is not active the study

	<u>Ivory Coast – Study sites</u><br>The Principal site and Satellite sites are overseen by the Principal Investigator who is located at Institute Pierre Richet (IPR) and the satellite sites : Agboville, Azaguié Health Center, Centre de Sante Urbain d'Azaguie (CSUA) and Centre de Sante Urbin de Grand Morie (CSUGM).<br>………………..<br>1) Cameroon: University of Yaoundé 1, Yaoundé and the satellite sites Mfou District Hospital and Etoug-Ebe Baptist Health Centre (EBHC).<br>2) Republic of Congo, Centre de santé FCRM – Massisia and the satellite sites: Centre de santé de Madibou , Brazzaville and Hôpital de base de Makélékélé, Brazzaville.<br>3) DRC : Université de Kinshasa (UoKPV) and the satellite sites Institut Médicale Évangélique de Kimpesé (IMEK) in Kimpesé (Bas Congo), Centre Hospitalier Mont-Amba (CHMA) in Kinshasa and Maternite de Binza (MB) in Kinshasa Delvaux.<br>4). Gabon, Albert Schweitzer Hospital at Lambaréné (CERMEL).<br>5) Ivory Coast, Institute Pierre Richet (IPR) and the satellite sites : Agboville, Azaguié Health Center, Centre de Sante Urbain d`Azaguie (CSUA) and Centre de Sante Urbin de Grand Morie (CSUGM).	<u>Ivory Coast – Study sites</u><br>The Principal site and Satellite sites are overseen by the Principal Investigator who is located at Institute Pierre Richet (IPR) and the satellite sites : Agboville, ~~Azaguié Health Center,~~ Centre de Santé Urbain d'Azaguié (CSUA) and Centre de Santé Urbain de Grand Morié (CSUGM).<br>………………..<br>1) Cameroon: University of Yaoundé 1, Yaoundé and the satellite site~~s~~ Mfou District Hospital~~. and Etoug-Ebe Baptist Health Centre (EBHC).~~<br>2) Republic of Congo, Centre de santé FCRM – Massisia and the ~~satellite~~**referring** sites: Centre de santé de Madibou , Brazzaville and Hôpital de base de Makélékélé, Brazzaville.<br>3) DRC : Universite de Kinshasa (UoKPV) and the satellite sites Institut Médicale Évangélique de Kimpesé (IMEK) in Kimpese (bas Congo), **and** Centre Hospitalier Mont-Amba (CHMA) in Kinshasa. ~~and Maternite de Binza (MB) in Kinshasa Delvaux.~~<br>4). Gabon, Albert Schweitzer Hospital at Lambaréné (CERMEL).<br>5) Ivory Coast, Institute Pierre Richet (IPR) and the satellite sites : Agboville, ~~Azaguié Health Center,~~ Centre de Santé Urbain d'Azaguié (CSUA) and Centre de Santé Urbain de Grand Morié (CSUGM).	Typos<br><br>Deleted: site is not active in the study<br>Site is referring patients to the principal site<br><br>Deleted: site is not active the study<br><br>Typos
**5. DESCRIPTION OF THE STUDY**<br>**5.1.1 Study assessments**<br>**(p. 25-26)**	Screening/Inclusion/Day 0:<br>Patients for whom a diagnosis of uncomplicated malaria (according to WHO criteria) is suspected, will undergo an RDT or microscopy. If the presence of malaria is confirmed and the patient meets all inclusion criteria and none of the exclusion criteria and the patient (or parent/ guardian in case of	Screening/Inclusion/Day 0:<br>Patients for whom a diagnosis of uncomplicated malaria (according to WHO criteria) is suspected, will undergo an RDT or microscopy. If the presence of malaria is confirmed and the patient meets all inclusion criteria and none of the exclusion criteria and the patient (or parent/ guardian in case of	

	children) provides his/her informed consent, he/she will be assigned a unique ID number and will be enrolled in the study. Blood will be taken for PCR analysis (blood spot), for retrospective LFTs (& Hb?) assessment as well as for a retrospective viral hepatitis assessment to be done only in those patients with abnormal baseline LFTs (AST or ALT >2 x ULN). A blood sample will be taken for Haematology (Hb mandatory).<br>………………..<br>Day 7 or any unforeseen visit<br>The patients will be visited at home at Day 7 ± 1 day, by a community health worker (CHW), in order to capture the adverse events (AEs) experienced. The patient's body temperature will be taken. In the case of signs or symptoms of hepatotoxicity or hepatic related AEs, the patients will be referred to the health facilities and blood will be taken on this occasion for LFTs and haematology (minimum Hb) assessment as well as for a full hepatitis panel. These investigations will be performed immediately. If a patient cannot be directly reached, the CHW will have to organise an appointment with this patient before Day 10. Special procedures will be followed in case of serious adverse events (SAEs) and/or severe adverse events and events classified as being of special interest (AESI) (see specific section).<br>………………..<br>Day 28<br>At Day 28 ±2 days, all patients will be seen by a CHW for a final assessment. At this occasion, blood will be taken for thick blood smear and spot. In the case of signs or symptoms of hepatotoxicity or hepatic related AEs occurring since the previous Day	children) provides his/her informed consent, he/she will be assigned a unique ID number and will be enrolled in the study. Blood will be taken for PCR analysis (blood spot), for retrospective LFT~~s (& Hb?)~~ assessment as well as for a retrospective viral hepatitis assessment to be done only in those patients with abnormal baseline LFTs (AST or ALT >2 x ULN). A blood sample will be taken for Haematology (Hb mandatory).<br>………………..<br>Day 7 or any unforeseen visit<br>The patients will be visited at home at Day 7 ± 1 day, by a community health worker (CHW), in order to capture the adverse events (AEs) experienced. The patient's body temperature will be taken. In the case of signs or symptoms of hepatotoxicity or hepatic related AEs, the patients will be referred to the health facilities and blood will be taken on this occasion for LFTs and haematology (~~minimum~~ Hb **mandatory**) assessment as well as for a full hepatitis panel. These investigations will be performed immediately. If a patient cannot be directly reached, the CHW will have to organise an appointment with this patient before Day 10. Special procedures will be followed in case of serious adverse events (SAEs) and/or severe adverse events and events classified as being of special interest (AESI) (see specific section).<br>………………..<br>Day 28<br>At Day 28 ±2 days, all patients will be seen by a CHW for a final assessment. At this occasion, blood will be taken for thick blood smear and spot. In the case of signs or symptoms of hepatotoxicity or hepatic related AEs occurring since the previous Day	Typo<br><br>Reworded for clarity

	7 visit, the patient will be referred to the health facilities and blood will be taken for LFTs and haematology (minimum Hb) assessment as well as for a full hepatitis panel.<br><br>Female patients must communicate to the local CHW or the study team if they get pregnant just before or after the start of the *Pyramax* treatment and for 2 months post-treatment. All pregnancies must be documented on the manufacturer's *Pyramax* Pregnancy Register. In these cases, the evolution of the pregnancy will be monitored with visits at 3, 6 and 9 months and 7 days after the delivery. Information on the drugs taken during the pregnancy as well as AEs/SAEs/AESIs and the health status of the newborn(s) will be collected.	7 visit, the patient will be referred to the health facilities and blood will be taken for LFTs and haematology (~~minimum~~ Hb **mandatory**) assessment as well as for a full hepatitis panel.<br><br>Female patients must communicate to the local CHW or the study team if they get pregnant just before or after the start of the *Pyramax* treatment and for 2 months post-treatment. All pregnancies must be documented on the manufacturer's *Pyramax* Pregnancy Register. In these cases, the evolution of the pregnancy will be monitored with visits at 3, 6 and 9 months and 7 days after the delivery. Information on the drugs taken during the pregnancy as well as AEs/SAEs/AESIs and the health status of the newborn(s) will be collected. **On the last day of follow-up (usually Day 28 ±2 or in case of early termination, on the respective day), a urine pregnancy test will be performed for all women of child-bearing potential, defined as women who have experienced menarche and who are not permanently sterile or postmenopausal (defined as 12 consecutive months with no menses without an alternative medical cause). The investigator shall decide whether this is applicable or not based on the medical history of the patient, and on customs and practices of the region.**	Reworded for clarity<br><br>Urinary pregnancy test added to increase the chance to detect early pregnancies
**7. STUDY PROCEDURE AND DATA COLLECTION**<br><br>**7.2.3 Follow-up visits/contacts and clinical tolerability follow-up questionnaire**<br><br>**(p. 32-33)**	………………..<br><br>On Day 28 (±2 day) the CHW will visit the patient for a final study visit at home. He/she will report:<br><br>- His/her own 4 digit identification number (first two digits are the number of the health centre he/she is attached to, followed by a 2 digit number which will be assigned to him/her when the study starts).<br><br>- Date of the visit/contact.	………………..<br><br>On Day 28 (±2 day) the CHW will visit the patient for a final study visit at home. He/she will report:<br><br>- His/her own 4 digit identification number (first two digits are the number of the health centre he/she is attached to, followed by a 2 digit number which will be assigned to him/her when the study starts).<br><br>- Date of the visit/contact.	

	- Clinical condition (recovered status or continuation of the illnesses).<br>- Body temperature will be taken.<br>- Description of the symptoms experienced by the patient in case of a positive answer to the question: "Have you noticed any unusual event/symptom since your last visit to the centre?". Collect date of first manifestation of this/these event(s), symptom(s), intensity, treatment dis-/continuation, and evolution, in the specific section of the patient chart dedicated to the collection of Adverse Events.<br>- Drops of blood will be taken for thick blood smear and spot for PCR.<br>- Pregnancy status will also be enquired about<br>- Current treatments<br><br>If the adverse event belongs to the hepatic category, after transfer to the health facility, blood should be drawn for chemistry (at least AST, ALT, Bilirubin total and conjugated, ALP) and haematology (Hb mandatory) and a full hepatitis panel.<br>Should the patient present at the clinic or make contact with the CHW, it might be required to conduct an unscheduled unforeseen visit to report:<br>• Date of the visit/contact.<br>• Clinical condition (recovered status or continuation of the illnesses).<br>• Body temperature will be recorded	- Clinical condition (recovered status or continuation of the illnesses).<br>- Body temperature will be taken.<br>- Description of the symptoms experienced by the patient in case of a positive answer to the question: *"Have you noticed any unusual event/symptom since your last visit to the centre**/contact with CHW**?".* Collect date of first manifestation of this/these event(s), symptom(s), intensity, treatment dis-/continuation, and evolution, in the specific section of the patient chart dedicated to the collection of Adverse Events.<br>- Drops of blood will be taken for thick blood smear and spot for PCR.<br>- **Any pregnancy: a urine pregnancy test will be performed for all women of child- bearing potentials.** ~~Pregnancy status will also be enquired about~~<br>- Current treatments<br><br>If the adverse event belongs to the hepatic category, after transfer to the health facility, blood should be drawn for chemistry (at least AST, ALT, Bilirubin total and conjugated, ALP) and haematology (Hb mandatory) and a full hepatitis panel.<br><br>Should the patient present at the clinic or make contact with the CHW, it might be required to conduct an unscheduled unforeseen visit to report:<br>• Date of the visit/contact.<br>• Clinical condition (recovered status or continuation of the illnesses).	Added: patient can contact the CHW<br><br>Urinary pregnancy test added to increase the chance to detect early pregnancies

	• Description of the symptoms experienced by the patient in case of a positive answer to the question: "Have you noticed any unusual event/symptom since your last visit to the centre?". Collect date of first manifestation of this/these event(s), symptom(s), intensity, treatment dis-/continuation, and evolution, in the specific section of the patient chart dedicated to the collection of Adverse Events.<br>• Drops of blood will be taken for thick blood smear and spot for PCR.<br>• Pregnancy status will be checked.<br>• Current treatments.	• Body temperature will be recorded<br>• Description of the symptoms experienced by the patient in case of a positive answer to the question: "Have you noticed any unusual event/symptom since your last visit to the centre**/contact with CHW**?". Collect date of first manifestation of this/these event(s), symptom(s), intensity, treatment dis- /continuation, and evolution, in the specific section of the patient chart dedicated to the collection of Adverse Events.<br>• Drops of blood will be taken for thick blood smear and spot for PCR.<br>• Pregnancy status ~~will be checked~~.<br>• **In case of early termination, a urine pregnancy test will be performed for all women of child-bearing potential.**<br>• Current treatments.	Added: patient can contact the CHW<br><br>Urinary pregnancy test added to increase the chance to detect early pregnancies
**7.3.2 Patient lost to follow-up**<br>**(p. 34)**	The study staff will take all appropriate measures to perform each patient's follow-up visits. However, some patients may be lost to follow up. This information will be recorded in the patient's chart. A patient will be considered lost to follow up if he cannot be visited by the CHW before Day 10.<br><br>If a subject is lost to follow-up, all reasonable efforts must be made by the study site personnel to contact the subject and to determine endpoint status and the reason for discontinuation/withdrawal.	The study staff will take all appropriate measures to perform each patient's follow-up visits. However, some patients may be lost to follow up. This information will be recorded in the patient's chart. A patient will be considered lost to follow up if he cannot be visited by the CHW before Day 10.<br><br>~~If a subject is lost to follow-up, all reasonable efforts must be made by the study site personnel to contact the subject and to determine endpoint status and the reason for discontinuation/withdrawal.~~<br><br>**All reasonable efforts must be made by the study site personnel to contact the subject for the Day 28 visit, to determine the endpoint status and the reason for discontinuation/withdrawal until a reasonable period.**	Deleted/added to clarify procedure

**8.2 Monitoring of adverse events**<br><br>**(p. 35-36)**	Patient charts (completely filled in with the contact visit at Day 7 (±1 day), and with any AE(s) reported in the 28 days after the start of the treatment) will be captured in the CRF by the Principal Investigator (PI) of the study centre via the electronic capture system. Chart review meetings shall be organised regularly, during which the PI, his/her team and the people responsible for the pharmacovigilance in the country participating in the study shall review AE data (intensity, causality, date of event manifestation in relation to the start of treatment and the initial parasite species identified on the patient). Given the absence of pharmacovigilance systems in most countries involved in the study, the experience of Shin Poong Pharmacovigilance (SPPV) and national pharmacovigilance center of DRC (CNPV) will be used in this study. The principal investigator in each country and his team will make a first analysis of the safety reports received in order to assess completeness. Safety data should be entered immediately in the eCRF. Safety reports are reviewed by Shin Poong Pharmacovigilance (SPPV) for quality control. Designated personnel of CNPV / UPC-PV will have access to the trial database. They will have access to the electronic version of the CRF and can address a request to the principal investigator of the site concerned to add any further information needed for the analysis. CNPV will conduct the final review of the CRF for any patients who have had an adverse event, then CNPV will code each adverse event reported according to standard medical terminology (MedDRA). The PI will make a causality assessment. SPPV/CNPV/PI should be informed about any new data on a patient for whom a causality link had already been established to reconsider its analysis and if necessary reassess the causality. Among these new data will be included any data acquired from the	Patient charts (completely filled in with the contact visit at Day 7 (±1 day), and with any AE(s) reported in the 28 days after the start of the treatment) will be captured in the CRF by the Principal Investigator (PI) of the study centre via the electronic capture system. Chart review meetings shall be organised regularly, during which the PI, his/her team and the people responsible for the pharmacovigilance in the country participating in the study shall review AE data (intensity, causality, date of event manifestation in relation to the start of treatment and the initial parasite species identified on the patient). Given the absence of pharmacovigilance systems in most countries involved in the study, the experience of Shin Poong Pharmacovigilance (SPPV) and national pharmacovigilance center of DRC (CNPV) will be used in this study. The principal investigator in each country and his team will make a first analysis of the safety reports received in order to assess completeness. Safety data should be entered immediately in the eCRF. Safety reports are reviewed by Shin Poong Pharmacovigilance (SPPV) for quality control. **The medical monitor** ~~Designated personnel of CNPV / UPC PV~~will have access to the trial database. **He** ~~They~~will have access to the electronic version of the CRF and can address a request to the principal investigator of the site concerned to add any further information needed for the analysis. **The medical monitor** ~~CNPV~~will conduct the final review of the CRF for any patient~~s~~ who have had an adverse event~~,~~ The eCRF provider ~~then CNPV~~will code each adverse event reported according to standard medical terminology (MedDRA)~~.~~ **and the medical monitor will then review the list of coded terms.** The PI will make a causality assessment. SPPV/CNPV/PI should be informed about any new data on a patient for whom a causality link had already been established to	Updated/added to clarify procedure

	hepatitis panel results, where appropriate, the recurrence of the same adverse event in a patient who is retreated after a subsequent episode of uncomplicated malaria, etc. Data shall only be modified following an official query procedure. Safety data will be transmitted to the WHO Collaborating Centre for Pharmacovigilance via the CNPV of DRC to achieve a quality control of the review and to provide necessary feedback to CNPV and SPPV.	reconsider its analysis and if necessary reassess the causality. Among these new data will be included any data acquired from the hepatitis panel results, where appropriate, the recurrence of the same adverse event in a patient who is retreated after a subsequent episode of uncomplicated malaria, etc. Data shall only be modified following an official query procedure. Safety data will be transmitted to the WHO Collaborating Centre for Pharmacovigilance via the CNPV of DRC to achieve a quality control of the review and to provide necessary feedback to CNPV and SPPV.	
**8.6 Pregnancy**<br>**(p. 39-40)**	………………..<br>Pregnancy is not an adverse event unless the outcome of the pregnancy fulfils one of the serious criteria as defined in Section 8.3. Pregnancy should be reported by using the appropriate pregnancy report form within 24 hours of knowledge to SPPV (Tel/Fax [redacted])].	………………..<br>Pregnancy is not an adverse event unless the outcome of the pregnancy fulfils one of the serious criteria as defined in Section 8.3. Pregnancy should be reported by using the appropriate pregnancy report form within 24 hours of knowledge to SPPV (Tel/Fax [redacted])].<br>**A urine pregnancy test will be performed for all women of child-bearing potential, on the last day of follow-up (usually Day 28 ±2 or in case of earlier termination, on the respective day), at home or at the health facility, if the patient is transferred to the health facility. Women of child-bearing potential are defined as women who have experienced menarche and who are not permanently sterile or postmenopausal, defined as 12 consecutive months with no menses without an alternative medical cause. The investigator shall decide whether this is applicable or not based on the medical history of the patient.**	Urinary pregnancy test added to increase the chance to detect early pregnancies
**12.3 Expected risks**<br>**(p. 46)**	………………..<br>The safety of *Pyramax* in pregnant women has not	………………..<br>The safety of *Pyramax* in pregnant women has not	

	been established. It is therefore not recommended in pregnant women. Women will be invited to inform the community health worker of any pregnancy occurring during the 2 months following the start of the *Pyramax* treatment. Pregnancy will be followed-up with visits at 3, 6 and 9 months and 7 days after delivery.	been established. It is therefore not recommended in pregnant women. Women will be invited to inform the community health worker of any pregnancy occurring during the 2 months following the start of the Pyramax treatment. Pregnancy will be followed-up with visits at 3, 6 and 9 months and 7 days after delivery. **On the last day of follow-up (usually Day 28 ±2 or in case of early termination, on the respective day), a urine pregnancy test will be performed for all women of child-bearing potential.**	Urinary pregnancy test added to increase the chance to detect early pregnancies

Protocol Amendment Number 2 - Approval and Authorisation

Protocol Number: SP-C-021-15

[redacted]

CLINICAL STUDY PROTOCOL AMENDMENT NUMBER 3 - GENERAL

Sponsor:	Shin Poong Pharmaceuticals Co. Ltd
Protocol Number:	SP-C-021-15
Study Title:	PHASE IIIB/IV COHORT EVENT MONITORING STUDY TO EVALUATE, IN REAL LIFE SETTING, THE SAFETY AND TOLERABILITY IN MALARIA PATIENTS OF THE FIXED-DOSE ARTEMISININ-BASED COMBINATION THERAPY PYRAMAX (PYRONARIDINE-ARTESUNATE)

Refers to the Current Approved Protocol Version 8.0, 13 September 2018

Date: 15 March 2019

1 INTRODUCTION

This protocol amendment is due to a lower incidence of very young children presenting with malaria and, therefore, a slightly lower proportion of malaria episodes coming from children below 1 year of age compared to that which was initially anticipated from epidemiological data provided by the site in Lambaréné and reported in the protocol:

1) One of the secondary evaluation criteria of the present study is to assess the safety (including hepatic safety) and tolerability of Pyramax treatment in a patient population below 1 year of age. The involvement of this vulnerable population was requested from the EMA, without a specific reference to the proportion of children to be enrolled from this youngest age group. In the planning for the study, based on non-published data, it was reported that children below 1 year of age constitute approximately 9% of paediatric malaria cases at a sentinel hospital in Lambaréné (Gabon). Based on this epidemiological data, a target of 2% of malaria episodes coming from children below 1 year of age was proposed and assumed as achievable by all sites.

   As of 11MAR2019 in line with the primary study objectives, a total of 8572 malaria episodes were reported in the recruited patients, including 135 malaria episodes in patients with elevated LFTs at baseline (ALT/ALT > 2xULN); both targets per protocol were therefore achieved (8572 and 120 episodes, respectively) and recruitment is considered completed. From the total number of malaria episodes, 147 of them were reported in children below the age of 1 year; the proportion being 1.7% and was, therefore, slightly below the expectation of 2%. Considering that this proportion of younger patients nonetheless provides an important contribution to the overall population of malaria patients treated with Pyramax, and that there are sufficient patients to satisfy the primary and secondary study objectives, it is proposed to amend the protocol to remove the specific reference to the 2% proportion of patients under 1 year.

2 CHANGES MADE TO THE PROTOCOL BY THIS AMENDMENT

Changes/additions to the text are marked in **yellow**, with addition in bold and deletion in strikethrough.

**Section Location**	Original Text (Final protocol version 8.0, dated 13 September 2018)	Amended Text (Amended protocol dated 15 March 2019)	Rationale
**2. SUMMARY**<br>**Design and duration of the study**	This is a non-comparative Cohort Event Monitoring study. All the patients for whom a diagnosis of uncomplicated malaria (according to the WHO criteria) is suspected, will undergo an	This is a non-comparative Cohort Event Monitoring study. All the patients for whom a diagnosis of uncomplicated malaria (according to the WHO criteria) is suspected, will undergo an	

(p. 13)	RDT/microscopy. If the presence of malaria is confirmed and the patient (or parent/ guardian in case of children) provides his/her informed consent, he/she will be assigned a unique ID number and will be enrolled in the study. Blood will be taken for a blood spot for PCR analysis, for retrospective LFT assessment as well as for a retrospective viral hepatitis assessment to be done only in patients with abnormal LFTs. A blood sample will be taken for haematology (Hb mandatory). The first dose of *Pyramax* will be given under Direct Observational Treatment (DOT) conditions, but patients will take their second and third doses of *Pyramax* under usual conditions (including unsupervised medication intake). Patients will be also required to attend the health care facilities if they have any clinical signs or symptoms of hepatotoxicity.<br><br>A cohort of at least 2% of children who are <1 year of age will also be included for monitoring of liver function.<br><br>The patients will be visited at home at Day 7 ±1 day, by a community health worker (CHW), in order to capture the adverse events (AEs) experienced. In the case of signs or symptoms of hepatotoxicity or hepatic related AEs, the patients will be referred to the health facilities and blood will be taken on this occasion for LFT assessment, haematology (Hb mandatory), and where appropriate for full hepatitis panel assessment. If a patient cannot be directly reached, the CHW must organize an appointment with this patient before Day 10. Special procedures will be followed in case of serious adverse events (SAEs) and/or severe adverse events and events classified as being of special interest (AESI) (see specific section).	RDT/microscopy. If the presence of malaria is confirmed and the patient (or parent/ guardian in case of children) provides his/her informed consent, he/she will be assigned a unique ID number and will be enrolled in the study. Blood will be taken for a blood spot for PCR analysis, for retrospective LFT assessment as well as for a retrospective viral hepatitis assessment to be done only in patients with abnormal LFTs. A blood sample will be taken for haematology (Hb mandatory). The first dose of *Pyramax* will be given under Direct Observational Treatment (DOT) conditions, but patients will take their second and third doses of *Pyramax* under usual conditions (including unsupervised medication intake). Patients will be also required to attend the health care facilities if they have any clinical signs or symptoms of hepatotoxicity.<br><br>A cohort of ~~at least~~ **approximately** 2% of children who are <1 year of age will also be included for monitoring of liver function.<br><br>The patients will be visited at home at Day 7 ±1 day, by a community health worker (CHW), in order to capture the adverse events (AEs) experienced. In the case of signs or symptoms of hepatotoxicity or hepatic related AEs, the patients will be referred to the health facilities and blood will be taken on this occasion for LFT assessment, haematology (Hb mandatory), and where appropriate for full hepatitis panel assessment. If a patient cannot be directly reached, the CHW must organize an appointment with this patient before Day 10. Special procedures will be followed in case of serious adverse events (SAEs) and/or severe adverse events and events classified as being of special interest (AESI) (see specific section).	To be less restrictive with the proportion of children below 1 year of age.

**5. DESCRIPTION OF THE STUDY**<br><br>**5.1 Description of the study design**<br><br>(p. 25)	This is a non-comparative Cohort Event Monitoring study of 8,572 malaria episodes to be conducted in Central and West Africa. Treatment is with *Pyramax* as per the product label and Investigator Brochure. Each patient will be provided with 3 days oral treatment with *Pyramax* to be administered on days 0, 1 & 2.<br><br>Total study duration for a single patient episode is 28±2 days.<br><br>A cohort of at least 2% of children who are <1 year of age will be included.	This is a non-comparative Cohort Event Monitoring study of 8,572 malaria episodes to be conducted in Central and West Africa. Treatment is with *Pyramax* as per the product label and Investigator Brochure. Each patient will be provided with 3 days oral treatment with *Pyramax* to be administered on days 0, 1 & 2.<br><br>Total study duration for a single patient episode is 28±2 days.<br><br>A cohort of ~~at least~~ **approximately** 2% of children who are <1 year of age will be included.	To be less restrictive with the proportion of children below 1 year of age.
**5.2 Study duration**<br><br>(p. 26)	The study is anticipated to last approximately 18 to 24 months and will end when 1) the total number of patients (n=8,572) is reached and 2) at least 120 episodes of malaria in patients with raised liver enzymes at baseline have been recruited and 3) at least 2% (n=172) children below the age of 1 year will have been recruited into the study.	The study is anticipated to last approximately 18 to 24 months and will end when 1) the total number of ~~patients~~ **malaria episodes** (n=8,572) is reached and 2) at least 120 episodes of malaria in patients with raised liver enzymes at baseline have been recruited. ~~and 3) at least 2% (n=172) children below the age of 1 year will have been recruited into the study.~~	Correction; to be consistent with the rest of the protocol.<br><br>Deleting the 2% of children below 1 year of age in the study duration criteria.
**6. STUDY POPULATION AND PATIENT SELECTION**<br><br>**6.1 Sample size**<br><br>(p. 28)	Based on the primary objective, 120 malaria episodes in patients with baseline raised AST/ALT value >2xULN will be needed for a 81.6% probability to observe one severe hepatic event in this population, defined as:<br><br>• Appearance of clinical signs and symptoms of possible hepatotoxicity associated with a rise in ALT/AST >2 x baseline value.	Based on the primary objective, 120 malaria episodes in patients with baseline raised AST/ALT value >2xULN will be needed for a 81.6% probability to observe one severe hepatic event in this population, defined as:<br><br>• Appearance of clinical signs and symptoms of possible hepatotoxicity associated with a rise in ALT/AST >2 x baseline value.	

	Given the screening rate in previous studies of 1.4% for malaria patients with AST/ALT >2xULN, recruitment will require an estimate of at least 8,572 malaria episodes. To mimic reality, patients can be included more than once in the study. At each new malaria episode, the patient will be assigned the same 8 first digits of his ID number, followed by E01, E02, etc, to illustrate repeated treatment 1, 2, etc.<br>…<br>The study duration is estimated to be approximately 18 to 24 months after study start, depending on enrolment being completed over 1 or 2 malaria seasons. However, the study will not be terminated until at least 120 malaria episodes in patients with LFTs >2xULN are enrolled and until at least 2% (n=172) children below the age of 1 year will be recruited.	Given the screening rate in previous studies of 1.4% for malaria patients with AST/ALT >2xULN, recruitment will require an estimate of at least 8,572 malaria episodes. To mimic reality, patients can be included more than once in the study. At each new malaria episode, the patient will be assigned the same 8 first digits of his ID number, followed by E01, E02, etc, to illustrate repeated treatment 1, 2, etc.<br>…<br>The study duration is estimated to be approximately 18 to 24 months after study start, depending on enrolment being completed over 1 or 2 malaria seasons. However, the study will not be terminated until at least 120 malaria episodes in patients with LFTs >2xULN are enrolled. ~~and until at least 2% (n=172) children below the age of 1 year will be recruited.~~	Deleting the 2% of children below 1 year of age in the study duration criteria.
**6.3.2 Patient selection** (p. 29)	At least 8,572 episodes of malaria are needed in the study. To mimic reality, patients can be included more than once in the study. A washout period of at least 28 days must be maintained between two consecutive treatments with *Pyramax*. A subset of 2% of children under the age of 1 year will be recruited.	At least 8,572 episodes of malaria are needed in the study. To mimic reality, patients can be included more than once in the study. A washout period of at least 28 days must be maintained between two consecutive treatments with *Pyramax*. A subset of **approximately** 2% of children under the age of 1 year will be recruited.	To be less restrictive with the proportion of children below 1 year of age.
**Design and duration of the study**<br>(p.12)<br><br>(p.13)	… Blood will be taken for a blood spot for PCR analysis, for retrospective LFT assessment as well as for a retrospective viral hepatitis assessment to be done only in patients with abnormal LFTs.<br>…<br>At Day 28 ±2 days, all patients will be seen by a CHW for a final assessment, including blood taken for thick blood smear and spot.	Blood will be taken for ~~a~~ blood spot**s** for PCR analysis, for retrospective LFT assessment as well as for a retrospective viral hepatitis assessment to be done only in patients with abnormal LFTs.<br>…<br>At Day 28 ±2 days, all patients will be seen by a CHW for a final assessment, including blood taken for thick blood smear**s** and spot**s**.	Correction of typographical error.

**STUDY FLOW CHART:**<br>(Table on p. 18)	Blood spot for PCR	Blood spot**s** for PCR	Correction of typographical error.
5.1.1. Study assessments<br>Day 7 or any unforeseen visit (p.26)<br>Day 28 (p.26)	At any unforeseen visit, in addition to the procedures above, malaria diagnostics and a blood spot for PCR analysis will be undertaken.<br>At Day 28 ±2 days, all patients will be seen by a CHW for a final assessment. At this occasion, blood will be taken for thick blood smear and spot.	At any unforeseen visit, in addition to the procedures above, malaria diagnostics and ~~a~~ blood spot ~~for~~ PCR analysis will be undertaken.<br>At Day 28 ±2 days, all patients will be seen by a CHW for a final assessment. At this occasion, blood will be taken for thick blood smear and **blood** spot**s**.	Correction of typographical error.
**6.3.2 Patient selection**<br>(p. 30)	A patient will be included in the study where he/she meets the selection criteria of the protocol and agrees to participate. In particular, to enter the study, the patient should accept: (i) that a small amount of blood is taken for the preparation of a blood spot, as well as for a LFTs assessment (ii) that the CHW visit him/her at home, (iii) that the data collected during those visits are registered and analysed and finally (iv) that, in case an SAE and/or an AE classified as severe and/or an AESI (see section 8.5) should occur he/she is to contact the CHW immediately.	A patient will be included in the study where he/she meets the selection criteria of the protocol and agrees to participate. In particular, to enter the study, the patient should accept: (i) that a small amount of blood is taken for the preparation of ~~a~~ blood spot**s**, as well as for a LFTs assessment (ii) that the CHW visit him/her at home, (iii) that the data collected during those visits are registered and analysed and finally (iv) that, in case an SAE and/or an AE classified as severe and/or an AESI (see section 8.5) should occur he/she is to contact the CHW immediately.	Correction of typographical error.
**7.2.3 Follow-up visits/contacts and clinical tolerability follow-up questionnaire**<br>(p. 32) …<br>(p.33)	In addition, a thick blood smear will be prepared as well as a blood spot for future analysis.<br>In case any treatment is prescribed by a STM staff or the study doctor, it will be reported in the CRF and on the patient chart.<br>…<br>- Drops of blood will be taken for thick blood smear and spot for PCR.	In addition, ~~a~~ thick blood smear**s** will be prepared as well as ~~a~~ blood spot**s** for future analysis.<br>In case any treatment is prescribed by a STM staff or the study doctor, it will be reported in the CRF and on the patient chart.<br>…<br>- Drops of blood will be taken for thick blood smear**s** and spot**s** for PCR.	Correction of typographical error.
**10.1 Collection and validation of data**<br>(p.42)	The study coordinator shall organise regular capture of these data by each study centre together with the results of the thick blood smears/RDT and blood spot of those patients.	The study coordinator shall organise regular capture of these data by each study centre together with the results of the thick blood smears/RDT**s** and blood spot**s** of those patients.	Correction of typographical error.

Protocol Amendment Number 3 - Approval and Authorisation

Protocol Number: SP-C-021-15

[redacted]

Statistical Analysis Plan (SAP)

**Protocol Title:**	Phase IIIB/IV Cohort Event Monitoring study to evaluate, in real life setting, the safety and tolerability in malaria patients of the fixed-dose Artemisinin-based Combination Therapy Pyramax (pyronaridine-artesunate).
**Protocol Number:**	SP-C-021-15
**Protocol Version, Date**	Final Version 8.0 Date: 13SEP2017
**ICON ID:**	3037/0007
**Document Version, Date:**	Final Version 1.0, 28Jan2019

Prepared by:

ICON Clinical Research Services

On behalf of:

SHIN POONG PHARMACEUTICALS/MMV

Confidentiality statement:

- The information provided in this document is strictly confidential.
- The recipients of the SAP must not disclose the confidential information contained within this document or any related information to other persons without the permission of the sponsor.
- In addition, the recipients of the SAP must keep this confidential document in a controlled environment which prevents unauthorized access to the document.

Statistical Analysis Plan (SAP)

SIGNATURE PAGE

Prepared at ICON Clinical Research by:

Prasanth Sasidharan, Sr. Statistician I	Date<br>(DD Mmm YYYY)

Reviewed at ICON Clinical Research by:

Rolf Dietl, Sr. Biostatistician I	Date<br>(DD Mmm YYYY)

Approved at SHIN POONG by:

Jang Sik Shin, Pyramax Project Leader, Shin Poong Pharmaceuticals	Date<br>(DD Mmm YYYY)

Statistical Analysis Plan (SAP)

Approved at MMV by:

Dr Stephan Duparc, Chief Medical Officer

Date
(DD Mmm YYYY)

Approved at University of Tübingen by:

Dr Michael Ramharter, Coordinating Principal Investigator

Date
(DD Mmm YYYY)

Statistical Analysis Plan (SAP)

REVISION HISTORY

Version/Date	Version name	Section	Changes implemented
Version 1.0/ 28-Jan-2019	Initial approved version	N/A	N/A

TABLE OF CONTENTS

SIGNATURE PAGE 2
REVISION HISTORY 4
TABLE OF CONTENTS 5
LIST OF ABBREVIATIONS 7
1 INTRODUCTION 9
2 STUDY OBJECTIVES 10
2.1 Primary objective 10
2.2 Secondary objectives 10
2.3 Exploratory objective 10
3 STUDY DESIGN 11
3.1 General study design 11
3.2 Randomization and blinding 12
3.3 Study treatments and assessments 12
4 STUDY ENDPOINTS 14
4.1 Primary endpoint 14
4.2 Secondary endpoints 14
4.3 Efficacy endpoints 15
5 SAMPLE SIZE AND POWER 16
6 ANALYSIS POPULATIONS 17
6.1 Enrolled population 17
6.2 Safety population (Safety) 17
6.3 Microbiological-Intention-To-Treat population (m-ITT) 17
6.4 Safety matched population 17
6.5 Per-Protocol population (PP) 17
6.6 Protocol deviations/violations and exclusions from analysis populations 18
7 Statistical Considerations and analysis 19
7.1 Derived Variables 19
7.1.1 Definitions relative to safety parameters 21
7.1.1.1 Definition of Adverse Event and Serious Adverse Event 21
7.1.1.2 Adverse Event of Special Interest (AESI) 21
7.2 Handling of missing data and outliers 22
7.2.1 Missing data analysis methods 22
7.2.2 Handling of missing or incomplete dates 22
7.2.2.1 Missing or incomplete dates for prior and concomitant medications 22
7.2.2.2 Missing or incomplete dates for adverse events 22
7.2.3 Handling of adverse events with no investigator causality 22
8 STATISTICAL METHODS 23

8.1 General statistical conventions 23
8.2 Subject disposition 23
8.3 Protocol deviations 23
8.4 Demographics and baseline characteristics 24
8.4.1 Demographics 24
8.4.2 Other baseline characteristics 24
8.4.3 Medical history 24
8.4.4 Prior and concomitant medications 24
8.5 Extent of exposure 25
8.5.1 Treatment compliance 25
8.6 Safety analyses 25
8.6.1 Adverse events 27
8.6.2 Clinical laboratory evaluations 28
8.6.3 Vital signs 28
8.6.4 Physical examinations 29
8.6.5 Electrocardiograms 29
8.6.6 Subgroup analysis 29
8.7 Efficacy analyses 29
8.7.1 Analysis of secondary efficacy endpoints 29
8.8 Interim analysis 30
9 CHANGES TO PLANNED ANALYSIS FROM STUDY PROTOCOL 31
10 REFERENCES 32
11 APPENDICES 33
Appendix I 33
Appendix II 34
Appendix III 35

LIST OF ABBREVIATIONS

The following abbreviations will be used within this SAP.

Abbreviation or special term	Explanation
AE	Adverse Event
AESI	Adverse Event of Special Interest
ALP	Alkaline phosphatase
ALT	Alanine aminotransferase
AST	Aspartate aminotransferase
ATC	Anatomical Therapeutic Chemical
BMI	Body Mass Index
CHW	Community Health Worker
CI	Confidence Interval
CRF	Case Report Form
CSR	Clinical Study Report
DBL	Database Lock
DILI	Drug Induced Liver Injury
DSMB	Drug Safety Monitoring Board
eCRF	Electronic Case Report Form
eDISH	Evaluation of Drug Induced Serious Hepatotoxicity
Hb	Haemoglobin
HIV	Human immunodeficiency virus
ICH	International Conference on Harmonisation
kg	Kilogram
LFTs	Liver function tests
MedDRA	Medical Dictionary for Regulatory Activities
m-ITT	Microbiological-Intention-To-Treat
MUAC	Mid-Upper Arm Circumference
PCR	Polymerase Chain Reaction
PP	Per Protocol
PS	Propensity Score
PT	Preferred Term

RDT	Rapid Diagnostic Test
SAE	Serious Adverse Event
SAP	Statistical Analysis Plan
SAS	Statistical Analysis System
SD	Standard Deviation
SOC	System Organ Class
TFLs	Tables, Figures and Listings
ULN	Upper Limit of Normal
WHODDE	World Health Organization Drug Dictionary Enhanced

1 INTRODUCTION

The purpose of this Statistical Analysis Plan (SAP) is to provide detailed descriptions of the statistical methods, data derivations and data displays for the study protocol SP-C-021-15 Final version 7.0 “Phase IIIB/IV Cohort Event Monitoring study to evaluate, in real life setting, the safety and tolerability in malaria patients of the fixed-dose Artemisinin-based Combination Therapy Pyramax (pyronaridine-artesunate)” dated 25 January 2017. The table of contents and templates for the Tables, Figures and Listings (TFLs) will be produced in a separate document.

Any deviations from this SAP will be described and justified in the Clinical Study Report (CSR).

The preparation of this SAP has been based on International Conference on Harmonisation (ICH) E3 and E9.

All data analyses and generation of TFLs will be performed using Statistical Analysis System (SAS) version 9.3 or higher.

2 STUDY OBJECTIVES

The main objectives of the study are to assess the safety of *Pyramax* particularly in malaria patients with underlying liver function test (LFT) abnormalities, in the general malaria patient population, but also in malaria patients who have co-morbid conditions, such as HIV (where known), and also in very small children (<1 year of age).

2.1 Primary objective

Evaluation and identification of hepatic safety events of *Pyramax* in a subgroup of patients enrolled with LFTs >2x Upper Limit of Normal (ULN) from blood taken immediately prior to treatment without any clinical signs or symptoms of hepatotoxicity and with signs and symptoms of uncomplicated malaria confirmed by a Rapid Diagnostic Test (RDT) or microscopy (thick blood smear).

2.2 Secondary objectives

Main secondary

Comparison of the clinical hepatic safety of *Pyramax* between a cohort of patients enrolled with LFTs >2xULN and a cohort of patients enrolled with normal LFTs matched for demographic characteristics.

Other secondary

- Evaluation of the safety and tolerability in patients with normal and abnormal LFTs at inclusion according to any possible hepatic underlying disease based on the finding of the hepatitis panel and according to their HIV status (where known), their nutritional status, their age (children < 1 year of age in comparison to the rest of the treated population) and their weight.
- Evaluation of the safety and tolerability in retreated patients with a special focus on the hepatic safety.
- Evaluation of the relationship between the occurrence of hepatic related adverse events with or without LFT abnormalities and the administration of concomitant medications (in particular paracetamol, herbal medicines and antiretroviral drug).
- Evaluation of the potential for hypersensitivity reactions.
- Evaluation of the efficacy and compliance of *Pyramax* when used under usual conditions (including unsupervised medication intake) in patients with signs and symptoms of uncomplicated malaria confirmed by a RDT or microscopy (thick blood smear).

2.3 Exploratory objective

- Not applicable

3 STUDY DESIGN

3.1 General study design

This is a non-comparative Cohort Event Monitoring study of 8,572 malaria episodes to be conducted in Central and West Africa. Treatment with *Pyramax* is as per the product label and Investigator Brochure. Each patient will be provided with 3 days oral treatment with *Pyramax* to be administered on days 0, 1 & 2.

Total study duration for a single patient episode is 28±2 days.

A cohort of at least 2% of children who are <1 year of age will be included. Even if this number is not reached, the study will be stopped after including 8572 malaria episodes and 120 episodes in patients with increased LFTs.

Study assessments

- Screening/Inclusion/Day 0
- Day 7 or any unforeseen visit
- Day 28

The study is anticipated to last approximately 18 to 24 months and will end when 1) the total number of malaria episodes (n=8,572) is reached and 2) when at least 120 episodes of malaria in patients with raised liver enzymes at baseline (i.e. at the start of the episode and not necessarily when the patient first enters the study) have been recruited and 3) when at least 2% (n=172) children below the age of 1 year have been recruited into the study.

3.2 Randomization and blinding

Not applicable, since this is an open label non-randomized study.

3.3 Study treatments and assessments

Total study duration for a single patient episode is 28±2 days.

The patients will be instructed to take one Pyramax (pyronaridine-artesunate) dose orally every 24 hours over a period of three consecutive days, i.e. at Day 0, then after 24 hours (Day 1) and after 48 hours (Day 2) from the first administration.

The dose will be based on body weight. Two formulations of *Pyramax* will be provided to facilitate the dosing in young infants, children and adults: 60:20mg granules presented in sachets and 180:60mg tablets of pyronaridine and artesunate respectively. For this study, *Pyramax* tablets are sent as bulk supplies to site in commercial boxes of blister packs containing 90 tablets per box. Within the box there are 10 blister packs with 9 tablets in each.

Only the first dose will be administered under supervision in the clinic, the second and third doses will be taken by the subject at home.

**Body weight**	**No. of Sachets (granule formulation) per day**
5 to < 8 kg	1
8 to < 15 kg	2
15 to < 20 kg	3
	**Number of Tablets per day**
20 to < 24 kg	1
24 to < 45 kg	2
45 to < 65 kg	3
≥ 65 kg	4

The detailed description of procedures and assessments to be conducted during this study is summarized in the Schedule of Study Assessments in Table 1 below.

Table 1: Schedule of Study Assessments

STUDY EXAMINATION	Screening/Inclusion Day 0/V1 (health centre)	Day 7 +/- 1 (home visit)	Day 28 +/- 2 (home visit)	Unforeseen Visit
Age / sex / weight / height	X			
Vital signs (pulse, blood pressure)	X			
Body Temperature	X	X	X	X
Malaria symptoms / Hepatic symptoms / Other symptoms	X	X	X	X
Rapid Diagnostic Test (RDT)* or thick blood smear	X		X	X
Informed Consent	X			
Medical history	X			
Physical examination	X			
Previous medications (last month)	X			
Blood spot for PCR	X		X	X
Blood sample taken for AST/ALT, Total Bilirubin+/- Conjugated Bilirubin, Alkaline phosphatase and haematology (haemoglobin mandatory)	X ¶	X(#)	X(#)	X(#)
Viral hepatitis assessment (hepatitis A, B, C, delta (if hepatitis B positive) and E)	X[a]			
Hepatitis panel		X(#)	X(#)	X(#)
Inclusion/Exclusion	X			
Prescription of *Pyramax*	X			
AE recording	X (only SAEs)	X	X	X
Concomitant medications	X	X	X	X
Compliance assessment		X		
Reported Pregnancy	X	X	X	X

*RDT only possible at D0, thick blood smear mandatory at D28.
¶LFT samples will be only analysed retrospectively
[a]Blood for viral hepatitis assessment will be drawn from all patients at baseline. For patients retrospectively found with baseline AST or ALT >2xULN, the blood for viral hepatitis assessment will be sent to the central laboratory for analysis, while for all other patients (i.e. with AST and ALT <2xULN) the blood for viral hepatitis assessment will be destroyed.
#In the case of clinical signs of possible hepatotoxicity/DILI, LFTs & Hb assessment will be performed as soon as the patient arrives at the health centre and blood for a full hepatitis panel will be taken in case of AESI or SAE related to hepatotoxicity.

4 STUDY ENDPOINTS

4.1 Primary endpoint

Hepatic adverse events captured by the Community Health Worker (CHW) at D7 ± 1 day or at any unforeseen visits in case of signs or symptoms of hepatotoxicity or hepatic related AEs, after clinical confirmation by a physician at the health centre then confirmation by biochemistry assessment.

Analysis of hepatic adverse events will be performed on a sub-group of patients retrospectively identified with abnormal baseline LFTs (AST or ALT >2xULN).

4.2 Secondary endpoints

Main secondary evaluation criterion:

Comparative analysis of the clinical hepatic safety (signs or symptoms of hepatotoxicity or hepatic related AEs confirmed biologically) will be made between patients having abnormal LFTs (AST or ALT >2xULN) at enrolment versus a cohort of patients with normal LFTs (AST and ALT ≤2xULN) matched for demographic characteristics.

Other secondary evaluation criteria

- A safety analysis will be performed in patients with normal and abnormal LFTs at inclusion according to any possible hepatic underlying disease based on the finding of the hepatitis panel and according to their HIV status (where known), their nutritional status, their age (children < 1 year of age in comparison to the rest of the treated population) and their weight.
- Safety (including hepatic safety) in retreated patients.
- At any unforeseen visits in case of signs or symptoms of hepatotoxicity or hepatic related AEs, special efforts will be made to assure and record adequate follow-up of serious and/or severe adverse events and of the adverse events of special interest (AESI) (see section 7.1.1.2).
- Biological/LFTs assessment (AST/ALT, bilirubin total and conjugated, alkaline phosphatases). Assessment of baseline LFT results where signs or symptoms of hepatotoxicity or hepatic related AEs were reported at Day 28 or at any unforeseen visit.
- The relationship between the occurrences of hepatic related adverse events with or without LFT abnormalities and the administration of concomitant medications (in particular paracetamol, herbal medicines and antiretroviral drugs) will be evaluated.
- Incidence of hypersensitivity reactions.
- Compliance will be assessed by interview and counting the number of tablets or sachets of granules dispensed but not taken, when the CHW visits the patient at home.

4.3 Efficacy endpoints

- Crude Day 28 cure rate for *P. falciparum*, Crude Day 28 cure rate by species and PCR-adjusted Day28 cure rate for *P. falciparum*.
- Time between malaria episodes and frequency of repeat episodes before and after Day 28.

5 SAMPLE SIZE AND POWER

Based on the primary objective, 120 malaria episodes in patients with baseline AST/ALT value >2xULN will be needed for an 81.6% probability to observe one severe hepatic event in this population, defined as:

- Appearance of clinical signs and symptoms of possible hepatotoxicity associated with a rise in ALT/AST >2 x baseline value.

Given the screening rate in previous studies of 1.4% for malaria patients with AST/ALT >2xULN, recruitment will require an estimate of at least 8,572 malaria episodes. To mimic reality, patients can be included more than once in the study. At each new malaria episode, the patient will be assigned the same 8 first digits of his ID number, followed by E01, E02, etc, to illustrate repeated treatment 1, 2, etc.

The study duration is estimated to be approximately 18 to 24 months after study start, depending on enrolment being completed over 1 or 2 malaria seasons. However, the study will not be terminated until at least 120 malaria episodes in patients with LFTs >2xULN are enrolled and until at least 2% (n=172) children below the age of 1 year will be recruited.

6 ANALYSIS POPULATIONS

Populations will be defined based on the episode-level, meaning a patient could have been included more than once for a given population and may be excluded from a specific analysis population for a specific episode only.

6.1 Enrolled population

All the malaria episodes for subjects that have signed the informed consent form and enrolled into the study as per the Inclusion/Exclusion criteria. This population will be used for the listings.

6.2 Safety population (Safety)

The Safety population will include all the enrolled population that have received any amount of study medication for a specific episode. i.e., an episode without any amount of study medication will be excluded from Safety for a specific episode only.

6.3 Microbiological-Intention-To-Treat population (m-ITT)

The m-ITT population will include all the malaria episodes that have received any amount of study medication for a specific episode and have confirmed positive parasitaemia at baseline.

6.4 Safety matched population

The Safety matched population will consist of all the enrolled malaria episodes in Safety population that had baseline abnormal LFTs which are matched to episodes with baseline normal LFTs using propensity score method. The unmatched malaria episodes of abnormal or normal LFTs will be excluded from the Safety matched population. The Safety matched population will include two distinct cohorts with different numbers of episodes, a) 1:5 matched cohort used for logistic regression and b) 1:1 matched cohort used for the description of hepatic events by SOC and PT.

6.5 Per-Protocol population (PP)

The PP population will consist of all the malaria episodes in m-ITT population meeting pre-defined criteria as follows:

1. Subjects having completed a full course of study medication and having known efficacy endpoints for the specific malaria episode.
2. Subjects who do not vomit after the study drug administration (except where a subject may vomit after the first drug administration and have a repeat full dose - in this case, the repeated dose should not be vomited).
3. No prior or concomitant medication (except paracetamol) which may interfere with the treatment outcome, up to Day 28. A medical review will be performed to confirm the exclusion of a subject from the PP population at the time of final analysis.

6.6 Protocol deviations/violations and exclusions from analysis populations

All violations and exclusions of a specific episode from analysis populations will be identified at the Classification Meeting just prior to the final database lock (DBL). Exclusion from the PP population will be identified based on a pre-defined list of potential major protocol deviations.

7 STATISTICAL CONSIDERATIONS AND ANALYSIS

7.1 Derived Variables

Variables	Formula
**Demographic and Baseline characteristics**	
Age at informed consent (in years)	Age (years) = (date of informed consent - date of birth + 1) / 365.25
[illegible]	Body mass index (BMI) will be calculated in kg/m2 as: weight (kg)/ (height (m))^2.
Malnourished	Malnourished = Subjects with age <6 years: Mid-upper arm circumference <115 mm; Subjects with age 6-19: BMI <-3 Standard deviation (SD) based on the BMI data (z score tables for boys and girls)[4]; Subjects with age >19 year: BMI <18.5 kg/m2<br>The rest of the patients will be considered as non-malnourished.
**Duration**	
Study day at any visit	Study day = (Assessment date – date of first *Pyramax* administration).
Time between malaria episodes (days)	Calculate only if Episode number is >1<br>[(Date of enrolment in x^{th} episode versus date of enrolment in x^{th}-1 episode)]
**Drug Compliance**	
Compliance	If patient did not vomit after study drug administration on day 0 and no re-dosing.<br>100 × [Number of sachets/tablets taken/ (total number of sachets/tablets planned to be taken per day (according to Subject body weight) × 3)]<br>If patient vomited within 30 minutes after study drug administration on day 0 and re-dosed consider only the amount of re-dose for compliance.<br>This analysis will be repeated only taking the sachets/tablets presented to the CHW into account.
**Prior and concomitant medications**	
Prior medications	Those that start and stop before study drug intake

Concomitant medications	Those taken after study drug intake, even if they start prior to dosing.
**Cure rate**	
Crude Day 28 cure rate	Number of episodes in patients with clearance of parasites within 7 days, and without subsequent return of parasites during the 28-day period following the first dose, as confirmed by thick blood smear result divided by number of malaria episodes at baseline.
Crude species specific Day 28 cure rate	Number of episodes in patients with clearance of parasites within 7 days, and without subsequent return of parasites during the 28-day period following the first dose, as confirmed by thick blood smear result divided by number of malaria episodes that tested positive for a given species.
PCR adjusted Day 28 cure rate	Number of episodes in patients with clearance of parasites within 7 days, and without subsequent return of the original infection during the 28-day period following the first dose, as confirmed by thick blood smear result and PCR genotyping divided by number of malaria episodes at baseline.
**Criteria for fever**	
Fever	Fever will be defined as axillary temperature ≥37.5°C, or rectal/oral/tympanic temperature ≥38°C.
**Criteria for clinical hepatic safety events**	
Hepatic safety events (Yes/No)	An episode will be classified as "hepatic event = Yes", if an episode with at least one symptom present based on the eCRF field of "Assessment of Hepatic Symptoms" AND/OR hepatic related AEs confirmed biologically (i.e. decoded AEs from AE data)
**Criteria for grouping at inclusion/baseline**	
Abnormal LFTs	AST/ALT > 2xULN
Normal LFTs	AST and ALT ≤ 2xULN

7.1.1 Definitions relative to safety parameters

7.1.1.1 Definition of Adverse Event and Serious Adverse Event

An Adverse Event (AE) is a sign, symptom, syndrome, disease or biological anomaly suffered by a patient or a subject participating in this clinical study and receiving *Pyramax*. This term does not imply a causal relationship with the concerned treatment. Clinical signs typical of an acute malaria episode will not be considered AEs unless the healthcare personnel considers these events as exceptional due to their evolution, their seriousness, or another factor related to these events.

A **Serious Adverse Event** (SAE) is an adverse event which:

- causes death or
- is life-threatening or
- necessitates hospitalisation or prolongs hospitalisation or
- results in persistent or significant disability/incapacity or
- is a congenital defect or malformation
- is another medically important event
- constitutes a possible Hy's Law

7.1.1.2 Adverse Event of Special Interest (AESI)

AESIs in this study can be related to

- **Hepatotoxicity**

  Hepatic AESIs should be reported as follows

  a) For patients with normal LFTs at baseline:

  - Present with fatigue, nausea, abdominal pain, itching or signs of jaundice such as:
    - dark urine,
    - putty or mastic coloured stools,
    - jaundice (yellowing of the whites of the eyes or skin).

    and

    - ALT or AST >3 x ULN

  b) For patients with baseline ALT/AST >2xULN:

  - Present with fatigue, nausea, abdominal pain itching or signs of jaundice such as:
    - dark urine,
    - putty or mastic coloured stools,
    - jaundice (yellowing of the whites of the eyes or skin).

        - ALT/AST >2 x baseline value

- **Hypersensitivity**
    - Hypersensitivity AESIs should be reported if patients show signs of hypersensitivity soon after treatment with *Pyramax* (particularly on repeat treatment) such as:
        - Flushing
        - The appearance of wheals / urticaria
        - Breathlessness
        - Faintness and/or fall in blood pressure

7.2 Handling of missing data and outliers

7.2.1 Missing data analysis methods

Patients with missing efficacy endpoint at Day 28 will be considered as failure in the m-ITT population and excluded from the PP population. For the multivariate logistic regression model, complete case analysis will be conducted if the missing values are <5% for all the variables included in the model. Otherwise, missing baseline data will be imputed using random normal function (i.e. mean + [SD*RANNOR(seed)]) for continuous variables or using simple imputation of binomial distribution for binary variables/multinomial distribution for categorical variables (see Appendix III for sample SAS code for imputation).

7.2.2 Handling of missing or incomplete dates

7.2.2.1 Missing or incomplete dates for prior and concomitant medications

If a medication cannot be classified due to missing/incomplete date, it will be classified as concomitant.

7.2.2.2 Missing or incomplete dates for adverse events

If the onset date of an AE is partially or completely missing such that it cannot be determined if the event onset was prior to start of study drug, the AE will be assumed to be treatment-emergent, unless the AE stop date is before the date of first dose.

7.2.3 Handling of adverse events with no investigator causality

Adverse events for which no causality has been provided by the investigator will be considered as related to study medication. Note that events will be considered related if either the investigator or medical monitor considers that there is at least a possible relationship to the study medication.

8 STATISTICAL METHODS

8.1 General statistical conventions

All statistical procedures will be completed using SAS version 9.3 or higher.

The statistical analysis will be mainly descriptive at episode level. The estimates of the incidence of adverse events will be based on crude rates. All estimates will be complemented with appropriate 95% confidence intervals.

Continuous variables will be summarized using descriptive statistics, including number of malaria episodes (n), mean, median, standard deviation (SD), first quartile (Q1), third quartiles (Q3), minimum and maximum.

For categorical variables, summaries will include counts of episodes (frequencies) and percentages. Percentages will be rounded to one decimal place.

For summary purposes, baseline will be defined as the last available pre-dose value.

All subject (episode-level) data, including those derived, will be presented in individual subject data listings. Unless otherwise stated, unscheduled visit results will be included in date/time chronological order, within patient listings only. All listings will be sorted by investigational site, patient/episode number, date/time and visit. Unless otherwise stated, data listings will be based on enrolled population.

All parameters will be summarized by subjects with abnormal LFTs (AST or ALT >2xULN), normal LFTs (AST and ALT ≤2xULN) at inclusion/enrolment for a specific episode and by total, unless otherwise specified.

8.2 Subject disposition

Subject disposition information will be summarized at episode level with normal and abnormal LFTs at inclusion for a specific episode and by total. The number and percent of screened episodes, screen failures and reason for screen failure, enrolled into the study (meets inclusion exclusion criteria), included in the m-ITT, Safety (1:5 matched cohort as well as 1:1 matched cohort) and PP populations, who completed the study and who withdrew early from the study for a specific malaria episode will be presented. The primary reason for early withdrawal will also be tabulated.

The number of malaria episodes will be used as the denominator for the percentage calculation. Subject disposition will be listed. In addition, listings of subjects who did not met inclusion exclusion criteria will also be provided.

8.3 Protocol deviations

All protocol deviations identified as per the protocol will be summarized by malaria episodes in patients with abnormal LFTs (AST/ALT >2xULN) and normal at inclusion and by total.

A listing will include the inclusion/exclusion criteria violated at enrolment visits as well as other protocol

deviations identified based on data recorded on the eCRF.

The number of patients excluded from m-ITT, Safety and per protocol populations for a specific episode and reasons for exclusion will be summarized by abnormal and normal LFTs at inclusion and overall. An additional similar table on episode level will be prepared.

Population membership details will be listed, including reason for exclusion from each population.

8.4 Demographics and baseline characteristics

No formal comparison on demographics and baseline characteristics will be done. Demographic and other baseline characteristics will be summarized descriptively using safety population by malaria episodes with abnormal LFTs (AST/ALT >2xULN) and normal at inclusion and by total.

8.4.1 Demographics

Continuous demographic variables such as age, height, weight, BMI and mid-upper arm circumference (MUAC) will be summarized descriptively. Age category (<1 year, ≥1 year), sex, MUAC (for <6 year subjects; <115 mm, ≥115 mm) and BMI (for ≥6 year subjects; <18.5 kg/m^2, 18.5 - <25 kg/m^2, ≥25 kg/m^2) and subjects malnourished/ non-malnourished (refer section 7.1) will be summarized using frequency table. All demographic variables collected at enrolment of each episode will be used for summary.

In addition, a separate table summarizing the number of episodes/per patient that occurred during the study will be provided. Descriptive summary as well as frequencies (1, 2, 3, 4…, episodes) will be reported.

Subject listing will also be provided for demographic variables as per the data collected on CRF

8.4.2 Other baseline characteristics

For each episode, baseline parasite count, baseline fever (Yes/No) and HIV status will be summarized using frequency table. In addition, data listing will also be provided.

8.4.3 Medical history

A summary of general medical history at the time of inclusion/enrolment for the safety population will be presented by system organ class (SOC) and preferred term (PT) using Medical Dictionary for Regulatory Activities (MedDRA) Version 20.0 or higher.

Subject listing will be provided for relevant medical history conditions as per the data collected on CRF.

8.4.4 Prior and concomitant medications

Medications used in this study will be coded by using the latest available version of the World Health Organization Drug Dictionary Enhanced (WHODDE).

Prior medications and concomitant medications will be summarised by Anatomical Therapeutic Chemical (ATC) Level 2 classification and Preferred Name by treatment regimen for the safety population through frequency distributions and percentages. The same drug can appear in more than one ATC group.

In addition, prior and concomitant medications taken for malaria will also be provided.

A data listing will be provided which will include the start date, stop date, reason for use and indication.

8.5 Extent of exposure

8.5.1 Treatment compliance

Study drug exposure/compliance summaries will be based on the Safety Population.

Exposure to study drug will be summarised with the following measures:

- ✓ Number and percentage of episodes in patients who received sachets/tablets
- ✓ Distribution of number of doses (sachets/ tablets) received. The number of doses will include doses that were vomited (included doses that were repeated on Day 0)
- ✓ Number and percentage of episodes in patients who vomited the first dose
- ✓ Number and percentage of episodes in patients who received a repeated dose for the first dose
- ✓ Number of episodes in patients who received the planned dose (according to Subject), a higher dose or a lower dose (according to Subject)

Compliance will be assessed based on the sachets/tablets presented to the community health worker (CHW) during home visits on day 7.

8.6 Safety analyses

This section describes the safety analyses that will be conducted on the treatment period, i.e., the safety analyses on all data collected during the treatment period and all data collected in subjects who dropped-out during the treatment period.

Safety analyses will be conducted on the Safety Population (treated episodes) and will be performed for all safety variables specified below.

All safety data will be summarized by group (malaria episodes having abnormal LFTs and with normal LFTs) at enrolment of each episodes and for all the episodes.

No statistical test will be performed.

Primary Objective

All hepatic safety events (Yes/No) will be summarized by episodes in patients having abnormal baseline LFTs (AST/ALT >2xULN). In addition, Clopper-Pearson 95% confidence interval will also be provided.

Secondary Objective

All hepatic safety events (Yes/No) will be summarized by episodes in patients having abnormal LFTs (AST/ALT >2xULN) at enrolment versus a 1:1 matching cohort of episodes in patients with normal LFTs (AST and ALT ≤2xULN) at enrolment/inclusion. The 1:1 matching cohort is selected based on the propensity scores (see below).

Propensity Score (PS) using multivariate logistic regression model (see Appendix III) will be performed to select the matched episode with normal LFTs for every abnormal LFTs (i.e. nearest neighbour PS with a matching tolerance PS difference of 0.2 or as appropriate between abnormal and normal LFTs at inclusion will be used to select the matched pairs[5]) without replacement. This model will include the group (normal and abnormal LFTs) as outcome and demographic characteristic such as age, sex (male, female), nutritional status (Malnourished/ Non-malnourished), weight, height, fever (Yes/No), HIV status (Yes/No), medical history other than malaria (Yes/No) as independent variables. The model will also include baseline systolic blood pressure, diastolic blood pressure, heart rate, temperature, haemoglobin, ALT and AST. Only those independent variables will be kept in the model used to estimate PS who have an effect on the outcome variable on the 5% significance level. PS summary statistics will be checked in matched pairs to confirm if mean PS is similar for both groups.

Once PS has been computed for each of the episode, a multivariate logistic regression model will be used to find association between the occurrence of hepatic events and various demographic characteristic such as abnormal/normal LFTs (AST/ALT >2xULN) at inclusion, age, sex (male, female), nutritional status (Malnourished/ Non-malnourished), weight, height, fever (Yes/No), HIV status (Yes/No), medical history other than malaria (Yes/No) as fixed effects and propensity score as a covariate. The model will also include baseline systolic blood pressure, diastolic blood pressure, heart rate, temperature, haemoglobin, ALT and AST. The model for the regression analysis will be adapted accordingly since episodes are not independent within same patients. Other models with different data distribution may be tested, if applicable.

In addition, 1:5 matched pairs (strata) will be identified using the PS matching approach as specified above (using an algorithm conducted independently from the one used to select the 1:1 matched pairs) and Conditional logistic regression model (see Appendix III) will be used to find association between the occurrence of hepatic events and baseline abnormal/normal LFTs (AST/ALT >2xULN) along with other demographic characteristic such as age, sex (male, female) nutritional status (Malnourished/Non-malnourished), weight, height, fever (Yes/No), HIV status and medical history other than malaria (Yes/No). The model will also include baseline systolic blood pressure, diastolic blood pressure, heart rate, temperature, haemoglobin, ALT and AST. The number of covariates in the model will be reduced when appropriate, since PS matching is expected to balance the characteristics between baseline abnormal/ normal LFTs groups. Matched odds ratio and its relevant 95% Wald confidence interval and p-value will be reported. This analysis will be performed based on the 1: 5 matched Safety population.

In addition, all the reported hepatic events will be summarized by System Organ Class (SOC) and Preferred Term (PT) by 1:1 matching cohort and for actual abnormal/normal LFTs (AST/ALT >2xULN) at inclusion groups.

Additional subgroup analyses (descriptive summary) based on the m-ITT population will be performed as detailed in section 8.6.6. A separate listing of hepatic events (including causality and related events) will be provided.

8.6.1 Adverse events

All Adverse events (AEs) will be classified by Primary System Organ Class (SOC) and Preferred Term (PT) according to MedDRA Version 20.0 or higher.

In summaries by SOC and PT, adverse events will be sorted according to the alphabetical order of SOC and within each SOC the PT will be sorted by decreasing frequency.

Adverse Event (AE): arising between the first administration of study medication and the last study visit. **Serious Adverse Event (SAE):** arising between enrolment and last study visit.

AEs incidences will be computed on the following classes of AEs:

- All AEs
- Serious AEs
- AEs of special interest (AESIs)
- AEs which caused early discontinuation of *Pyramax*
- AEs related to *Pyramax* treatment
- SAEs related to *Pyramax* treatment
- AESI related to *Pyramax* treatment

An overall summary of AEs will be provided. The total number and proportion of episodes experiencing any AEs, Serious AEs, AESI (refer section 7.1.1.2), AEs leading to early discontinuation of *Pyramax*, AEs related to *Pyramax,* serious AEs related to *P*yramax and AESI related to *Pyramax* will be presented by Normal/Abnormal LFTs at enrolment/inclusion and total.

Additionally, all AEs, serious AEs, AESI (refer section 7.1.1.2), AEs leading to early discontinuation of *Pyramax*, AEs related to *Pyramax* and serious AEs related to *Pyramax* will be summarised by SOC and PT will be presented by normal/abnormal LFTs at enrolment and total. All AEs will also be summarised separately by maximum severity grade for each SOC and PT and by normal/abnormal LFTs at enrolment/inclusion and total.

Where an episode has the same adverse event, based on preferred terminology, reported multiple times in the treatment period, the episode will only be counted once at the preferred terminology level in adverse event frequency tables.

Where an episode has multiple adverse events within the same system organ class in the treatment period, the episode will only be counted once at the system organ class level in adverse event frequency tables.

When reporting adverse events by severity, in addition to providing a summary table based on the event selection criteria detailed above, summary table will also be provided based on the most intense event during the treatment period - independent of relationship to study treatment.

Compliance will be computed for each patient‘s episode as the ratio between the number of tablets or sachets actually taken and the number of tablets or sachets that the patient should have taken. Two groups of patients shall be described: compliant = 100% treatment compliance and noncompliant = other cases.

In addition, the above classes of AEs will be compared by compliant vs non-compliant patients.

8.6.2 Clinical laboratory evaluations

Haematology and blood chemistry will be conducted at Screening/Inclusion Day 0 and in case of symptoms confirmed by a clinician after referral at the health centres on Day 7 ±1, Day 28 ±2 and during unforeseen visit (if any).

Descriptive statistics for all the episodes will be presented for quantitative clinical laboratory parameters at each scheduled time-point. Similarly, changes from baseline will be summarised. Values outside the normal range will be categorised as H (above the normal range) or L (below the normal range) based on the laboratory's reference range and these will be flagged in the individual data listings along with the Investigator's assessment. Unforeseen visit information will be included only in listings.

In addition, the number of episodes with laboratory values for liver function and enzymes ≥2*ULN, ≥3*ULN, ≥5*ULN and ≥10*ULN will be presented. Shift tables displaying baseline values vs. worst post-baseline values will also be prepared. Individual data listings of these laboratory values will also be provided.

In particular, a listing containing individual data clinical laboratory values outside the normal reference ranges will be provided. This listing will include data from scheduled and unscheduled time points.

Comparisons of total bilirubin, ALT and AST values between first and second malaria episodes will be displayed by using evaluation of drug induced serious hepatotoxicity (eDISH) graphs.

Pregnancy test results will be listed.

8.6.3 Vital signs

Vital sign parameters such as heart rate, systolic blood pressure (SBP), diastolic blood pressure (DBP) will be performed at Inclusion Day 0. Body temperature will be assessed at Inclusion Day 0, Day 7, Day 28 and any unforeseen visit. Descriptive statistics for all the episodes will be provided for all absolute vital sign measurements. Change from baseline will be provided for body temperature at Day 7, Day 28 and any unforeseen visit.

A data listing of vital signs will also be provided.

8.6.4 Physical examinations

Physical examination will be performed at Inclusion Day 0. All physical examination data and abnormalities will be listed by episode and body system.

Malaria signs and symptoms will be summarized by count and percentage. Individual listing will also be provided.

8.6.5 Electrocardiograms

Not applicable

8.6.6 Subgroup analysis

The subgroup analysis is part of safety endpoints and is included under the corresponding section.

All AEs and hepatic events will be summarized by demographic characteristics such as

- age (<1 years, ≥1 year),
- sex (male, female).

In addition, the AEs and hepatic events will also be summarized by the subgroups

- HIV status
- Viral hepatitis status (hepatitis A, B, C, delta [if hepatitis B positive] and E)
- Any abnormality detected on the hepatitis panel (Yes, No)
- Nutritional status (Malnourished/ Non-malnourished)
    - Malnourished Subjects with age <6 years: Mid-upper arm circumference <115 mm; Subjects with age 6-19 ≥: BMI <-3 SD based on the BMI data of z score tables for boys and girls; Subjects with age >19 year; BMI <18.5 kg/m2
    - Non-malnourished
- Weight (5 - <20 kg and ≥20 kg)
- Use of concomitant medications (i.e. Paracetamol, herbal medicine or antiretroviral drugs).
- Compliance category
    - compliant = 100% treatment compliance;
    - non-compliant = other cases.
- Country and by Center
- Retreated patients

8.7 Efficacy analyses

8.7.1 Analysis of secondary efficacy endpoints

PCR adjusted and unadjusted day 28 cure rate and cure rate by species will be summarized by count and percentage for the patients for a specific episode. Clopper Pearson 95% confidence interval will also be provided.

This will be repeated for the subgroups of Country, dosing (granules and tablets), malnourished subjects as well as those aged <1 years. This analysis will be carried out using m-ITT and PP population.

Time between malaria episodes in days (refer section 7.1) will be summarized by the number of subjects (n), mean, standard deviation, median, min, and max.

Frequency summaries will be provided for the number of malaria episodes before and after Day 28.

For the analysis of time between malaria episodes, number of malaria episodes before and after Day 28 will be presented only for total.

8.8 Interim analysis

No formal interim analysis planned for this study.

Interim data will be assessed under the DSMB Charter by the independent DSMB.

9 CHANGES TO PLANNED ANALYSIS FROM STUDY PROTOCOL

All analysis planned in the protocol are followed.

10 REFERENCES

1. ICH Topic E3: Structure and Content of Clinical Study Reports (CPMP/ICH/137/95- adopted December 1995).
2. ICH Topic E9: Statistical Principles for Clinical Trials (CPMP/ICH/363/96 – adopted March 1998).
3. World Health Organisation: Guidelines for the treatment of malaria (third edition), 2015 [http://www.who.int/malaria/publications/atoz/9789241549127/en/].
4. http://www.who.int/growthref/who2007_bmi_for_age/en/
5. A General SAS® Macro to Implement Optimal N:1 Propensity Score Matching Within a Maximum Radius [http://support.sas.com/resources/papers/proceedings17/0812-2017.pdf]

11 APPENDICES

Appendix I

Definition of Severe Malaria

WORLD HEALTH ORGANISATION CRITERIA 2014

Clinical features of severe malaria in children in high transmission area:

**Group 1**	Prostrate children (prostration is the inability to sit upright in a child normally able to do so or to drink in the case of children too young to sit). Three subgroups of increasing severity should be distinguished:<br>Prostrate but fully conscious<br>Prostrate with impaired consciousness but not in deep coma<br>Coma (the inability to localise a painful stimulus)<br>Respiratory distress (acidotic breathing):<br>Mild – sustained nasal flaring and/or mild intercostal indrawing (recession)<br>Severe – the presence of either marked indrawing (recession) of the bony structure of the lower chest wall or deep (acidotic) breathing<br>Shock compensated or decompensated (see definition above)
**Group 2**	Children who, although able to be treated with oral antimalarials, require supervised management because of the risk of clinical deterioration but who show none of the features of group 1 (above)*. These include children with any of the following:<br>Haemoglobin <5 g/dl or haematocrit < 15%<br>2 or more convulsions within a 24-h period<br>Haemoglobinuria (blackwater)<br>Jaundice
**Group 3**	Children who require parenteral treatment because of persistent vomiting but who lack any specific clinical or laboratory features of groups 1 or 2 (above)

*If parasite counts are immediately available, a parasitaemia over 10% should be included in group 2.
Children are defined as <12 years old.

Clinical features of severe malaria in adults:

Group 1	Adults at increased risk of dying immediately who require parenteral antimalarials and appropriate supportive therapy<br>Prostrated or obtunded adults (prostration is the inability to sit or to drink). Four subgroups of increasing severity should be distinguished:<br>Prostrate but fully conscious<br>Prostrate with impaired consciousness but not in deep coma (GCS > 11)<br>Confusion and agitation (GCS > 11)<br>Coma (the inability to localise a painful stimulus) (GCS < 11)<br>Respiratory distress (acidotic breathing)<br>Mild – sustained nasal flaring and/or mild intercostal indrawing (recession)<br>Severe – the presence of either marked indrawing (recession) of the bony structure of the lower chest wall or deep (acidotic) breathing<br>Shock (hypotension:systolic BP < 80 mmHg)<br>Anuria<br>Significant upper gastrointestinal haemorrhage
Group 2	Adults who, although able to be treated with oral ACTs, require supervised management because of the risk of clinical deterioration but who show none of the features of group 1 (above)*. This group includes adults with any of the following:<br>Haemoglobin <7 g/dl or haematocrit <20%<br>One or more convulsions within a 24-h period<br>Haemoglobinuria (blackwater)<br>Jaundice
Group 3	Adults who require parenteral treatment because of persistent vomiting but who lack any specific clinical or laboratory features of groups 1 or 2 (above)

*If parasite counts are immediately available a parasitaemia over 4% should be included in group 2.

Appendix II

Adverse Events of Special Interests: Drugs and the Liver

Checklist for Serious Liver Reactions:

The following liver reactions have to be considered as potentially serious:

Possible Hy's law case is defined as a subject with any value of ALT or AST >3xULN together WITH an increase in bilirubin to a value > 2xULN (>35% direct) and NOT associated to an ALP value > 2xULN.

Other definitions of hepatic adverse events of special interest (AESIs):

For patients with normal LFTs at baseline:

- Present with fatigue, nausea, abdominal pain itching or signs of jaundice such as:
  - dark urine,
  - putty or mastic coloured stools,
  - jaundice (yellowing of the whites of the eyes or skin).

and

- ALT or AST >3 x ULN.

For patients with baseline ALT/AST >2xULN:

- Present with fatigue, nausea, abdominal pain itching or signs of jaundice such as:
  - dark urine,

- o putty or mastic coloured stools,
- o jaundice (yellowing of the whites of the eyes or skin).

and

- ALT/AST >2 x baseline value.

In the event of biological signs of hepatotoxicity associated with clinical symptoms/suspected Drug Induced Liver Injury (DILI), blood will need to be taken to perform the following additional tests (full hepatitis panel): This blood sample shall be sent as soon as possible to the central lab for further analysis.

- Hepatitis A, B, C (Anti-HAV IgM, Anti-HBc IgM, HBsAg, and hepatitis C RNA),
- Hepatitis E IgM antibody,
- Cytomegalovirus (CMV) testing polymerase chain reaction (PCR) testing,
- pp65 antigen, or IgM antibody,
- Epstein Barr virus (EBV) viral capsid antigen IgM antibody,
- Serum creatinine phosphokinase (CPK) and lactate dehydrogenase (LDH),
- Alkaline phosphatase (if not already collected),
- Serum transferrin saturation and serum ferritin (diagnosis of hemochromatosis),
- Liver biosynthetic capacity: albumin and prothrombin time (+ prealbumin, serum ceruloplasmin, procollagen III peptide, a-1-antitrypsin and a-feto protein, when possible).

Appendix III

SAS code for Propensity Score:

```
PROC LOGISTIC DATA = xxx descending;
CLASS sex <other categorical variables>;
MODEL binary_out  (event = “Abnormal”) = age sex weight <other baseline characteristics>;
OUTPUT OUT= Propen pred=prob_grp;
RUN;
```

Notes:

- binary_out is the binary outcome variable (e.g. LFT with normal or Abnormal condition)

SAS code for Conditional logistic regression:

```
PROC LOGISTIC DATA = xxx;
CLASS sex <other categorical variables>;
MODEL binary_out (event=”1”) = hepatic_event age sex weight <other variables>;
STRATA id;
RUN;
```

Notes:

- The variable binary_out is used to determine whether the given episode is a case (=1, abnormal LFTs) or a control (=0, normal LFTs). Note that the data set is created so that all the cases have the same event time and the controls have later censored times (i.e. “0”).
- Hepatic_event represents the grouping variable (Yes/No for Occurrence of hepatic events)
- The matching variable id is used in the STRATA statement so that each unique id value defines a stratum.

SAS code for Kaplan-Meier estimates and Log rank test:

```
 PROC LIFETEST DATA=dataset METHOD = KM PLOTS = (SURVIVAL (ATRISK));
   TIME time*Event(0);
   STRATA Trt / TEST = (logrank);
   SURVIVAL OUT=XXXX STDERR TIMELIST= T1 T2 T3 T4…;
 RUN;
```

Notes:

- Time in days or months;
- Event represents the censoring indicator (0 = censored);
- Trt represents the treatment variable/group
- The Kaplan-Meier Estimate at 1-month, 3-months, 6-months, 9-months etc., will be derived using the TIMELIST= option in PROC LIFETEST

SAS code to Impute continuous variables:

```
 DATA dataset;
   SET dataset;
   IF var_x NE . THEN new_var = var_x;
   ELSE new_var = mean(var_x) + [SD(var_x)*RANNOR(seed)];
RUN;
```

Notes:

- Var_x is the continuous variable which need to be imputed
- Fix the seed to 123

SAS code to Impute (simple) categorical variables:

```
 PROC MI DATA=dataset SEED = 123 NIMPUTE = 1 SIMPLE OUT =Out_dataset;
   CLASS c_var1 c_var2 c_var3..;
   MONOTONE LOGISTIC (impute  var = var1 c  var1 var2 c  var2 var3 c  var3.../ DETAILS)
/*  MONOTONE DISCRIM (impute_var = var1 c_var1 var2 c_var2 var3 c_var3.../ DETAILS) */
/*  FCS LOGISTIC (impute_var = var1 c_var1 var2 c_var2 var3 c_var3.../ DETAILS) */
```

```
/*  FCS DISCRIM (impute  var = var1 c  var1 var2 c  var2 var3 c_var3.../ DETAILS) */
    VAR var1 c_var1 var2 c_var2  var3 c var3...impute_var;
  RUN;
```

Notes:

- Impute_var is the variable (binary/nominal) which need to be imputed
- Use LOGISTIC for binary variable or DISCRIMINANT for nominal variables for imputation
- In case of MONOTONE missing pattern, please use MONOTONE LOGISTIC for binary/ordinal variables and MONOTONE DISCRIM for nominal variables
- In case of NON-MONOTONE missing pattern (i.e. arbitrary), please use FCS LOGISTIC for binary/ordinal variables and FCS DISCRIM for nominal variables
- Reference: https://pharmasug.org/proceedings/2017/SP/PharmaSUG-2017-SP01.pdf

Statistical Analysis Plan (SAP)
Amendment 1

**Protocol Title:**	Phase IIIB/IV Cohort Event Monitoring study to evaluate, in real life setting, the safety and tolerability in malaria patients of the fixed-dose Artemisinin-based Combination Therapy Pyramax® (pyronaridine-artesunate).
**Protocol Number:**	SP-C-021-15
**Protocol Version, Date**	Final Version 8.0 Date: 13SEP2017
**ICON ID:**	3037/0007
**Document Version, Date:**	Amendment 1, Final Version 1.0, 28Jun2019

Prepared by:

ICON Clinical Research Services

On behalf of:

SHIN POONG PHARMACEUTICALS/MMV

Confidentiality statement:

- The information provided in this document is strictly confidential.
- The recipients of the SAP must not disclose the confidential information contained within this document or any related information to other persons without the permission of the sponsor.
- In addition, the recipients of the SAP must keep this confidential document in a controlled environment which prevents unauthorized access to the document.

SIGNATURE PAGE

Prepared at ICON Clinical Research by:

Rolf Dietl, Sr. Biostatistician I	Date (DD Mmm YYYY)

Reviewed at ICON Clinical Research by:

Rolf Hoevelmann, Senior Manager Biostatistics	Date (DD Mmm YYYY)

Approved at SHIN POONG by:

Jang Sik Shin, Pyramax Project Leader, Shin Poong Pharmaceuticals	Date (DD Mmm YYYY)

Statistical Analysis Plan (SAP)
Amendment 1

Approved at MMV by:

Dr Stephan Duparc, Chief Medical Officer	Date (DD Mmm YYYY)

Approved at University of Tübingen by:

Dr Michael Ramharter, Coordinating Principal Investigator	Date (DD Mmm YYYY)

TABLE OF CONTENTS

SIGNATURE PAGE 2
TABLE OF CONTENTS 4
1 INTRODUCTION 5
2 ADDITIONAL RULES FOR EFFICACY ANALYSES 6
3 CHANGES IN FINAL SAP FROM 28JAN2019 9

Statistical Analysis Plan (SAP)
Amendment 1

1 INTRODUCTION

This Statistical Analysis Plan (SAP) Amendment 1 describes additional rules and clarifications for the efficacy analysis of the SP-C-021-15 study developed during the preparation of the efficacy analysis datasets and efficacy tables programming as well as necessary modifications and/ or changes in the final version of the SAP dated 28 Jan 2019.

2 ADDITIONAL RULES FOR EFFICACY ANALYSES

The table below displays different potential scenarios of malaria parasites related to PCR genotyping and the corresponding assessments/ rules regarding treatment outcome crude ACPR (adequate clinical and parasitological response) and treatment outcome PCR-adjusted ACPR for the PP and the mITT analysis set. These assessments will be taken into account for the respective efficacy analyses for this study.

	Potential scenario	PP		mITT		Time period
		Treatment Outcome Crude ACPR28*	Treatment Outcome PCR-adjusted ACPR28	Treatment Outcome Crude ACPR28	Treatment Outcome PCR-adjusted ACPR28	Number means the day included. For ex: From D26 means Day 26 included
1	Completed up to Day 28 without re-emergence (re-infection/ recrudescence) of parasites after initial clearance, thus ACPR28.	Success	Success	Success	Success	From D26 to D30
2	Missing assessment on Day 28, but parasite free (No *P. falciparum* asexual parasites) after Day 28	Success	Success	Success	Success	After D30
3	Missing assessment on Day 28, no more assessments thereafter.	Excluded	Excluded	failure	failure	Last assessment before D26
4	Missing assessment on Day 28, recrudescence (confirmed by PCR) at first assessment after Day 28	Failure	Failure	Failure	Failure	Recrudescence after D30
5	Missing assessment on Day 28, re-infection (confirmed by PCR) at first assessment after Day 28.	Failure	Success	Failure	Success	Re-infection after D30
6	Re-infection before Day 28 (thus on or after Day 7 to before 28)	Failure	Excluded	Failure	Failure	From D6 to D25
7	Re-infection on Day 28.	Failure	Success	Failure	Success	From D26 to D30
8	Recrudescence before or on Day 28 (thus on or after Day 7 to 28)	Failure	Failure	Failure	Failure	From D6 to D30
9	Re-emergence before or on Day 28 but PCR 'uninterpretable' or missing.	Failure	Excluded	Failure	Failure	From D6 to D30

10	Late clinical failure from Day 4 to Day 6.	Failure	Failure	Failure	Failure	Before D6 except if we have an unforeseen visit between D4 and D6
11	Early treatment failure (Day 1 to Day 3)	Failure	Failure	Failure	Failure	Before D6 except if we have an unforeseen visit between D1 and D3
12	Other *Plasmodium* species before Day 28 (in the absence of *P. falciparum*).	Excluded	Excluded	Failure	Failure	From D6 to D25
13	Other *Plasmodium* species on Day 28 (in the absence of *P. falciparum*).	Success	Success	Success	Success	D26 to D30
14	Other *Plasmodium* species before or on Day 28 (in the presence of *P. falciparum*), PCR missing, 'negative' or 'uninterpretable'.	Failure	Excluded	Failure	Failure	From D6 to D30
15	Prematurely discontinued from the study before Day 28 and SoC.	Failure	Excluded	Failure	Failure	< D28<br><br>FromD6 to D25
16	Prematurely discontinued from the study before Day 28 and no record of SoC.	Excluded	Excluded	Failure	Failure	< D28<br><br>FromD6 to D25
17	Completed up to Day 28. Have records in MB (Microbiology Specimen dataset), Infection present='N', but PCR wasn't done and has 'SAMPLE TAKEN BUT NOT SENT FOR PCR ANALYSIS '.	Success	Success	Success	Success	From D26 to D30

*) ACPR28: Adequate clinical and parasitological response on Day 28

General rules:

1) The cases with PCR result = negative will be ignored.

2) If a patient is cured at D42 and did not take antimalarial drugs since D3 and if there was no re-appearance of parasites between D3 and D42, he/ she should be considered as cured in PP and ITT.

3) If a patient has missing assessment on Day 28 but has an assessment on Day 42 and has taken antimalarial drugs between D28 and D42 (or at any time prior to D42), he/ she should be excluded from the PP population and be considered as failure in the ITT population.

4) The information leaflet “Study Guideline updates” version 4 dated 12Oct2018 will be followed.

Explanations related to scenarios above:

To 11: D1 to D3 means at any time between D1 and D3 and even before D6. If a patient has signed of early treatment failure as defined by the WHO, he/ she should be considered as failure in both PP and ITT and without PCR as PCR is not done before D7. The WHO definition of treatment failure is as follows: 'Treatment failure is the inability to clear parasites from a patient’s blood or to prevent their recrudescence after the administration of an antimalarial.’

To 15: After PCR adjustment even if the result shows it was a new infection as the patient has received an antimalarial drug, we cannot be sure he/ she would not have had a recrudescence between D28 and therefore he/ she will have to be excluded.

To 17: If a patient is negative at D28 and/ or D42, the sample for PCR that has been taken before the result of the slide, needs to be destroyed. If it is sent by mistake, it must not be analysed. If it is analysed by mistake, the result must not be written in the CRF as it is without sense. In case that this D28 or D42 PCR result shows there is a slide, it would be impossible to interpret the results.

3 CHANGES IN FINAL SAP FROM 28JAN2019

The following topics will be updated as follows:

3.1 PCR adjusted Day 28 cure rate (section 7.1)

Old version:

Variables	Formula
**Cure rate**	
PCR adjusted Day 28 cure rate	Number of episodes in patients with clearance of parasites within 7 days, and without subsequent return of the original infection during the 28-day period following the first dose, as confirmed by thick blood smear result and PCR genotyping, in case that parasites have been detected, divided by number of malaria episodes at baseline.

3.2 Definition of per-protocol population (section 6.5)

The PP population will consist of all the malaria episodes in m-ITT population meeting pre-defined criteria as follows:

1. Subjects with scenarios #3, 12, 16 excluded from the PP population.
2. Subjects having completed a full course of study medication and having known efficacy endpoints for the specific malaria episode.
3. Subjects who do not vomit after the study drug administration (except where a subject may vomit after the first drug administration and have a repeat full dose - in this case, the repeated dose should not be vomited).
4. No prior or concomitant medication (except paracetamol) which may interfere with the treatment outcome, up to Day 28. A list of patients which have to be excluded will be provided before the final analysis" will be added to condition 3. A medical review will be performed to confirm the exclusion of a subject from the PP population at the time of final analysis and be considered as failure in the ITT population.

The definition will be modified as follows:

- Condition 1 will be removed and the rules above will be followed instead.

- Condition 3: Additional text will be included due to consistency purposes.

Statistical Analysis Plan (SAP)
Amendment 2

**Protocol Title:**	Phase IIIB/IV Cohort Event Monitoring study to evaluate, in real life setting, the safety and tolerability in malaria patients of the fixed-dose Artemisinin-based Combination Therapy Pyramax® (pyronaridine-artesunate).
**Protocol Number:**	SP-C-021-15
**Protocol Version, Date**	Final Version 9.0 Date: 15MAR2019
**ICON ID:**	3037/0007
**Document Version, Date:**	Amendment 2, Final Version 1.0, 08Nov2019

Prepared by:

ICON Clinical Research Services

On behalf of:

SHIN POONG PHARMACEUTICALS/MMV

Confidentiality statement:

- The information provided in this document is strictly confidential.
- The recipients of the SAP must not disclose the confidential information contained within this document or any related information to other persons without the permission of the sponsor.
- In addition, the recipients of the SAP must keep this confidential document in a controlled environment which prevents unauthorized access to the document.

SIGNATURE PAGE

Prepared at ICON Clinical Research by:

Rolf Dietl, Sr. Biostatistician I	Date<br>(DD Mmm YYYY)

Reviewed at ICON Clinical Research by:

Rolf Hoevelmann, Senior Manager Biostatistics	Date<br>(DD Mmm YYYY)

SIGNATURE PAGE

Approved at SHIN POONG by:

Jang Sik Shin, Pyramax Project Leader, Shin Poong Pharmaceuticals	Date (DD Mmm YYYY)

Approved at MMV by:

Dr Stephan Duparc, Chief Medical Officer	Date (DD Mmm YYYY)

Approved at University of Tübingen by:

Dr Michael Ramharter, Coordinating Principal Investigator	Date (DD Mmm YYYY)

TABLE OF CONTENTS

SIGNATURE PAGE 2
SIGNATURE PAGE 3
TABLE OF CONTENTS 4
1 INTRODUCTION 5
2 CHANGES IN FINAL SAP FROM 28JAN2019 6
3 CHANGES IN SAP AMENDMENT 1 FROM 01AUG2019 7

Statistical Analysis Plan (SAP)
Amendment 2

1 INTRODUCTION

This Statistical Analysis Plan (SAP) Amendment 2 describes additional rules and clarifications for the analysis of the SP-C-021-15 study discussed during the classification meeting held on 24 Oct 2019 and resulting from client comments on the dry run tables.

2 CHANGES IN FINAL SAP FROM 28JAN2019

The following changes will be performed:

1) The penultimate sentence of section 8.6.2 (Clinical laboratory evaluations) will be removed, since no eDISH graphs will be prepared due to non-availability of corresponding data.

Old version:

Comparisons of total bilirubin, ALT and AST values between first and second malaria episodes will be displayed by using evaluation of drug induced serious hepatotoxicity (eDISH) graphs.

New version (changes are underlined):

~~Comparisons of total bilirubin, ALT and AST values between first and second malaria episodes will be displayed by using evaluation of drug induced serious hepatotoxicity (eDISH) graphs.~~

2) As a result of reviewing the dry run tables, the definition of the subgroup regarding nutritional status will be changed.

Old version:

- Nutritional status (Malnourished/ Non-malnourished)
  - Malnourished Subjects with age <6 years: Mid-upper arm circumference <115 mm; Subjects with age 6-19 ≥: BMI <-3 SD based on the BMI data of z score tables for boys and girls; Subjects with age >19 year; BMI <18.5 kg/m2
  - Non-malnourished

New version (changes are underlined):

- Nutritional status (Malnourished/ Non-malnourished)
  - Malnourished Subjects with age <6 years: Mid-upper arm circumference <115 mm; Subjects with age 6-17 years: BMI <-3 SD based on the BMI data of z score tables for boys and girls; Subjects with age ≥18 years: BMI <18.5 kg/m2
  - Non-malnourished

3 CHANGES IN SAP AMENDMENT 1 FROM 01AUG2019

The following changes will be performed:

Old version:

The PP population will consist of all the malaria episodes in m-ITT population meeting pre-defined criteria as follows:

1. Subjects with scenarios #3, 12, 16 excluded from the PP population.
2. Subjects who do not vomit after the study drug administration (except where a subject may vomit after the first drug administration and have a repeat full dose - in this case, the repeated dose should not be vomited).
3. No prior or concomitant medication (except paracetamol) which may interfere with the treatment outcome, up to Day 28. A list of patients which have to be excluded will be provided before the final analysis" will be added to condition 3. A medical review will be performed to confirm the exclusion of a subject from the PP population at the time of final analysis and be considered as failure in the ITT population.

The definition will be modified as follows:

- Wording in condition 1 to 3 will be changed.
- Condition 4 will be added.

New version (changes are underlined):

The PP population will consist of all the malaria episodes in m-ITT population meeting pre-defined criteria as follows:

1. Episodes of subjects with scenarios #3, 12, 16 excluded from the PP population.
2. Episodes of subjects who do not vomit after the study drug administration (except where a subject may vomit after the first drug administration and have a repeat full dose - in this case, the repeated dose should not be vomited).
3. No prior or concomitant medication (except paracetamol) which may interfere with the treatment outcome, up to Day 28. A list of subjects which have to be excluded was provided before the final analysis" and will be added to condition 3 of the SAP. A medical review will be performed to confirm the exclusion of a subject from the PP population at the time of final analysis and be considered as failure in the ITT population.
4. All major protocol deviations identified during the classification meeting held on 24Oct2019 will be excluded from the PP population.

Statistical Analysis Plan (SAP)
Amendment 3

**Protocol Title:**	Phase IIIB/IV Cohort Event Monitoring study to evaluate, in real life setting, the safety and tolerability in malaria patients of the fixed-dose Artemisinin-based Combination Therapy Pyramax® (pyronaridine-artesunate).
**Protocol Number:**	SP-C-021-15
**Protocol Version, Date**	Final Version 9.0 Date: 15MAR2019
**ICON ID:**	3037/0007
**Document Version, Date:**	Amendment 3, Final Version 1.0, 25May2020

Prepared by:

ICON Clinical Research Services

On behalf of:

SHIN POONG PHARMACEUTICALS/MMV

Confidentiality statement:

- The information provided in this document is strictly confidential.
- The recipients of the SAP must not disclose the confidential information contained within this document or any related information to other persons without the permission of the sponsor.
- In addition, the recipients of the SAP must keep this confidential document in a controlled environment which prevents unauthorized access to the document.

SIGNATURE PAGE

Prepared at ICON Clinical Research by:

[redacted]

Rolf Dietl, Sr. Biostatistician I

Date
(DD Mmm YYYY)

Reviewed at ICON Clinical Research by:

[redacted]

Rolf Hoevelmann, Senior Manager Biostatistics

Date
(DD Mmm YYYY)

DocUUID : cf9d069c-4a8e-49d0-b0f2-7cdcea1f06b9

SIGNATURE PAGE

Approved at SHIN POONG by:

[redacted]

Jang Sik Shin, Pyramax Project Leader, Shin Poong Pharmaceuticals — Date (DD Mmm YYYY)

Approved at MMV by:

[redacted]

Dr Stephan Duparc, Chief Medical Officer — Date (DD Mmm YYYY)

Approved at University of Tübingen by:

[redacted]

(DD Mmm YYYY)

TABLE OF CONTENTS

SIGNATURE PAGE 2
SIGNATURE PAGE 3
TABLE OF CONTENTS 4
1 INTRODUCTION 5
2 CHANGES IN SAP AMENDMENT 1 FROM 01AUG2019 6

Statistical Analysis Plan (SAP)
Amendment 3

1 INTRODUCTION

This Statistical Analysis Plan (SAP) Amendment 3 describes corrections in the additional rules for the efficacy analyses of the SP-C-021-15 study written in the SAP amendment 1 dated 01Aug2019.

2 CHANGES IN SAP AMENDMENT 1 FROM 01AUG2019

The following changes will be performed:

Old version:

The table below displays different potential scenarios of malaria parasites related to PCR genotyping and the corresponding assessments/ rules regarding treatment outcome crude ACPR (adequate clinical and parasitological response) and treatment outcome PCR-adjusted ACPR for the PP and the mITT analysis set. These assessments will be taken into account for the respective efficacy analyses for this study.

	Potential scenario	PP		mITT		Time period
		Treatment Outcome Crude ACPR28*	Treatment Outcome PCR-adjusted ACPR28	Treatment Outcome Crude ACPR28	Treatment Outcome PCR-adjusted ACPR28	Number means the day included. For ex: From D26 means Day 26 included
1	Completed up to Day 28 without re-emergence (re-infection/ recrudescence) of parasites after initial clearance, thus ACPR28.	Success	Success	Success	Success	From D26 to D30
2	Missing assessment on Day 28, but parasite free (No *P. falciparum* asexual parasites) after Day 28	Success	Success	Success	Success	After D30
3	Missing assessment on Day 28, no more assessments thereafter.	Excluded	Excluded	failure	failure	Last assessment before D26
4	Missing assessment on Day 28, recrudescence (confirmed by PCR) at first assessment after Day 28	Failure	Failure	Failure	Failure	Recrudescence after D30
5	Missing assessment on Day 28, re-infection (confirmed by PCR) at first assessment after Day 28.	Failure	Success	Failure	Success	Re-infection after D30
6	Re-infection before Day 28 (thus on or after Day 7 to before 28)	Failure	Excluded	Failure	Failure	From D6 to D25
7	Re-infection on Day 28.	Failure	Success	Failure	Success	From D26 to D30
8	Recrudescence before or on Day 28 (thus on or after Day 7 to 28)	Failure	Failure	Failure	Failure	From D6 to D30

9	Re-emergence before or on Day 28 but PCR ‘uninterpretable’ or missing.	Failure	Excluded	Failure	Failure	From D6 to D30
10	Late clinical failure from Day 4 to Day 6.	Failure	Failure	Failure	Failure	Before D6 except if we have an unforeseen visit between D4 and D6
11	Early treatment failure (Day 1 to Day 3)	Failure	Failure	Failure	Failure	Before D6 except if we have an unforeseen visit between D1 and D3
12	Other *Plasmodium* species before Day 28 (in the absence of *P. falciparum*).	Excluded	Excluded	Failure	Failure	From D6 to D25
13	Other *Plasmodium* species on Day 28 (in the absence of *P. falciparum*).	Success	Success	Success	Success	D26 to D30
14	Other *Plasmodium* species before or on Day 28 (in the presence of *P. falciparum*), PCR missing, ‘negative’ or ‘uninterpretable’.	Failure	Excluded	Failure	Failure	From D6 to D30
15	Prematurely discontinued from the study before Day 28 and SoC.	Failure	Excluded	Failure	Failure	< D28<br><br>FromD6 to D25
16	Prematurely discontinued from the study before Day 28 and no record of SoC.	Excluded	Excluded	Failure	Failure	< D28<br><br>FromD6 to D25
17	Completed up to Day 28. Have records in MB (Microbiology Specimen dataset), Infection present=’N’, but PCR wasn’t done and has ‘SAMPLE TAKEN BUT NOT SENT FOR PCR ANALYSIS ‘.	Success	Success	Success	Success	From D26 to D30

*) ACPR28: Adequate clinical and parasitological response on Day 28

New version (changes are underlined):

The table below displays different potential scenarios of malaria parasites related to PCR genotyping and the corresponding assessments/ rules regarding treatment outcome crude (unadjusted) cure rate and treatment outcome PCR-adjusted cure rate for the PP and the mITT analysis set. These assessments will be taken into account for the respective efficacy analyses for this study.

	Potential scenario	PP		mITT		Time period
		Treatment Outcome Crude (unadjusted) Cure Rate	Treatment Outcome PCR-adjusted Cure Rate	Treatment Outcome Crude (unadjusted) Cure Rate	Treatment Outcome PCR-adjusted Cure Rate	Number means the day included. For ex: From D26 means Day 26 included
1	Completed up to Day 28 without re-emergence (re infection/ recrudescence) of parasites after initial clearance, thus cure rate.	Success	Success	Success	Success	From D26 to D30
2	Missing assessment on Day 28, but parasite free (No *P. falciparum* asexual parasites) after Day 28	Success	Success	Success	Success	After D30
3	Missing assessment on Day 28, no more assessments thereafter.	Excluded	Excluded	failure	failure	Last assessment before D26
4	Missing assessment on Day 28, recrudescence (confirmed by PCR) at first assessment after Day 28	Failure	Failure	Failure	Failure	Recrudescence after D30
5	Missing assessment on Day 28, re-infection (confirmed by PCR) at first assessment after Day 28.	Failure	Success	Failure	Success	Re-infection after D30
6	Re infection before Day 28 (thus on or after Day 7 to before 28)	Failure	Excluded	Failure	Failure	From D6 to D25
7	Re-infection on Day 28.	Failure	Success	Failure	Success	From D26 to D30
8	Recrudescence before or on Day 28 (thus on or after Day 7 to 28)	Failure	Failure	Failure	Failure	From D6 to D30
9	Re-emergence before or on Day 28 but PCR 'uninterpretable' or missing.	Failure	Excluded	Failure	Failure	From D6 to D30
10	Late clinical failure from Day 4 to Day 6.	Failure	Failure	Failure	Failure	Before D6

						except if we have an unforeseen visit between D4 and D6
11	Early treatment failure (Day 1 to Day 3)	Failure	Failure	Failure	Failure	Before D6 except if we have an unforeseen visit between D1 and D3
12	Other *Plasmodium* species before Day 28 (in the absence of *P. falciparum*).	Excluded	Excluded	Failure	Failure	From D6 to D25
13	Other *Plasmodium* species on Day 28 (in the absence of *P. falciparum*).	Success	Success	Success	Success	D26 to D30
14	Other *Plasmodium* species before or on Day 28 (in the presence of *P. falciparum*), PCR missing, 'negative' or 'uninterpretable'.	Failure	Excluded	Failure	Failure	From D6 to D30
15	Prematurely discontinued from the study before Day 28 and SoC.	Failure	Excluded	Failure	Failure	< D28<br><br>FromD6 to D25
16	Prematurely discontinued from the study before Day 28 and no record of SoC.	Excluded	Excluded	Failure	Failure	< D28<br><br>FromD6 to D25
17	Completed up to Day 28. Have records in MB (Microbiology Specimen dataset), Infection present='N', but PCR wasn't done and has 'SAMPLE TAKEN BUT NOT SENT FOR PCR ANALYSIS '.	Success	Success	Success	Success	From D26 to D30

These corrections do not change anything regarding the efficacy results.
