# Supplementary material for: Pyronaridine–artesunate real-world safety, tolerability, and effectiveness in malaria patients in 5 African countries: A single-arm, open-label, cohort event monitoring study
Source: PLoS Med. 2021 Jun 15;18(6):e1003669. doi: 10.1371/journal.pmed.1003669 (PMC8205155; doi:10.1371/journal.pmed.1003669)
Supplement: S1 Methods — (PDF) [file pmed.1003669.s003.pdf]

S1 Methods Rapid diagnostic tests.

Rapid diagnostic tests (RDT) used in the countries involved in the CANTAM network.

Site	RDT used	Antigen
Cameroon	SD Bioline (Pf and PAN)	HRP-2 and pLDH
DRC	SD Bioline (Pf and PAN)	HRP-2 and pLDH
Gabon	SD Bioline (Pf and PAN)	HRP-2 and pLDH
Gabon	Paracheck (Pf)	HRP-2
Gabon	Vikia (Pf and PAN)	HRP-2 and Aldolase
Ivory Coast	CareStart (Pf)	HRP-2
Congo	SD Bioline (Pf and PAN)	HRP-2 and pLDH
