# Supplementary material for: Pyronaridine–artesunate real-world safety, tolerability, and effectiveness in malaria patients in 5 African countries: A single-arm, open-label, cohort event monitoring study
Source: PLoS Med. 2021 Jun 15;18(6):e1003669. doi: 10.1371/journal.pmed.1003669 (PMC8205155; doi:10.1371/journal.pmed.1003669)
Supplement: S1 Table — (PDF) [file pmed.1003669.s004.pdf]

S1 Table Adverse events of any cause.

**Primary system organ class**<br>**Preferred term**	**Normal baseline ALT/AST (N=6961)**	**Abnormal baseline ALT/AST (N=158)**	**Unknown baseline ALT/AST (N=35)**	**Total (N=7154)**
Any adverse event	1449 (20.8)	27 (17.1)	14 (40.0)	1490 (20.8)
Blood and lymphatic system disorders	31 (0.4)	1 (0.6)	2 (5.7)	34 (0.5)
Anemia	26 (0.4)	1 (0.6)	2 (5.7)	29 (0.4)
Splenomegaly	2 (<0.1)	0	0	2 (<0.1)
Leukopenia	1 (<0.1)	0	0	1 (<0.1)
Lymphadenitis	1 (<0.1)	0	0	1 (<0.1)
Sickle cell anemia with crisis	1 (<0.1)	0	0	1 (<0.1)
White blood cell disorder	1 (<0.1)	0	0	1 (<0.1)
Cardiac disorders	6 (0.1)	0	0	6 (0.1)
Palpitations	6 (0.1)	0	0	6 (0.1)
Ear and labyrinth disorders	23 (0.3)	1 (0.6)	0	24 (0.3)
Vertigo	17 (0.2)	0	0	17 (0.2)
Ear pain	1 (<0.1)	1 (0.6)	0	2 (<0.1)
Tinnitus	2 (<0.1)	0	0	2 (<0.1)
Ear congestion	1 (<0.1)	0	0	1 (<0.1)
Ear swelling	1 (<0.1)	0	0	1 (<0.1)
Misophonia	1 (<0.1)	0	0	1 (<0.1)
Eye disorders	4 (0.1)	0	0	4 (0.1)
Conjunctival pallor	1 (<0.1)	0	0	1 (<0.1)
Eye pain	1 (<0.1)	0	0	1 (<0.1)
Eye pruritus	1 (<0.1)	0	0	1 (<0.1)
Eyelid edema	1 (<0.1)	0	0	1 (<0.1)
Vision blurred	1 (<0.1)	0	0	1 (<0.1)
Gastrointestinal disorders	494 (7.1)	11 (7.0)	10 (28.6)	515 (7.2)
Vomiting	285 (4.1)	8 (5.1)	10 (28.6)	303 (4.2)
Diarrhea	79 (1.1)	2 (1.3)	0	81 (1.1)
Abdominal pain	74 (1.1)	0	0	74 (1.0)
Nausea	31 (0.4)	0	0	31 (0.4)
Abdominal pain upper	14 (0.2)	0	0	14 (0.2)
Constipation	6 (0.1)	0	0	6 (0.1)
Gastritis	5 (0.1)	0	0	5 (0.1)
Toothache	5 (0.1)	0	0	5 (0.1)
Epigastric discomfort	3 (<0.1)	0	0	3 (<0.1)
Stomatitis	3 (<0.1)	0	0	3 (<0.1)
Enteritis	2 (<0.1)	0	0	2 (<0.1)
Feces discolored	2 (<0.1)	0	0	2 (<0.1)
Hematochezia	2 (<0.1)	0	0	2 (<0.1)
Oral disorder	2 (<0.1)	0	0	2 (<0.1)
Abdominal pain lower	1 (<0.1)	0	0	1 (<0.1)
Dry mouth	1 (<0.1)	0	0	1 (<0.1)
Dyspepsia	0	1 (0.6)	0	1 (<0.1)
Dysphagia	1 (<0.1)	0	0	1 (<0.1)
Gastric disorder	1 (<0.1)	0	0	1 (<0.1)
Hematemesis	1 (<0.1)	0	0	1 (<0.1)
Lip swelling	1 (<0.1)	0	0	1 (<0.1)
Lip ulceration	1 (<0.1)	0	0	1 (<0.1)

**Primary system organ class**<br>**Preferred term**	**Normal baseline ALT/AST (N=6961)**	**Abnormal baseline ALT/AST (N=158)**	**Unknown baseline ALT/AST (N=35)**	**Total (N=7154)**
Mouth ulceration	1 (<0.1)	0	0	1 (<0.1)
Oral mucosal eruption	1 (<0.1)	0	0	1 (<0.1)
Oral pain	1 (<0.1)	0	0	1 (<0.1)
Tongue ulceration	1 (<0.1)	0	0	1 (<0.1)
Tooth disorder	1 (<0.1)	0	0	1 (<0.1)
General disorders and administration site conditions	519 (7.5)	6 (3.8)	1 (2.9)	526 (7.4)
Pyrexia	361 (5.2)	4 (2.5)	1 (2.9)	366 (5.1)
Fatigue	65 (0.9)	0	0	65 (0.9)
Asthenia	48 (0.7)	1 (0.6)	0	49 (0.7)
Influenza like illness	40 (0.6)	1 (0.6)	0	41 (0.6)
Chest pain	4 (0.1)	0	0	4 (0.1)
Chills	4 (0.1)	0	0	4 (0.1)
Peripheral swelling	4 (0.1)	0	0	4 (0.1)
Pain	3 (<0.1)	0	0	3 (<0.1)
Drowning	1 (<0.1)	0	0	1 (<0.1)
Hyperthermia	1 (<0.1)	0	0	1 (<0.1)
Induration	1 (<0.1)	0	0	1 (<0.1)
Malaise	1 (<0.1)	0	0	1 (<0.1)
Mucosal discoloration	1 (<0.1)	0	0	1 (<0.1)
Edema peripheral	1 (<0.1)	0	0	1 (<0.1)
Suprapubic pain	1 (<0.1)	0	0	1 (<0.1)
Hepatobiliary disorders	1 (<0.1)	0	0	1 (<0.1)
Hepatomegaly	1 (<0.1)	0	0	1 (<0.1)
Immune system disorders	1 (<0.1)	0	0	1 (<0.1)
Hypersensitivity	1 (<0.1)	0	0	1 (<0.1)
Infections and infestations	196 (2.8)	8 (5.1)	2 (5.7)	206 (2.9)
Influenza	43 (0.6)	3 (1.9)	0	46 (0.6)
Malaria	16 (0.2)	1 (0.6)	1 (2.9)	18 (0.3)
Acarodermatitis	13 (0.2)	0	1 (2.9)	14 (0.2)
Nasopharyngitis	12 (0.2)	0	0	12 (0.2)
Bronchitis	11 (0.2)	0	0	11 (0.2)
Urinary tract infection	8 (0.1)	1 (0.6)	0	9 (0.1)
Tonsillitis	8 (0.1)	0	0	8 (0.1)
Pneumonia	7 (0.1)	0	0	7 (0.1)
Respiratory tract infection	7 (0.1)	0	0	7 (0.1)
Gastroenteritis	6 (0.1)	0	0	6 (0.1)
Parasitic gastroenteritis	6 (0.1)	0	0	6 (0.1)
Abscess limb	5 (0.1)	0	0	5 (0.1)
Bacterial infection	5 (0.1)	0	0	5 (0.1)
Abscess	3 (<0.1)	1 (0.6)	0	4 (0.1)
Oral herpes	4 (0.1)	0	0	4 (0.1)
Pharyngitis	4 (0.1)	0	0	4 (0.1)
Helminthic infection	3 (<0.1)	0	0	3 (<0.1)
Tinea capitis	3 (<0.1)	0	0	3 (<0.1)
Dysentery	1 (<0.1)	1 (0.6)	0	2 (<0.1)
Ear infection	2 (<0.1)	0	0	2 (<0.1)

Primary system organ class<br>Preferred term	Normal baseline ALT/AST (N=6961)	Abnormal baseline ALT/AST (N=158)	Unknown baseline ALT/AST (N=35)	Total (N=7154)
Impetigo	2 (<0.1)	0	0	2 (<0.1)
Infection	2 (<0.1)	0	0	2 (<0.1)
Mumps	2 (<0.1)	0	0	2 (<0.1)
Oral candidiasis	2 (<0.1)	0	0	2 (<0.1)
Rhinitis	2 (<0.1)	0	0	2 (<0.1)
Sepsis	2 (<0.1)	0	0	2 (<0.1)
Skin infection	2 (<0.1)	0	0	2 (<0.1)
Staphylococcal skin infection	2 (<0.1)	0	0	2 (<0.1)
Subcutaneous abscess	2 (<0.1)	0	0	2 (<0.1)
Tinea infection	2 (<0.1)	0	0	2 (<0.1)
Typhoid fever	2 (<0.1)	0	0	2 (<0.1)
Varicella	2 (<0.1)	0	0	2 (<0.1)
Abscess oral	1 (<0.1)	0	0	1 (<0.1)
Appendicitis perforated	1 (<0.1)	0	0	1 (<0.1)
Bacterial parotitis	0	1 (0.6)	0	1 (<0.1)
Bacterial vaginosis	1 (<0.1)	0	0	1 (<0.1)
Chest wall abscess	1 (<0.1)	0	0	1 (<0.1)
Dermatophytosis	1 (<0.1)	0	0	1 (<0.1)
Erysipelas	1 (<0.1)	0	0	1 (<0.1)
Fungal infection	1 (<0.1)	0	0	1 (<0.1)
Furuncle	1 (<0.1)	0	0	1 (<0.1)
Genital infection	1 (<0.1)	0	0	1 (<0.1)
Genitourinary tract infection	1 (<0.1)	0	0	1 (<0.1)
Infection parasitic	1 (<0.1)	0	0	1 (<0.1)
Measles	1 (<0.1)	0	0	1 (<0.1)
Otitis externa	1 (<0.1)	0	0	1 (<0.1)
Peritonitis	1 (<0.1)	0	0	1 (<0.1)
Pulmonary tuberculosis	1 (<0.1)	0	0	1 (<0.1)
Pulpitis dental	1 (<0.1)	0	0	1 (<0.1)
Salmonellosis	1 (<0.1)	0	0	1 (<0.1)
Schistosomiasis	1 (<0.1)	0	0	1 (<0.1)
Injury, poisoning and procedural complications	8 (0.1)	2 (1.3)	0	10 (0.1)
Injury	2 (<0.1)	1 (0.6)	0	3 (<0.1)
Post-traumatic pain	2 (<0.1)	0	0	2 (<0.1)
Forearm fracture	1 (<0.1)	0	0	1 (<0.1)
Joint injury	0	1 (0.6)	0	1 (<0.1)
Limb injury	1 (<0.1)	0	0	1 (<0.1)
Lip injury	1 (<0.1)	0	0	1 (<0.1)
Mouth injury	1 (<0.1)	0	0	1 (<0.1)
Investigations	2 (<0.1)	0	0	2 (<0.1)
Hemoglobin decreased	1 (<0.1)	0	0	1 (<0.1)
Weight decreased	1 (<0.1)	0	0	1 (<0.1)
Metabolism and nutrition disorders	48 (0.7)	0	0	48 (0.7)
Decreased appetite	42 (0.6)	0	0	42 (0.6)
Increased appetite	3 (<0.1)	0	0	3 (<0.1)
Dehydration	2 (<0.1)	0	0	2 (<0.1)
Hyperphagia	1 (<0.1)	0	0	1 (<0.1)

**Primary system organ class**<br>**Preferred term**	**Normal baseline ALT/AST (N=6961)**	**Abnormal baseline ALT/AST (N=158)**	**Unknown baseline ALT/AST (N=35)**	**Total (N=7154)**
Musculoskeletal and connective tissue disorders	48 (0.7)	0	0	48 (0.7)
Arthralgia	16 (0.2)	0	0	16 (0.2)
Myalgia	12 (0.2)	0	0	12 (0.2)
Back pain	7 (0.1)	0	0	7 (0.1)
Neck pain	5 (0.1)	0	0	5 (0.1)
Joint swelling	3 (<0.1)	0	0	3 (<0.1)
Pain in extremity	3 (<0.1)	0	0	3 (<0.1)
Musculoskeletal pain	2 (<0.1)	0	0	2 (<0.1)
Muscle spasms	1 (<0.1)	0	0	1 (<0.1)
Musculoskeletal chest pain	1 (<0.1)	0	0	1 (<0.1)
Nervous system disorders	264 (3.8)	0	1 (2.9)	265 (3.7)
Headache	201 (2.9)	0	1 (2.9)	202 (2.8)
Dizziness	65 (0.9)	0	0	65 (0.9)
Seizure	3 (<0.1)	0	0	3 (<0.1)
Ageusia	1 (<0.1)	0	0	1 (<0.1)
Burning sensation	1 (<0.1)	0	0	1 (<0.1)
Dysgeusia	1 (<0.1)	0	0	1 (<0.1)
Febrile convulsion	1 (<0.1)	0	0	1 (<0.1)
Hypersomnia	1 (<0.1)	0	0	1 (<0.1)
Hypoesthesia	1 (<0.1)	0	0	1 (<0.1)
Migraine	1 (<0.1)	0	0	1 (<0.1)
Neuralgia	1 (<0.1)	0	0	1 (<0.1)
Somnolence	1 (<0.1)	0	0	1 (<0.1)
Pregnancy, puerperium and perinatal conditions	1 (<0.1)	0	0	1 (<0.1)
Uterine hypertonus	1 (<0.1)	0	0	1 (<0.1)
Psychiatric disorders	1 (<0.1)	0	0	1 (<0.1)
Insomnia	1 (<0.1)	0	0	1 (<0.1)
Renal and urinary disorders	6 (0.1)	0	0	6 (0.1)
Chromaturia	3 (<0.1)	0	0	3 (<0.1)
Hematuria	2 (<0.1)	0	0	2 (<0.1)
Renal pain	1 (<0.1)	0	0	1 (<0.1)
Reproductive system and breast disorders	4 (0.1)	0	0	4 (0.1)
Dysmenorrhea	1 (<0.1)	0	0	1 (<0.1)
Genital hemorrhage	1 (<0.1)	0	0	1 (<0.1)
Testicular pain	1 (<0.1)	0	0	1 (<0.1)
Vulval disorder	1 (<0.1)	0	0	1 (<0.1)
Respiratory, thoracic and mediastinal disorders	128 (1.8)	3 (1.9)	3 (1.9)	131 (1.8)
Cough	100 (1.4)	3 (1.9)	3 (1.9)	103 (1.4)
Rhinorrhea	23 (0.3)	0	0	23 (0.3)
Oropharyngeal pain	6 (0.1)	0	0	6 (0.1)
Dyspnea	3 (<0.1)	0	0	3 (<0.1)
Epistaxis	2 (<0.1)	0	0	2 (<0.1)
Asphyxia	1 (<0.1)	0	0	1 (<0.1)
Pneumonitis	1 (<0.1)	0	0	1 (<0.1)
Productive cough	1 (<0.1)	0	0	1 (<0.1)

Primary system organ class<br>Preferred term	Normal baseline ALT/AST (N=6961)	Abnormal baseline ALT/AST (N=158)	Unknown baseline ALT/AST (N=35)	Total (N=7154)
Skin and subcutaneous tissue disorders	100 (1.4)	1 (0.6)	1 (0.6)	101 (1.4)
Pruritus	40 (0.6)	0	0	40 (0.6)
Rash	22 (0.3)	0	0	22 (0.3)
Hyperhidrosis	12 (0.2)	0	0	12 (0.2)
Dermatitis	7 (0.1)	1 (0.6)	0	8 (0.1)
Rash pruritic	5 (0.1)	0	0	5 (0.1)
Urticaria	5 (0.1)	0	0	5 (0.1)
Miliaria	3 (<0.1)	0	0	3 (<0.1)
Swelling face	3 (<0.1)	0	0	3 (<0.1)
Dermatosis	2 (<0.1)	0	0	2 (<0.1)
Dermatitis allergic	1 (<0.1)	0	0	1 (<0.1)
Erythema	1 (<0.1)	0	0	1 (<0.1)
Pruritus generalized	1 (<0.1)	0	0	1 (<0.1)
Rash maculo-papular	1 (<0.1)	0	0	1 (<0.1)
Rash papular	1 (<0.1)	0	0	1 (<0.1)
Skin swelling	1 (<0.1)	0	0	1 (<0.1)
Stevens-Johnson syndrome	1 (<0.1)	0	0	1 (<0.1)
Vascular disorders	3 (<0.1)	0	0	3 (<0.1)
Hypertension	2 (<0.1)	0	0	2 (<0.1)
Hemodynamic instability	1 (<0.1)	0	0	1 (<0.1)

Patients may have had more than one adverse event. Normal liver function tests were alanine aminotransferase (ALT) or aspartate aminotransferase (AST) ≤2x the upper limit of normal (ULN) and abnormal values were AST or ALT >2xULN at baseline. Adverse events were coded using MedDRA (version 22).
