# Supplementary material for: Pyronaridine–artesunate real-world safety, tolerability, and effectiveness in malaria patients in 5 African countries: A single-arm, open-label, cohort event monitoring study
Source: PLoS Med. 2021 Jun 15;18(6):e1003669. doi: 10.1371/journal.pmed.1003669 (PMC8205155; doi:10.1371/journal.pmed.1003669)
Supplement: S2 Table — (PDF) [file pmed.1003669.s005.pdf]

S2 Table Adverse events of any cause following repeated pyronaridine-artesunate treatment.

**Primary system organ class**<br>**Preferred term**	**Normal baseline ALT/AST (N=1023)**	**Abnormal baseline ALT/AST (N=16)**	**Unknown baseline ALT/AST (N=12)**	**Total (N=1051)**
Any adverse event	167 (16.3)	3 (18.8)	4 (33.3)	174 (16.6)
Blood and lymphatic system disorders	3 (0.3)	0	0	3 (0.3)
Anemia	3 (0.3)	0	0	3 (0.3)
Ear and labyrinth disorders	1 (0.1)	0	0	1 (0.1)
Ear pain	1 (0.1)	0	0	1 (0.1)
Eye disorders	1 (0.1)	0	0	1 (0.1)
Eyelid edema	1 (0.1)	0	0	1 (0.1)
Gastrointestinal disorders	49 (4.8)	0	2 (16.7)	51 (4.9)
Vomiting	35 (3.4)	0	2 (16.7)	37 (3.5)
Diarrhea	5 (0.5)	0	0	5 (0.5)
Abdominal pain	6 (0.6)	0	0	6 (0.6)
Nausea	2 (0.2)	0	0	2 (0.2)
Gastritis	1 (0.1)	0	0	1 (0.1)
Toothache	1 (0.1)	0	0	1 (0.1)
Stomatitis	1 (0.1)	0	0	1 (0.1)
General disorders and administration site conditions	61 (6.0)	3 (18.8)	0	64 (6.1)
Pyrexia	46 (4.5)	1 (6.3)	0	47 (4.5)
Fatigue	6 (0.6)	0	0	6 (0.6)
Asthenia	2 (0.2)	1 (6.3)	0	3 (0.3)
Influenza like illness	5 (0.5)	1 (6.3)	0	6 (0.6)
Peripheral swelling	2 (0.2)	0	0	2 (0.2)
Infections and infestations				
Influenza	2 (0.2)	0	0	2 (0.2)
Malaria	1 (0.1)	1 (6.3)	0	2 (0.2)
Acarodermatitis	3 (0.3)	0	0	3 (0.3)
Nasopharyngitis	2 (0.2)	0	0	2 (0.2)
Bronchitis	1 (0.1)	0	0	1 (0.1)
Urinary tract infection	1 (0.1)	0	0	1 (0.1)
Tonsillitis	3 (0.3)	0	0	3 (0.3)
Pneumonia	3 (0.3)	0	0	3 (0.3)
Gastroenteritis	3 (0.3)	0	0	3 (0.3)
Parasitic gastroenteritis	1 (0.1)	0	0	1 (0.1)
Abscess limb	1 (0.1)	0	0	1 (0.1)
Oral herpes	1 (0.1)	0	0	1 (0.1)
Tinea capitis	1 (0.1)	0	0	1 (0.1)
Mumps	1 (0.1)	0	0	1 (0.1)
Oral candidiasis	1 (0.1)	0	0	1 (0.1)
Subcutaneous abscess	1 (0.1)	0	0	1 (0.1)
Injury, poisoning and procedural complicati ons	1 (0.1)	0	0	1 (0.1)
Injury	1 (0.1)	0	0	1 (0.1)
Metabolism and nutrition disorders	6 (0.6)	0	0	6 (0.6)
Decreased appetite	6 (0.6)	0	0	6 (0.6)
Musculoskeletal and connective tissue disorders	3 (0.3)	0	0	3 (0.3)
Arthralgia	2 (0.2)	0	0	2 (0.2)

Primary system organ class<br>Preferred term	Normal baseline ALT/AST (N=1023)	Abnormal baseline ALT/AST (N=16)	Unknown baseline ALT/AST (N=12)	Total (N=1051)
Joint swelling	1 (0.1)	0	0	1 (0.1)
Nervous system disorders	29 (2.8)	0	1 (8.3)	30 (2.9)
Headache	24 (2.3)	0	1 (8.3)	25 (2.4)
Dizziness	4 (0.4)	0	0	4 (0.4)
Burning sensation	1 (0.1)	0	0	1 (0.1)
Respiratory, thoracic and mediastinal disorders	25 (2.4)	0	0	25 (2.4)
Cough	18 (1.8)	0	0	18 (1.7)
Rhinorrhea	10 (1.0)	0	0	10 (1.0)
Oropharyngeal pain	1 (0.1)	0	0	1 (0.1)
Skin and subcutaneous tissue disorders	12 (1.2)	0	0	12 (1.1)
Pruritus	3 (0.3)	0	0	3 (0.3)
Rash	4 (0.4)	0	0	4 (0.4)
Dermatitis	2 (0.2)	0	0	2 (0.2)
Rash pruritic	1 (0.1)	0	0	1 (0.1)
Urticaria	2 (0.2)	0	0	2 (0.2)
Rash papular	1 (0.1)	0	0	1 (0.1)
Swelling face	1 (0.1)	0	0	1 (0.1)

Adverse events were coded using MedDRA (version 22).
