# Supplementary material for: Pyronaridine–artesunate real-world safety, tolerability, and effectiveness in malaria patients in 5 African countries: A single-arm, open-label, cohort event monitoring study
Source: PLoS Med. 2021 Jun 15;18(6):e1003669. doi: 10.1371/journal.pmed.1003669 (PMC8205155; doi:10.1371/journal.pmed.1003669)
Supplement: S3 Table — (PDF) [file pmed.1003669.s006.pdf]

S3 Table Adverse events of any cause by age.

**Primary system organ class**<br>**Preferred term**	**<1 year (N=134)**	**≥1 to <5 years (N=1711)**	**>5 to <18 years (N=3609)**	**≥18 years (N=1700)**
Any adverse event	39 (29.1)	384 (22.4)	611 (16.9)	459 (27.0)
Blood and lymphatic system disorders	1 (0.7)	20 (1.2)	10 (0.3)	3 (0.2)
Anemia	1 (0.7)	18 (1.1)	7 (0.2)	3 (0.2)
Splenomegaly	0	1 (0.1)	1 (<0.1)	0
Leukopenia	0	1 (0.1)	0	0
Lymphadenitis	0	0	1 (<0.1)	0
Sickle cell anemia with crisis	0	0	1 (<0.1)	0
White blood cell disorder	0	1 (0.1)	0	0
Cardiac disorders	0	0	0	6 (0.4)
Palpitations	0	0	0	6 (0.4)
Ear and labyrinth disorders	0	3 (0.2)	6 (0.2)	15 (0.9)
Vertigo	0	0	5 (0.1)	12 (0.7)
Ear pain	0	1 (0.1)	1 (<0.1)	0
Tinnitus	0	0	0	2 (0.1)
Ear congestion	0	1 (0.1)	0	0
Ear swelling	0	1 (0.1)	0	0
Misophonia	0	0	0	1 (0.1)
Eye disorders	0	1 (0.1)	2 (0.1)	1 (0.1)
Conjunctival pallor	0	0	1 (<0.1)	0
Eye pain	0	0	1 (<0.1)	0
Eye pruritus	0	0	0	1 (0.1)
Eyelid edema	0	1 (0.1)	0	0
Vision blurred	0	0	1 (<0.1)	0
Gastrointestinal disorders	18 (13.4)	170 (9.9)	191 (5.3)	138 (8.1)
Vomiting	15 (11.2)	131 (7.7)	112 (3.1)	47 (2.8)
Diarrhea	3 (2.2)	33 (1.9)	22 (0.6)	23 (1.4)
Abdominal pain	1 (0.7)	6 (0.4)	40 (1.1)	27 (1.6)
Nausea	0	2 (0.1)	8 (0.2)	21 (1.2)
Abdominal pain upper	0	0	4 (0.1)	10 (0.6)
Constipation	0	1 (0.1)	2 (0.1)	3 (0.2)
Gastritis	0	0	1 (<0.1)	4 (0.2)
Toothache	0	0	3 (0.1)	2 (0.1)
Epigastric discomfort	0	0	1 (<0.1)	2 (0.1)
Stomatitis	0	0	2 (0.1)	1 (0.1)
Enteritis	0	0	0	2 (0.1)
Feces discolored	0	1 (0.1)	0	1 (0.1)
Hematochezia	0	1 (0.1)	1 (<0.1)	0
Oral disorder	0	1 (0.1)	0	1 (0.1)
Abdominal pain lower	0	0	0	1 (0.1)
Dry mouth	0	0	0	1 (0.1)
Dyspepsia	0	0	0	1 (0.1)
Dysphagia	0	0	1 (<0.1)	0
Gastric disorder	0	0	0	1 (0.1)
Hematemesis	0	0	1 (<0.1)	0
Lip swelling	0	0	0	1 (0.1)
Lip ulceration	0	0	0	1 (0.1)
Mouth ulceration	0	0	1 (<0.1)	0

Primary system organ class<br>Preferred term	<1 year (N=134)	≥1 to <5 years (N=1711)	>5 to <18 years (N=3609)	≥18 years (N=1700)
Oral mucosal eruption	0	0	1 (<0.1)	0
Oral pain	0	0	1 (<0.1)	0
Tongue ulceration	0	0	1 (<0.1)	0
Tooth disorder	0	1 (0.1)	0	0
General disorders and administration site conditions	13 (9.7)	141 (8.2)	225 (6.2)	148 (8.7)
Pyrexia	10 (7.5)	115 (6.7)	176 (4.9)	66 (3.9)
Fatigue	2 (1.5)	2 (0.1)	18 (0.5)	43 (2.5)
Asthenia	0	7 (0.4)	16 (0.4)	26 (1.5)
Influenza like illness	1 (0.7)	16 (0.9)	10 (0.3)	14 (0.8)
Chest pain	0	0	3 (0.1)	1 (0.1)
Chills	0	0	1 (<0.1)	3 (0.2)
Peripheral swelling	0	0	0	2 (0.1)
Pain	0	0	2 (0.1)	3 (0.2)
Drowning	0	0	1 (<0.1)	0
Hyperthermia	0	1 (0.1)	0	0
Induration	0	0	1 (<0.1)	0
Malaise	0	0	1 (<0.1)	0
Mucosal discoloration	0	0	1 (<0.1)	0
Edema peripheral	0	0	0	1 (0.1)
Suprapubic pain	0	0	0	1 (0.1)
Hepatobiliary disorders	0	0	1 (<0.1)	0
Hepatomegaly	0	0	1 (<0.1)	0
Immune system disorders	0	0	1 (<0.1)	0
Hypersensitivity	0	0	1 (<0.1)	0
Infections and infestations	7 (5.2)	68 (4.0)	85 (2.4)	46 (2.7)
Influenza	0	17 (1.0)	21 (0.6)	8 (0.5)
Malaria	0	8 (0.5)	6 (0.2)	4 (0.2)
Acarodermatitis	1 (0.7)	2 (0.1)	8 (0.2)	3 (0.2)
Nasopharyngitis	1 (0.7)	6 (0.4)	3 (0.1)	2 (0.1)
Bronchitis	1 (0.7)	9 (0.5)	1 (<0.1)	
Urinary tract infection	0	2 (0.1)	3 (0.1)	4 (0.2)
Tonsillitis	0	4 (0.2)	4 (0.1)	
Pneumonia	0	2 (0.1)	2 (0.1)	3 (0.2)
Respiratory tract infection	1 (0.7)	2 (0.1)	3 (0.1)	
Gastroenteritis	0	2 (0.1)	2 (0.1)	2 (0.1)
Parasitic gastroenteritis	0	2 (0.1)	4 (0.1)	
Abscess limb	0	1 (0.1)	2 (0.1)	2 (0.1)
Bacterial infection	0	0	3 (0.1)	2 (0.1)
Abscess	1 (0.7)	1 (0.1)	1 (<0.1)	1 (0.1)
Oral herpes	0	1 (0.1)	1 (<0.1)	2 (0.1)
Pharyngitis	0	1 (0.1)	3 (0.1)	0
Helminthic infection	0	0	1 (<0.1)	2 (0.1)
Tinea capitis	0	0	3 (0.1)	0
Dysentery	0	2 (0.1)	0	0
Ear infection	0	1 (0.1)	1 (<0.1)	0
Impetigo	1 (0.7)	1 (0.1)	0	0
Infection	0	0	1 (<0.1)	1 (0.1)

Primary system organ class<br>Preferred term	<1 year (N=134)	≥1 to <5 years (N=1711)	>5 to <18 years (N=3609)	≥18 years (N=1700)
Mumps	0	0	2 (0.1)	0
Oral candidiasis	0	2 (0.1)		0
Rhinitis	0	1 (0.1)	1 (<0.1)	0
Sepsis	0	1 (0.1)	1 (<0.1)	0
Skin infection	0	0	1 (<0.1)	1 (0.1)
Staphylococcal skin infection	0	1 (0.1)	1 (<0.1)	0
Subcutaneous abscess	0	1 (0.1)	0	1 (0.1)
Tinea infection	0	0	2 (0.1)	0
Typhoid fever	0	1 (0.1)	0	1 (0.1)
Varicella	0	1 (0.1)	1 (<0.1)	0
Abscess oral	0	1 (0.1)	0	0
Appendicitis perforated	0	0	0	1 (0.1)
Bacterial parotitis	0	1 (0.1)	0	0
Bacterial vaginosis	0	0	0	1 (0.1)
Chest wall abscess	0	0	1 (<0.1)	0
Dermatophytosis	0	0	1 (<0.1)	0
Erysipelas	0	0	1 (<0.1)	0
Fungal infection	0	1 (0.1)	0	0
Furuncle	0	0	0	1 (0.1)
Genital infection	0	0	0	1 (0.1)
Genitourinary tract infection	0	0	0	1 (0.1)
Infection parasitic	0	0	1 (<0.1)	0
Measles	1 (0.7)	0	0	0
Otitis externa	0	0	0	1 (0.1)
Peritonitis	0	0	0	1 (0.1)
Pulmonary tuberculosis	0	0	0	1 (0.1)
Pulpitis dental	0	0	1 (<0.1)	0
Salmonellosis	0	0	0	1 (0.1)
Schistosomiasis	0	0	1 (<0.1)	0
Injury, poisoning and procedural complications	0	0	3 (0.1)	7 (0.4)
Injury	0	0	0	3 (0.2)
Post-traumatic pain	0	0	1 (<0.1)	1 (0.1)
Forearm fracture	0	0	1 (<0.1)	0
Joint injury	0	0	1 (<0.1)	0
Limb injury	0	0	0	1 (0.1)
Lip injury	0	0	0	1 (0.1)
Mouth injury	0	0	0	1 (0.1)
Investigations	0	1 (0.1)	1 (<0.1)	0
Hemoglobin decreased	0	1 (0.1)	0	0
Weight decreased	0	0	1 (<0.1)	0
Metabolism and nutrition disorders	3 (2.2)	15 (0.9)	10 (0.3)	20 (1.2)
Decreased appetite	3 (2.2)	13 (0.8)	7 (0.2)	19 (1.1)
Increased appetite	0	0	3 (0.1)	0
Dehydration	0	2 (0.1)	0	0
Hyperphagia	0	0	0	1 (0.1)
Musculoskeletal and connective tissue disorders	0	2 (0.1	11 (0.3)	35 (2.1)
Arthralgia	0	2 (0.1	5 (0.1)	9 (0.5)

**Primary system organ class**<br>**Preferred term**	**<1 year (N=134)**	**≥1 to <5 years (N=1711)**	**>5 to <18 years (N=3609)**	**≥18 years (N=1700)**
Myalgia	0	0	4 (0.1)	8 (0.5)
Back pain	0	0	0	7 (0.4)
Neck pain	0	0	0	5 (0.3)
Joint swelling	0	0	1 (<0.1)	2 (0.1)
Pain in extremity	0	0	0	3 (0.2)
Musculoskeletal pain	0	0	0	2 (0.1)
Muscle spasms	0	0	0	1 (0.1)
Musculoskeletal chest pain	0	0	1 (<0.1)	0
Nervous system disorders	0	14 (0.8)	113 (3.1)	138 (8.1)
Headache	0	11 (0.6)	97 (2.7)	94 (5.5)
Dizziness	0	2 (0.1)	17 (0.5)	46 (2.7)
Seizure	0	1 (0.1)	0	2 (0.1)
Ageusia	0	0	0	1 (0.1)
Burning sensation	0	0	0	1 (0.1)
Dysgeusia	0	0	0	1 (0.1)
Febrile convulsion	0	0	1 (<0.1)	0
Hypersomnia	0	0	0	1 (0.1)
Hypoesthesia	0	0	0	1 (0.1)
Migraine	0	0	0	1 (0.1)
Neuralgia	0	0	0	1 (0.1)
Somnolence	0	1 (0.1)	0	0
Pregnancy, puerperium and perinatal conditions	0	0	0	1 (0.1)
Uterine hypertonus	0	0	0	1 (0.1)
Psychiatric disorders	0	0	0	1 (0.1)
Insomnia	0	0	0	1 (0.1)
Renal and urinary disorders	0	0	2 (0.1)	4 (0.2)
Chromaturia	0	0	1 (<0.1)	2 (0.1)
Hematuria	0	0	1 (<0.1)	1 (0.1)
Renal pain	0	0	0	1 (0.1)
Reproductive system and breast disorders	0	0	2 (0.1)	2 (0.1)
Dysmenorrhea	0	0	0	1 (0.1)
Genital hemorrhage	0	0	0	1 (0.1)
Testicular pain	0	0	1 (<0.1)	0
Vulval disorder	0	0	1 (<0.1)	0
Respiratory, thoracic and mediastinal disorders	7 (5.2)	57 (3.3)	52 (1.4)	15 (0.9)
Cough	7 (5.2)	42 (2.5)	44 (1.2)	10 (0.6)
Rhinorrhea	1 (0.7)	15 (0.9)	7 (0.2)	0
Oropharyngeal pain	0	2 (0.1)	2 (0.1)	2 (0.1)
Dyspnea	0	1 (0.1)	0	2 (0.1)
Epistaxis	0	2 (0.1)	0	0
Asphyxia	0	0	0	1 (0.1)
Pneumonitis	0	0	1 (<0.1)	0
Productive cough	0	1 (0.1)	0	0
Skin and subcutaneous tissue disorders	1 (0.7)	23 (1.3)	36 (1.0)	41 (2.4)
Pruritus	0	6 (0.4)	8 (0.2)	26 (1.5)
Rash	0	5 (0.3)	9 (0.2)	8 (0.5)
Hyperhidrosis	0	5 (0.3)	1 (<0.1)	6 (0.4)

**Primary system organ class**<br>**Preferred term**	**<1 year (N=134)**	**≥1 to <5 years (N=1711)**	**>5 to <18 years (N=3609)**	**≥18 years (N=1700)**
Dermatitis	0	4 (0.2)	3 (0.1)	1 (0.1)
Rash pruritic	0	0	5 (0.1)	
Urticaria	0	0	4 (0.1)	1 (0.1)
Miliaria	1 (0.7)	1 (0.1)	1 (<0.1)	0
Swelling face	0	1 (0.1)	2 (0.1)	0
Dermatosis	0	1 (0.1)	1 (<0.1)	0
Dermatitis allergic	0	0	1 (<0.1)	0
Erythema	0	0	1 (<0.1)	0
Pruritus generalized	0	1 (0.1)	0	0
Rash maculo-papular	0	0	0	1 (0.1)
Rash papular	0	0	1 (<0.1)	0
Skin swelling	0	1 (0.1)	0	0
Stevens-Johnson syndrome	0	0	1 (<0.1)	0
Vascular disorders	0	0	0	3 (0.2)
Hypertension	0	0	0	2 (0.1)
Hemodynamic instability	0	0	0	1 (0.1)

Patients may have had more than one adverse event. Patients may have been categorized differently for successive malaria episodes. Normal liver function tests were alanine aminotransferase (ALT) or aspartate aminotransferase (AST) ≤2x the upper limit of normal (ULN) and abnormal values were AST or ALT >2xULN at baseline. Adverse events were coded using MedDRA (version 22).
