# Supplementary material for: Pyronaridine–artesunate real-world safety, tolerability, and effectiveness in malaria patients in 5 African countries: A single-arm, open-label, cohort event monitoring study
Source: PLoS Med. 2021 Jun 15;18(6):e1003669. doi: 10.1371/journal.pmed.1003669 (PMC8205155; doi:10.1371/journal.pmed.1003669)
Supplement: S4 Table — (PDF) [file pmed.1003669.s007.pdf]

S4 Table Adverse events of any cause by category.

Category	Adverse event frequency, n (%)	95%CI
All patients (n=7154)	1490 (20.8)	19.9, 21.8
Baseline ALT/AST		
Normal ALT/AST (n=6961)	1449 (20.8)	19.9, 21.8
Abnormal ALT/AST (n=158)	27 (17.1)	11.6, 23.9
ALT/AST abnormality unknown (n=35)	14 (40.0)	23.9, 57.9
Sex		
Female (n=3585)	802 (22.4)	21.0, 23.8
Male (n=3569)	688 (19.3)	18.0, 20.6
Age		
<1 year (n=134)	39 (29.1)	21.6, 37.6
1 year to <5 years (n=1711)	384 (22.4)	20.5, 24.5
5 years to <18 years (n=3609)	611 (16.9)	15.7, 18.2
≥18 years (n=1700)	459 (27.0)	24.9, 29.2
Weight		
5 to <20 kg (n=2641)	539 (20.4)	18.9, 22.0
≥20 kg (n=4573)	953 (20.8)	19.7, 22.0
Nutritional status		
Malnourished (n=383)	76 (19.8)	16.0, 24.2
Non-malnourished (n=6805)	1414 (20.8)	19.8, 21.8
HIV status		
HIV negative (n=379)	94 (24.8)	20.5, 29.5
HIV positive (n=15)	4 (26.7)	7.8, 55.1
Malaria re-treatment		
First malaria episode (n=7085)	1349 (19.0)	18.1, 19.9
Malaria retreatment (n=1051)	174 (16.6)	14.4, 18.9
