# Supplementary material for: Pyronaridine–artesunate real-world safety, tolerability, and effectiveness in malaria patients in 5 African countries: A single-arm, open-label, cohort event monitoring study
Source: PLoS Med. 2021 Jun 15;18(6):e1003669. doi: 10.1371/journal.pmed.1003669 (PMC8205155; doi:10.1371/journal.pmed.1003669)
Supplement: S5 Table — (PDF) [file pmed.1003669.s008.pdf]

S5 Table Adverse events considered related to pyronaridine-artesunate.

Primary system organ class<br>Preferred term	Normal baseline ALT/AST (N=6961)	Abnormal baseline ALT/AST (N=158)	Unknown baseline ALT/AST (N=35)	Total (N=7154)
Subjects with at least one drug related adverse event	663 (9.5)	12 (7.6)	10 (28.6)	685 (9.6)
Blood and lymphatic system disorders	6 (0.1)	1 (0.6)	0	7 (0.1)
Anemia	5 (0.1)	1 (0.6)	0	6 (0.1)
Lymphadenitis	1 (<0.1)	0	0	1 (<0.1)
Cardiac disorders	6 (0.1)	0	0	6 (0.1)
Palpitations	6 (0.1)	0	0	6 (0.1)
Ear and labyrinth disorders	16 (0.2)	0	0	16 (0.2)
Vertigo	13 (0.2)	0	0	13 (0.2)
Ear congestion	1 (<0.1)	0	0	1 (<0.1)
Misophonia	1 (<0.1)	0	0	1 (<0.1)
Tinnitus	1 (<0.1)	0	0	1 (<0.1)
Eye disorders	2 (<0.1)	0	0	2 (<0.1)
Eye pruritus	1 (<0.1)	0	0	1 (<0.1)
Eyelid edema	1 (<0.1)	0	0	1 (<0.1)
Gastrointestinal disorders	341 (4.9)	7 (4.4)	9 (25.7)	357 (5.0)
Vomiting	213 (3.1)	6 (3.8)	9 (25.7)	228 (3.2)
Abdominal pain	43 (0.6)	0	0	43 (0.6)
Diarrhea	41 (0.6)	1 (0.6)	0	42 (0.6)
Nausea	26 (0.4)	0	0	26 (0.4)
Abdominal pain upper	9 (0.1)	0	0	9 (0.1)
Gastritis	4 (0.1)	0	0	4 (0.1)
Constipation	3 (<0.1)	0	0	3 (<0.1)
Epigastric discomfort	3 (<0.1)	0	0	3 (<0.1)
Feces discolored	2 (<0.1)	0	0	2 (<0.1)
Oral disorder	2 (<0.1)	0	0	2 (<0.1)
Stomatitis	2 (<0.1)	0	0	2 (<0.1)
Dry mouth	1 (<0.1)	0	0	1 (<0.1)
Enteritis	1 (<0.1)	0	0	1 (<0.1)
Gastric disorder	1 (<0.1)	0	0	1 (<0.1)
Hematemesis	1 (<0.1)	0	0	1 (<0.1)
Hematochezia	1 (<0.1)	0	0	1 (<0.1)
Lip swelling	1 (<0.1)	0	0	1 (<0.1)
Lip ulceration	1 (<0.1)	0	0	1 (<0.1)
Oral mucosal eruption	1 (<0.1)	0	0	1 (<0.1)
Tongue ulceration	1 (<0.1)	0	0	1 (<0.1)
General disorders and administration site conditions	131 (1.9)	1 (0.6)	0	132 (1.8)
Pyrexia	43 (0.6)	0	0	43 (0.6)
Asthenia	37 (0.5)	1 (0.6)	0	38 (0.5)
Fatigue	35 (0.5)	0	0	35 (0.5)
Influenza like illness	9 (0.1)	0	0	9 (0.1)
Chest pain	2 (<0.1)	0	0	2 (<0.1)
Peripheral swelling	2 (<0.1)	0	0	2 (<0.1)
Malaise	1 (<0.1)	0	0	1 (<0.1)
Mucosal discoloration	1 (<0.1)	0	0	1 (<0.1)
Edema peripheral	1 (<0.1)	0	0	1 (<0.1)

Primary system organ class<br>Preferred term	Normal baseline ALT/AST (N=6961)	Abnormal baseline ALT/AST (N=158)	Unknown baseline ALT/AST (N=35)	Total (N=7154)
Pain	1 (<0.1)	0	0	1 (<0.1)
Immune system disorders	1 (<0.1)	0	0	1 (<0.1)
Hypersensitivity	1 (<0.1)	0	0	1 (<0.1)
Infections and infestations	26 (0.4)	2 (1.3)	0	28 (0.4)
Nasopharyngitis	4 (0.1)	0	0	4 (0.1)
Tonsillitis	4 (0.1)	0	0	4 (0.1)
Gastroenteritis	3 (<0.1)	0	0	3 (<0.1)
Influenza	3 (<0.1)	0	0	3 (<0.1)
Oral candidiasis	2 (<0.1)	0	0	2 (<0.1)
Oral herpes	2 (<0.1)	0	0	2 (<0.1)
Respiratory tract infection	2 (<0.1)	0	0	2 (<0.1)
Urinary tract infection	1 (<0.1)	1 (0.6)	0	2 (<0.1)
Abscess	0	1 (0.6)	0	1 (<0.1)
Abscess limb	1 (<0.1)	0	0	1 (<0.1)
Abscess oral	1 (<0.1)	0	0	1 (<0.1)
Acarodermatitis	1 (<0.1)	0	0	1 (<0.1)
Bronchitis	1 (<0.1)	0	0	1 (<0.1)
Parasitic gastroenteritis	1 (<0.1)	0	0	1 (<0.1)
Pharyngitis	1 (<0.1)	0	0	1 (<0.1)
Pneumonia	1 (<0.1)	0	0	1 (<0.1)
Tinea infection	1 (<0.1)	0	0	1 (<0.1)
Injury, poisoning and procedural complications	1 (<0.1)	0	0	1 (<0.1)
Mouth injury	1 (<0.1)	0	0	1 (<0.1)
Investigations	1 (<0.1)	0	0	1 (<0.1)
Hemoglobin decreased	1 (<0.1)	0	0	1 (<0.1)
Metabolism and nutrition disorders	30 (0.4)	0	0	30 (0.4)
Decreased appetite	27 (0.4)	0	0	27 (0.4)
Increased appetite	2 (<0.1)	0	0	2 (<0.1)
Dehydration	1 (<0.1)	0	0	1 (<0.1)
Musculoskeletal and connective tissue disorders	15 (0.2)	0	0	15 (0.2)
Arthralgia	3 (<0.1)	0	0	3 (<0.1)
Back pain	3 (<0.1)	0	0	3 (<0.1)
Myalgia	3 (<0.1)	0	0	3 (<0.1)
Neck pain	3 (<0.1)	0	0	3 (<0.1)
Joint swelling	1 (<0.1)	0	0	1 (<0.1)
Muscle spasms	1 (<0.1)	0	0	1 (<0.1)
Musculoskeletal pain	1 (<0.1)	0	0	1 (<0.1)
Pain in extremity	1 (<0.1)	0	0	1 (<0.1)
Nervous system disorders	123 (1.8)	0	1 (2.9)	124 (1.7)
Headache	71 (1.0)	0	1 (2.9)	72 (1.0)
Dizziness	49 (0.7)	0	0	49 (0.7)
Ageusia	1 (<0.1)	0	0	1 (<0.1)
Dysgeusia	1 (<0.1)	0	0	1 (<0.1)
Hypersomnia	1 (<0.1)	0	0	1 (<0.1)
Hypoesthesia	1 (<0.1)	0	0	1 (<0.1)
Seizure	1 (<0.1)	0	0	1 (<0.1)

Primary system organ class<br>Preferred term	Normal baseline ALT/AST (N=6961)	Abnormal baseline ALT/AST (N=158)	Unknown baseline ALT/AST (N=35)	Total<br><br>(N=7154)
Nervous system disorders	1 (<0.1)	0	0	1 (<0.1)
Somnolence	1 (<0.1)	0	0	1 (<0.1)
Psychiatric disorders	1 (<0.1)	0	0	1 (<0.1)
Insomnia	1 (<0.1)	0	0	1 (<0.1)
Renal and urinary disorders	4 (0.1)	0	0	4 (0.1)
Chromaturia	3 (<0.1)	0	0	3 (<0.1)
Hematuria	1 (<0.1)	0	0	1 (<0.1)
Reproductive system and breast disorders	2 (<0.1)	0	0	2 (<0.1)
Genital hemorrhage	1 (<0.1)	0	0	1 (<0.1)
Vulval disorder	1 (<0.1)	0	0	1 (<0.1)
Respiratory, thoracic and mediastinal disorders	17 (0.2)	0	0	17 (0.2)
Cough	9 (0.1)	0	0	9 (0.1)
Dyspnea	3 (<0.1)	0	0	3 (<0.1)
Rhinorrhea	2 (<0.1)	0	0	2 (<0.1)
Asphyxia	1 (<0.1)	0	0	1 (<0.1)
Epistaxis	1 (<0.1)	0	0	1 (<0.1)
Oropharyngeal pain	1 (<0.1)	0	0	1 (<0.1)
Skin and subcutaneous tissue disorders	65 (0.9)	1 (0.6)	0	66 (0.9)
Pruritus	30 (0.4)	0	0	30 (0.4)
Hyperhidrosis	9 (0.1)	0	0	9 (0.1)
Rash	9 (0.1)	0	0	9 (0.1)
Urticaria	5 (0.1)	0	0	5 (0.1)
Rash pruritic	4 (0.1)	0	0	4 (0.1)
Dermatitis	2 (<0.1)	1 (0.6)	0	3 (<0.1)
Swelling face	3 (<0.1)	0	0	3 (<0.1)
Dermatitis allergic	1 (<0.1)	0	0	1 (<0.1)
Dermatosis	1 (<0.1)	0	0	1 (<0.1)
Miliaria	1 (<0.1)	0	0	1 (<0.1)
Pruritus generalized	1 (<0.1)	0	0	1 (<0.1)
Rash maculo-papular	1 (<0.1)	0	0	1 (<0.1)
Rash papular	1 (<0.1)	0	0	1 (<0.1)
Skin swelling	1 (<0.1)	0	0	1 (<0.1)
Vascular disorders	1 (<0.1)	0	0	1 (<0.1)
Hypertension	1 (<0.1)	0	0	1 (<0.1)

Patients may have had more than one adverse event. Normal liver function tests were alanine aminotransferase (ALT) or aspartate aminotransferase (AST) ≤2x the upper limit of normal (ULN) and abnormal values were AST or ALT >2xULN at baseline. Adverse events were coded using MedDRA (version 22).
