# Supplementary material for: Pyronaridine–artesunate real-world safety, tolerability, and effectiveness in malaria patients in 5 African countries: A single-arm, open-label, cohort event monitoring study
Source: PLoS Med. 2021 Jun 15;18(6):e1003669. doi: 10.1371/journal.pmed.1003669 (PMC8205155; doi:10.1371/journal.pmed.1003669)
Supplement: S6 Table — (PDF) [file pmed.1003669.s009.pdf]

S6 Table Adverse events leading to early discontinuation of pyronaridine-artesunate.

**Primary system organ class**<br>**Preferred term**	**Normal baseline ALT/AST (N=6961)**	**Abnormal baseline ALT/AST (N=158)**	**Unknown baseline ALT/AST (N=35)**	**Total (N=7154)**
Subjects with drug discontinuation owing to adverse events	42 (0.6)	2 (1.3)	10 (28.6)	54 (0.8)
Blood and lymphatic system disorders	0	0	1 (2.9)	1 (<0.1)
Anemia	0	0	1 (2.9)	1 (<0.1)
Cardiac disorders	1 (<0.1)	0	0	1 (<0.1)
Palpitations	1 (<0.1)	0	0	1 (<0.1)
Ear and labyrinth disorders	3 (<0.1)	0	0	3 (<0.1)
Vertigo	3 (<0.1)	0	0	3 (<0.1)
Gastrointestinal disorders	27 (0.4)	2 (1.3)	9 (25.7)	38 (0.5)
Vomiting	26 (0.4)	2 (1.3)	9 (25.7)	37 (0.5)
Lip swelling	1 (<0.1)	0	0	1 (<0.1)
General disorders and administration site conditions	3 (<0.1)	0	1 (2.9)	4 (0.1)
Asthenia	1 (<0.1)	0	0	1 (<0.1)
Chest pain	1 (<0.1)	0	0	1 (<0.1)
Fatigue	1 (<0.1)	0	0	1 (<0.1)
Pyrexia	0	0	1 (2.9)	1 (<0.1)
Immune system disorders	1 (<0.1)	0	0	1 (<0.1)
Hypersensitivity	1 (<0.1)	0	0	1 (<0.1)
Infections and infestations	8 (0.1)	0	1 (2.9)	9 (0.1)
Malaria	7 (0.1)	0	1 (2.9)	8 (0.1)
Sepsis	1 (<0.1)	0	0	1 (<0.1)
Metabolism and nutrition disorders	1 (<0.1)	0	0	1 (<0.1)
Decreased appetite	1 (<0.1)	0	0	1 (<0.1)
Nervous system disorders	2 (<0.1)	0	0	2 (<0.1)
Dizziness	1 (<0.1)	0	0	1 (<0.1)
Headache	1 (<0.1)	0	0	1 (<0.1)
Respiratory, thoracic and mediastinal disorders	1 (<0.1)	0	0	1 (<0.1)
Epistaxis	1 (<0.1)	0	0	1 (<0.1)
Skin and subcutaneous tissue disorders	1 (<0.1)	0	0	1 (<0.1)
Rash maculo-papular	1 (<0.1)	0	0	1 (<0.1)
Vascular disorders	1 (<0.1)	0	0	1 (<0.1)
Hemodynamic instability	1 (<0.1)	0	0	1 (<0.1)

Patients may have had more than one adverse event. Normal liver function tests were alanine aminotransferase (ALT) or aspartate aminotransferase (AST) ≤2x the upper limit of normal (ULN) and abnormal values were AST or ALT >2xULN at baseline. Adverse events were coded using MedDRA (version 22).
