# Supplementary material for: Pyronaridine–artesunate real-world safety, tolerability, and effectiveness in malaria patients in 5 African countries: A single-arm, open-label, cohort event monitoring study
Source: PLoS Med. 2021 Jun 15;18(6):e1003669. doi: 10.1371/journal.pmed.1003669 (PMC8205155; doi:10.1371/journal.pmed.1003669)
Supplement: S7 Table — (PDF) [file pmed.1003669.s010.pdf]

S7 Table Severe and life-threatening adverse events of any cause.

**Primary system organ class Preferred term**	**Severity**	**Normal baseline ALT/AST (N=6961)**	**Abnormal baseline ALT/AST (N=158)**	**Unknown baseline ALT/AST (N=35)**	**Total (N=7154)**
Patients with at least one adverse event	Mild	841 (12.1)	15 (9.5)	4 (11.4)	860 (12.0)
	Moderate	568 (8.2)	11 (7.0)	9 (25.7)	588 (8.2)
	Severe	32 (0.5)	0	0	32 (0.4)
	Life-threatening	8 (0.1)	1 (0.6)	1 (2.9)	10 (0.1)
Severe or life-threatening events					
Anemia	Severe	3 (<0.1)	0	0	3 (<0.1)
	Life-threatening	1 (<0.1)	1 (0.6)	1 (2.9)	3 (<0.1)
Sickle cell anemia crisis	Severe	1 (<0.1)	0	0	1 (<0.1)
Vomiting	Severe	4 (0.1)	0	0	4 (0.1)
Diarrhea	Severe	1 (<0.1)	0	0	1 (<0.1)
	Life-threatening	1 (<0.1)	0	0	1 (<0.1)
Abdominal pain	Severe	1 (<0.1)	0	0	1 (<0.1)
Hematemesis	Severe	1 (<0.1)	0	0	1 (<0.1)
Pyrexia	Severe	4 (0.1)	0	1 (2.9)	5 (0.1)
Asthenia	Severe	4 (0.1)	0	0	4 (0.1)
Drowning	Life-threatening	1 (<0.1)	0	0	1 (<0.1)
Malaria	Severe	6 (0.1)	0	1 (2.9)	7 (0.1)
	Life-threatening	2 (<0.1)	0	0	2 (<0.1)
Pneumonia	Severe	1 (<0.1)	0	0	1 (<0.1)
Gastroenteritis	Severe	1 (<0.1)	0	0	1 (<0.1)
Infection	Severe	1 (<0.1)	0	0	1 (<0.1)
Sepsis	Severe	1 (<0.1)	0	0	1 (<0.1)
	Life-threatening	1 (<0.1)	0	0	1 (<0.1)
Typhoid fever	Severe	1 (<0.1)	0	0	1 (<0.1)
Appendicitis perforated	Life-threatening	1 (<0.1)	0	0	1 (<0.1)
Otitis externa	Severe	1 (<0.1)	0	0	1 (<0.1)
Peritonitis	Severe	1 (<0.1)	0	0	1 (<0.1)
Pulmonary tuberculosis	Severe	1 (<0.1)	0	0	1 (<0.1)
Headache	Severe	3 (<0.1)	0	0	3 (<0.1)
Dizziness	Severe	1 (<0.1)	0	0	1 (<0.1)
Seizure	Severe	2 (<0.1)	0	0	2 (<0.1)
Uterine hypertonus	Life-threatening	1 (<0.1)	0	0	1 (<0.1)
Cough	Severe	1 (<0.1)	0	0	1 (<0.1)
Hypertension	Severe	1 (<0.1)	0	0	1 (<0.1)
Hemodynamic instability	Severe	1 (<0.1)	0	0	1 (<0.1)

Patients may have had more than one adverse event. Normal liver function tests were alanine aminotransferase (ALT) or aspartate aminotransferase (AST) ≤2x the upper limit of normal (ULN) and abnormal values were AST or ALT >2xULN at baseline. Adverse events were coded using MedDRA (version 22).
