# Supplementary material for: Pyronaridine–artesunate real-world safety, tolerability, and effectiveness in malaria patients in 5 African countries: A single-arm, open-label, cohort event monitoring study
Source: PLoS Med. 2021 Jun 15;18(6):e1003669. doi: 10.1371/journal.pmed.1003669 (PMC8205155; doi:10.1371/journal.pmed.1003669)
Supplement: S8 Table — (PDF) [file pmed.1003669.s011.pdf]

S8 Table Serious adverse events of any cause.

**Primary system organ class**<br>**Preferred term**	**Normal baseline ALT/AST (N=6961)**	**Abnormal baseline ALT/AST (N=158)**	**Unknown baseline ALT/AST (N=35)**	**Total (N=7154)**
Patients with at least one serious adverse event	27 (0.4)	1 (0.6)	1 (2.9)	29 (0.4)
Blood and lymphatic system disorders	6 (0.1)	1 (0.6)	1 (2.9)	8 (0.1)
Anemia	5 (0.1)	1 (0.6)	1 (2.9)	7 (0.1)
Sickle cell anemia with crisis	1 (<0.1)	0	0	1 (<0.1)
Gastrointestinal disorders	1 (<0.1)	0	0	1 (<0.1)
Diarrhea	1 (<0.1)	0	0	1 (<0.1)
General disorders and administration site conditions	1 (<0.1)	0	0	1 (<0.1)
Drowning	1 (<0.1)	0	0	1 (<0.1)
Infections and infestations	15 (0.2)	0	1 (2.9)	16 (0.2)
Malaria	8 (0.1)	0	1 (2.9)	9 (0.1)
Sepsis	2 (<0.1)	0	0	2 (<0.1)
Appendicitis perforated	1 (<0.1)	0	0	1 (<0.1)
Gastroenteritis	1 (<0.1)	0	0	1 (<0.1)
Otitis externa	1 (<0.1)	0	0	1 (<0.1)
Pneumonia	1 (<0.1)	0	0	1 (<0.1)
Pulmonary tuberculosis	1 (<0.1)	0	0	1 (<0.1)
Nervous system disorders	1 (<0.1)	0	0	1 (<0.1)
Headache	1 (<0.1)	0	0	1 (<0.1)
Seizure	1 (<0.1)	0	0	1 (<0.1)
Pregnancy, puerperium and perinatal conditions	1 (<0.1)	0	0	1 (<0.1)
Uterine hypertonus	1 (<0.1)	0	0	1 (<0.1)
Respiratory, thoracic and mediastinal disorders	1 (<0.1)	0	0	1 (<0.1)
Epistaxis	1 (<0.1)	0	0	1 (<0.1)
Skin and subcutaneous tissue disorders	1 (<0.1)	0	0	1 (<0.1)
Stevens-Johnson syndrome	1 (<0.1)	0	0	1 (<0.1)
Vascular disorders	2 (<0.1)	0	0	2 (<0.1)
Hemodynamic instability	1 (<0.1)	0	0	1 (<0.1)
Hypertension	1 (<0.1)	0	0	1 (<0.1)

Patients may have had more than one adverse event. Normal liver function tests were alanine aminotransferase (ALT) or aspartate aminotransferase (AST) ≤2x the upper limit of normal (ULN) and abnormal values were AST or ALT >2xULN at baseline. Adverse events were coded using MedDRA (version 22).
