# Supplementary material for: Pyronaridine–artesunate real-world safety, tolerability, and effectiveness in malaria patients in 5 African countries: A single-arm, open-label, cohort event monitoring study
Source: PLoS Med. 2021 Jun 15;18(6):e1003669. doi: 10.1371/journal.pmed.1003669 (PMC8205155; doi:10.1371/journal.pmed.1003669)
Supplement: S9 Table — (PDF) [file pmed.1003669.s012.pdf]

S9 Table Laboratory values at baseline and changes at day 7 and day 28.

Parameter	Day	Normal baseline ALT/AST (N=8367)		Abnormal baseline ALT/AST (N=158)		Unknown baseline ALT/AST (N=35)		Total (N=8560)	
		N	Mean (SD)	N	Mean (SD)	N	Mean (SD)	N	Mean (SD)
Erythrocytes, x10^{12}/L	Baseline	842	4.2 (0.6)	14	3.9 (0.8)	1	4.8	857	4.2 (0.6)
	Δ Day 7	8	−0.16 (0.13)	1	3.7	0	–	8	−0.16 (0.13)
	Δ Day 28	6	−0.32 (0.74)	0	–	0	–	6	−0.32 (0.74)
Hemoglobin, g/L	Baseline	8295	110.7 (19.2)	154	104.5 (21.0)	34	109.7 (18.5)	8483	110.6 (19.2)
	Δ Day 7	83	−6.1 (13.3)	3	−18.0 (12.1)	0	–	86	−6.5 (13.4)
	Δ Day 28	129	3.5 (16.3)	4	−0.25 (26.6)	1	−18.0	134	3.3 (16.2)
Hematocrit, fraction of 1	Baseline	842	32.4 (5.5)	14	28.1 (5.8)	1	27.3	857	32.3 (5.5)
	Δ Day 7	8	−1.5 (1.0)	1	21.8	0	–	8	−1.5 (1.0)
	Δ Day 28	6	−2.0 (6.4)	0	–	0	–	6	−2.0 (6.4)
Reticulocytes, x10^3/µL	Baseline	1	4.1	0	–	0	–	1	4.1
	Δ Day 7	0	–	0	–	0	–	0	–
	Δ Day 28	0	–	0	–	0	–	0	–
Platelets, x10^9/L	Baseline	842	176.6 (92.8)	14	124.3 (87.3)	1	317.0	857	176.0 (93.0)
	Δ Day 7	8	23.5 (101.4)	1	319.0	0	–	8	23.5 (101.4)
	Δ Day 28	6	−4.3 (129.1)	0	–	0	–	6	−4.3 (129.1)
Leukocytes, x10^9/L	Baseline	842	7.8 (3.2)	14	8.9 (4.5)	1	12.9	857	7.8 (3.2)
	Δ Day 7	8	−0.73 (4.3)	0	–	0	–	8	−0.73 (4.3)
	Δ Day 28	6	1.9 (4.6)	0	–	0	–	6	1.9 (4.6)
Neutrophils/leukocytes, %	Baseline	814	45.8 (18.0)	14	45.2 (21.4)	1	33.6	829	45.8 (18.0)
	Δ Day 7	6	−10.9 (16.6)	0	–	0	–	6	−10.9 (16.6)
	Δ Day 28	6	8.2 (18.2)	0	–	0	–	6	8.2 (18.2)
Eosinophils/Leukocytes, %	Baseline	822	6.8 (8.0)	14	1.4 (1.2)	1	23.3	837	6.8 (8.0)
	Δ Day 7	6	−1.1 (5.6)	0	–	0	–	6	−1.1 (5.6)
	Δ Day 28	6	−0.58 (2.6)	0	–	0	–	6	−0.58 (2.6)
Basophils/Leukocytes, %	Baseline	842	2.1 (2.3)	14	3.6 (2.4)	1	1.2	857	2.1 (2.3)
	Δ Day 7	8	−0.15 (0.67)	0	–	0	–	8	−0.15 (0.67)
	Δ Day 28	6	1.0 (2.7)	0	–	0	–	6	1.0 (2.7)
Lymphocytes/Leukocytes, %	Baseline	842	33.4 (14.2)	14	37.6 (16.2)	1	26.6	857	33.5 (14.3)
	Δ Day 7	8	6.0 (15.7)	1	22.8	0	–	8	6.0 (15.7)

Parameter	Day	Normal baseline ALT/AST (N=8367)		Abnormal baseline ALT/AST (N=158)		Unknown baseline ALT/AST (N=35)		Total (N=8560)	
		N	Mean (SD)	N	Mean (SD)	N	Mean (SD)	N	Mean (SD)
	Δ Day 28	6	−8.3 (16.7)	0	–	0	–	6	−8.3 (16.7)
Monocytes/Leukocytes, %	Baseline	833	11.6 (7.8)	14	12.3 (7.3)	1	15.3	848	11.6 (7.8)
	Δ Day 7	8	3.6 (8.3)	0	–	0	–	8	3.6 (8.3)
	Δ Day 28	6	−0.35 (8.1)	0	–	0	–	6	−0.35 (8.4)
Alanine aminotransferase, IU/L	Baseline	8367	12.8 (8.2)	158	100.7 (146.4)	0	–	8525	14.4 (24.5)
	Δ Day 7	1	−3.0	0	–	0	–	1	−3.0
	Δ Day 28	2	1.7 (2.4)	1	−12.6	0	–	3	−3.1 (8.4)
Aspartate aminotransferase, IU/L	Baseline	8367	28.7 (12.2)	157	182.6 (271.4)	0	–	8524	31.5 (43.9)
	Δ Day 7	1	−13.0	0	–	0	–	1	−13.0
	Δ Day 28	2	7.2 (10.1)	1	−103.3	0	–	3	−29.7 (64.2)
Bilirubin, µmol/L	Baseline	8108	14.0 (13.0)	154	18.5 (21.5)	0	–	8262	14.1 (13.3)
	Δ Day 7	1	1.5	0	–	0	–	1	1.5
	Δ Day 28	2	−0.86 (1.2)	1	4.8	0	–	3	1.0 (3.4)
Direct bilirubin, µmol/L	Baseline	5428	5.7 (5.2)	112	10.5 (16.1)	0	–	5540	5.7 (5.7)
	Δ Day 7	1	0	0	–	0	–	1	0
	Δ Day 28	2	0.86 (1.2)	0	–	0	–	2	0.86 (1.2)
Alkaline phosphatase, IU/L	Baseline	8095	184.5 (104.1)	154	258.0 (128.4)	0	–	8249	185.9 (105.0)
	Δ Day 7	1	1.0	0	–	0	–	1	1.0
	Δ Day 28	2	28.5 (40.3)	0	–	0	–	2	28.5 (40.3)

–, no observations. No subject had a baseline observation for sodium, potassium or glucose.
