# Supplementary material for: Pyronaridine–artesunate real-world safety, tolerability, and effectiveness in malaria patients in 5 African countries: A single-arm, open-label, cohort event monitoring study
Source: PLoS Med. 2021 Jun 15;18(6):e1003669. doi: 10.1371/journal.pmed.1003669 (PMC8205155; doi:10.1371/journal.pmed.1003669)
Supplement: S10 Table — (PDF) [file pmed.1003669.s013.pdf]

S10 Table Pyronaridine-artesunate day 28 unadjusted cure rate in African patients with acute uncomplicated malaria by *Plasmodium* species at baseline.

**Population**	**N**	**Unadjusted cure — no. (%)**	**95% CI**
Per-protocol population	7746	7221 (93.2)	92.6–93.8
P. falciparum	7744	7222 (93.3)	92.7–93.8
P. vivax	1	1 (100)	2.5–100
P. ovale	69	59 (85.5)	75.0–92.8
P. malariae	53	52 (98.1)	89.9–100
Intention-to-treat population	8480	7285 (85.9)	85.1–86.6
P. falciparum	8478	7286 (85.9)	85.2–86.7
P. vivax	1	1 (100)	2.5–100
P. ovale	70	59 (84.3)	73.6–91.9
P. malariae	55	52 (94.5)	84.9–98.9
