# Supplementary material for: Pyronaridine–artesunate real-world safety, tolerability, and effectiveness in malaria patients in 5 African countries: A single-arm, open-label, cohort event monitoring study
Source: PLoS Med. 2021 Jun 15;18(6):e1003669. doi: 10.1371/journal.pmed.1003669 (PMC8205155; doi:10.1371/journal.pmed.1003669)
Supplement: S11 Table — (PDF) [file pmed.1003669.s014.pdf]

S11 Table Pyronaridine-artesunate day 28 cure rate in African patients with acute uncomplicated malaria by category.

**Country**	**Per-protocol population**					**Intention-to-treat population**				
	**N**	**Unadjusted cure — no. (%)**	**95% CI**	**PCR-adjusted cure — no. (%)**	**95% CI**	**N**	**Unadjusted cure — no. (%)**	**95% CI**	**PCR-adjusted cure — no. (%)**	**95% CI**
All patients	7746	7221 (93.2)	92.6–93.8	7639 (98.6)	98.3–98.9	8480	7285 (85.9)	85.1–86.6	7705 (90.9)	90.2–91.5
Country										
Ivory Coast	1655	1515 (91.5)	90.1–92.8	1630 (98.5)	97.8–99.0	1756	1522 (86.7)	85.0–88.2	1637 (93.2)	91.9–94.4
Cameroon	771	694 (90.0)	87.7–92.0	756 (98.1)	96.8–98.9	951	701 (73.7)	70.8–76.5	763 (80.2)	77.6–82.7
Democratic Republic of Congo	2997	2827 (94.3)	93.4–95.1	2966 (99.8)	98.5–99.3	3165	2839 (89.7)	88.6–90.7	2979 (94.1)	93.2–94.9
Republic of Congo	676	664 (98.2)	96.9–99.1	670 (99.1)	98.1–99.7	693	666 (96.1)	94.4–97.4	672 (97.0)	95.4–98.1
Gabon	1647	1521 (92.3)	91.0–93.6	1617 (98.2)	97.4–98.8	1915	1557 (81.3)	79.5–83.0	1654 (86.4)	84.8–87.9
Formulation										
Granules	2925	2566 (87.7)	86.5–88.9	2849 (97.4)	96.8–97.9	3238	2598 (80.2)	78.8–81.6	2881 (89.0)	87.8–90.0
Tablets	4821	4655 (96.6)	96.0–97.1	4790 (99.4)	99.1–99.6	5142	4687 (89.4)	88.5–90.2	4824 (92.0)	91.3–92.7
Nutritional status										
Malnourished	370	358 (96.8)	94.4–98.3	370 (100)	99.0–100	404	361 (89.4)	85.9–92.2	373 (92.3)	89.3–94.7
Non-malnourished	7376	6863 (93.0)	92.4–93.6	7269 (98.5)	98.2–98.8	8076	6924 (85.7)	85.0–86.5	7332 (90.8)	90.1–91.4
Age group										
<1 year	130	111 (85.4)	78.1–91.0	126 (96.9)	92.3–99.2	141	113 (80.1)	72.6–86.4	128 (90.8)	84.7–95.0
≥ 1 year	7616	7110 (93.4)	92.8–93.9	7513 (98.6)	98.4–98.9	8339	7172 (86.0)	85.2–86.7	7577 (90.9)	90.2–91.5
