# Supplementary material for: Pyronaridine–artesunate real-world safety, tolerability, and effectiveness in malaria patients in 5 African countries: A single-arm, open-label, cohort event monitoring study
Source: PLoS Med. 2021 Jun 15;18(6):e1003669. doi: 10.1371/journal.pmed.1003669 (PMC8205155; doi:10.1371/journal.pmed.1003669)
Supplement: S1 Fig — (PDF) [file pmed.1003669.s015.pdf]

S1 Fig Mean number of days between malaria episodes treated with pyronaridine-artesunate (intention-to-treat population).

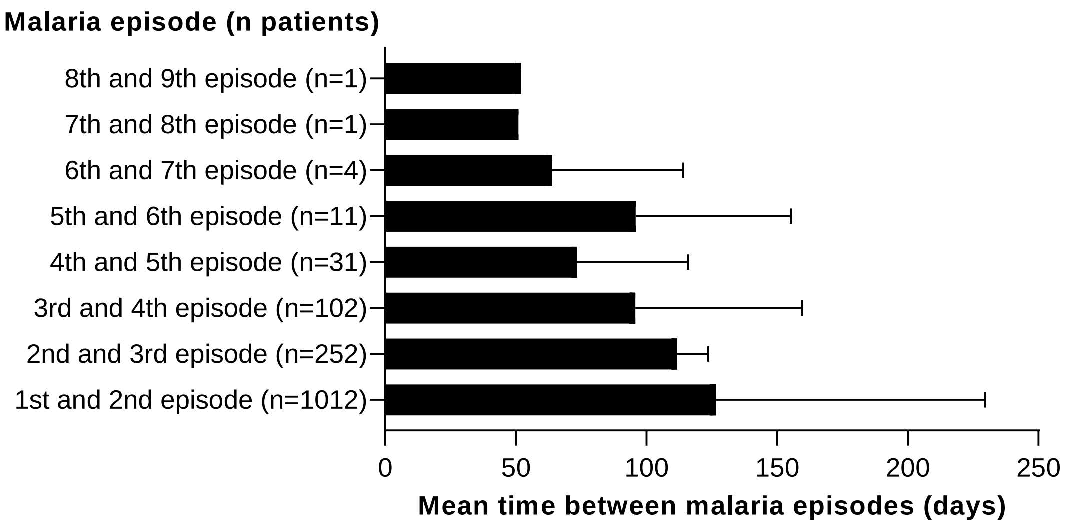

Malaria episode	Mean time (days)	SD	N patients
1st and 2nd episode	126.5	103.01	1012
2nd and 3rd episode	111.7	11.9	252
3rd and 4th episode	95.7	63.8	102
4th and 5th episode	73.4	42.54	31
5th and 6th episode	95.9	59.39	11
6th and 7th episode	63.8	50.31	4
7th and 8th episode	51		1
8th and 9th episode	52		1
